# Short-chain fructo-oligosaccharides supplementation to suckling piglets: Assessment of pre- and post-weaning performance and gut health

**DOI:** 10.1371/journal.pone.0233910

**Published:** 2020-06-05

**Authors:** Miriam Ayuso, Joris Michiels, Sander Wuyts, Honglin Yan, Jeroen Degroote, Sarah Lebeer, Cindy Le Bourgot, Emmanuelle Apper, Maryam Majdeddin, Noémie Van Noten, Charlotte Vanden Hole, Steven Van Cruchten, Mario Van Poucke, Luc Peelman, Chris Van Ginneken

**Affiliations:** 1 Department of Veterinary Medicine, Faculty of Pharmaceutical, Biomedical and Veterinary Sciences, University of Antwerp, Wilrijk, Belgium; 2 Department of Animal Sciences and Aquatic Ecology, Laboratory for Animal Production and Animal Product Quality, Faculty of Bioscience Engineering, Ghent University, Ghent, Belgium; 3 Department of Bioengineering, Faculty of Sciences, University of Antwerp, Wilrijk, Belgium; 4 R&D Department, Tereos, Moussy-Le-Vieux, France; 5 Department of Nutrition, Genetics and Ethology, Faculty of Veterinary Medicine, Ghent University, Merelbeke, Belgium; University of Illinois, UNITED STATES

## Abstract

Farmers face difficulties in redeeming their investment in larger litter sizes since this comes with larger litter heterogenicity, lower litter resilience and risk of higher mortality. Dietary oligosaccharides, given to the sow, proved beneficial for the offspring’s performance. However, giving oligosaccharides to the suckling piglet is poorly explored. Therefore, this field trial studied the effect of dietary short-chain fructo-oligosaccharides (scFOS; 1g/day; drenched) supplementation to low (LBW, lower quartile), normal (NBW, two intermediate quartiles) and high (HBW, upper quartile) birth weight piglets from birth until 7 or 21 days of age. Performance parameters, gut microbiome and short-chain fatty acids profile of feces and digesta were assessed at birth (d 0), d 7, weaning (d 21.5) and 2 weeks post-weaning (d 36.5). Additional parameters reflecting gut health (intestinal integrity and morphology, mucosal immune system) were analysed at d 36.5. Most parameters changed with age or differed with the piglet’s birth weight. Drenching with scFOS increased body weight by 1 kg in NBW suckling piglets and reduced the post-weaning mortality rate by a 100%. No clear difference in the IgG level, the microbiota composition and fermentative activity between the treatment groups was observed. Additionnally, intestinal integrity, determined by measuring intestinal permeability and regenerative capacity, was similar between the treatment groups. Also, intestinal architecture (villus lenght, crypt depth) was not affected by scFOS supplementation. The density of intra-epithelial lymphocytes and the expression profiles (real-time qPCR) for immune system-related genes (*IL-10*, *IL-1ß*, *IL-6*, *TNFα* and *IFNγ*) were used to assess mucosal immunity. Only IFNγ expression, was upregulated in piglets that received scFOS for 7 days. The improved body weight and the reduced post-weaning mortality seen in piglets supplemented with scFOS support the view that scFOS positively impact piglet’s health and resilience. However, the modes of action for these effects are not yet fully elucidated and its potential to improve other performance parameters needs further investigation.

## Background

During the last decades, pig selection has aimed at raising profitability by increasing litter size [1]. Unfortunately this increasing litter size generally comes with a higher mortality. The high mortality in these larger litters is attributed to the higher prevalence of low birth weight (LBW) piglets [2–4]. At birth and around the time of weaning, changes in the diet, environment and microbiome occur and the piglet’s gut has to rapidly adjust its digestive and immune functions [5]. These processes are not optimally coordinated in case of a LBW and in consequence these piglets have impaired gut functions even beyond weaning [5–10]. In addition, the LBW piglet has difficulties getting access to sufficient colostrum and milk [3] and has a lower feed intake after weaning [4, 11]. This poor milk and feed intake further aggravates their suboptimal gut health contributing to the high preweaning [2, 3] and postweaning [4] mortality rates observed in LBW piglets. Those LBW piglets that survive continue to have impaired gut functions [6–10] with a higher risk to develop intestinal disorders and infections, and show long-lasting suboptimal growth performance [2, 11–15]. Nevertheless, the farmer has the opportunity for supporting these piglets by either nutrition or management strategies, including a close surveillance of these piglets to increase their colostrum and milk intake [4, 16, 17]. Due to the aforementioned impact of gut health on resiliance and performance, optimizing and maintaining gut health [5] should be key in chosing the intervention to improve performance and survival. Dietary non-digestible oligosaccharides have proven beneficial in this respect. The health promoting effects of these oligosaccharides, such as fructo-oligosaccharides (FOS), are attributed to a shift in the maternal microbiome transmitted to the neonate at birth and a change in the immune and nutritional quality of the sow’s colostrum and milk in case of maternal supplementation [18–21]. In case of FOS given to nursery piglets, a direct impact on the microbiome is observed in addition to effects on the piglet’s intestinal morphology, gene expression and immune system maturation [18, 19, 22–25]. It is suggested that the latter effects on the immune system are mediated by a shift in the microbiota and the downstream effects of the short-chain fatty acids (SCFAs) that are produced via fermentation of FOS [18, 19, 23, 26]. The aforementioned shift in the microbiome consist of promoting the growth of beneficial bacteria while they are not used for the growth of potentially pathogenic bacteria, resulting in a decrease in some potentially harmful bacteria as *Clostridium perfringens*, *Clostridium difficile*, *Escherichia coli or Streptococcus thermophiles*, limiting their adhesion to the mucosa [27, 28]. In addition, some prebiotics can exert a direct antimicrobial effect, as they can adhere to the binding sites of bacteria on the enterocyte surface and thus, block the adhesion of pathogenic bacteria to intestinal epithelial cells [29, 30]. Next to promoting beneficial bacteria (i.e those who promote body functioning and health such as *Bifidobacterium* and *Lactobacillus*), regular supplementation with FOS leads also to the production of SCFA through fermentation of FOS [18, 19, 23, 26]. These SCFA having important metabolic functions and play a crucial role in intestinal health and immune cells [31, 32]. Indeed, microbiota-derived signals, including SCFA, influence the crosstalk between epithelial cells and gut dendritic cells, thereby modulating the nature and intensity of intestinal B and T cell responses. By this way, it has been demonstrated that FOS supplementation stimulated IgA secretion [33, 34], cytokine release (especially IFNγ) [33–35] and increased the numbers of natural killer cells and memory T cells [35].

Short-chain fructo-oligosaccharides (scFOS) have a lower degree of polymerization (DP)(DP between 3 and 5) when compared to the other FOS (DP 2–10) and inulin (DP 10–60). A relevant number of publications clearly displays that the growth of beneficial bacteria differs according to the type of prebiotic used, the growth being generally more effective and faster with the smaller polymers like scFOS than longer chains as oligofructose and inulin [36, 37]. In vitro experiments of fermentation with faecal inoculum have also shown that scFOS are more effective and quicker to stimulate SCFA production than long-chain prebiotics [38].

To our knowledge, the effects of short-chain fructo-oligosaccharides (scFOS) on the piglet’s gut health and performance by supplementing scFOS to piglets exclusively during the suckling period have not been investigated. Based on previous research using maternal and/or post-weaning scFOS supplementation [18, 19, 22, 26], scFOS given to the suckling piglet is expected to shape gut function and stimulate immune system maturation. This would improve the overall performance of these piglets resulting in increased weight gain and reduced mortality. However it is not clear how long the piglet needs to be supplemented to experience these benefits on gut health and performance. Therefore, two supplementation schemes were compared, i.e. scFOS supplementation from birth until 7 days *versus* supplementation from birth until 21 days of age. The effects of this supplementation on piglet performance (until 2 weeks after weaning), the piglet’s microbiome and various gut health parameters were studied in a field trial comprising piglets of different birth weights (BiW): low, normal, high. We hypothesized that 1) supplementation of scFOS improves gut microbiota, gut health and piglet’s performance in the suckling and nursery phase depending on length of administration during suckling, and 2) improvements are expected to especially benefit small piglets.

## Methods

### Animals, housing and experimental design

This experiment was approved by Ethics Committee of Faculty of Veterinary Medicine, Ghent University under the ID EC2016/79. The study was performed at a local farm (Kalmthout, Belgium). Seventeen sows from 2^nd^ to 8^th^ parity, with more than 14 live-born piglets (Hypor x Piétrain) and that farrowed within a range of 2 days, were included. A total of 157 piglets were selected at birth. Sows and piglets were housed in conventional farrowing units. During gestation, the sows were fed according to their body condition score and they received a standard diet during both the gestation (Aveve nr. 9037, pellet, AVEVE N.V., Merksem, Belgium) and lactation periods (Aveve nr. 9085, pellet, AVEVE N.V., Merksem, Belgium).

The day of the farrowing was set as d 0. After completion of the partus, all live-born piglets in each litter were weighed and assigned to one of three BiW categories: high BiW (HBW, BiW included within the upper quartile of the sample BiW), normal BiW (NBW, BiW between 75% and 25% of the sample BiW) and low BiW (LBW; (BiW included within the lower quartile). At birth, the piglets in each litter were randomly assigned to one of the three different treatments [control (CON), T1, and T2], considering BiW categories within the litter. This way, a uniform distribution of the treatments across litters and BiW categories was assured (p = 0.9). Mean (± SEM) weight per BiW category and treatment at the start of the experiment can be found in Table 3, first row (d 0). The pigs of the control group (CON; *n* = 53, 29 females, 24 males) were drenched daily with 2 mL of solvent (lukewarm tap water) from d 0 until d 7, by means of a dosing pipet, enlarged with a 7-cm flexible tube (5 mm diameter). A first treatment group (T1; *n* = 53, 28 females, 25 males) was drenched daily with 1 g of short-chain fructo-oligosaccharides (scFOS, Profeed L95: total scFOS: 95g/100g, Beghin-Meiji, Tereos, Marckolsheim, France) (dose based on [18, 22, 26, 34] where weaned pigs were fed *ad libitum* a diet supplemented with 0.15% scFOS equivalent to 1.2 g scFOS per pig per day)) dissolved in 2 mL solvent from d 0 until d 7. A second treatment group (T2; *n* = 51, 23 females, 28 males) was given 1 g scFOS (Profeed L95, Beghin-Meiji, Tereos, Marckolsheim, France) dissolved in 2 mL solvent from d 0 until weaning age (weaning age varied between 20 and 23 days of age) (d 21.5: mean age at weaning).

All pigs were allowed to suckle the dam until weaning. In addition, pigs had free access to supplementary milk (d 0 to d 7, Nuklospray®, Sloten, The Netherlands) and *ad libitum* creep feed (d 7 to d 15, Aveve nr. 9204, meal; d 15 to weaning (d 21.5), Aveve nr. 9105, pellet, AVEVE NV, Merksem, België).

At weaning, pigs representing the 3 treatments and having a normal BiW (from 8 out of the 17 litters; 24 piglets, one of which died before the last sampling point) were transferred to a separate nursery (Melle, Belgium), allocated in pairs (ensuring that piglets from the same treatment and litter were housed together) in 2 m^2^ pens and raised under standard conditions until sampling. Pigs were fed *ad libitum* and the ingredient and nutrient composition of the weaner diet in the nursery is given in the S1 File. No antibiotics, ZnO or any other medicaments were added to the feeds during the experimental period. These 20 pigs were euthanized at 2 weeks post-weaning (see sampling section). The remaining 137 pigs (comprising pigs belonging to the different BiW categories) were transferred to the nursery at the farm in Kalmthout. In the nurseries, the room was ventilated via a conventional scheme, with a starting ambient temperature of 30°C that was gradually decreased to 28°C at 15 d post-weaning. The light schedule was 23 h of light and 1 h of dark during the first 5 d and 16 h of light and 8 h of dark (1900 until 0300 h) afterwards. Cage enrichment was provided following EC requirements.

Performance data (body weight, colostrum intake, fecal score, average daily gain, mortality) were collected from the 137 pigs (comprising pigs belonging to the different BiW categories) that remained at the farm (Kalmthout, Belgium). Pigs were weighed (no restriction to feed or water) at d 0, d 1, d 2, d 7, d 21.5 (d 21,5 is used as the mean weaning age: piglets were weaned between 20 and 23 days of age) and two weeks after weaning (at ages varying between 34 and 37 days of age (d 36.5: mean age 2 weeks post weaning)). At 24h, colostrum intake was estimated using the formula of Theil et al. (2014) [39]. For different time periods, the average daily gain (ADG, kg/d), was calculated. All pigs were checked daily for health issues and a fecal score was given at pen level (Table 1). Mortality was reported as the percentage of dead pigs (number of pigs that died / number of pigs included in the group at the day of birth).

**Table 1:** Fecal consistency scoring.

Score	Description	Interpretation
0	No feces	Normal
1	Normal brown soft stool	Normal
2	Yellow or dark black (bloody) soft formed sticky feces	Indication of diarrhea
3*	Watery, stool or yellow or dark black (bloody) diarrhea.	Diarrhea
4	All piglets of a litter have score 3	Severe diarrhea

*In case of score 3, the number of piglets showing wet backsides (indicative for diarrhea) will be counted.

### Sampling

Fecal samples were taken from 8 pigs per treatment at d 7, d 21.5 and d 36.5. Fecal samples for microbiome determination were snap frozen and stored at -80°C pending further analysis. Fecal samples pending determination of short-chain fatty acids (SCFAs) were acidified with 2% (ratio H_2_SO_4_/feces volume) of a 6MH_2_SO_4_ solution, prior to storage at -20°C.

At d 36.5 (2 weeks post-weaning), the 20 pigs (CON: *n* = 7, 3 females, 4 males; T1: *n* = 8, 5 females, 3 males; T2: *n* = 8, 2 female, 6 males) that were transferred to the seperate nursery (see above), were euthanized by an overdose of intraperitoneal Na-pentobarbiturate (90 mg/kg) followed by exsanguination after which blood, digesta and tissue samples were collected.

Serum samples were allowed to clot for 20 min at room temperature. Plasma samples were collected in EDTA-tubes. Both samples were centrifuged at 4°C at 3,000 g for 10 min and stored at -20°C pending analysis for IgG and calprotectin.

Immediately after the pigs were euthanized, the abdomen was opened, the gastrointestinal tract was removed and the lengths of the small and large intestine recorded. The small intestine was equally divided into a proximal, middle and distal part and 5-cm segments were collected and flushed with saline. Two segments were immediately frozen in liquid nitrogen and stored at -80°C and two segments were fixated in 4% paraformaldehyde solution (0.1 *M*, pH 7.4) at room temperature for 2 h. Samples for next generation sequencing were taken from the different small intestinal regions, where 10 cm segments were flushed, the mucosa scraped, collected, transferred to vials and immediately immersed in liquid N_2_ and stored at -80°C pending further analysis. A small intestinal segment of approximately 20 cm was collected proximal to 75% of the total small intestinal length and immediately transferred to the Ussing chamber set-up to measure mucosal permeability.

Digesta pending determination of short-chain fatty acids (SCFAs) were collected from the most caudal 10% of the small intestine and from a 20-cm section distal to 50% of the total colon length. Samples were acidified with 2% of a 6 *M* H_2_SO_4_ solution, prior to storage at -20°C.

### Analysis of feces

Fecal samples were used for microbiome analysis, metabolic profiling (SCFAs analysis) and calprotectin measurement.

Out of the fecal samples collected at d 7, d 21.5 and d 36.5, DNA was extracted using the PowerFecal DNA-extraction kit (Qiagen, Hilden, Germany) according to the manufacturer’s instructions. DNA samples were quantified using a Qubit 3.0 Fluorometer (Thermo Fisher Scientific, Waltham, USA). Subsequent 16S rRNA sequencing was performed as described by Kozich et al. (2013) [40]. In summary, PCR amplicons resulting from a barcoded PCR assay were cleaned and equimolary pooled using the SequalPrep normalization kit (Invitrogen, Life Technologies, Grand Island, USA). The pooled amplicon library was loaded on a 0.8% agarose gel after which the amplicons were cut out and purified with the NucleoSpin gel and PCR clean-up kit (Macherey-Nagel, Düren, Germany). The final library was denatured with 0.2 N NaOH, diluted to 7 p*M*, spiked with 10% PhiX control DNA (Illumina, San Diego, USA) and subsequently sequenced using 2 x 250 cycles on the Illumina MiSeq sequencer. Sequences were processed using the DADA2 package, following the DADA2 standard operation protocol [41]. Sequences were classified using RDp version 14 [42] and the resulting sequence and taxonomy table were imported in to the Phyloseq package v1.18.1 [43] to calculate alpha and beta diversity and plotting of taxonomic profiles as described previously [44].

Fecal samples collected at d 36.5 were used for SCFA’s analysis and calprotectin measurements. To 1 g of sample, 5 mL 10% formic acid solution with 1 mg/mL 2-ethylbutyric acid as internal standard was added, vortexed and centrifuged at 1,200 g for 5 min. After filtration, the supernatant was injected for gas chromatographic separation to determine SCFA’s [45]. Fecal calprotectin levels were determined using a (human) calprotectin ELISA kit (CALPROLAB Calprotectin ELISA (ALP), CALPRO AS, Lysaker, Norway). Sample preparation was performed using a lower dilution factor than recommended in manufacturer’s guidelines, as calprotectin levels are expected to be lower in pigs than in human babies [46]. Briefly, fecal samples were thawed and 50 mg of feces were transferred to a 2 mL Eppendorf tube and suspended in 995 μl of extraction buffer (weight:volume ratio 1:20). Samples were sonicated (5 cycles of a 5 s pulse followed by 10 s off) on ice, subsequently put on a shaker for 30 min at 400 rpm, quick-spun down and the supernatants analyzed. All the samples, standards and controls were assayed in duplicate and expressed as μg/g feces.

### Analysis of blood samples

Plasma IgG content and calprotectin contents were determined using an IgG ELISA kit (Pig IgG ELISA kit, Alpha Diagnostic Intl., Texas, USA) and a calprotectin ELISA kit (CALPROLAB Calprotectin ELISA (ALP), CALPRO AS, Lysaker, Norway), respectively. Both kits were used according to the manufacturer’s instructions. No dilution was carried out in plasma samples prior to the calprotectin assay, whereas a final dilution of 1:100,000 was applied for the the IgG Elisa kit. All the samples, standards and controls were assayed in duplicate. The concentrations of IgG and calprotectin were expressed as ng/mL serum.

### Analysis of digesta

Lactic acid was assessed in microdiffusion cells following Conway [47] and expressed as μ*M*/g of fresh matter. The concentrations of SCFAs (acetate, butyrate, isobutyrate, proprionate, valeric acid and isovaleric acid) were determined by using gas chromatography according to the method used for the metabolic profiling of feces (see above) and described by Jin *et al*. 2017 [45]. The total and the individual SCFA concentrations in digesta were expressed as μ*M*/g of fresh matter.

### Mucosal permeability

Intestinal mucosal permeability was assessed *ex vivo* by measuring the translocation of macromolecular markers using the Ussing chamber technique. The 20-cm segments of the distal small intestine were rinsed with saline. The mucosal layer was stripped from the seromuscular layer and pinned onto 1.07 cm^2^ sliders that were mounted into Ussing chambers (Muβler Scientific Instruments, Aachen, Germany). Tissues were immersed in 6.5 mL Ringer solution (115 m*M* NaCl, 5 m*M* KCl, 25 m*M* NaHCO_3_, 2.4 m*M* Na_2_HPO_4_, 0.4 m*M* NaH_2_PO_4_, 1.25 m*M* CaCl_2_, 1 m*M* MgSO_4_) with 6 m*M* of mannitol or glucose in the luminal and serosal side, respectively. The system was water-jacketed to 37°C and oxygenated with 95% O_2_ and 5% CO_2_. After an equilibration period of 20 min, 4 kDa fluorescein isothiocyanate-dextran (FD-4, Sigma-Aldrich, Bornem, Belgium) was added to the luminal side to a final concentration of 0.8 mg/mL. Samples of the buffer solution were taken from the serosal chamber at 20, 40, 60 and 100 min after adding FD-4. Fluorescence intensity of FD-4 in the medium was measured at excitation wavelength of 485 nm and emission wavelength of 538 nm using a fluorescence plate reader (Thermo Scientific, Marietta, USA). The apparent permeation coefficient (Papp) was calculated as:

Papp (cm/s) = (dc/dt) × V/c_0_/A, where dc/dt is the change in the marker (FD-4) concentration at the serosal side (acceptor) between 20 and 100 min, (μg • mL^-1^ • s^-1^) calculated from the slope of the concentration–time curve, V is the buffer volume in the luminal side (donor) of the compartment (mL), C_0_ is the initial marker concentration in the donor compartment (μg • mL^-1^) and A is the exposed tissue surface area (cm^2^).

### Immunohistochemistry for tight junction proteins

Immunohistochemistry of the most relevant tight junction proteins [occludin (OCLN), zona occludens-1 (ZO-1) and claudin-2 (CLDN-2)] was performed on 4-μm, paraffin sections from the small intestine and the colon. Antigen retrieval was carried out for OCLN and ZO-1 by incubating with proteinase K (Dako; Glostrup, Denmark) for 1 min at 37°C. Then, the sections were incubated with 3% H_2_O_2_ dissolved in methanol, for 30 min and subsequently incubated with 10% (CLDN-2) or 20% (OCLN and ZO-1) normal goat serum (Jackson Immunoresearch Inc, West Grove, USA) dissolved in Tris-buffered saline (TBS) (0.01 *M* Tris, pH 7.4) for 30 min at room temperature. Subsequently, sections were incubated overnight at 4°C with polyclonal rabbit anti-OCLN (1/75, Invitrogen, Camarillo, USA), rabbit anti-ZO-1 (1/100 Santa Cruz Biotechnology Inc., Texas, USA), or rabbit anti-CLDN-2 antibody (1/50, Invitrogen, Camarillo, USA). All primary antibodies were diluted in TBS enriched with 0.3% Triton-X-100 (Sigma-Aldrich, Bornem, Belgium). After washing in TBS, sections were incubated with biotinylated goat anti-rabbit antibody (1/200 diluted in TBS enriched with 0.3% Triton-X-100 and 1% bovine serum albumin (BSA; ThermoFisher Scientific, Waltham, USA) for 30 min at room temperature. Following rinsing with TBS, sections were incubated for 30 min with streptavidin-conjugated horseradish peroxidase (sHRP) (1/200 diluted in TBS enriched with 0.3% Triton-X-100 and 1% BSA) (Agilent, Santa Clara, USA) at room temperature. After rinsing with TBS and demineralized water, tight junction proteins were visualized with 3,3’-diaminobenzidinetetrahydrochloride (Sigma-Aldrich, Bornem, Belgium). The presence of the different tight junction proteins was evaluated considering the number of positively stained sections, presence of staining in the crypts, base of the villus, mid villus and tip of the villus.

### Histomorphology

After deparaffinization, 4-μm sections were conventionally stained with haematoxylin-eosin (HE). The morphometric measurements [villus height, crypt depth, and number of intra-epithelial lymphocytes (IEL) per μm villus height (small intestine) or crypt depth (colon)] were performed on at least 15 sections per animal which resulted in measuring 30 longitudinally cut villi and their adjacent crypts (Olympus BX 61, analySIS Pro, Aartselaar, Belgium).

### Western blot analysis for regeneration capacity

Frozen samples of the proximal small intestinal region were homogenized in lysis buffer (50 m*M* Tris, 150 m*M* NaCl, 1% Nonidet P40 (vol/vol), 0.5% deoxycholate (wt/vol)) complemented with a complete protease inhibitor cocktail tablet (Roche Applied Science, Mannheim, Germany). Protein extracts (10 μg) were resolved by Mini protean TGX 4% to 15% gradient gels (Bio-Rad Laboratories, Hercules, USA) and transferred to a nitrocellulose membrane (Bio-Rad Laboratories, Hercules, USA). After blocking nonspecific protein binding with 5% nonfat dry milk in Tris buffer (10 m*M* Tris, 150 m*M* NaCl, 0.05% Tween-20, pH 7.6) for 1 h, blots were probed with anti-Proliferating Cell Nuclear Antigen (anti-PCNA) (1:5,000 in Tris buffer, Dakocytomation, Glostrup, Denmark) or anti-caspase-3 (1:1,000 in Tris buffer, Sigma-Aldrich, Bornem, Belgium). Subsequently, secondary antibodies were applied at room temperature: for PCNA a sHRP-conjugated goat anti-mouse antibody (1:5,000 in Tris buffer, Dakocytomation, Glostrup, Denmark) and for caspase-3, a sHRP-conjugated goat anti-rabbit antibody (1:1,000 in Tris Buffer, Dakocytomation, Glostrup, Denmark) was applied. Detection of a positive immunoreaction was performed using a chemiluminescence system (SuperSignal West Femto Maximum Sensitivity Substrate, Thermo Scientific, Rockford, Illinois, USA). Protein band intensities were quantified using densitometry (GeneSnap and GeneTools software, Syngene, Cambridge, UK). A normalized optical density (OD) was obtained by dividing the protein density by the density of the loading control β-actin (Sigma-Aldrich, Steinheim, Germany).

### Real time qPCR

RT-qPCR (*MUC2*, *IL1β*, *IL6*, *TNFα*, *INFγ*, *IL10*) was performed according to the MIQE guidelines [48]. In brief, mucosal total RNA was extracted using the Bio-Rad Aurum Total RNA Fatty and Fibrous Tissue Kit (Bio-Rad Laboratories, Inc., Hercules, USA) according to the manufacturer’s instructions, including an on-column DNase I treatment to remove genomic DNA (gDNA). The concentration and purity (OD_260/280_) of RNA were measured with the NanoDrop ND-1000 (NanoDrop Technologies, Thermo Scientific, Wilmington, USA). One μg RNA was analyzed by 1% agarose gel electrophoresis to check RNA integrity (28S and 18S rRNA bands). In addition, a minus-RT control PCR was performed using *YWHAZ* as primer to verify the absence of any gDNA contamination. Following this, 1 μg of high quality DNA-free RNA was reverse transcribed in the 20 μL reverse-transcription reaction with the ImProm-II cDNA synthesis kit (Promega, Madison, USA), containing both oligo dT and random primers. The obtained cDNA was diluted 10 times with molecular grade water and a control PCR using 2μL cDNA was performed to verify the reverse-transcription reaction.

Primers (Table 2) used for genes in the study were designed with Primer3Plus. The repeats, the secondary structure and single nucleotide polymorphism in the target sequence were checked with RepeatMarker, mfold and dbSNP, respectively. All these primer sequences were gene isoform specific as they were designed based on certain exon-exon boundaries of published pig gene sequences corresponding to the accession number. Primers were then purchased from IDT (Integrated DNA Technologies, Leuven, Belgium).

**Table 2:** Primer sequences used for reverse-transcription quantitative real-time PCR.

Gene symbol^1^	Accession number	Nucleotide sequence of primers, 5’-3’		Product length (bp)	Ta °C
		Forward	Reverse		
*MUC2*	XM_013989745	AGGACGACACCATCTACCTCACTC	GGCCAGCTCGGGAATAGACCTT	132	58
*IL-1β*	NM_214055.1	GCACCCAAAACCTGGACCT	CTGGGAGGAGGGATTCTTCA	143	58
*IL-6*	NM_214399.1	AGCCCACCAGGAACGAAAGAGAG	GGCAGTAGCCATCACCAGAAGCA	165	58
*TNF-α*	NM_214022.1	CATGATCCGAGACGTGGAGC	AACCTCGAAGTGCAGTAGGC	151	62
*IFN-γ*	NM_213948.1	GCTTTTCAGCTTTGCGTGACT	CACTCTCCTCTTTCCAATTCTTCA	166	58
*IL-10*	XM_013979620.1	GAAGACGTAATGCCGAAGGC	GCTGGTCTGCTACTCACACAG	122	62
*TLR-4*	NM_001113039.2	TTCTTGCAGTGGGTCAAGGA	GACGGCCTCGCTTATCTGAC	135	58
*IAP*	XM_003133729.3	GGCCAACTACCAGACCATCG	CCGACTTCCCTGCTTTCTTG	116	60
*ACTß*	XM_003124280.3	TCTGGCACCACACCTTCT	TGATCTGGGTCATCTTCTCAC	114	60
*TBP*	DQ178129	GATGGACGTTCGGTTTAGG	AGCAGCACAGTACGAGCAA	124	59
*TOP2ß*	NM_001258386.1	AACTGGATGATGCTAATGATGCT	TGGAAAAACTCCGTATCTGTCTC	137	60

^1^*MUC2*: mucin 2; *IL-1β*: interleukin 1 beta; *IL-6*: interleukin 6; *TNF-α*: tumor necrosis factor alpha; *IFNγ*: interferon gamma; *IL-10*: interleukin 10; *TLR-4*: toll-like receptor-4; *IAP*: intestinal alkaline phosphatase; *ACTß*: actin beta; *TBP*: TATA-binding protein; *TOP2ß*: DNA topoisomerase II beta.

The RT-qPCR was carried out on the CFX96 Touch Real-Time PCR Detection System (Bio-Rad Laboratories, Inc.). Briefly, 2μL cDNA template, 5 μL 2X KAPA SYBR FAST qPCR Kit Master Mix (Kapa Biosystems, Inc., Wilmington, USA), 2 μL molecular grade water, 0.5 μL forward primer and 0.5 μL reverse primer (5 μ*M* each) were added to a total volume of 10 μL. The amplification conditions were as follows: 1) enzyme activation and initial denaturation (95°C for 3 min); 2) denaturation (95°C for 20 s) and annealing/extension and data acquisition (annealing temperature depending on primer for 40 s) repeated 40 cycles; and 3) dissociation (melt curve analysis from 70 to 90°C with 0.5°C increment every 5 s).

The primers used in this study were first optimized by gradient quantitative real-time PCR. A 5-fold dilution series (5 points, from 1 to 625 times dilution) of cDNA as a standard curve was included at 3 gradient temperatures to determine PCR amplification efficiency and specificity. The standard curve was also included in each run to determine PCR efficiency. In this study, PCR amplification efficiencies were consistently between 90 and 110%. Gene-specific amplification was verified by agarose gel electrophoresis and melting curve analysis. Efficiency was used to convert the Cq value into raw data with the highest expressed samples (lowest Cq value) as a calibrator for the normalization of raw data. The relative expression was expressed as a ratio of the target gene to the geometric mean of 3 stable expressed reference genes (*ACTB*, *TOP2B* and *TBP*) [45, 49].

### Statistical analysis

The data from the piglets that remained at the farm were used for the analysis of performance parameters. For the fecal samples and the parameters determined using samples of piglets euthanized at d 15 post-weaning the data were not split up according to the BiW category since only NBW piglets were used.

Linear mixed models were fitted to evaluate the effect of treatment, age and BiW (performance data only) on the outcome variables (JMP pro 13.0). Fixed factors included treatment, age, BiW (performance data only) and the interaction between the different fixed factors. To account for the dependence between the observations on littermates, sow was included as a random factor. For the different measurements of body weight on the same pigs at different ages, piglet nested in sow was added as a random factor. Additionally, a random slope for age *vs* piglet nested in sow was checked. For the analysis of the digesta and the morphology samples that were collected at the level of the small and large intestine at the time of euthanasia, the fixed factor for age was replaced by sampling site (small *vs* large intestine) and the random slope sampling site vs piglet nested in sow was checked. The starting model was simplified using stepwise backwards modelling.

Alpha level used for significance determination ≤ 0.05. *Post-hoc* analysis with Tukey’s correction was used for the comparison of different treatments, ages and BiW categories. The fecal score was analyzed with the Chi Square test. Mortality rates between the sexes, BiW categories and treatments were analyzed via the non-parametric Kaplan-Meier method and *post-hoc* analysis was carried out via pair-wise comparisons using the Wilcoxon Rank test. Differences in alpha diversity of the gut microbiota were assessed using the Wilcoxon Rank test, while a t-test was used to estimate differences in mean relative abundance. Fecal calprotectin levels, lactic acid and proprionate levels in the digesta, and *MUC2* and *IL-6* mRNA ratios were analyzed using the non-parametric Wilcoxon Rank test. Data on small intestine length, relative to BW small intestinal length, total SCFA’s and butyrate in digesta, Papp of FD-4, number of IEL per μm villus or crypt, and *IAP*, *IL-10*, *IFN*γ mRNA ratios were log-transformed to meet normality and/or homoscedasticity assumptions. The piglet is considered the experimental unit. All data are presented as means ± SEM.

## Results

### Growth performance, colostrum intake and mortality

There was no effect on body weight between males and females (p = 0.7), and no interaction effect between sex and treatment (p = 0.4), sex and age (p = 0.4) and sex and BiW (p = 0.6). There was no interaction effect between treatment and age (p = 0.062) regarding body weight (Table 3). There was a significant interaction effect on body weight between age and BiW (p < 0.001). Therefore, differences in body weight between the six time-points (d 0, d 1, d 2, d 7, d 21.5, d 36.5) were analyzed within each BiW and the difference between the BiW was checked within each time point. In each BiW, body weight started to significantly increase after two days of life (LBW: p < 0.001; NBW: p = 0.008; HBW: p < 0.001) (Table 3). Additionally, at each time instance, the body weight of LBW was significantly lower than that of NBW and HBW (for all *post-hoc* comparisons). Yet, piglets belonging to NBW and HBW reached comparable body weights at 2 weeks post-weaning (p = 0.1) (Table 3).

**Table 3:** Body weight (BW; kg; mean ± SEM) in relation to supplementation with scFOS, age and birth weight category.

BW, kg	LBW^1^			NBW^1^			HBW^1^		
	CON^2^	T1^2^	T2^2^	CON^2^	T1^2^	T2^2^	CON^2^	T1^2^	T2^2^
	(*n =* 13)	(*n =* 12)	(*n =* 11)	(*n =* 21)	(*n =* 23)	(*n =* 21)	(*n =* 13)	(*n =* 11)	(*n =* 12)
**d 0**	**1.02 ± 0.02**^**a**^ ^**-**^ ^**x**^			**1.35 ± 0.01**^**a**^ ^**-**^^**y**^			**1.69 ± 0.02**^**a**^^**-**^^**z**^		
	1.02 ± 0.04	1.02 ± 0.03	1.03 ± 0.03	1.35 ± 0.03	1.35 ± 0.02	1.37 ± 0.03	1.68 ± 0.03	1.67 ± 0.02	1.72 ± 0.03
**d 1**	**1.07 ± 0.02**^**a**^ ^**-**^ ^**x**^			**1.43 ± 0.02**^**a**^ ^**-**^^**y**^			**1.75 ± 0.02**^**a**^ ^**-**^ ^**z**^		
	1.08 ± 0.04	1.07 ± 0.03	1.06 ± 0.04	1.44 ± 0.03	1.40 ± 0.03	1.45 ± 0.03	1.75 ± 0.03	1.72 ± 0.03	1.77 ± 0.05
**d 2**	**1.13 ± 0.03**^**a**^ ^**-**^ ^**x**^			**1.53 ± 0.02**^**a**^ ^**-**^^**y**^			**1.86 ± 0.03**^**a**^ ^**-**^ ^**z**^		
	1.14 ± 0.06	1.14 ± 0.04	1.10 ± 0.05	1.53 ± 0.04	1.50 ± 0.04	1.56 ± 0.04	1.87 ± 0.05	1.85 ± 0.06	1.86 ± 0.06
**d 7**	**1.68 ± 0.07**^**b**^ ^**–**^ ^**x**^			**2.26 ± 0.06**^**b**^ ^**-**^ ^**y**^			**2.71 ± 0.07**^**b**^ ^**-**^ ^**z**^		
	1.58 ± 0.14	1.76 ± 0.12	1.71 ± 0.13	2.16 ± 0.11	2.15 ± 0.09	2.47 ± 0.11	2.79 ± 0.11	2.67 ± 0.16	2.67 ± 0.11
**d 21.5**^**3**^	**4.52 ± 0.23**^**c**^ ^**-**^ ^**x**^			**5.61 ± 0.19**^**c**^ ^**-**^ ^**y**^			**6.26 ± 0.18**^**c**^ ^**–**^ ^**z**^		
	4.25 ± 0.58	4.71 ± 0.31	4.57 ± 0.40	5.03 ± 0.41	5.37 ± 0.27	6.36 ± 0.23	6.10 ± 0.36	6.59 ± 0.23	6.16 ± 0.27
**d 36.5**^**4**^	**6.62 ± 0.23**^**d**^ ^**-**^ ^**x**^			**8.36 ± 0.20**^**d**^ ^**–**^ ^**y**^			**8.74 ± 0.22**^**d**^ ^**-**^ ^**y**^		
	6.99 ± 0.31	6.99 ± 0.37	5.95 ± 0.35	7.99 ± 0.46	7.96 ± 0.30	8.98 ± 0.29	8.69 ± 0.36	8.97 ± 0.23	8.59 ± 0.48

^1^Birth weight categories (LBW: low birth weight; NBW: normal birth weight; HBW: high birth weights)

^2^Treatments: CON = Piglets drenched with 2 mL tap water until d 7; T1 = Piglets drenched with 1 g short-chain fructo-oligosaccharides (scFOS, Profeed P95, Beghin-Meiji) dissolved in 2 mL tap water until d 7; T2 = Piglets drenched with 1 g scFOS dissolved in 2 mL tap water until d 21.5 (weaning age)

^3^End suckling period

^4^2 weeks post-weaning

^a-d^Different superscripts within a column (birth weight category) indicate significantly different body weights between the time instants (p < 0.05).

^x-z^ Different superscripts within a row (time instants) indicate significantly different body weights between the birth weight categories (p < 0.05).

Finally, we found an interaction effect between treatment, age and BiW (p < 0.001) (Table 3). This means that any effect of scFOS supplementation on body weight differs between ages and BiW. Thus, in order to facilitate the understanding of our results, we looked at the interaction treatment and age in the different BiW categories. There was no significant effect in LBW and HBW piglets (p = 0.2 and p = 0.9, respectively). However, in NBW, the piglets supplemented lukewarm water (CON) and those supplemented scFOS for 7 days (T1) had lower body weights compared with those supplemented scFOS for 21 days (T2) at weaning and at the end of the experiment (p < 0.001 for the interaction effect) (Table 3). Alternatively, the interaction effect between treatment and BiW could mean that differences in body weight between piglets having other BiWs are not the same within each treatment group. This was not the case since *post-hoc* analysis revealed that in each of the treatment groups (CON: p < 0.001, T1: p < 0.001; T2: p < 0.001), the body weight of the LBW stayed below that of the NBW and HBW and that the body weight of NBW stayed below that of HBW (all *post-hoc* comparisons p < 0.001).

Regarding colostrum intake, no interaction effects were observed between sex and BiW (p = 0.2), between sex and treatment (p = 0.7) and between treatment and BiW (p = 0.9). As expected, sex (p = 0.4) (female: 326.8 ± 6.4 g; male: 326.6 ± 6.7 g) and treatment (p = 0.4) (CON: 325.6 ± 8.0 g; T1: 324.2 ± 7.7 g; T2: 330.5 ± 8.4 g) did not affect colostrum intake whereas BiW significantly did (p < 0.001). Colostrum intake was lowest in LBW (258.5 ± 3.6 g), intermediate in NBW (325.5 ± 3.0 g) and highest in HBW (391.5 ± 3.2 g) (for all *post-hoc* comparisons p < 0.001).

ADG, determined at the end of three different periods, was not affected by interaction effects between sex and BiW (d 0 to d 7: p = 0.3; suckling period: p = 0.087; 2 weeks post-weaning: p = 0.6), between sex and treatment (d 0 to d 7: p = 0.5; suckling period: p = 0.4; 2 weeks post-weaning: p = 0.6) nor between treatment and BiW (d 0 to d 7: p = 0.2; suckling period: p = 0.1; 2 weeks post-weaning: p = 0.3). Sex did not affect the ADG during the suckling period (d 0 to d 7: p = 0.2; female: 115.0 ± 6.8 g; male: 135.9 ± 8.6 g) (suckling period: p = 0.2; female: 187.3 ± 7.8; male: 205.6 ± 8.2 g) and during the 2 weeks post-weaning (p = 0.4; female: 150.5 ± 8.5 g; male: 143.2 ± 6.8 g). In addition, the ADG of the piglets was not affected by drenching scFOS (d 0 to d 7: p = 0.7; suckling period: p = 0.3; 2 weeks post-weaning: p = 0.2) (Table 4). However, ADG significantly differed when comparing piglets belonging to different BiW during the suckling period (d 0 to d 7: p < 0.001; suckling period: p < 0.001; 2 weeks post-weaning: p = 0.1). Throughout the suckling period, LBW gained less weight when compared with NBW and HBW (Table 4).

**Table 4:** Average daily gain (ADG) (g per day; mean ± SEM) at different ages and for different birth weight category and supplemented or not with scFOS.

ADG, g/d	LBW^1^			NBW^1^			HBW^1^			
	CON^2^	T1^2^	T2^2^	CON^2^	T1^2^	T2^2^	CON^2^	T1^2^	T2^2^
	(*n =* 13)	(*n =* 12)	(*n =* 11)	(*n =* 11)	(*n =* 23)	(*n =* 21)	(*n =* 13)	(*n =* 11)	(*n =* 12)
**d 0–7**	**95.2 ± 9.4**^**a**^			**127.9 ± 8.1**^**b**^			**145.3 ± 9.7**^**c**^			
	81.8 ± 19.2	105.6 ± 15.6	98.4 ± 13.8	116.1 ± 13.9	114.1 ± 10.5	154.3 ± 16.3	157.1 ± 14.2	141.4 ± 23.2	135.2 ± 14.3
**d 0–21.5**^**3**^	**167.2 ± 11.7**^**a**^			**198.3 ± 8.8**^**b**^			**213.5 ± 8.3**^**c**^			
	154.3 ± 29.0	176.4 ± 16.9	168.5 ± 17.0	172.0 ± 19.8	186.1 ± 12.2	233.8 ± 10.2	207.9 ± 16.8	229.3 ± 11.3	206.9 ± 12.7
**d 21.5–36.5**^**4**^	**116.7 ± 9.2**			**157.2 ± 7.1**			**155.0 ± 11.4**			
	119.2 ± 8.8	142.7 ± 8.9	86.2 ± 18.9	148.9 ± 18.3	156.4 ± 10.2	163.5 ± 10.7	161.9 ± 12.5	148.9 ± 19.5	152.1 ± 27.4

^1^Birth weight categories (LBW: low birth weight; NBW: normal birth weight; HBW: high birth weight).

^2^CON = Piglets drenched with 2 mL tap water until d 7; T1 = Piglets drenched with 1 g short-chain fructo-oligosaccharides (scFOS, Profeed P95, Beghin-Meiji) dissolved in 2 mL tap water until d 7; T2 = Piglets drenched with 1 g scFOS dissolved in 2 mL tap water until d 21.5 (weaning age)

^3^Suckling period

^4^Post-weaning period

^a-c^Different superscripts within a row (time instants) indicate a significantly different ADG between the birth weight categories (p < 0.05).

No clear signs of diarrhea (overall fecal score 1.72 ± 0.05, which is considered normal) were observed throughout the experimental period but differences were observed when comparing the fecal scores for the different BiW (p = 0.036). The mean fecal score of LBW (1.81 ± 0.08; p = 0.022) as well as HBW (1.74 ± 0.1; p = 0.042) were slightly higher when compared with NBW (1.67 ± 0.06). Drenching with scFOS did not affect the fecal score (CON = 1.71± 0.18; T1 = 1.84± 0.16; T2 = 1.60 ± 0.22; p = 0.9). There was no interaction between BiW and scFOS drenching (p = 0.5).

**Fig 1:**
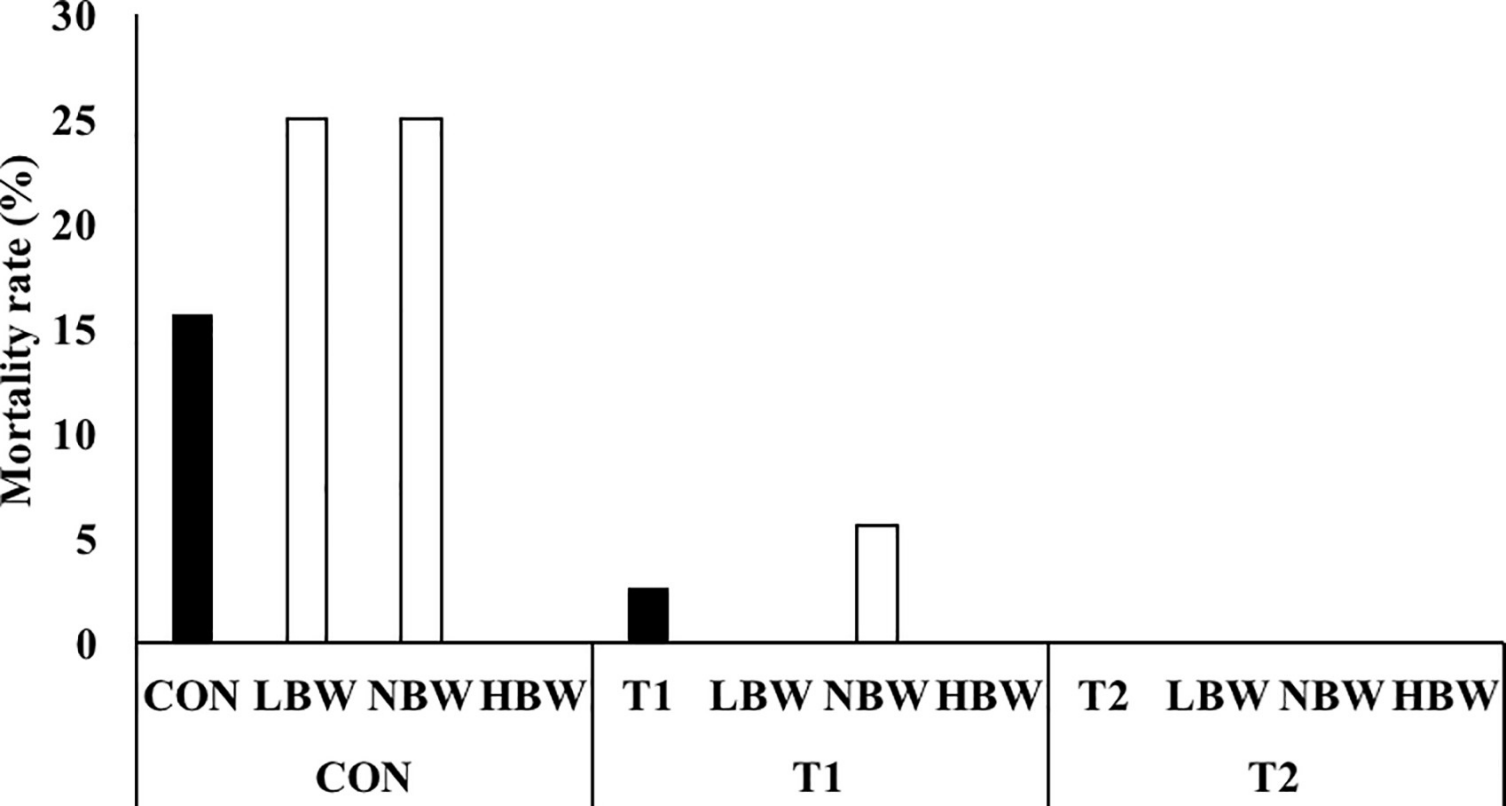
**Post-weaning mortality rate according to treatment (black bars) and according to birth weight category x treatment (white bars).** LBW (piglets with a birth weight in the lower quartile of the birth weight ranges), NBW (between 75% and 25%) and HBW category high (lower quartile) birth weight categories. CON represents piglets drenched with 2 mL tap water until d 7 (*n =* 13 (HBW), *n =* 21 (NBW) and *n =* 13 (LBW)); T1 represents piglets drenched with 1 g short-chain fructo-oligosaccharides (scFOS, Profeed P95, Beghin-Meiji) dissolved in 2 mL tap water until d 7((*n =* 12 (HBW), *n =* 23 (NBW) and *n =* 11 (LBW)) and T2 represents piglets drenched with 1 g scFOS dissolved in 2 mL tap water until d 21.5 (weaning age) (*n =* 11 (HBW), *n =* 21 (NBW) and *n =* 12 (LBW)).

The mortality rate was similar between sexes (p = 0.8; female: 20.8%, male: 21.5%). As expected, the overall mortality rate differed between the piglets with differing BiW (p = 0.048) where the mortality rate was lowest in HBW (8.3%) compared to NBW (24.2%) and LBW (30.6%). It needs to be noted that the average mortality rate in the farrowing unit at the farm was high during the experiment (16.5%). Given mortality in the first weeks after birth is mostly attributed to the starvation-crushing-hypothermia complex [3], and no treatment effect was observed on the pre-weaning mortality rates (when looking at overall treatment affect and treatment effect by BiW category), we focused on post-weaning mortality rates to discern a possible effect of treatment. In contrast to sex (p = 0.3; female: 8.1%; male: 3.8%) and BiW (p = 0.2; LBW: 7.4%; NBW: 9.6%; HBW: 0.0%), treatment had a significant effect on the post-weaning mortality rates (p = 0.025). *Post-hoc* analysis revealed that the post-weaning mortality rate was significantly lower in T2 (0.0%, p = 0.018), and numerically lower in T1 (2.6%, p = 0.082), when compared with the piglets that were not supplemented (CON: 13.9%) (Fig 1). Moreover, we investigated the effect of the treatment in each individual BiW category. In this regard, we did not observe any difference in heavy piglets (post-weaning mortality was 0% in all treatments), but we observed a trend in LBW piglets (two animals belonging to the CON group died, while there was no mortality in T1 and T2 groups; p = 0.072). A significant difference was observed in NBW piglets, with four out of the five deaths reported in the CON piglets and one out of five being in the T1 group; p = 0.027).

### Microbiota

**Fig 2:**
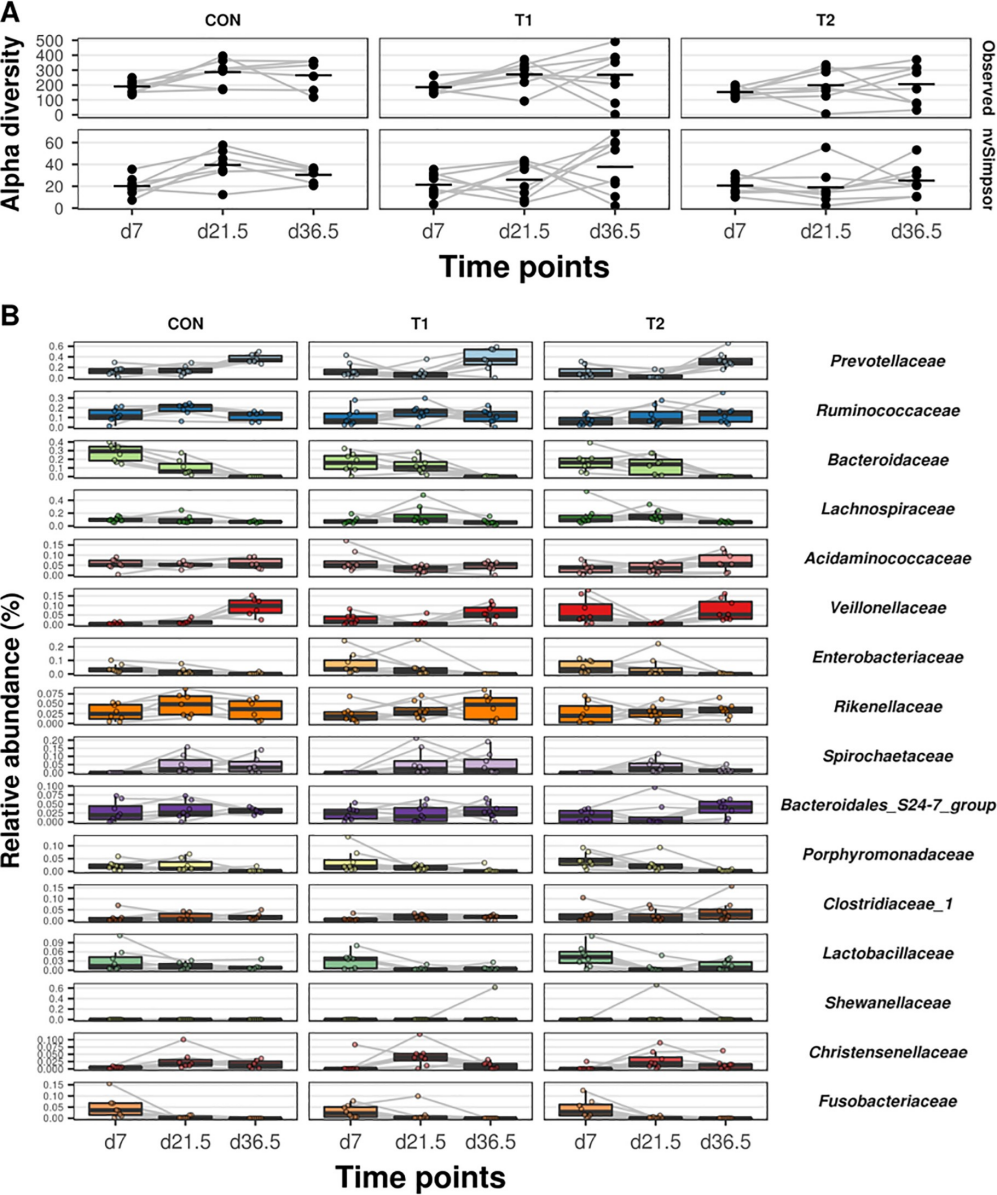
Alpha diversity (panel A) and the taxonomic profiles (panel B) of the microbiome determined in feces collected at d 7, d 21.5 and d 36.5 of life in CON (piglets drenched with 2 mL tap water until d 7) (*n =* 7); T1 (piglets drenched with 1 g short-chain fructo-oligosaccharides (scFOS, Profeed P95, Beghin-Meiji) dissolved in 2 mL tap water until d 7) (*n =* 8) and T2 (piglets drenched with 1 g scFOS dissolved in 2 mL tap water until d 21.5 (weaning age)) (*n =* 8). No differences were observed between the different treatment groups.

The alpha-diversity of the gut microbiota was not affected by the supplementation of scFOS (all comparisons p > 0.05; Fig 2A). However, the mean relative abundance of the main microbial species differed between the samples collected at different time points (d 7, d 21.5 and d 36.5) (Fig 2B). Fecal samples collected at d 7 were characterized by a relative high abundance of *Bacteroides* (d 7 vs d 36.5; CON: p < 0.001; T1: p = 0.004; T2: p = 0.004) and *Escherichia* (d 7 vs d 36.5; CON: p = 0.02; T1: p = 0.03; T2: p = 0.02) whereas at 2 weeks post-weaning (d 36.5) *Prevotella* (d 7 vs d 36.5; CON: p = 0.001; T1: p = 0.06; T2: p = 0.005) predominated over other species. The relative abundance of *Lactobacillus* spp. (d 7 vs d 36.5; CON: p = 0.2; T1: p = 0.04; T2: p = 0.04) decreased over time. *Bifidobacteriaceae* were only detected in a few samples.

In general, no effect of scFOS treatment on the mean relative abundance was found (all comparisons p > 0.05). However, compared to the control group, the mean relative abundance of *Bacteroides* was found to be temporarily reduced by scFOS supplementation in the first week of life in the T1 group only (p = 0.049; Fig 2B). Furthermore, a higher mean relative abundance for *Lachnoclostridium* was found at d 21.5 in the T2 group (p = 0.03; Fig 2B).

### Metabolic profiling: Analysis of short-chain fatty acids in fecal and digesta samples

**Fig 3:**
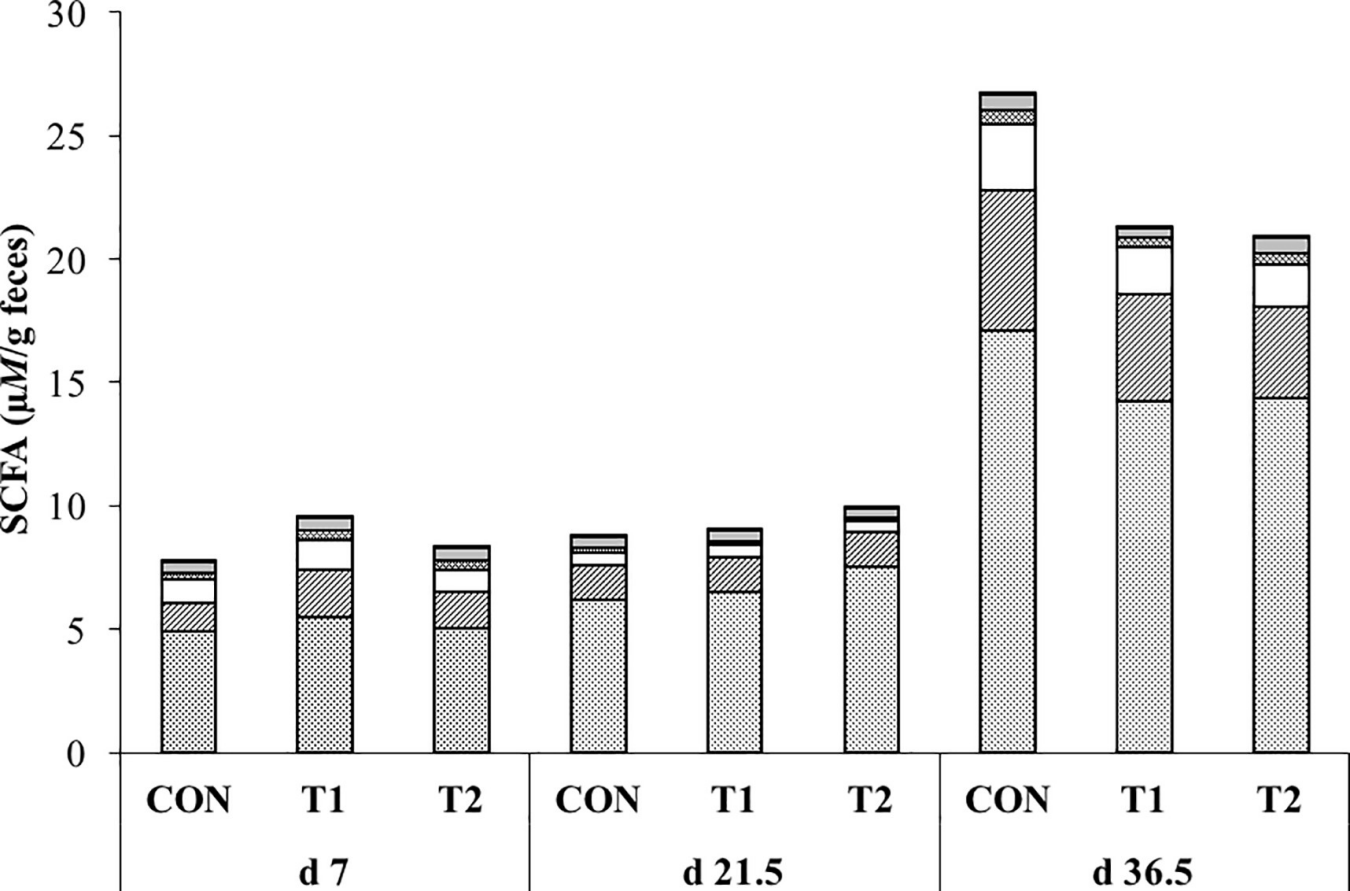
**Concentration (μ*M*) of short chain fatty acids (SCFA) per g feces at d 7, d 21.5 and d 36.5 of life: Acetate (dotted), proprionate (dashed), butyrate (white), valerate (double dashed), isovalarate (gray) and isobutyrate (black).** CON represents piglets drenched with 2 mL tap water until d 7 (*n =* 7); T1 represents piglets drenched with 1 g short-chain fructo-oligosaccharides (scFOS, Profeed P95, Beghin-Meiji) dissolved in 2 mL tap water until d 7 (*n =* 8) and T2 represents piglets drenched with 1 g scFOS dissolved in 2 mL tap water until d 21.5 (weaning age) (*n =* 8). No differences were observed between the different treatment groups. The concentration of the total SCFAs and its major constituents (acetate, proprionate, butyrate and valerate) was significantly higher after weaning. The concentrations of butyrate and valerate showed a dip at the end of the suckling period (d 21.5).

Short-chain fatty acids were quantified in 24 fecal samples taken at d 7, d 21.5 and d 36.5 (Fig 3). The total concentration of SCFAs was unaffected by drenching of scFOS (p = 0.2) but differed significantly when comparing different ages (p < 0.001). In this regard, the total concentration of SCFAs was significantly higher after weaning when compared with the levels seen during the suckling period (8.75 ± 0.67, 8.99 ± 0.54 and 23.00 ± 1.19 μ*M*/g feces on d 7, d 21.5, and d 36.5, respectively; p < 0.001). A similar observation was made for the individual SCFAs: acetate, proprionate, butyrate and valerate (Fig 3). None of the levels of these SCFAs differed between the treatment groups (acetate p = 0.5; proprionate p = 0.7; butyrate p = 0.3; valerate p = 0.8) and all of them reached higher concentrations 2 weeks post-weaning when compared to the values seen at day 7 and 21 (for all SCFAs p < 0.001). In addition to these changes with age, butyrate and valerate dropped to half of their levels in the feces of 1-week old piglets (p < 0.001) at the end of the suckling period (d 21.5) to then reach higher values at the end of the experiment. The concentration of the branched SCFAs isovalerate and isobutyrate, which are break-down products of amino acids rather than carbohydrates, were not affect by the treatment (isovalerate p = 0.5; isobutyrate p = 0.4). In contrast to the other SCFAs their levels remained constant throughout the experiment (isovalerate p = 0.3; isobutyrate p = 0.2).

Short-chain fatty acids were also analysed in digesta samples, which were collected from the distal small intestine and mid colon at the end of the experimental period (d 36.5) (Table 5). Treatment did not affect the total concentrations of SCFAs (small intestine p = 0.7; colon p = 0.8) nor that of the individual SCFAs (small intestine: acetate p = 0.8; proprionate p = 0.8; butyrate p = 0.9; mid colon: acetate p = 0.5; proprionate p = 0.9; butyrate p = 0.2; valerate p = 0.9), short-branched SCFAs (mid colon: isovalerate p = 0.6; isobutyrate p = 0.3) and lactic acid (small intestine: p = 0.4; mid colon p = 0.4) at any intestinal site (Table 5). Valerate and the branched SCFAs could not be detected in the digesta samples of the distal small intestine.

**Table 5:** Concentrations of short-chain fatty acids and lactate acid (mean ± SEM) in d 36.5 digesta of NBW piglets that received scFOS or not.

	CON^1^	T1^1^	T2^1^	*P*^*2*^
	(*n =* 7)	(*n =* 8)	(*n =* 8)	
**Distal small intestine**				
Total SCFA (μ*M*/g)	1.49 ± 0.23	1.43 ± 0.13	1.75 ± 0.29	0.71
Acetate (μ*M*/g)	1.34 ± 0.19	1.46 ± 0.20	1.64 ± 0.27	0.78
Proprionate (μ*M*/g)	0.031 ± 0.003	0.029 ± 0.007	0.027 ± 0.003	0.79
Butyrate (μ*M*/g)	0.065 ± 0.012	0.067 ± 0.012	0.078 ± 0.017	0.98
Lactic acid (μ*M*/g)	12.8 ± 2.8	19.9 ± 5.2	15.1 ± 3.4	0.41
**Mid colon**				
Total SCFA (μ*M*/g)	10.47 ± 0.12	10.30 ± 0.72	11.15 ± 0.71	0.79
Acetate (μ*M*/g)	6.84 ± 0.30	6.28 ± 0.42	6.93 ± 0.32	0.45
Proprionate (μ*M*/g)	2.67 ± 0.08	2.61 ± 0.23	2.82 ± 0.28	0.86
Butyrate (μ*M*/g)	2.13 ± 0.88	1.07 ± 0.16	1.26 ± 0.21	0.16
Valerate (μ*M*/g)	0.28 ± 0.14	0.17 ± 0.03	0.15 ± 0.02	0.88
Isobutyrate (μ*M*/g)	0.009 ± 0.001	0.007 ± 0.001	0.009 ± 0.001	0.33
Isovalerate (μ*M*/g)	0.120 ± 0.023	0.095 ± 0.016	0.120 ± 0.016	0.55
Lactic acid (μ*M*/g)	8.39 ± 0.14	8.13 ± 0.13	8.33 ± 0.27	0.42

^1^Treatments: CON = Piglets drenched with 2 mL tap water until d 7; T1 = Piglets drenched with 1 g short-chain fructo-oligosaccharides (scFOS, Profeed P95, Beghin-Meiji) dissolved in 2 mL tap water until d 7; T2 = Piglets drenched with 1 g scFOS dissolved in 2 mL tap water until d 21.5 (weaning age).

^2^*P*-value of the comparison between the 3 treatments.

### Calprotectin

Calprotectin concentration, a marker for intestinal inflammation, was determined in fecal samples taken at d 7, d 21.5 and d 36.5 and in blood samples collected at d 36.5. Calprotectin levels were below the detection limit in all blood samples and in fecal samples collected at d 7 and d 21.5. At 2 weeks post-weaning, calprotectin was only detected in some of the fecal samples. Given the high number of samples with values below the detection limit (these samples were recorded as 0 μg/g feces) and the resulting high variability, we failed to detect a statistically significant difference regarding the concentration of calprotectin between the different treatment groups in spite of the numerical difference (CON: 157 ± 76 μg/g feces (in 4/8 samples calprotectin was below the limit of detection); T1: 75 ± 29 μg/g feces (in 3/8 samples calprotectin was below the limit of detection); T2: 94 ± 38 μg/g of feces (in 4/8 samples calprotectin was below the limit of detection)) (p = 0.8).

### IgG

IgG levels in blood samples collected at 36.5 d were not different between the treatment groups (CON: 18.9 ± 3.3 ng/mL; T1: 27.9 ± 4.5 ng/mL; T2: 22.3 ± 4.4 ng/mL) (p = 0.2).

### Intestinal function: Morphometry, barrier function

Since small intestine dimensions are an important determinant for the digestive capacity of the gut, both macroscopic and microscopic parameters were determined on NBW samples taken on d 36.5.

The length of the small intestine did not differ between the treatment groups (CON: 1,022 ± 22 cm; T1: 985 ± 28 cm; T2: 1,086 ± 48 cm) (p = 0.2) nor was its relative length (expressed as cm/kg body weight at d 36.5) affected by the intake of scFOS (CON: 113 ± 6 cm/kg; T1: 146 ± 16 cm/kg; T2: 132 ± 7 cm/kg) (p = 0.093).

**Fig 4:**
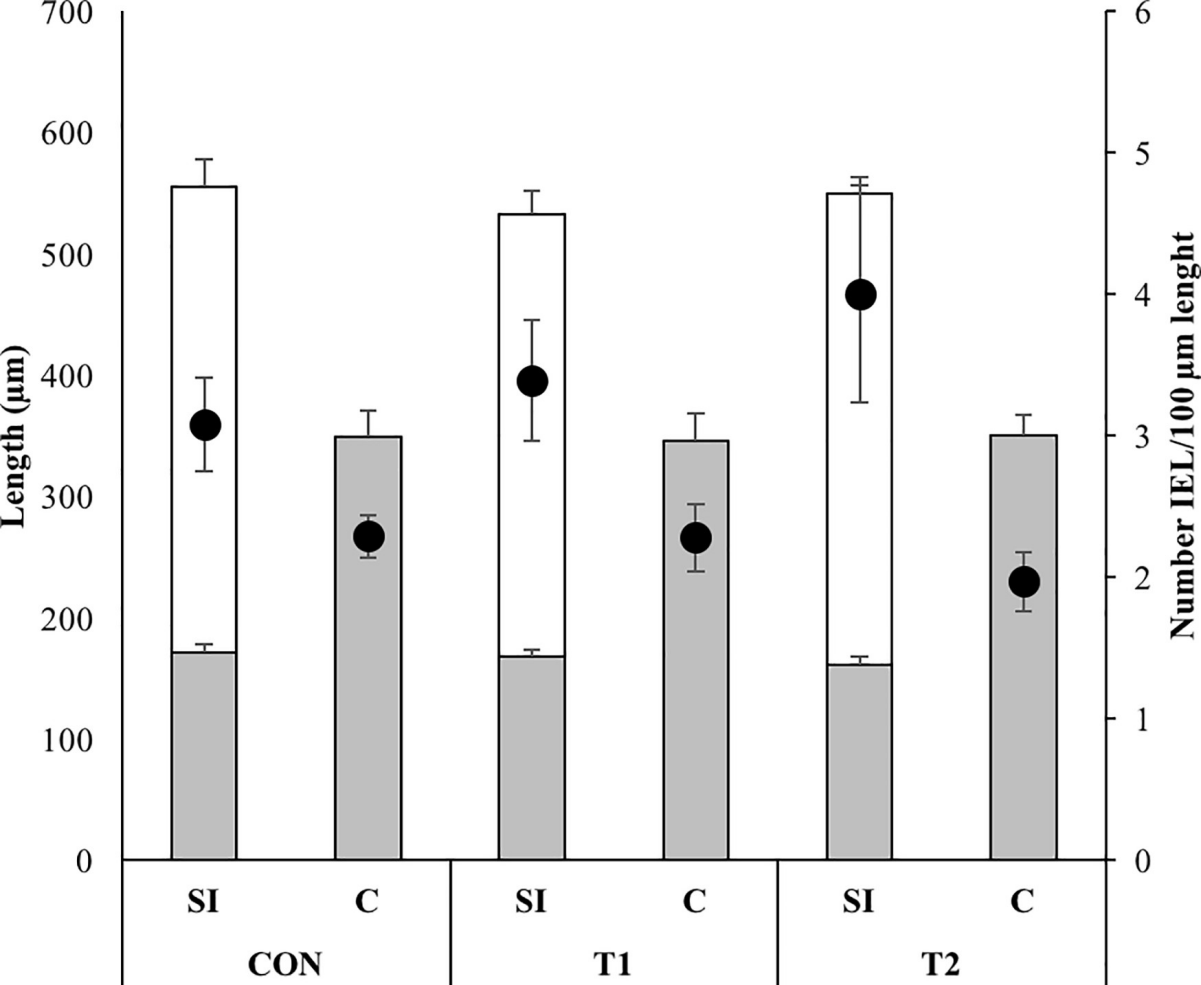
**Villus lenght (white bar; mean + SE), crypt depth (grey bar; mean + SE) and number of intra-epithelial lymphocytes (IEL) per 100 μm villus lenght (full circle; mean ± SE) in the distal small intestine (SI) and crypt depth and number of IEL per 100 μm crypt depth in the mid colon (C) of CON (piglets drenched with 2 mL tap water until d 7) (*n =* 7), T1 (piglets drenched with 1 g short-chain fructo-oligosaccharides (scFOS, Profeed P95, Beghin-Meiji) dissolved in 2 mL tap water until d 7) (*n =* 8) and T2 (piglets drenched with 1 g scFOS dissolved in 2 mL tap water until d 21.5 (weaning age) (*n =* 8)) at 36.5 days of age.** No differences were observed between the different treatment groups for villus length, crypt depth and IELs/100 μm.

Similarly, the length of the villi (p = 0.2) as well as the depth of the crypts at the level of the distal small intestine (p = 0.5) and of the mid colon (p = 0.9) of 36.5 day old piglets were not affected by the supplementation with scFOS (Fig 4).

Closely related to villus length and crypt depth is the ratio between mitosis and apoptosis. In this experiment an estimate of this ratio was carried out by determining the relative (to ß-actin) presence of proliferating cell nuclear antigen (PCNA) and caspase-3 (Fig 4). Both proteins had a higher relative abundance in the small intestine when compared to the large intestine (PCNA: p = 0.003; caspase-3: p = 0.009). Irrespective of intestinal location, none of them was affected by the supplementation with scFOS (PCNA: p = 0.6; caspase-3: p = 0.2).

**Fig 5:**
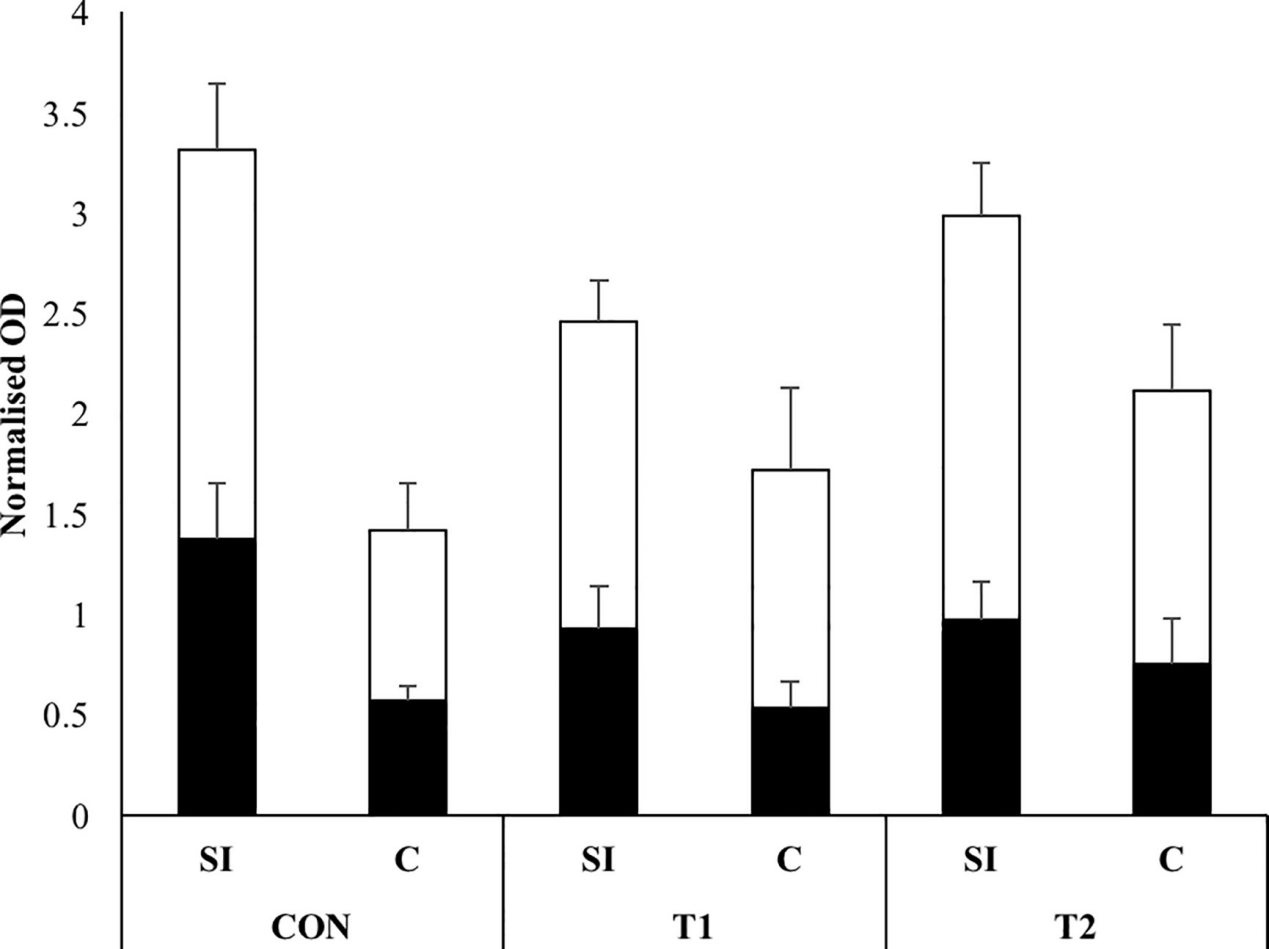
**Normalized OD (relative to ß-actin OD) of PCNA (black bar; mean + SEM) and caspase- 3 (white bar; mean + SEM) in the distal small intestine (SI) and in the mid colon (C) of CON (piglets drenched with 2 mL tap water until d 7; *n =* 7), T1 (piglets drenched with 1 g short-chain fructo-oligosaccharides (scFOS, Profeed P95, Beghin-Meiji) dissolved in 2 mL tap water until d 7; *n =* 8) and T2 (piglets drenched with 1 g scFOS dissolved in 2 mL tap water until d 21.5 (weaning age); *n =* 8) at 36.5 days of age.** No differences were observed between the treatment groups. The normalized OD of PCNA and caspase-3 were significantly higher in the small intestine when compared to the colon.

More IEL were present in the small intestine when compared with the colon (p < 0.001). This regional difference and the overall presence of IEL was unaffected by scFOS supplementation (p = 0.9) (Fig 5).

The intestinal barrier function was evaluated by the *ex vivo* translocation of FD-4 from the mucosal to the serosal side of the intestine. The Papp of FD-4 (2.35x10^-6^ ± 0.37x10^-6^ cm/s) did not differ between treatment groups and between the small and large intestine (Table 6).

**Table 6:** Effects of drenching with scFOS on apparent permeability for FITC-dextran, 4kDa (mean ± SEM) in distal small intestine and mid colon on day 36.5 of life of NBW piglets.

Papp FITC-dextran (cm/s 10^−7^)	CON^1^	T1^1^	T2^1^	p-value	p-value
	(*n =* 7)	(*n =* 8)	(*n =* 8)	Treatment	Location
**Distal small intestine**	21.9 ± 9.0	20.0 ± 5.8	16.6 ± 6.2	0.87	0.70
Mid colon	32.5 ± 15.4	23.2 ± 9.4	28.9 ± 9.2	0.85	

^1^Treatments: CON = Piglets drenched with 2 mL tap water until d 7; T1 = Piglets drenched with 1 g short-chain fructo-oligosaccharides (scFOS, Profeed P95, Beghin-Meiji) dissolved in 2 mL tap water until d 7; T2 = Piglets drenched with 1 g scFOS dissolved in 2 mL tap water until d 21.5 (weaning age).

Tight junctions seal enterocytes together and modulate transcellular movement of molecules. Immunohistochemical staining against key-constituents of the tight junction complex (OCLN, ZO-1 and CLDN-2) (S1 Fig) revealed the presence of tight junctions in the small and large intestine in the samples of the different treatment groups (data not shown). OCLN-immunoreactivity (IR) formed a more or less continuous band lining the villus and villus base. ZO-1-IR and CLDN-2-IR showed a more patchy staining which was limited to the villus. In the colon, OCLN-, ZO-1- and CLDN-2-IR were seen at the mucosa surface. OCLN-IR was present in the top half of the crypts. ZO-1-IR was also seen in cells in the lamina mucosae of the mid colon region.

### mRNA gene expressions

The mRNA gene expression profile in the intestine did not show any regional differences, nor was the effect of treatment region-dependent. Supplementation with scFOS modified the abundance of IFNγ mRNA (p = 0.025), which was significantly higher in the group of piglets that received scFOS for 7 days after birth compared to the piglets that were drenched with tap water. All other mRNA expression values were similar accross the treatment groups (Table 7).

**Table 7:** Effects of drenching with scFOS on the expression of mRNA (mean ± SEM) related to digestion and inflammation in NBW piglets.

Item^1^	CON^2^	T1^2^	T2^2^	*P*^*3*^
	(*n =* 7)	(*n =* 8)	(*n =* 8)	
*MUC2*	1.60 ± 0.30	1.17 ± 0.27	1.12 ± 0.22	0.4
*IAP*	1.07 ± 0.20	0.72 ± 0.18	1.28 ± 0.35	0.4
*IL-1ß*	1.06 ± 0.17	0.86 ± 0.13	0.95 ± 0.15	0.7
*IL-6*	1.41 ± 0.29	1.26 ± 0.30	1.23 ± 0.14	0.8
*IL-10*	0.82 ± 0.07	1.02 ± 0.12	1.00 ± 0.14	0.7
*IFNγ*	0.67 ± 0.15^a^	1.25 ± 0.21^b^	0.75 ± 0.12^a,b^	0.025
*TNFα*	1.38 ± 0.23	0.94 ± 0.11	1.13 ± 0.12	0.2
*TLR-4*	1.49 ± 0.24	1.30 ± 0.16	1.43 ± 0.17	0.5

^1^*MUC2*, mucin 2; *IL-1β*, interleukin 1 beta; *IL-6*, interleukin 6; *TNF-α*, tumor necrosis factor, alpha; *IFN-γ*, interferon gamma; *IL-10*, interleukin 10; *TLR-4*, toll-like receptor-4; *IAP*, intestinal alkaline phosphatase.

^2^Treatments: CON = Piglets drenched with 2 mL tap water until d 7; T1 = Piglets drenched with 1 g short-chain fructo-oligosaccharides (scFOS, Profeed P95, Beghin-Meiji) dissolved in 2 mL tap water until d 7; T2 = Piglets drenched with 1 g scFOS dissolved in 2 mL tap water until d 21.5 (weaning age).

^3^*P*-value of the comparison between the 3 treatments. Means with different superscript are significantly different (p ≤ 0.05).

## Discussion

Low birth weight piglets showed worse performance (as indicated by the results for body weight, colostrum intake, average daily gain and mortality) compared to NBW and HBW, which complies with literature [2, 12–15]. The causal relationship between BiW on the one hand and ADG on the other hand has been shown by various other reports [2, 3, 12–15] and explains why LBW piglets fail to catch up with their littermates. Nevertheless this does not preclude that some of the piglets born with a LBW level up their performance and catch up later during the nursing phase [4, 16, 17]. In agreement with this, we observed that 22% of the LBW piglets in this study reached a post-weaning weight that fell in the two intermediate quartiles, while a 17% of NBW piglets had their body weight within the upper quartile at the end of the experiment. Given this window of opportunity, we hypothesized that drenching of short-chain fructo-oligosaccharides (scFOS) would lead to a better performance, especially in the LBW piglets. However, scFOS supplementation did not impact performance during the experimental period in LBW piglets. On the contrary, in NBW piglets, a treatment effect was observed with an increase in the body weight with the higher duration of scFOS supplementation. Similar inconsistent results have been reported in the literature. For example, the direct supplementation of FOS to piglets during the lactation period has been shown to increase [24] [50], but also decrease [51] growth. It has also been demonstrated that maternal scFOS supplementation did not affect the growth of piglets during lactation [18, 22, 25, 26, 34] with controversial beneficial effects on growth after weaning [18, 22, 25, 26, 34]. These conflicting results indicate that the outcome of FOS supplementation may be affected by age, BiW, diet, administration route and even health status of the animals [52, 53]. In the present study, we accounted for some of these factors by including different BiW categories and different durations of supplementation during the suckling period (for 7 or for 21 days of age).

We observed rather high mortality rates (16.5%) in our experiment, especially during the suckling phase. This could be due to the use of highly prolific sows in the experiment (only litters with more than 14 piglets were included), with a large number of LBW pigs and a low average piglet BiW per litter, but also due to the fact that we limited piglet’s handling in order to not interfere with the scFOS treatment. This high pre-weaning mortality could not be reduced by supplementation with scFOS. One could conjecture, also considering the larger effects regarding growth were observed in NBW than LBW piglets, that the efficacy of scFOS is limited in very compromised piglets. The presence of high numbers of very compromised piglets during the suckling period–in our setting in the LBW group–may have reduced the chance to observe effects of scFOS supplementation. Alternatively, it could also be possible that scFOS need a longer supplementation time or higher concentration for positive effects to be observed (see later). Despite the lack of effects during suckling, one of the most interesting results found in our study, especially from an economic point of view, is the significant reduction in post-weaning mortality after scFOS supplementation for 7 or 21 days. This has been previously reported [54] and represents a clear gain for the pig industry. This effect was clearly observed in NBW piglets, in agreement with results in ADG and body weight, that pinpoints this BiW category as the most responsive to the treatment. However, LBW piglets seem to be responsive as well, with no piglets dying in the last 2 weeks of the experiment. It is striking that these differences are only observed in the postweaning period.

Interestingly, we did not observe any difference in mortality rate between BiW categories after weaning, contrary to the lower mortailty observed in suckling HBW when compared to LBW and NBW piglets. This a consequence of a reduction in mortality observed in all BiW categories, but especially in LBW (from 30.6% to 7.4% mortality) and NBW (from 24.2% to 9.6% mortality) piglets. This is most likely a consequence of the higher mortality observed in the first weeks of age, mostly attributed to the starvation-crushing-hypothermia complex [3], with the weakest piglets dying in this period and thus, not reaching the postweaning phase. These weakest piglets were probably too weak to benefit from possible beneficial effects of scFOS. Whereas those piglets which survived the suckling period, are reaching the threshold resilience that can be uplifted via scFOS resulting in a better post-weaning survival. In addition to performance parameters, and to gain more understanding on the mode of action of scFOS on performance and health, we studied the effects of scFOS on different parameters related to gut health.

The health-promoting effects of FOS are mostly linked to their direct bifidogenic effects [23, 24, 50]. Schokker et al. showed an increase in the abundance of *Bifidobacteria* in digesta samples after 10 days of supplementing both scFOS (9 g per day) and long-chain FOS (1 g per day) to suckling piglets [24]. Similarly, Shim et al. saw a rise of *Bifidobacteria* in digesta samples of suckling piglets fed creep feed rich in scFOS from day 7 for either 10 days or until weaning [50]. In our study, piglets received 1 g scFOS from birth until day 7 of life or until weaning and fecal samples were used to describe the composition of the microbiome. In both regimens, no significant difference in diversity and composition of the microbiome between the treatment and control groups were seen, not even for several taxa commonly associated with fiber consumption—including *Lactobacillus spp*., *Bifidobacteriaceae*, and butyrate-producing *Megasphaera*. Possible explanations for the absence of a detectable bifidogenic effect in this study could lay in the sample (feces vs digesta) and dosage of scFOS [52]. We opted for fecal samples to longitudinally follow the changes in the microbiome. In addition, the *Bifidobacteria* population in pigs is significantly lower than in humans, representing less than 0.1% of total sequences in fecal samples of 22-week-old pigs [55]. Although *Bifidobacteria* are usually considered the primary target, scFOS can additionally affect other gut bacteria. We observed a temporary decline in the relative abundance of *Bacteroides* when the piglets received scFOS and a significant increase with age in the presence of *Prevotella* which was unrelated to scFOS intake. Similar to our observations, Schokker and co-workers saw a temporary decline in the abundance of *Bacteroides* and a rise in *Prevotella* in the piglets fed scFOS [24]. In a study of Le Bourgot, where sows were supplemented with scFOS and the offspring received dietary scFOS up till 1 month after weaning, again a decrease in *Bacteroides* and a relative increase in *Prevotella* in the fecal microbiome of the suckling offspring was observed, which persisted into adulthood [34]. It should be noted that in both studies the intake of scFOS was either substantially higher in dosage [24] or longer [34] than in our study which could explain the minor and temporary changes observed in our study. Notwithstanding we did not observe a clear bifidogenic effect, the slight changes observed in the bacterial population reflect an adaptation of the microbiome to changes in the diet (these being scFOS supplementation or weaning) [56, 57].

Most of the effects of scFOS on gut health are attributed to the fermentation of the oligosaccharides into SCFA’s by the adapted gut microbiome [18, 19, 23, 24, 26, 56]. The main fermentation products of FOS are proprionate and acetate [34, 56, 58] that affect gut health via different mechanisms [34, 59]. Le Bourgot demonstrated that the increased proportion of *Prevotella* in suckling piglets of sows that receive scFOS resulted in increased concentrations of acetate, propionate but also valerate and caproate at weaning. However—in accordance with our results on the microbiome—the metabolic profile (detailing the concentrations of SCFAs) of digesta and fecal samples was not different between the treatment groups. Nevertheless, the concentrations of the SCFAs in feces changed with age, compliant with other reports and illustrating the relative increase in *Prevotella* in the gut’s microbiome and the adaptation to a diet high in carbohydrates [57].

The supplementation of scFOS was expected to affect different functional morphological characteristics of gut health by means of an increase or change in the composition of the SCFAs [59]. In our study both short- and long-term supplementation of 1 g scFOS did not affect intestinal integrity (determined by measuring intestinal permeability (FD-4-translocation in Ussing chambers), immunohistochemistry against occludin, ZO-1, claudin-2, and real time qPCR for MUC2), regenerative capacity (Western blot PCNA and caspase-3), and intestinal architecture (villus lenght, crypt depth). It should be mentioned these results were obtained in a subset of the piglets that all had a normal BiW. These results are consistent with the lack of a clear effect of scFOS on the microbiome and SCFAs production. Several studies that saw a bifidogenic effect of FOS in the pig, reported different effects on intestinal architecture [24, 26, 60].

Previous research has shown that FOS could exert microbiota-independent effects, on especially the maturing immune system [20]. In these studies it was shown that FOS given to young animals (infant mice or piglets) increased IgA secretion [33, 34], cytokine release (especially IFNγ) [33–35] and the number of natural killer cells and memory T cells [35] at the level of the intestine. These are interesting observations in view of the targeted decrease in post-weaning mortality via scFOS supplementation. Due to the lack or the very scarce effects on the microbiome, the intestinal morphology or its structural integrity, we hypothesize that scFOS excerted non-microbiome related effects that improved the maturation of the (intestinal) immune system. In order to get an idea of the impact of scFOS on the intestinal immune response, the serum levels of IgG, the density of intra-epithelial lymphocytes, and the expression profiles for several immune system-related genes (IL-10, IL-1ß, IL-6, TNFα and IFNγ) were estimated. Reports on the effects of FOS on the immune system under healthy conditions disagree. Most reports in pigs and mice clearly observed increased levels of immunoglobulins, cytokines and especially CD4 cells [33, 34, 61, 62]. In our study, we only observed a numerical increase in IgG levels (without joining significance) while the density of intra-epithelial lymphocytes was not differing between the treatment groups. Fukusawa and co-workers suggested potential genetic markers–of which interferon (IFN) was important—to study the immunomodulating effects of FOS [63]. Similar to others [63, 64], IFNγ mRNA levels were upregulated in piglets that received scFOS whereas the mRNA levels of the other cytokines remained unchanged. It is to be expected that the increased mRNA level of IFNγ leads to an increased release of this cytokine, since others already showed an increase concentration of IFNγ in ileal mucosa in case of maternal supplementation of scFOS [18, 26]. However, it is doubtable that the sole upregulation of IFNγ expression indicates an inflammatory status given the fact that we did not observe any indication of gastrointestinal dysfunction (i.c. no indication of diarrhea, no differences in the levels of calprotectin, which is an inflammatory marker). Increased IFNγ mRNA can rather indicate—as suggested by others—that scFOS partakes in the maturation of the immune system via a polarization of the Th1 immune response [18, 35, 65] which suggests a more mature immune system in scFOS-supplemented piglets. The combination of a better immunity in supplemented piglets together with the increased presence of *Prevotella* observed in all piglets included in this study, could drive the decrease in post-weaning mortality observed after a low dose supplementation of scFOS to suckling piglets. Moreover it should be mentioned that the increased presence of *Prevotella* is of interest since an enterotype rich in *Prevotallaceae*, *Lachnospiraceae*, *Ruminocacaceae* and *Lactobacillaceae* decreases the susceptibility to post-weaning diarrhea [57, 66].

In general, inconsistent results have been reported in literature regarding the beneficial effect of supplementation of scFOS to suckling and weaning piglets. In a study assessing the effects of scFOS supplementation to the sow and to the offspring on SCFA and other intestinal health markers, it was observed that no additional benefit was obtained when supplementation to the offspring was continued after weaning [23]. Thus, scFOS seem to be more effective when administered to the perinatal sow than to the offspring. In agreement with that, results reporting the prebiotic effects of FOS and mannan-oligosaccharides are more consistent when administered to the sow rather than the offspring [67, 68]. We can speculate that changes in the microbiome are more difficult in the suckling piglet. Also, there is limited information available regarding the structure and function of the gut microbiome of piglets in early-life in association with health and growth performance. It has been observed that bacterial populations remain similar after different rearing conditions (suckling vs formula) in the first 5 days of life, after which milk composition affected the piglets’ microbiome [69, 70]. Moreover, a sequential change in microbiome occurs during early life, with a very limited number of bacteria genera present in the first days of life that increase during the colonization process [71]. This reduced abundance in bacteria genera may limit the beneficial effects of scFOS in the early postnatal period. Also, the immaturity of the gut and the changes in gut morphology experienced during the first weeks of life could impair the use of prebiotics by the microbiome. However, previous studies demonstrated that formula-fed babies supplemented with oligosaccharides showed a change in the bacterial population [72, 73]. Another reason for the inconsistency of results could be the effect of the food matrix on the efficacy and bacterial use of these non-digestible fibers. In contrast with our experimental set-up, where piglets received scFOS independently of milk/creep feed consumption, results in babies where FOS was added directly to milk formula, report a more marked effect on bacterial populations [72, 73], although a similar set up in neonatal piglets only led to a trend in the increase of *Bifidobacteria* [74]. Also, rats given FOS in combination with probiotics showed a an increased gut permeability when fed a highly digestible but not a normal diet [53]. Regardless of the aforementioned constraints (i.e. dose, experimental conditions and administration route), our set up has the advantage of allowing the direct administration of a precise low dose to all the animals included in the study, independent of their daily food intake.

Based on the variety of experimental conditions and results, it seems several factors are implicated in the efficacy of scFOS in promoting (intestinal) health. Moreover, it cannot be ruled out that scFOS will exert a more pronounced effect when given at higher doses (as reported previously), a different administration route and/or under not optimal environmental conditions. Further studies are necessary to unravel the different mechanisms of action that may be implicated when scFOS are supplemented to adults and young animals. This may help to identify the right stage for scFOS supplementation (i.e. suckling versus weaning) based on the desired outcomes.

## Conclusions

Giving a low dose of scFOS (1 g/day per piglet) improved growth of suckling piglets and reduced post-weaning mortality to 0%, which supports the view that scFOS positively impact piglet’s health and resilience. However, the increased body weight after supplementation was only observed in piglets born with a body weight between the 25% and 75% quartiles (NBW) and not in LBW piglets, as was hypothesized. Moreover, the modes of action for these effects are not yet fully elucidated, since the effects of such dose on gut health in our experimental conditions are subtle. Further field research is granted in order to optimize scFOS dosage based on the expected outcome (i.e. mortality, ADG), the length of the treatment, the age and the handling/sanitary challenges to which piglets are exposed to.

## Supporting information

S1 Fig **A view on a paraffin section of a small intestinal sample of a piglet that received Profeed L95 (1 g scFOS per day and per pig) until weaning by drenching (left panel).** A positive immunohistochemical staining against occludin (1A), cloudin 2 (1B) and ZO-1 (1C) just below the brush border can be seen at the level of the villus tip (cloudin 2), and at the villus tip and body (occludin and ZO-1).
(TIF)

S1 File Feed ingredients and nutrient composition of weaner diet in the nursery phase. (DOCX)

S1 Data (PDF)

S2 Data (PDF)

S3 Data (PDF)

S4 Data (PDF)

S5 Data (PDF)

S6 Data (PDF)

S7 Data (PDF)

S8 Data (PDF)

S9 Data (PDF)

S10 Data (PDF)

S11 Data (PDF)

S12 Data (PDF)

S13 Data (PDF)

S14 Data (PDF)

S15 Data (PDF)

## Abbreviations

ACTß: actin beta
ADG: average daily gain
BiW: birth weight
BSA: bovine serum albumin
BW: body weight
CLDN-2: claudin-2
CON: control
FD-4: 4 kDa fluorescein isothiocyanate-dextran
FOS: fructooligosaccharides
HBW: heavy birth weight piglet
IAP: intestinal alkaline phosphatase
IEL: intra-epithelial lymphocytes
IFNγ: interferon gamma
IL: interleukin
LBW: low birth weight piglet
NBW: normal birth weight piglet
MUC2: mucin 2
OCLN: occludin
OD: optical density
OS: oligosaccharides
Papp: apparent permeation coefficient
PCNA: proliferating cell nuclear antigen
SCFA: short chain fatty acid
scFOS: short-chain fructo-oligosaccharides
SEM: standard error mean
sHRP: streptavidin-conjugated horseradish peroxidase
T: treatment
TBP: TATA-binding protein
TBS: Tris buffered saline
TLR-4: toll-like receptor-4
TNFα: tumor necrosis factor alpha
TOP2ß: DNA topoisomerase II beta
ZO-1: zonula occludens-1
