# Supplementary figures and images for: Short-chain fructo-oligosaccharides supplementation to suckling piglets: Assessment of pre- and post-weaning performance and gut health
Source: PLoS One. 2020 Jun 5;15(6):e0233910. doi: 10.1371/journal.pone.0233910 (PMC7274435; doi:10.1371/journal.pone.0233910)

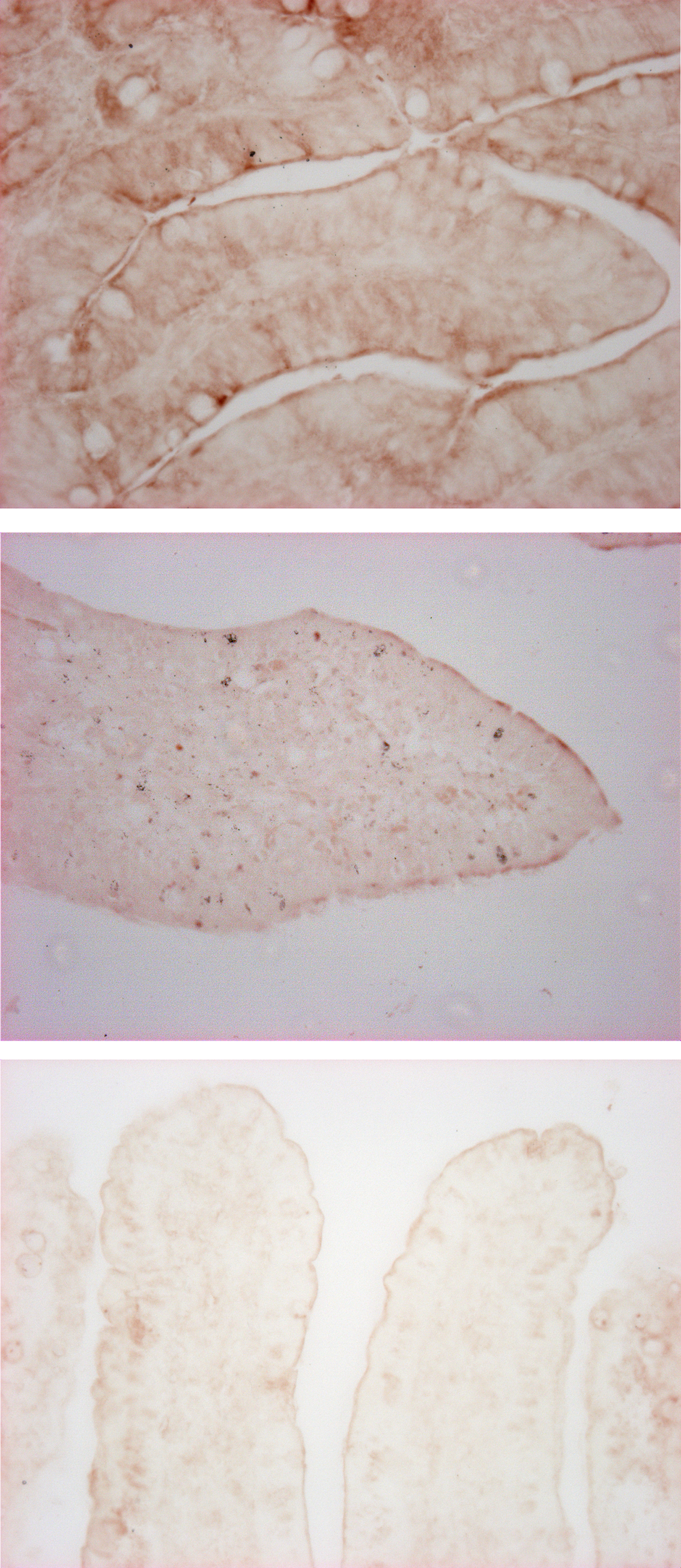

Supplement: S1 Fig — A view on a paraffin section of a small intestinal sample of a piglet that received Profeed L95 (1 g scFOS per day and per pig) until weaning by drenching (left panel). A positive immunohistochemical staining against occludin (1A), cloudin 2 (1B) and ZO-1 (1C) just below the brush border can be seen at the level of the villus tip (cloudin 2), and at the villus tip and body (occludin and ZO-1). (TIF) [file pone.0233910.s001.tif]
