# Supplementary material for: Short-chain fructo-oligosaccharides supplementation to suckling piglets: Assessment of pre- and post-weaning performance and gut health
Source: PLoS One. 2020 Jun 5;15(6):e0233910. doi: 10.1371/journal.pone.0233910 (PMC7274435; doi:10.1371/journal.pone.0233910)
Supplement: S1 File — (DOCX) [file pone.0233910.s002.docx]

## Supplementary file 1: Feed ingredients and nutrient composition of weaner diet in the nursery phase

| **Ingredients** | **Weaner diet (d0-14)** |
| --- | --- |
|  | **(%)** |
| BARLEY | 25,000 |
| CORN | 18,000 |
| WHEAT | 12,000 |
| SOYBEAN MEAL CP49 | 11,909 |
| TOASTED SOYBEANS | 12,000 |
| OAT FLAKES + EXTRUDED BARLEY | 10,000 |
| SWEET WHEYPOWDER | 4,000 |
| LACTOSE | 2,000 |
| SOYBEAN OIL | 0,689 |
| ANIMAL FAT |  |
| PREMIX TRACE MIN &VIT (*) | 1,000 |
| SUGAR BEET PULP | 1,000 |
| MONOCALCIUMPHOSPHATE | 0,210 |
| LIME | 0,764 |
| SODIUMBICARBONATE | 0,205 |
| LYSINE-HCL | 0,520 |
| SALT | 0,126 |
| DL-METHIONINE | 0,159 |
| L-THREONINE | 0,220 |
| L-VALINE | 0,121 |
| L-TRYPTOPHAN | 0,078 |
| SUM | 100,000 |
|  |  |
| **Calculated composition** | **Value** |
| Dry matter % | 88,7 |
| Ruw eiwit % | 17,8 |
| Ruw vet % | 5,00 |
| Zetmeel Ew. % | 36,9 |
| Suikers % | 7,92 |
| Suikers + zet % | 45,6 |
| Ruwe as % | 4,60 |
| Ruwe celstof % | 4,23 |
| NE Varkens KCAL | 2370 |
| Calcium % | 0,600 |
| Fosfor % | 0,410 |
| P verteerb. Va | 0,284 |
| Ca/Pv | 2,11 |
| Natrium % | 0,140 |
| Kalium % | 0,834 |
| Chloor % | 0,286 |
| Magnesium | 0,151 |
| EB (Na+K-Cl; meq/100g) | 195,0 |
| Lysine % | 1,27 |
| AID Lys Vark | 1,10 |
| AID Ile Vark | 0,567 |
| AID M+C Vark | 0,649 |
| AID Thr Vark | 0,682 |
| AID Trp Vark | 0,237 |
| AID Val Vark | 0,748 |
| AID Ile Vark/AID Lys Vark | 0,52 |
| AID M+C Vark/AID Lys Vark | 0,59 |
| AID Thr Vark/AID Lys Vark | 0,62 |
| AID Trp Vark/AID Lys Vark | 0,22 |
| AID Val Vark/AID Lys Vark | 0,68 |

(*) Providing per kg of diet: vit A (retinyl acetate), 15000 IU; vit D3 (cholecalciferol), 2000 IU; vit E (all-rac-alfa-tocopherylacetate), 50.0 mg; vit K3 (menadion), 4.0 mg; vit B1 (thiamine mononitrate), 3.1 mg; vit B2 (riboflavine), 8.0 mg; vit B3 (calcium-D-pantothenate), 20 mg; vit B6 (pyridoxine hydrochloride), 6.0 mg; vit B12 (cyanocobalamine), 50.0 µg; vit PP (niacinamide), 40.0 mg; folic acid, 2.0 mg; biotin, 0.3 mg; betaine anhydrate, 285 mg; endo-1,4-beta-glucanase E3.2.1.4, 250 TGU; endo-1,4-beta-xylanase E3.2.1.8, 560 TXU; 6-phytase, 500 OTU; Fe (iron(II)sulphate monohydrate), 24.0 mg; Cu (copper(II)sulphate pentahydrate), 155.0 mg; Zn (Zn MHA), 100.0 mg; Mn (manganese(II)oxide), 48.0 mg; I (calciumjodate anhydrate), 1.9 mg; Se (sodium selenite), 200 µg; Se (selenomethionine produced by *Saccharomyces cerevisae* NCYC-R397), 100 µg; E306 extract of vegetable oils rich in tocopherols, tocopherols, 228 mg; clinoptioliet, 1.64 g, aromatic compounds, 72 mg.
