# Supplementary material for: Short-chain fructo-oligosaccharides supplementation to suckling piglets: Assessment of pre- and post-weaning performance and gut health
Source: PLoS One. 2020 Jun 5;15(6):e0233910. doi: 10.1371/journal.pone.0233910 (PMC7274435; doi:10.1371/journal.pone.0233910)
Supplement: S3 Data — (PDF) [file pone.0233910.s005.pdf]

Image Report: B-actin_LADDER_analyse_Dd1-Dd13

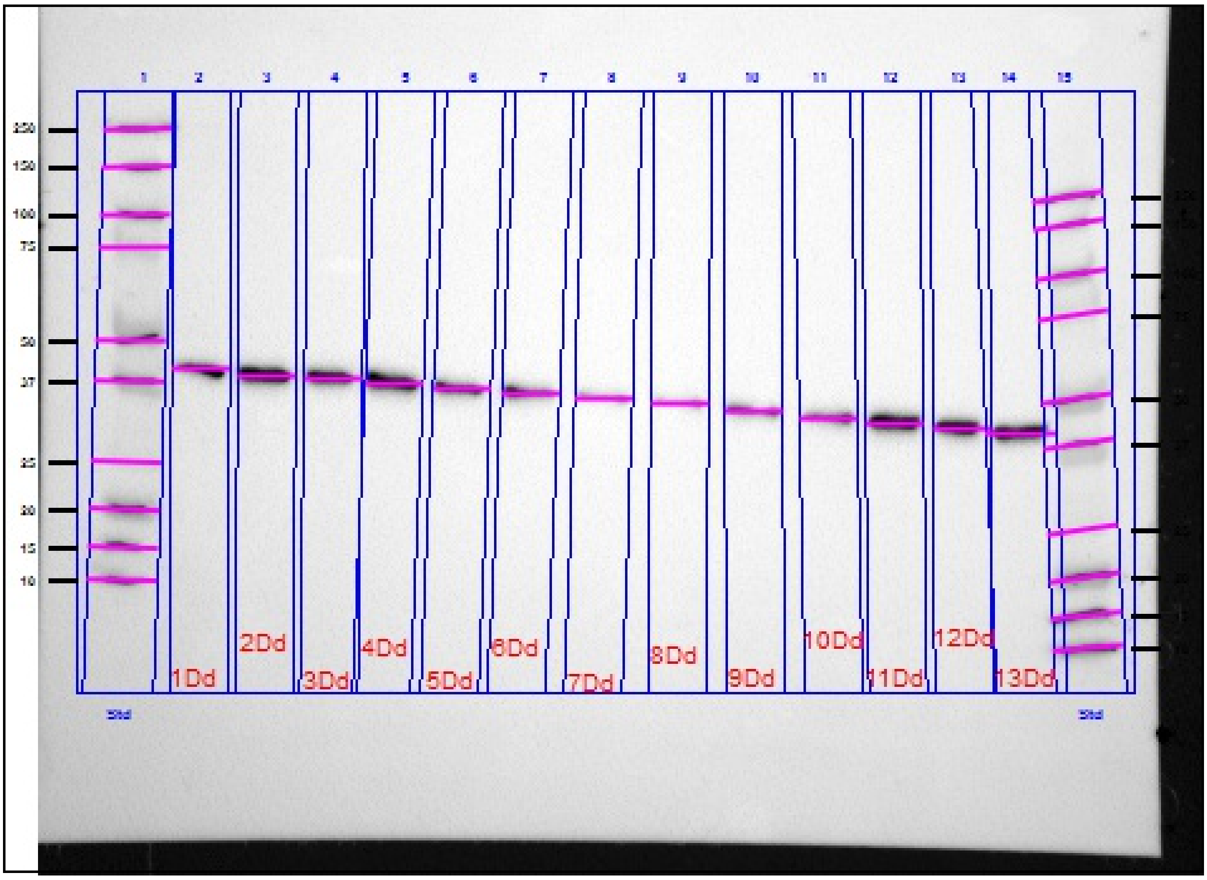

C:\Users\Bio-Rad\Desktop\Katty_Tereos\Dd28-32Dd\B-actin_LADDER_analyse_Dd1-Dd13.scn

Acquisition Information

Imager	Merged Image

Image Information

Acquisition Date	17/05/2017 13:14:43
User Name	Bio-Rad
Image Area (mm)	X: 95.0 Y: 71.0
Pixel Size (um)	X: 204.7 Y: 205.1
Data Range (Int)	153 - 63745

Notes

Merged images:
Image 1: B-actin6_LADDER
Image 2: B-actin2+B-actin6

Analysis Settings

Detection	Lane detection:<br>Manually created lanes<br><br>Band detection:

	Manually adjusted bands<br><br>Lane Background Subtraction:<br>Lane background subtracted with disk size: 10<br><br>Lane width: Variable
Mol. Weight Analysis	Standard: Bio-Rad Precision Plus<br>Standard lanes: first last<br>Regression method: Point to Point (semi-log)

Lane And Band Analysis

Lane 1 - Bio-Rad Precision Plus

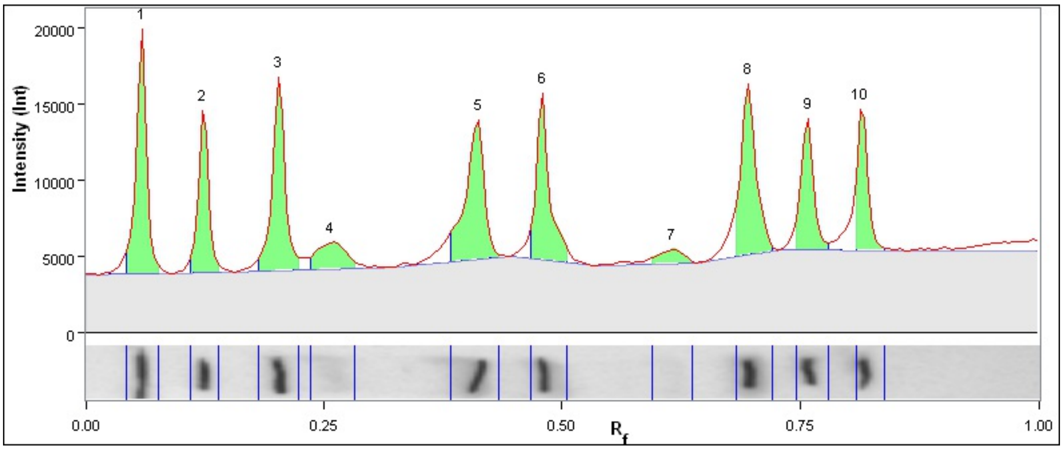

Band No.	Band Label	Mol. Wt. (KDa)	Relative Front	Volume (Int)	Abs. Quant.	Rel. Quant.	Band %	Lane %
1		250,0	0,063	1.528.828	N/A	N/A	14,3	12,0
2		150,0	0,126	1.016.008	N/A	N/A	9,5	8,0
3		100,0	0,206	1.488.480	N/A	N/A	13,9	11,7
4		75,0	0,261	457.660	N/A	N/A	4,3	3,6
5		50,0	0,416	1.517.992	N/A	N/A	14,2	11,9
6		37,0	0,483	1.293.488	N/A	N/A	12,1	10,1
7		25,0	0,618	202.132	N/A	N/A	1,9	1,6
8		20,0	0,697	1.471.568	N/A	N/A	13,7	11,5
9		15,0	0,761	959.028	N/A	N/A	9,0	7,5
10		10,0	0,815	776.440	N/A	N/A	7,2	6,1

Lane Background	Lane background subtracted with disk size: 10
Lane Width	5.73 mm
Regression Equation	A single equation is not available for this method

Lane 2

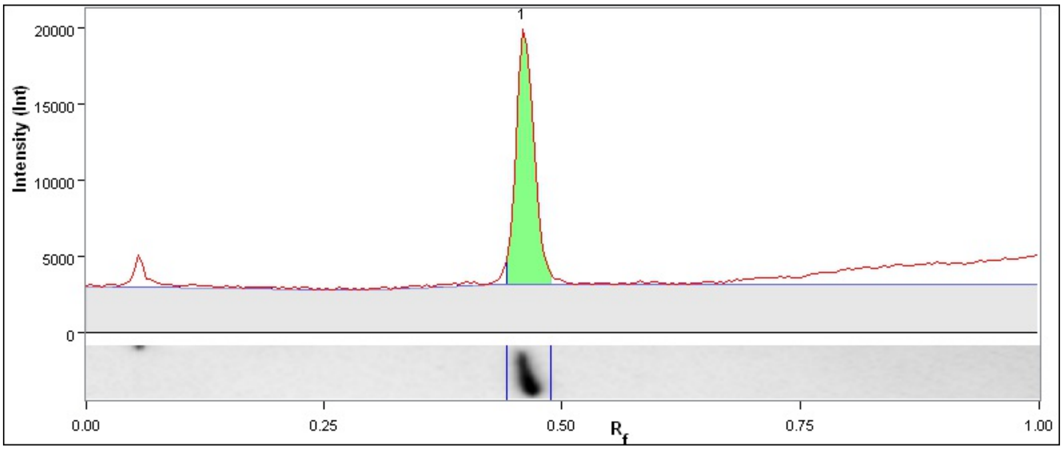

Band No.	Band Label	Mol. Wt. (KDa)	Relative Front	Volume (Int)	Abs. Quant.	Rel. Quant.	Band %	Lane %
1		41,9	0,462	2.411.343	N/A	N/A	100,0	44,4

Lane Background	Lane background subtracted with disk size: 10
Lane Width	4.71 mm
Regression Equation	A single equation is not available for this method

Lane 3

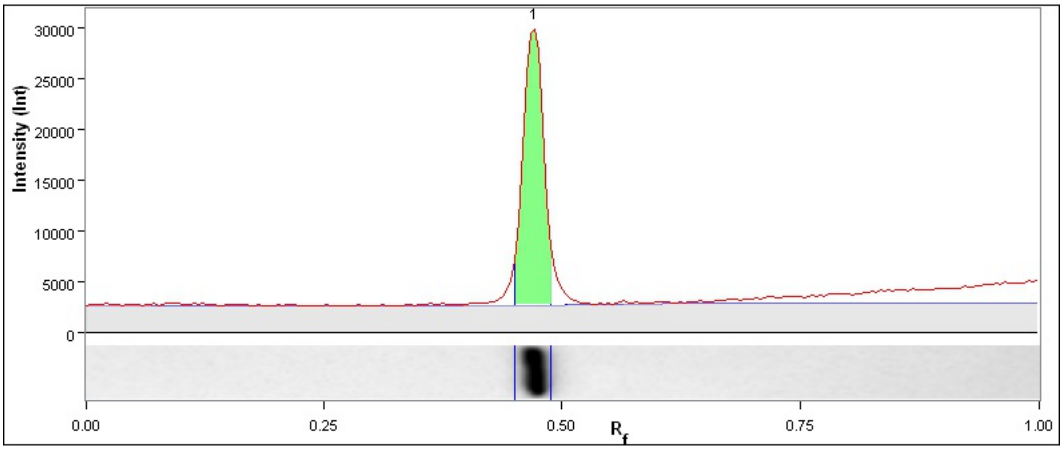

Band No.	Band Label	Mol. Wt. (KDa)	Relative Front	Volume (Int)	Abs. Quant.	Rel. Quant.	Band %	Lane %
1		40,9	0,475	3.974.906	N/A	N/A	100,0	56,1

Lane Background	Lane background subtracted with disk size: 10
Lane Width	4.71 mm
Regression Equation	A single equation is not available for this method

Lane 4

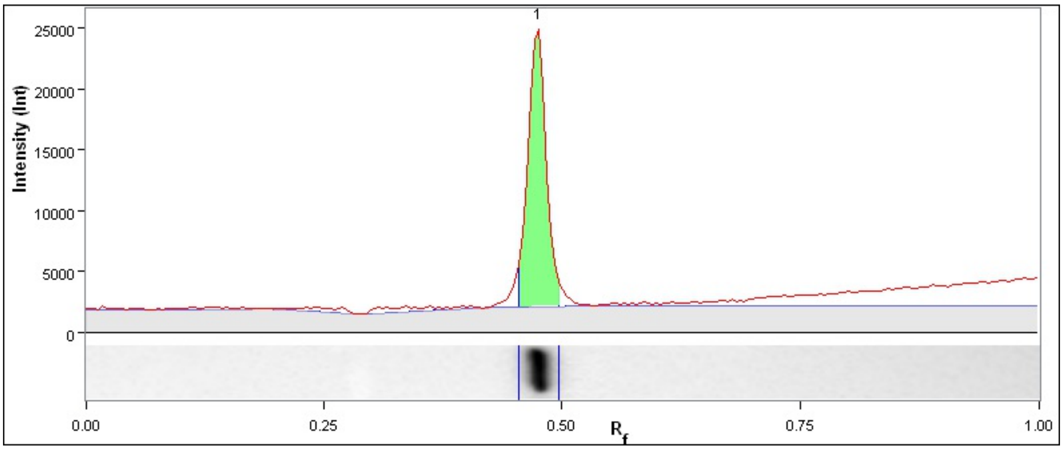

Band No.	Band Label	Mol. Wt. (KDa)	Relative Front	Volume (Int)	Abs. Quant.	Rel. Quant.	Band %	Lane %
1		41,5	0,479	3.074.341	N/A	N/A	100,0	45,8

Lane Background	Lane background subtracted with disk size: 10
Lane Width	4.71 mm
Regression Equation	A single equation is not available for this method

Lane 5

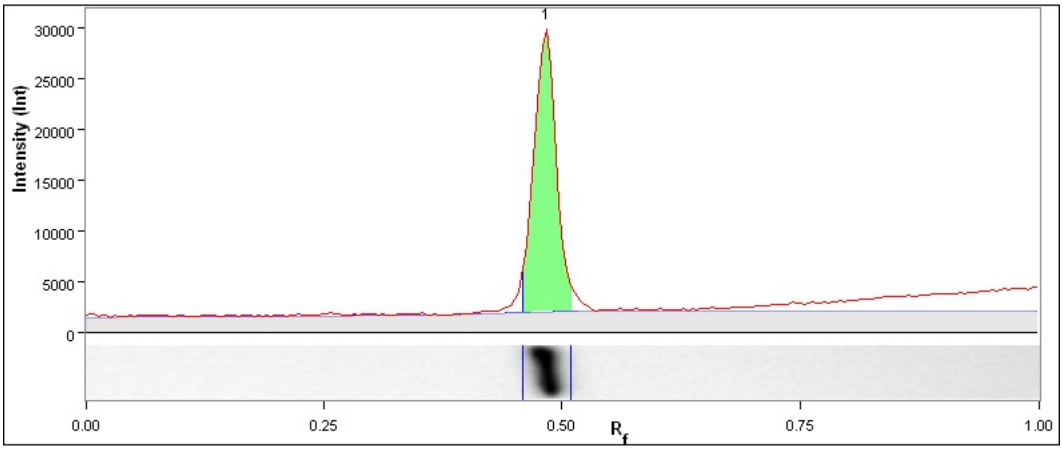

Band No.	Band Label	Mol. Wt. (KDa)	Relative Front	Volume (Int)	Abs. Quant.	Rel. Quant.	Band %	Lane %
1		41,4	0,487	4.163.782	N/A	N/A	100,0	58,6

Lane Background	Lane background subtracted with disk size: 10
Lane Width	4.71 mm
Regression Equation	A single equation is not available for this method

Lane 6

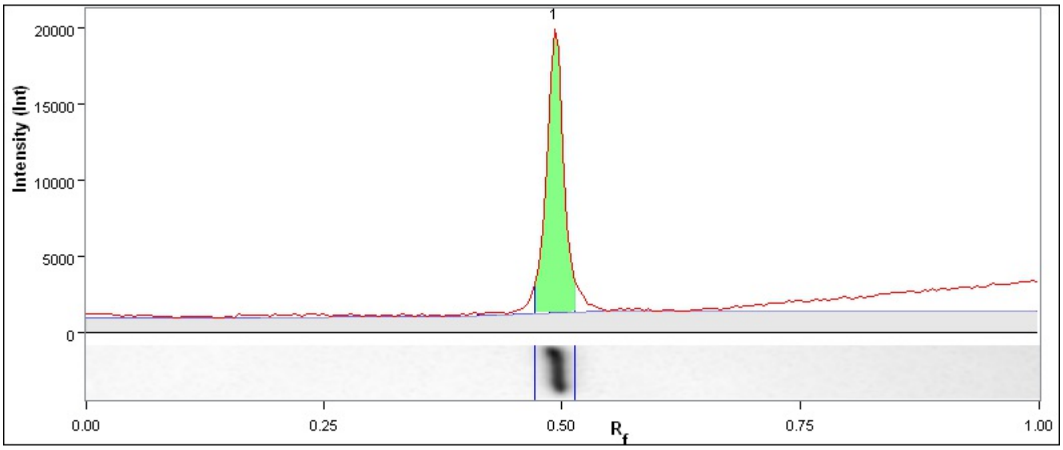

Band No.	Band Label	Mol. Wt. (KDa)	Relative Front	Volume (Int)	Abs. Quant.	Rel. Quant.	Band %	Lane %
1		41,2	0,496	2.491.429	N/A	N/A	100,0	45,7

Lane Background	Lane background subtracted with disk size: 10
Lane Width	4.71 mm
Regression Equation	A single equation is not available for this method

Lane 7

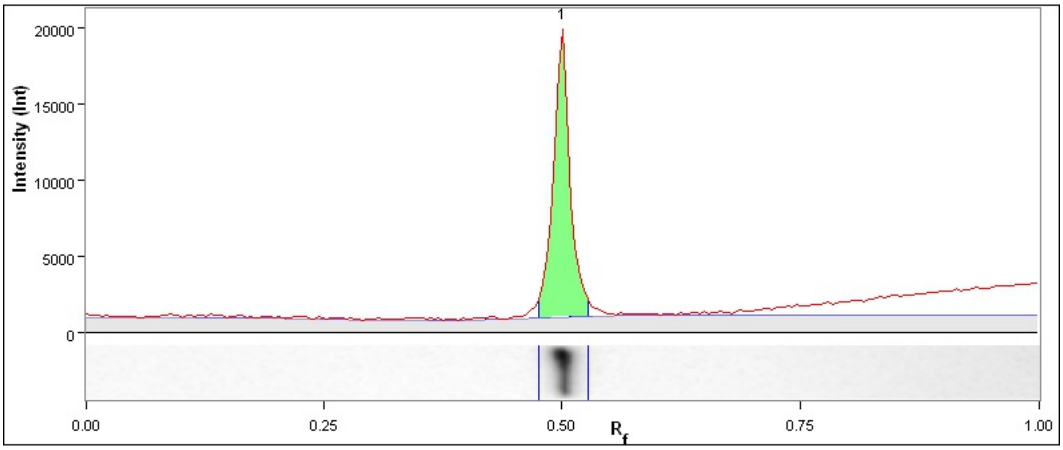

Band No.	Band Label	Mol. Wt. (KDa)	Relative Front	Volume (Int)	Abs. Quant.	Rel. Quant.	Band %	Lane %
1		41,1	0,504	2.370.449	N/A	N/A	100,0	44,6

Lane Background	Lane background subtracted with disk size: 10
Lane Width	4.71 mm
Regression Equation	A single equation is not available for this method

Lane 8

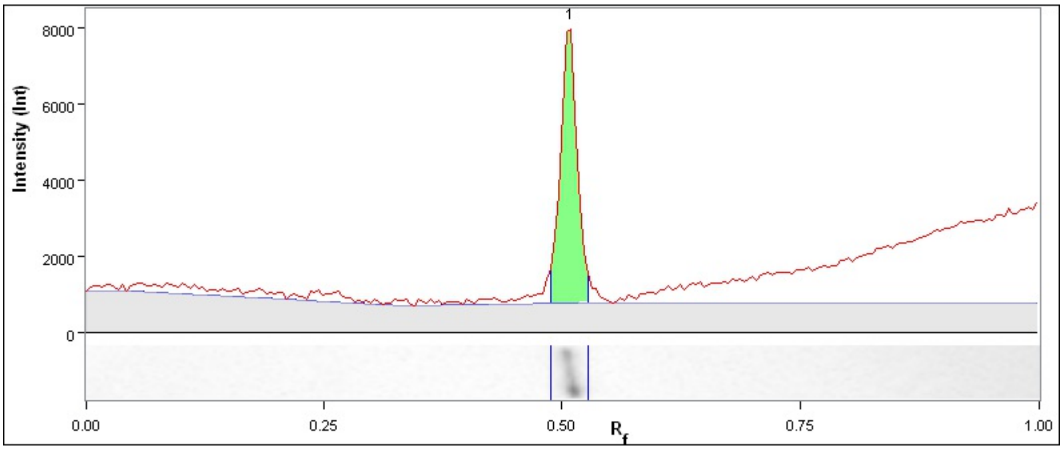

Band No.	Band Label	Mol. Wt. (KDa)	Relative Front	Volume (Int)	Abs. Quant.	Rel. Quant.	Band %	Lane %
1		41,0	0,513	824.987	N/A	N/A	100,0	19,4

Lane Background	Lane background subtracted with disk size: 10
Lane Width	4.71 mm
Regression Equation	A single equation is not available for this method

Lane 9

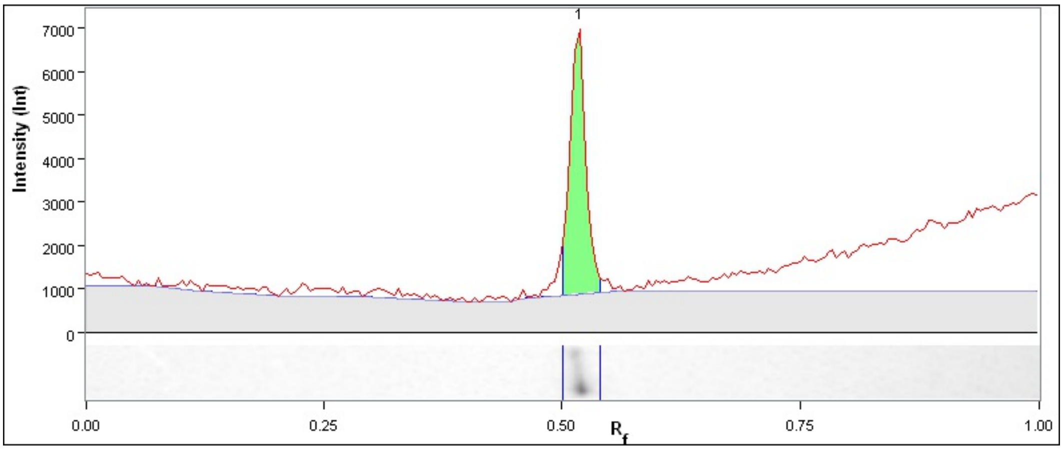

Band No.	Band Label	Mol. Wt. (KDa)	Relative Front	Volume (Int)	Abs. Quant.	Rel. Quant.	Band %	Lane %
1		40,9	0,521	653.085	N/A	N/A	100,0	18,7

Lane Background	Lane background subtracted with disk size: 10
Lane Width	4.71 mm
Regression Equation	A single equation is not available for this method

Lane 10

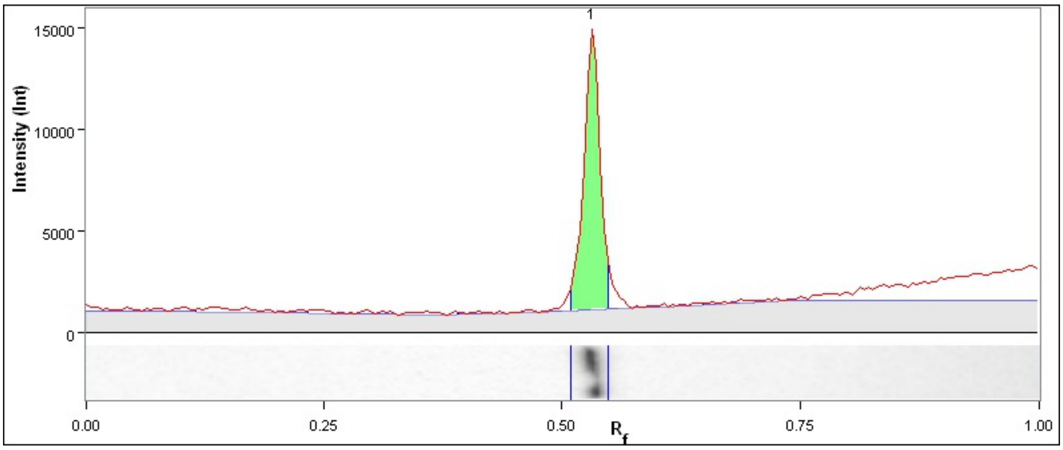

Band No.	Band Label	Mol. Wt. (KDa)	Relative Front	Volume (Int)	Abs. Quant.	Rel. Quant.	Band %	Lane %
1		40,0	0,534	1.666.511	N/A	N/A	100,0	46,8

Lane Background	Lane background subtracted with disk size: 10
Lane Width	4.71 mm
Regression Equation	A single equation is not available for this method

Lane 11

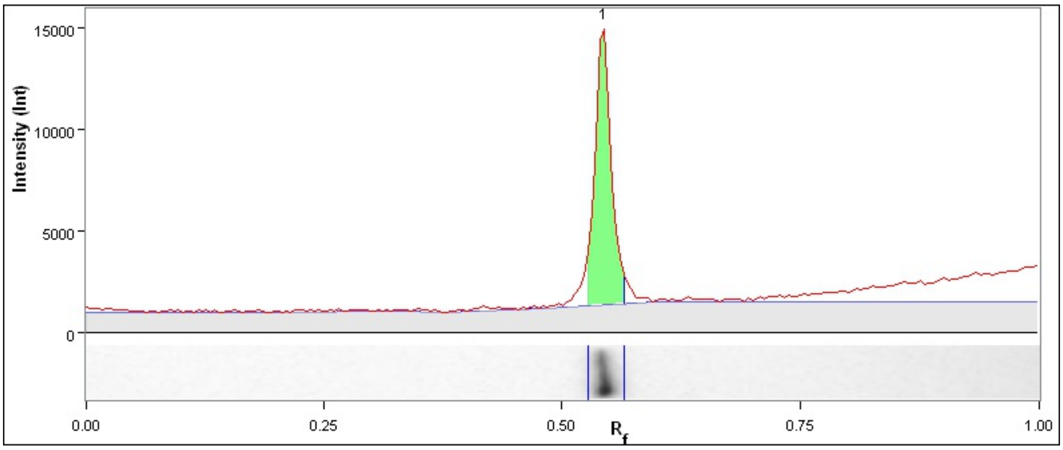

Band No.	Band Label	Mol. Wt. (KDa)	Relative Front	Volume (Int)	Abs. Quant.	Rel. Quant.	Band %	Lane %
1		39,2	0,546	1.737.213	N/A	N/A	100,0	43,5

Lane Background	Lane background subtracted with disk size: 10
Lane Width	4.71 mm
Regression Equation	A single equation is not available for this method

Lane 12

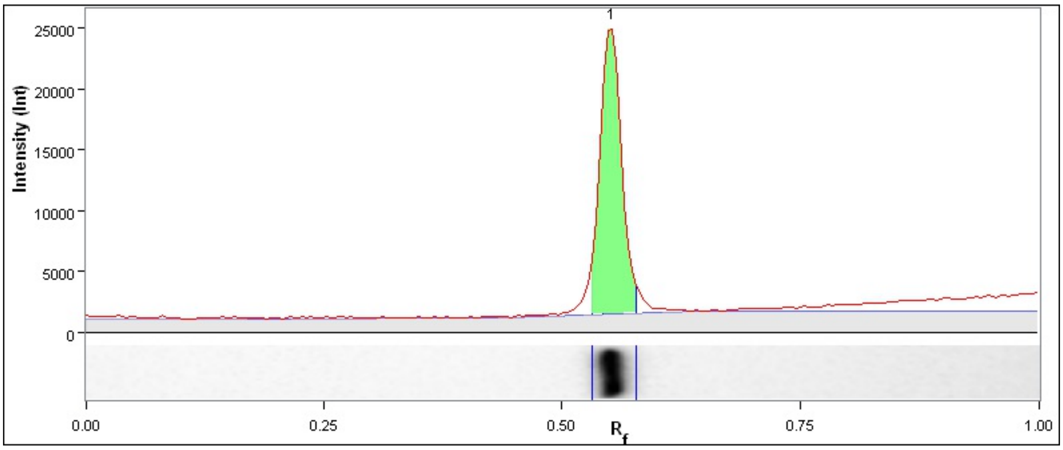

Band No.	Band Label	Mol. Wt. (KDa)	Relative Front	Volume (Int)	Abs. Quant.	Rel. Quant.	Band %	Lane %
1		39,1	0,555	4.032.245	N/A	N/A	100,0	62,5

Lane Background	Lane background subtracted with disk size: 10
Lane Width	4.71 mm
Regression Equation	A single equation is not available for this method

Lane 13

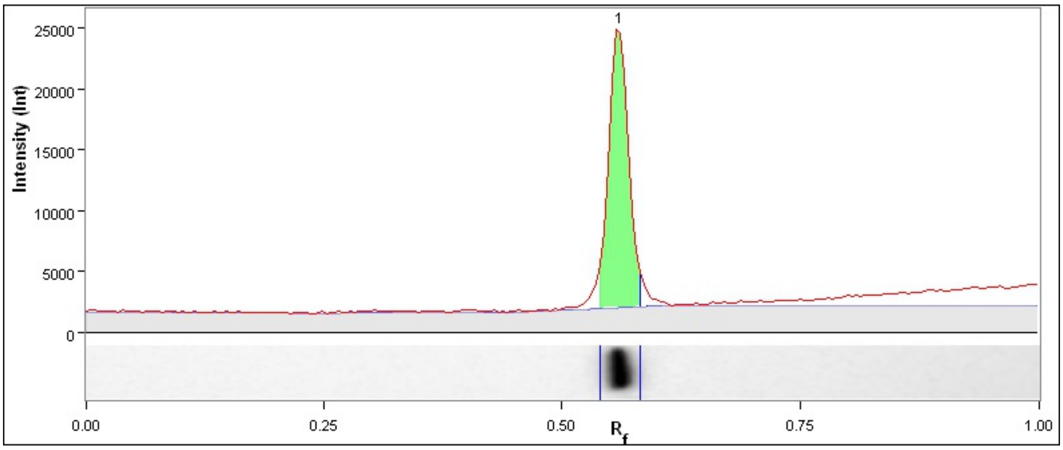

Band No.	Band Label	Mol. Wt. (KDa)	Relative Front	Volume (Int)	Abs. Quant.	Rel. Quant.	Band %	Lane %
1		39,0	0,563	3.210.961	N/A	N/A	100,0	58,2

Lane Background	Lane background subtracted with disk size: 10
Lane Width	4.71 mm
Regression Equation	A single equation is not available for this method

Lane 14

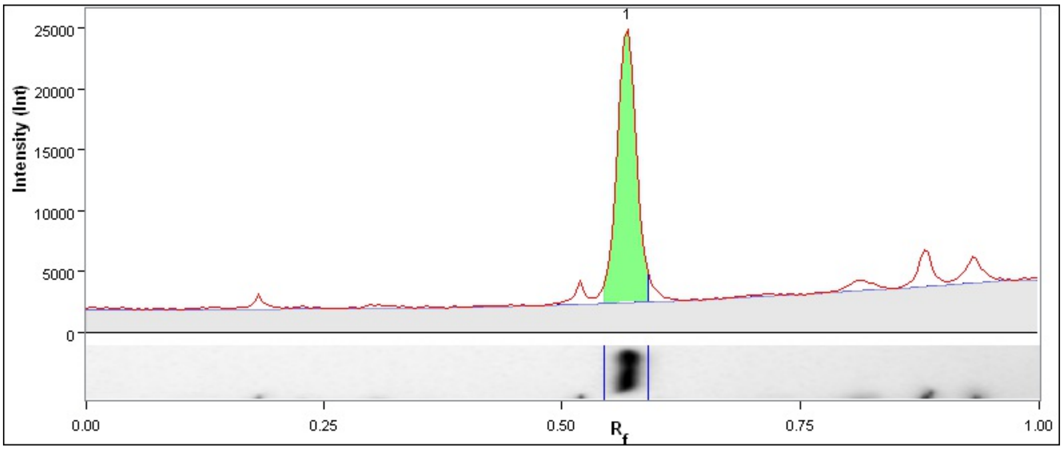

Band No.	Band Label	Mol. Wt. (KDa)	Relative Front	Volume (Int)	Abs. Quant.	Rel. Quant.	Band %	Lane %
1		38,6	0,571	3.869.656	N/A	N/A	100,0	67,3

Lane Background	Lane background subtracted with disk size: 10
Lane Width	5.73 mm
Regression Equation	A single equation is not available for this method

Lane 15 - Bio-Rad Precision Plus

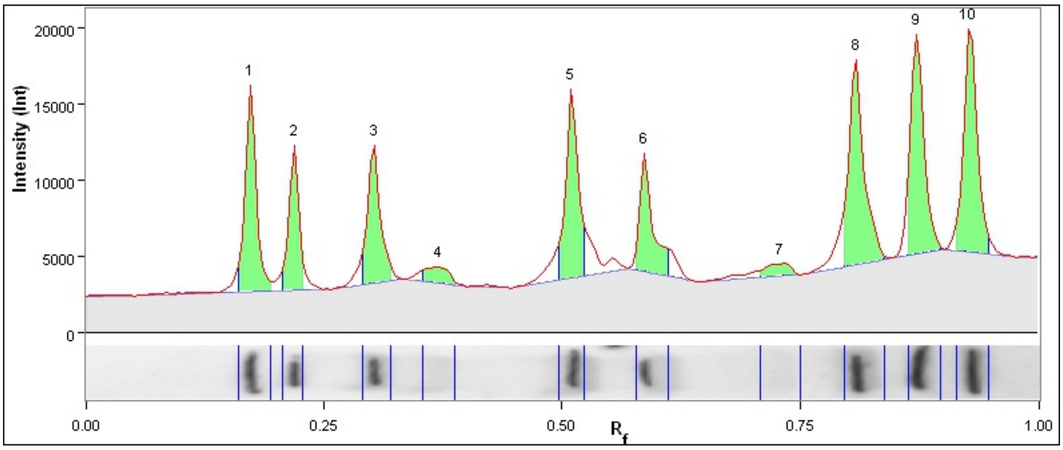

Band No.	Band Label	Mol. Wt. (KDa)	Relative Front	Volume (Int)	Abs. Quant.	Rel. Quant.	Band %	Lane %
1		250,0	0,176	1.333.472	N/A	N/A	12,1	10,3
2		150,0	0,223	862.960	N/A	N/A	7,8	6,7
3		100,0	0,307	1.012.984	N/A	N/A	9,2	7,8
4		75,0	0,374	215.628	N/A	N/A	2,0	1,7
5		50,0	0,513	1.361.444	N/A	N/A	12,3	10,5
6		37,0	0,588	943.040	N/A	N/A	8,5	7,3
7		25,0	0,731	176.148	N/A	N/A	1,6	1,4
8		20,0	0,811	1.745.072	N/A	N/A	15,8	13,5
9		15,0	0,874	1.580.376	N/A	N/A	14,3	12,2
10		10,0	0,929	1.801.464	N/A	N/A	16,3	13,9

Lane Background	Lane background subtracted with disk size: 10
Lane Width	5.73 mm
Regression Equation	A single equation is not available for this method
