# Supplementary material for: Short-chain fructo-oligosaccharides supplementation to suckling piglets: Assessment of pre- and post-weaning performance and gut health
Source: PLoS One. 2020 Jun 5;15(6):e0233910. doi: 10.1371/journal.pone.0233910 (PMC7274435; doi:10.1371/journal.pone.0233910)
Supplement: S4 Data — (PDF) [file pone.0233910.s006.pdf]

Image Report: B-actin_5_LADDER+B-actin_2

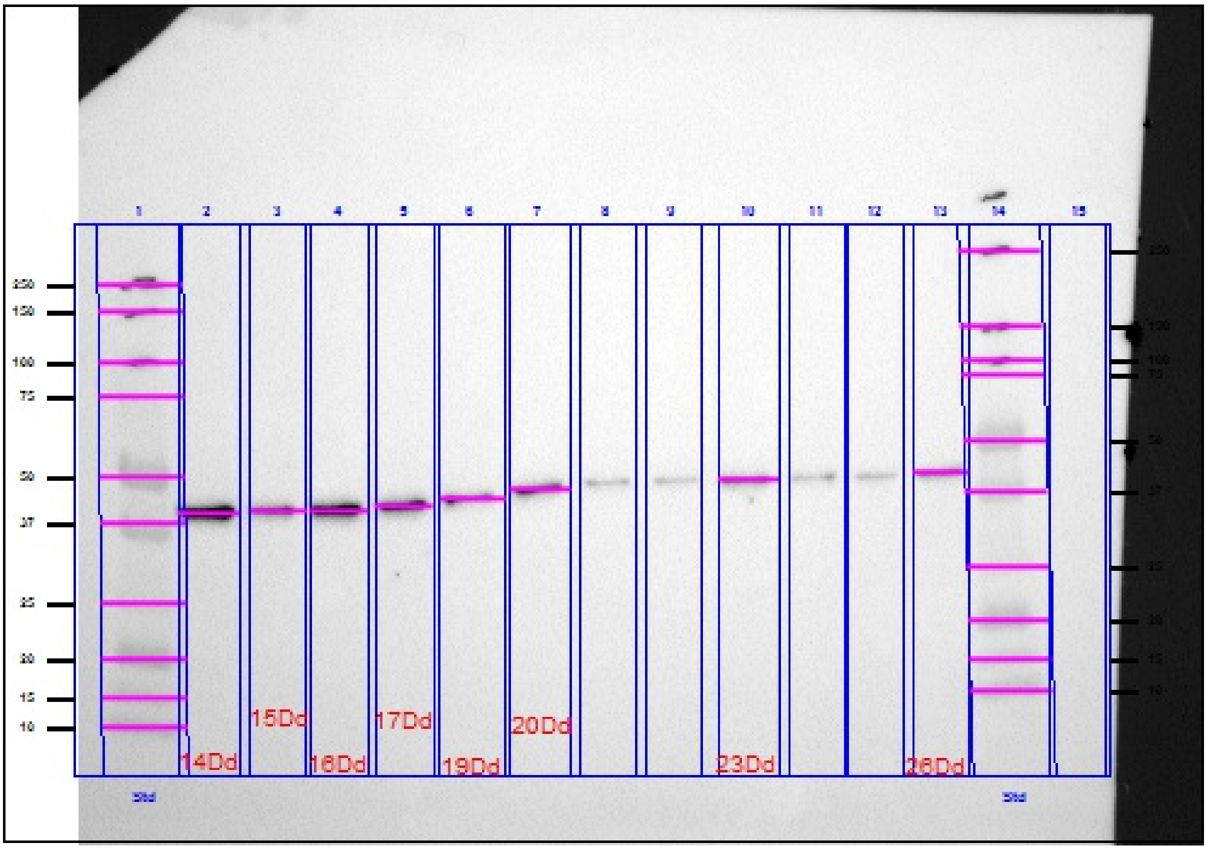

Acquisition Information

Imager	Merged Image

Image Information

Acquisition Date	17/05/2017 13:29:42
User Name	Bio-Rad
Image Area (mm)	X: 95.0 Y: 71.0
Pixel Size (um)	X: 204.7 Y: 205.1
Data Range (Int)	0 - 64750

Notes

Merged images:
Image 1: B-actin_5_LADDER
Image 2: B-actin_2

Analysis Settings

Detection	Lane detection:<br>Manually created lanes<br><br>Band detection:<br><br>Manually adjusted bands

	Lane Background Subtraction:<br>Lane background subtracted with disk size: 10<br><br>Lane width: Variable
Mol. Weight Analysis	Standard: Bio-Rad Precision Plus<br>Standard lanes: first 14<br>Regression method: Point to Point (semi-log)

Lane And Band Analysis

Lane 1 - Bio-Rad Precision Plus

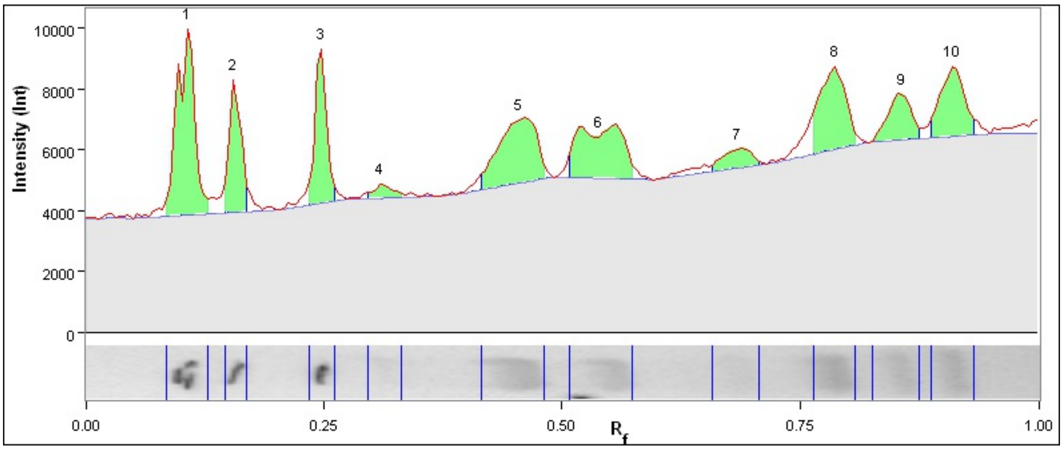

Band No.	Band Label	Mol. Wt. (KDa)	Relative Front	Volume (Int)	Abs. Quant.	Rel. Quant.	Band %	Lane %
1		250,0	0,110	1.232.840	N/A	N/A	18,9	16,4
2		150,0	0,159	592.130	N/A	N/A	9,1	7,9
3		100,0	0,251	656.390	N/A	N/A	10,1	8,7
4		75,0	0,313	108.220	N/A	N/A	1,7	1,4
5		50,0	0,458	945.350	N/A	N/A	14,5	12,6
6		37,0	0,542	894.390	N/A	N/A	13,7	11,9
7		25,0	0,687	226.730	N/A	N/A	3,5	3,0
8		20,0	0,789	784.350	N/A	N/A	12,0	10,4
9		15,0	0,859	433.860	N/A	N/A	6,6	5,8
10		10,0	0,912	655.480	N/A	N/A	10,0	8,7

Lane Background	Lane background subtracted with disk size: 10
Lane Width	7.17 mm
Regression Equation	A single equation is not available for this method

Lane 2

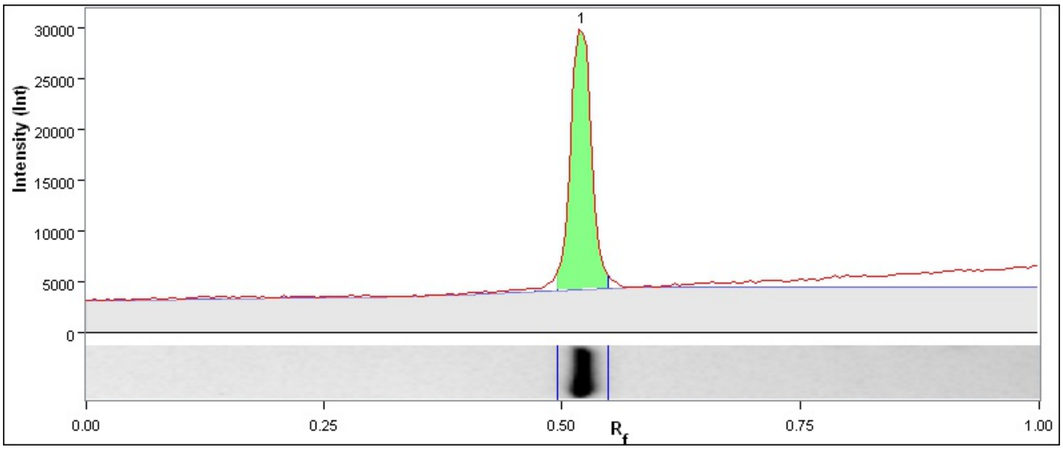

Band No.	Band Label	Mol. Wt. (KDa)	Relative Front	Volume (Int)	Abs. Quant.	Rel. Quant.	Band %	Lane %
1		38,8	0,524	4.330.225	N/A	N/A	100,0	55,8

Lane Background	Lane background subtracted with disk size: 10
Lane Width	5.12 mm
Regression Equation	A single equation is not available for this method

Lane 3

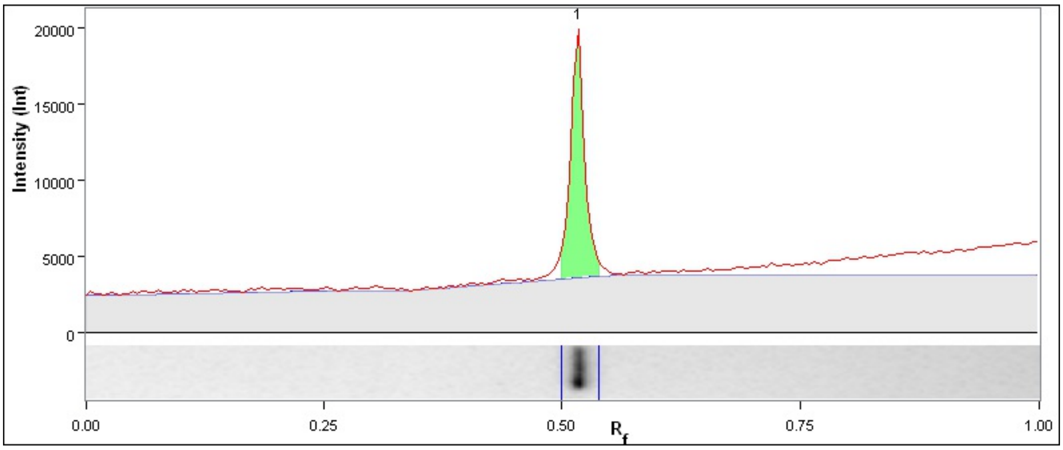

Band No.	Band Label	Mol. Wt. (KDa)	Relative Front	Volume (Int)	Abs. Quant.	Rel. Quant.	Band %	Lane %
1		38,7	0,520	1.641.947	N/A	N/A	100,0	34,5

Lane Background	Lane background subtracted with disk size: 10
Lane Width	4.71 mm
Regression Equation	A single equation is not available for this method

Lane 4

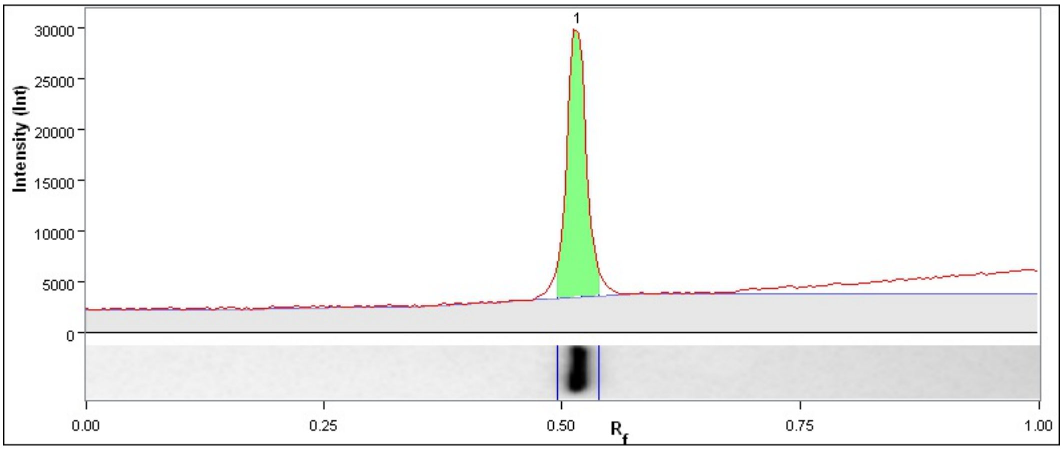

Band No.	Band Label	Mol. Wt. (KDa)	Relative Front	Volume (Int)	Abs. Quant.	Rel. Quant.	Band %	Lane %
1		38,1	0,520	3.561.216	N/A	N/A	100,0	54,0

Lane Background	Lane background subtracted with disk size: 10
Lane Width	4.91 mm
Regression Equation	A single equation is not available for this method

Lane 5

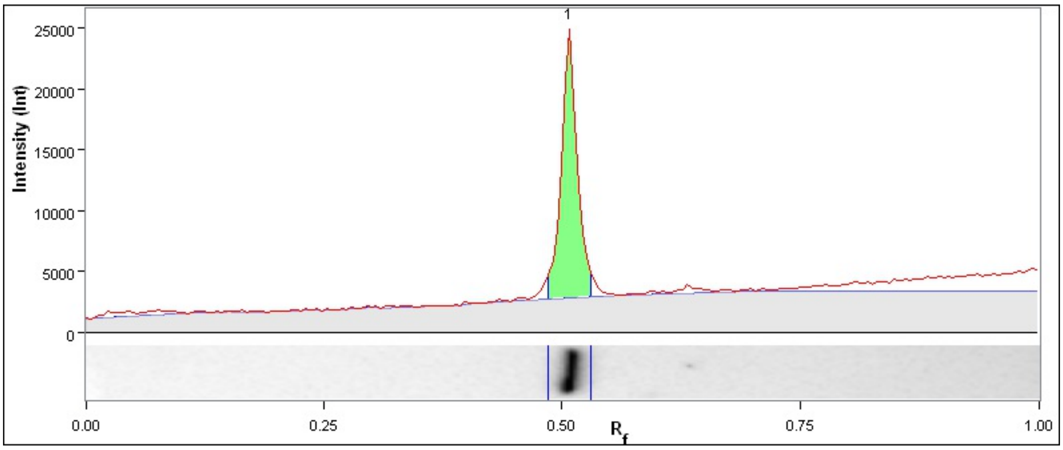

Band No.	Band Label	Mol. Wt. (KDa)	Relative Front	Volume (Int)	Abs. Quant.	Rel. Quant.	Band %	Lane %
1		38,7	0,511	2.689.416	N/A	N/A	100,0	54,2

Lane Background	Lane background subtracted with disk size: 10
Lane Width	4.91 mm
Regression Equation	A single equation is not available for this method

Lane 6

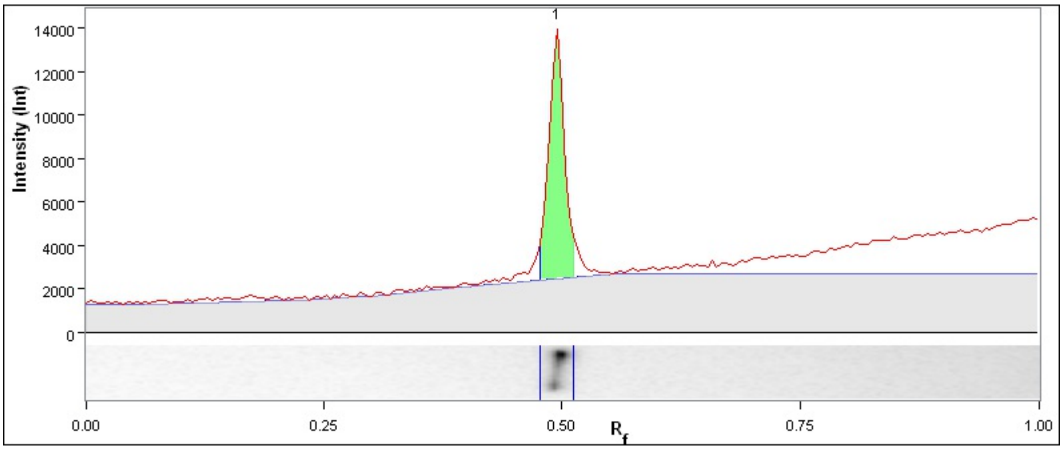

Band No.	Band Label	Mol. Wt. (KDa)	Relative Front	Volume (Int)	Abs. Quant.	Rel. Quant.	Band %	Lane %
1		39,9	0,498	1.413.504	N/A	N/A	100,0	26,7

Lane Background	Lane background subtracted with disk size: 10
Lane Width	5.53 mm
Regression Equation	A single equation is not available for this method

Lane 7

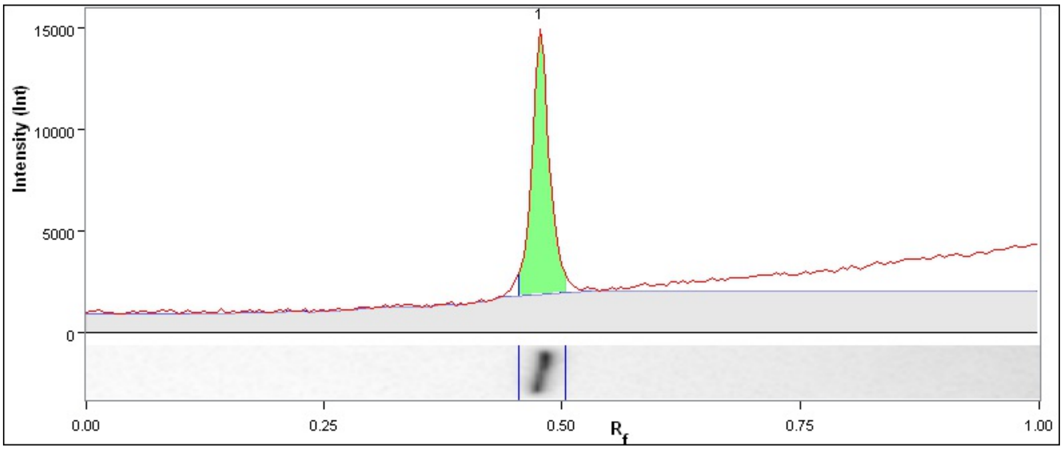

Band No.	Band Label	Mol. Wt. (KDa)	Relative Front	Volume (Int)	Abs. Quant.	Rel. Quant.	Band %	Lane %
1		41,7	0,480	1.881.900	N/A	N/A	100,0	34,8

Lane Background	Lane background subtracted with disk size: 10
Lane Width	5.12 mm
Regression Equation	A single equation is not available for this method

Lane 8

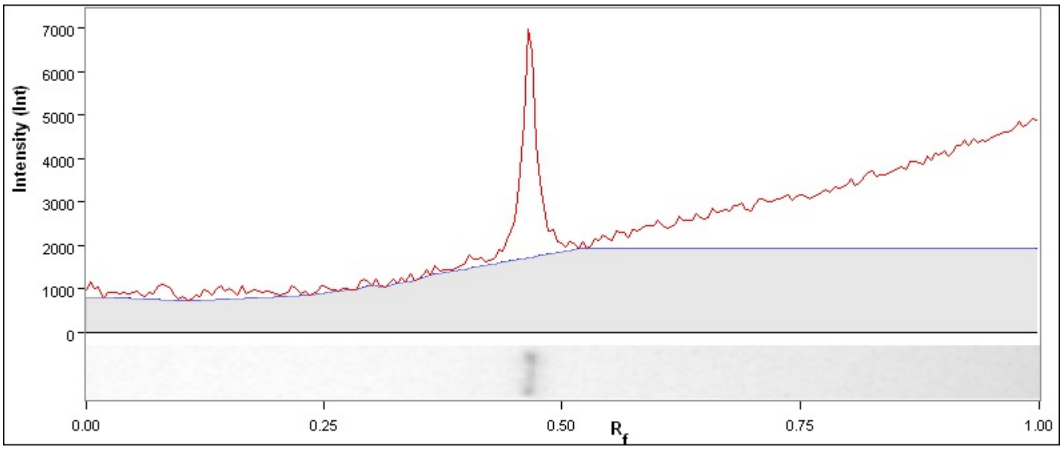

Band No.	Band Label	Mol. Wt. (KDa)	Relative Front	Volume (Int)	Abs. Quant.	Rel. Quant.	Band %	Lane %

Lane Background	Lane background subtracted with disk size: 10
Lane Width	4.71 mm
Regression Equation	A single equation is not available for this method

Lane 9

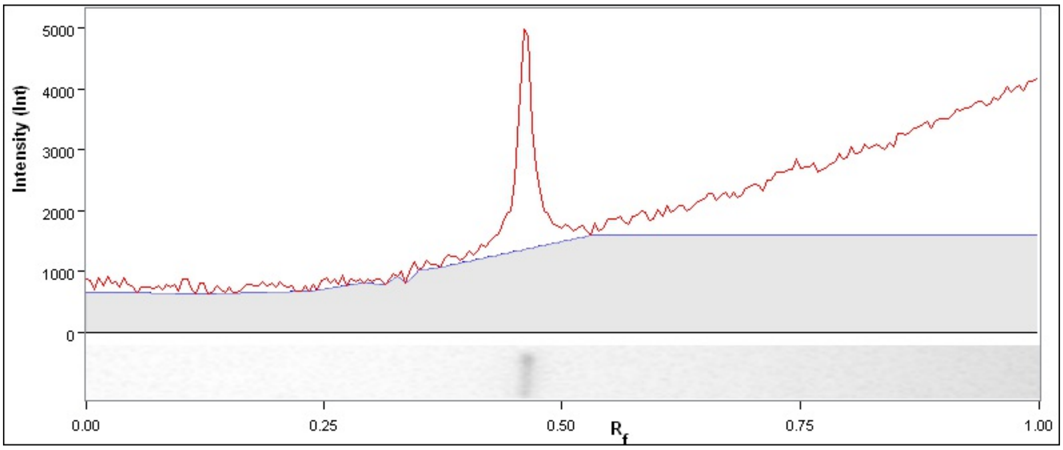

Band No.	Band Label	Mol. Wt. (KDa)	Relative Front	Volume (Int)	Abs. Quant.	Rel. Quant.	Band %	Lane %

Lane Background	Lane background subtracted with disk size: 10
Lane Width	4.71 mm
Regression Equation	A single equation is not available for this method

Lane 10

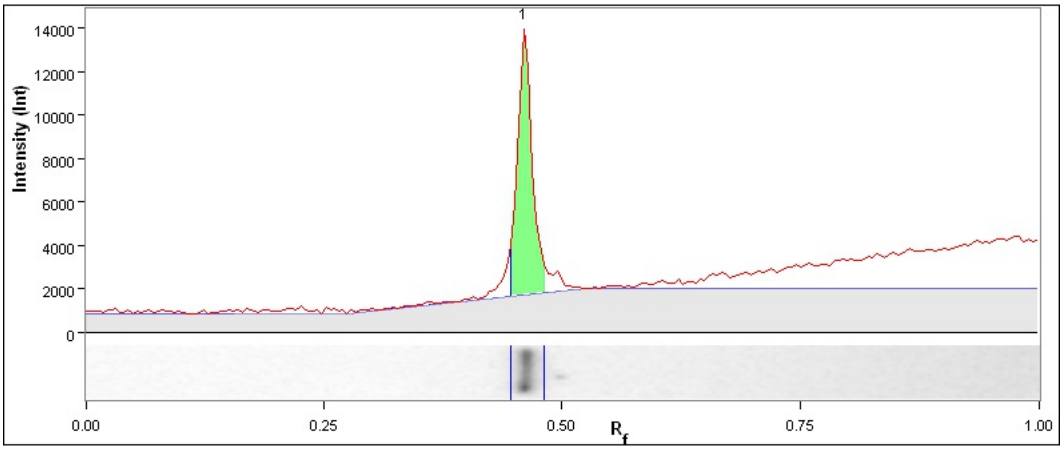

Band No.	Band Label	Mol. Wt. (KDa)	Relative Front	Volume (Int)	Abs. Quant.	Rel. Quant.	Band %	Lane %
1		42,1	0,463	1.307.625	N/A	N/A	100,0	25,5

Lane Background	Lane background subtracted with disk size: 10
Lane Width	5.12 mm
Regression Equation	A single equation is not available for this method

Lane 11

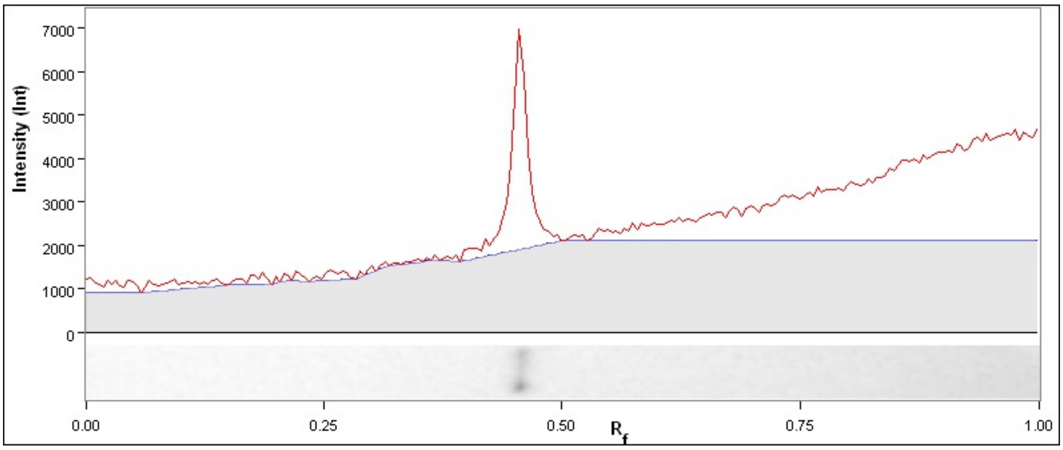

Band No.	Band Label	Mol. Wt. (KDa)	Relative Front	Volume (Int)	Abs. Quant.	Rel. Quant.	Band %	Lane %

Lane Background	Lane background subtracted with disk size: 10
Lane Width	4.71 mm
Regression Equation	A single equation is not available for this method

Lane 12

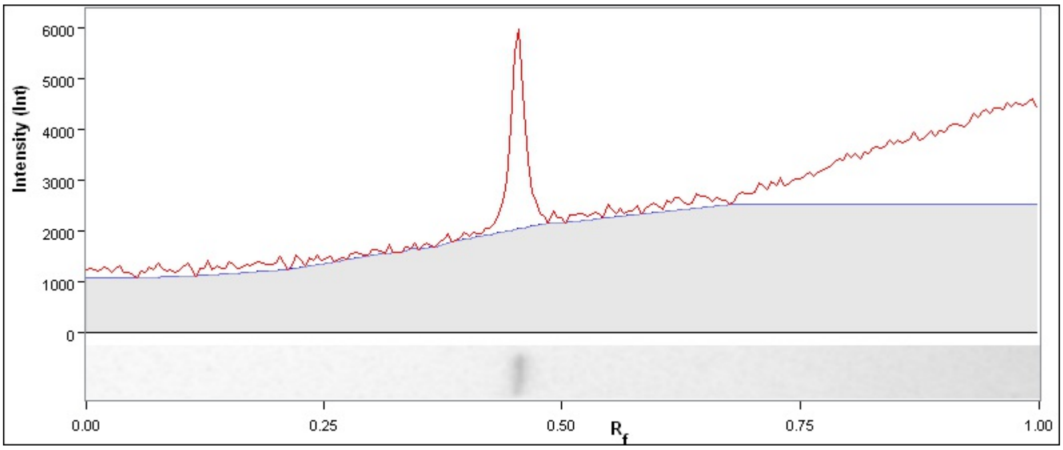

Band No.	Band Label	Mol. Wt. (KDa)	Relative Front	Volume (Int)	Abs. Quant.	Rel. Quant.	Band %	Lane %

Lane Background	Lane background subtracted with disk size: 10
Lane Width	4.71 mm
Regression Equation	A single equation is not available for this method

Lane 13

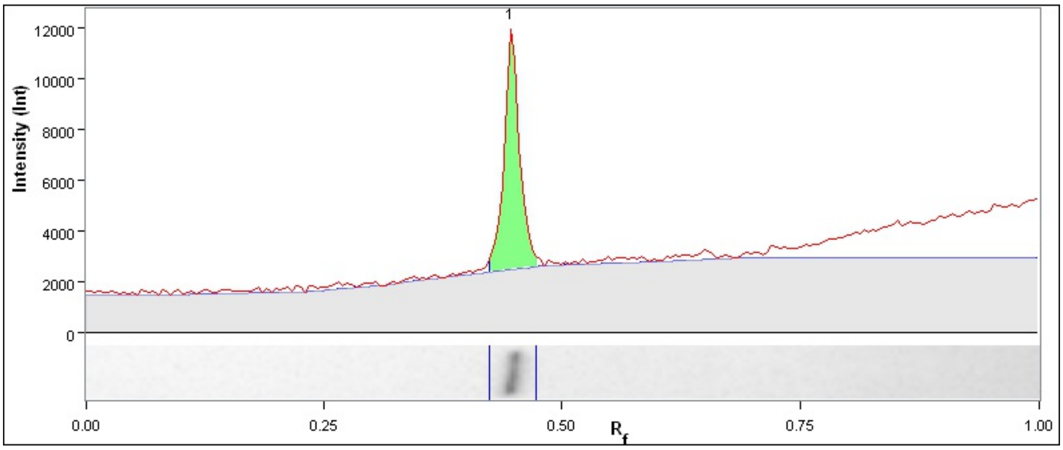

Band No.	Band Label	Mol. Wt. (KDa)	Relative Front	Volume (Int)	Abs. Quant.	Rel. Quant.	Band %	Lane %
1		42,1	0,449	1.006.733	N/A	N/A	100,0	28,5

Lane Background	Lane background subtracted with disk size: 10
Lane Width	4.71 mm
Regression Equation	A single equation is not available for this method

Lane 14 - Bio-Rad Precision Plus

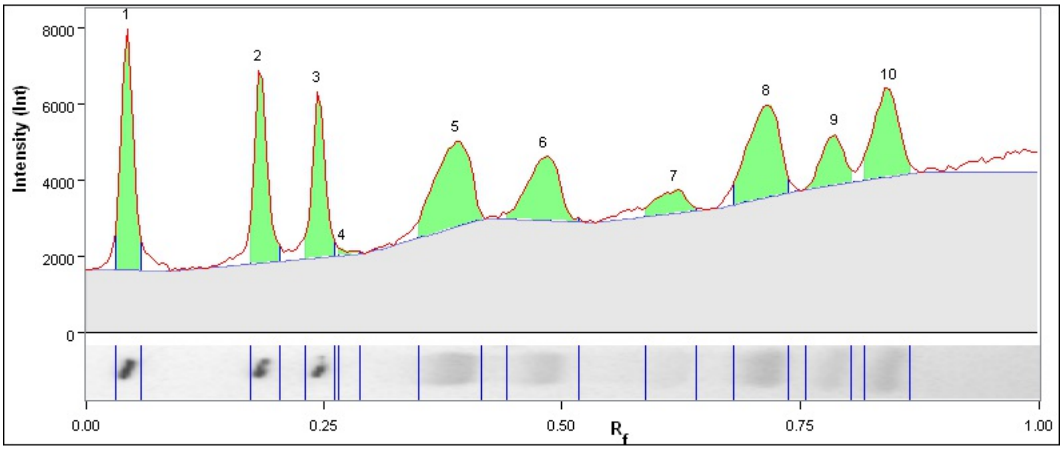

Band No.	Band Label	Mol. Wt. (KDa)	Relative Front	Volume (Int)	Abs. Quant.	Rel. Quant.	Band %	Lane %
1		250,0	0,048	904.400	N/A	N/A	14,7	12,7
2		150,0	0,185	758.166	N/A	N/A	12,3	10,7
3		100,0	0,247	648.652	N/A	N/A	10,6	9,1
4		75,0	0,273	21.624	N/A	N/A	0,4	0,3
5		50,0	0,392	947.444	N/A	N/A	15,4	13,3
6		37,0	0,485	641.172	N/A	N/A	10,4	9,0
7		25,0	0,621	229.194	N/A	N/A	3,7	3,2
8		20,0	0,718	925.106	N/A	N/A	15,1	13,0
9		15,0	0,789	363.494	N/A	N/A	5,9	5,1
10		10,0	0,846	705.704	N/A	N/A	11,5	9,9

Lane Background	Lane background subtracted with disk size: 10
Lane Width	6.96 mm
Regression Equation	A single equation is not available for this method

Lane 15

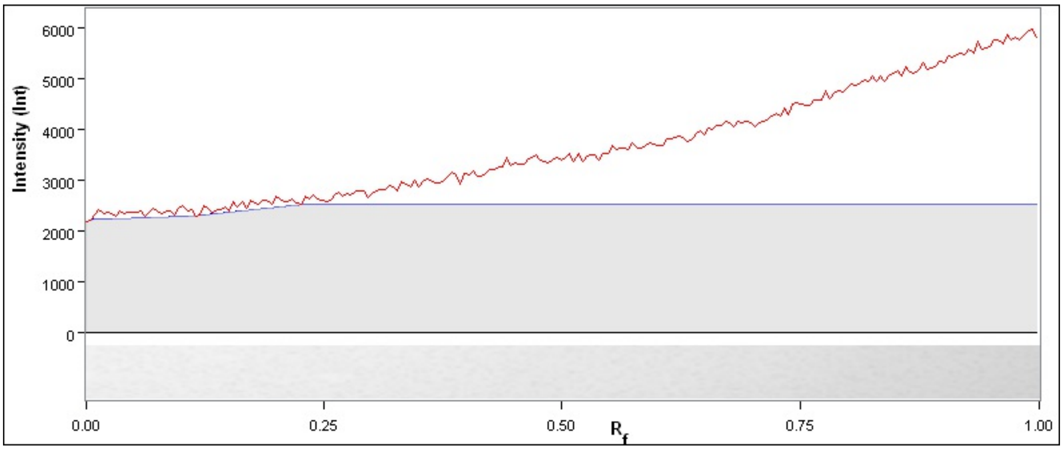

Band No.	Band Label	Mol. Wt. (KDa)	Relative Front	Volume (Int)	Abs. Quant.	Rel. Quant.	Band %	Lane %

Lane Background	Lane background subtracted with disk size: 10
Lane Width	4.71 mm
Regression Equation	A single equation is not available for this method
