# Supplementary material for: Short-chain fructo-oligosaccharides supplementation to suckling piglets: Assessment of pre- and post-weaning performance and gut health
Source: PLoS One. 2020 Jun 5;15(6):e0233910. doi: 10.1371/journal.pone.0233910 (PMC7274435; doi:10.1371/journal.pone.0233910)
Supplement: S5 Data — (PDF) [file pone.0233910.s007.pdf]

Image Report: B-actin7_LADDER+B-actin7

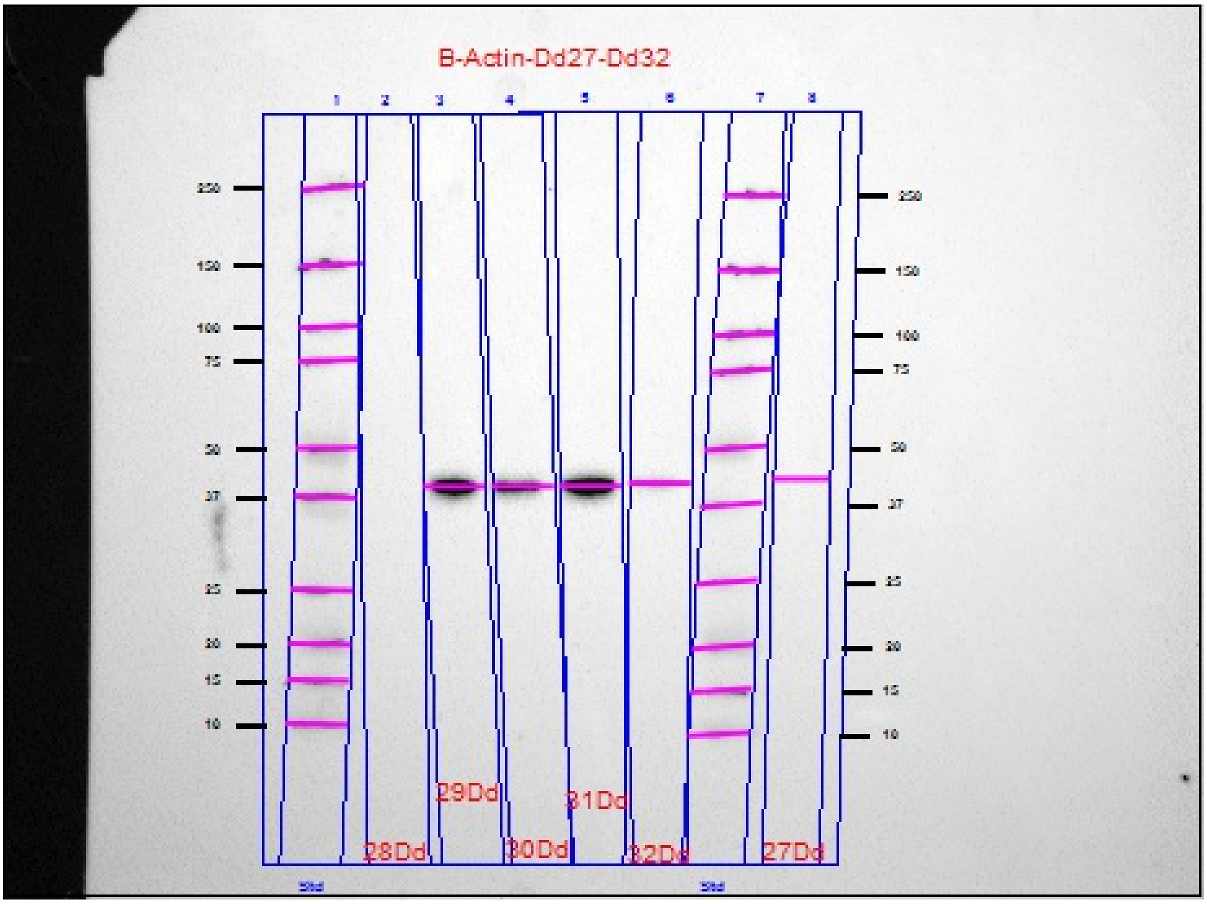

Acquisition Information

Imager	Merged Image

Image Information

Acquisition Date	19/05/2017 10:35:29
User Name	Bio-Rad
Image Area (mm)	X: 95.0 Y: 71.0
Pixel Size (um)	X: 204.7 Y: 205.1
Data Range (Int)	0 - 46192

Notes

Merged images:
Image 1: B-actin7_LADDER
Image 2: B-actin7

Analysis Settings

Detection	Lane detection:<br>Automatically detected lanes with manual adjustments<br><br>Band detection:

	Manually adjusted bands<br><br>Lane Background Subtraction:<br>Lane background subtracted with disk size: 10<br><br>Lane width: Variable
Mol. Weight Analysis	Standard: Bio-Rad Precision Plus<br>Standard lanes: first 7<br>Regression method: Point to Point (semi-log)

Lane And Band Analysis

Lane 1 - Bio-Rad Precision Plus

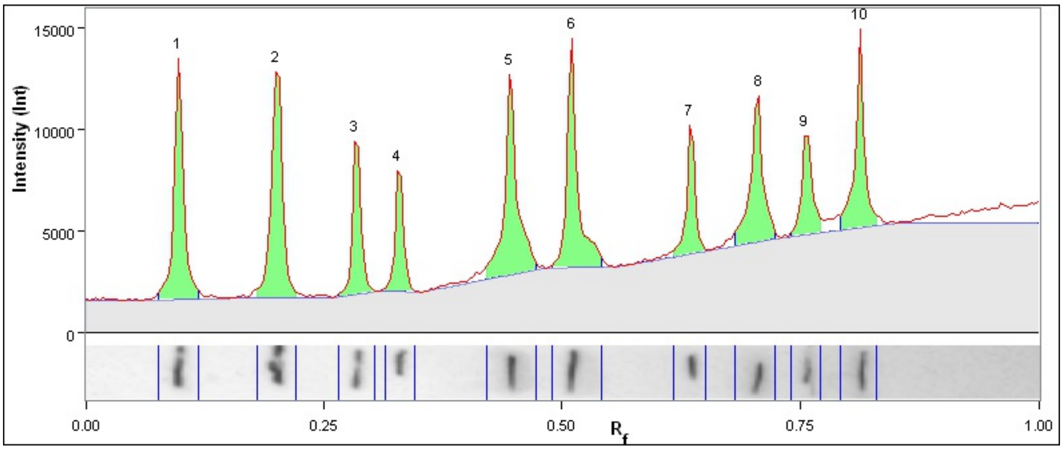

Band No.	Band Label	Mol. Wt. (KDa)	Relative Front	Volume (Int)	Abs. Quant.	Rel. Quant.	Band %	Lane %
1		250,0	0,100	1.121.184	N/A	N/A	12,7	11,3
2		150,0	0,203	1.301.568	N/A	N/A	14,8	13,1
3		100,0	0,285	671.424	N/A	N/A	7,6	6,8
4		75,0	0,330	502.800	N/A	N/A	5,7	5,1
5		50,0	0,447	1.225.608	N/A	N/A	13,9	12,4
6		37,0	0,512	1.162.656	N/A	N/A	13,2	11,7
7		25,0	0,636	545.544	N/A	N/A	6,2	5,5
8		20,0	0,708	891.312	N/A	N/A	10,1	9,0
9		15,0	0,756	549.648	N/A	N/A	6,2	5,6
10		10,0	0,814	841.680	N/A	N/A	9,5	8,5

Lane Background	Lane background subtracted with disk size: 10
Lane Width	4.91 mm
Regression Equation	A single equation is not available for this method

Lane 2

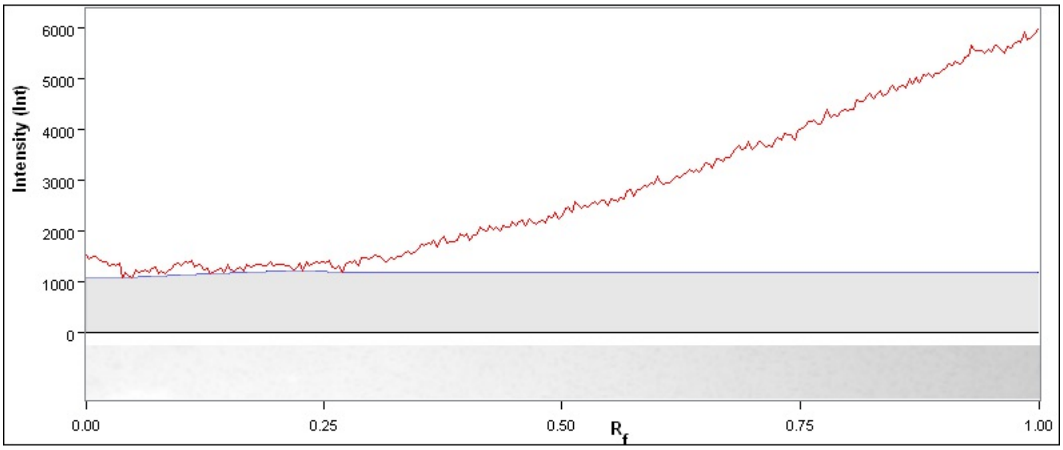

Band No.	Band Label	Mol. Wt. (KDa)	Relative Front	Volume (Int)	Abs. Quant.	Rel. Quant.	Band %	Lane %

Lane Background	Lane background subtracted with disk size: 10
Lane Width	4.91 mm
Regression Equation	A single equation is not available for this method

Lane 3

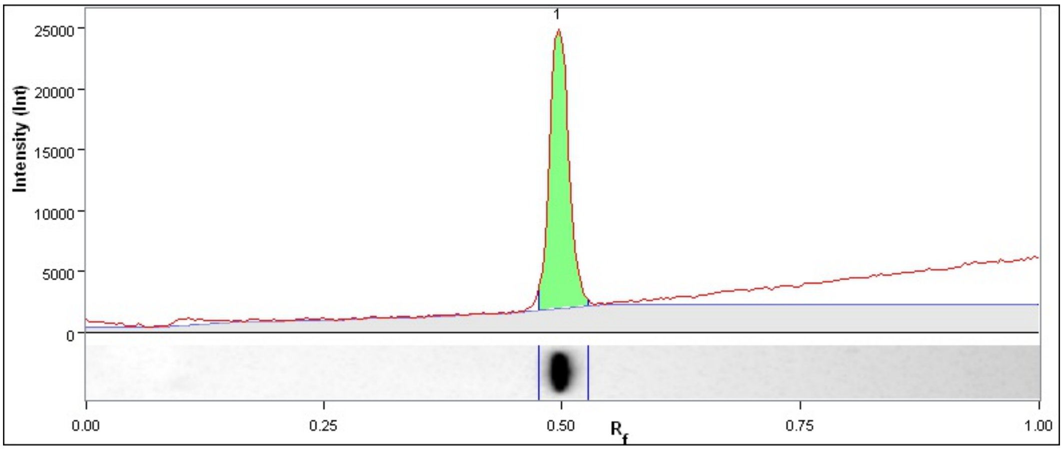

Band No.	Band Label	Mol. Wt. (KDa)	Relative Front	Volume (Int)	Abs. Quant.	Rel. Quant.	Band %	Lane %
1		39,9	0,498	4.011.024	N/A	N/A	100,0	36,2

Lane Background	Lane background subtracted with disk size: 10
Lane Width	4.91 mm
Regression Equation	A single equation is not available for this method

Lane 4

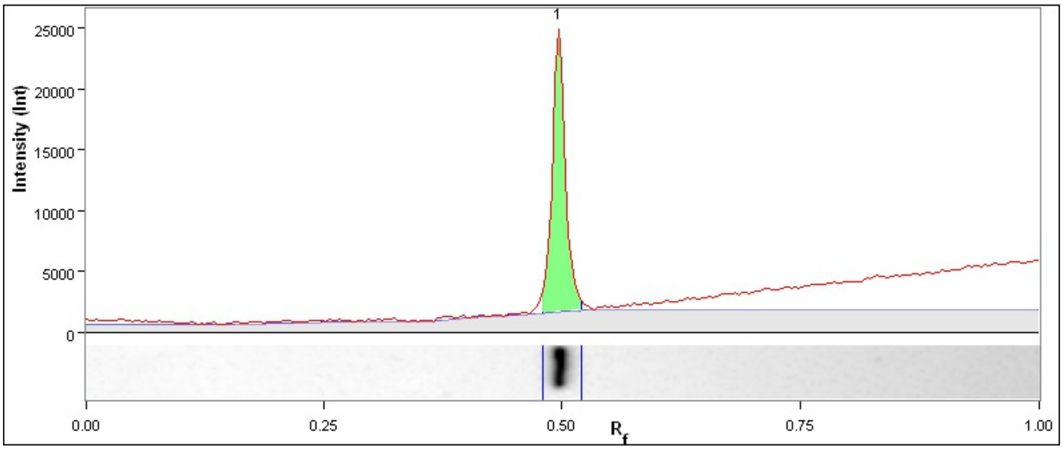

Band No.	Band Label	Mol. Wt. (KDa)	Relative Front	Volume (Int)	Abs. Quant.	Rel. Quant.	Band %	Lane %
1		40,1	0,498	2.679.264	N/A	N/A	100,0	26,2

Lane Background	Lane background subtracted with disk size: 10
Lane Width	4.91 mm
Regression Equation	A single equation is not available for this method

Lane 5

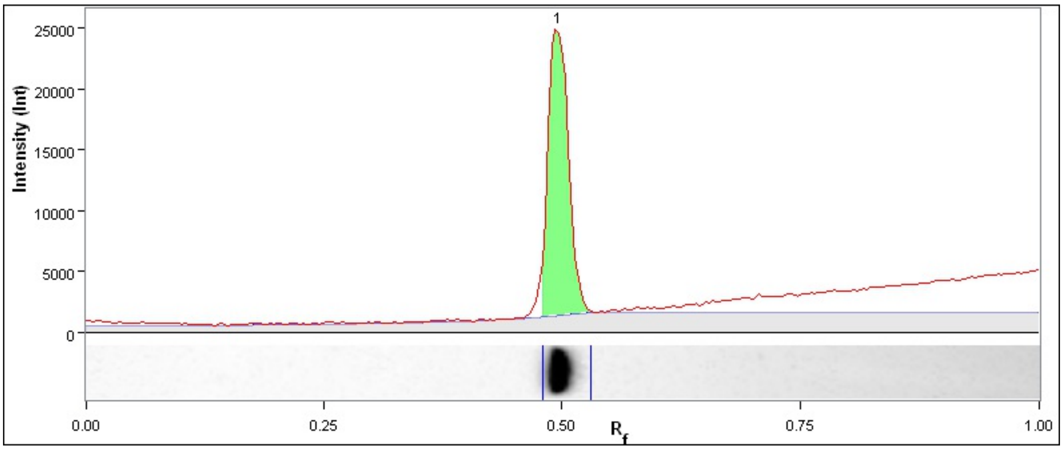

Band No.	Band Label	Mol. Wt. (KDa)	Relative Front	Volume (Int)	Abs. Quant.	Rel. Quant.	Band %	Lane %
1		40,4	0,498	4.685.544	N/A	N/A	100,0	40,2

Lane Background	Lane background subtracted with disk size: 10
Lane Width	4.91 mm
Regression Equation	A single equation is not available for this method

Lane 6

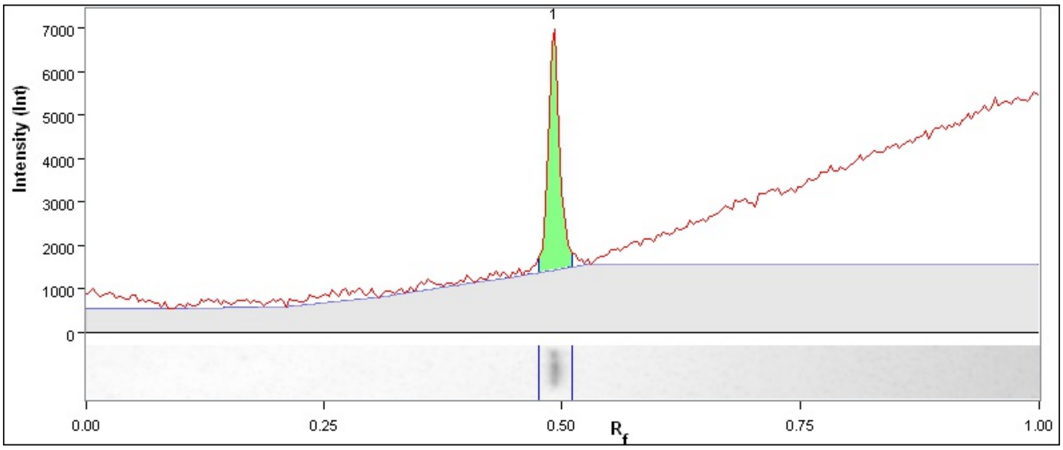

Band No.	Band Label	Mol. Wt. (KDa)	Relative Front	Volume (Int)	Abs. Quant.	Rel. Quant.	Band %	Lane %
1		41,3	0,495	587.664	N/A	N/A	100,0	7,1

Lane Background	Lane background subtracted with disk size: 10
Lane Width	4.91 mm
Regression Equation	A single equation is not available for this method

Lane 7 - Bio-Rad Precision Plus

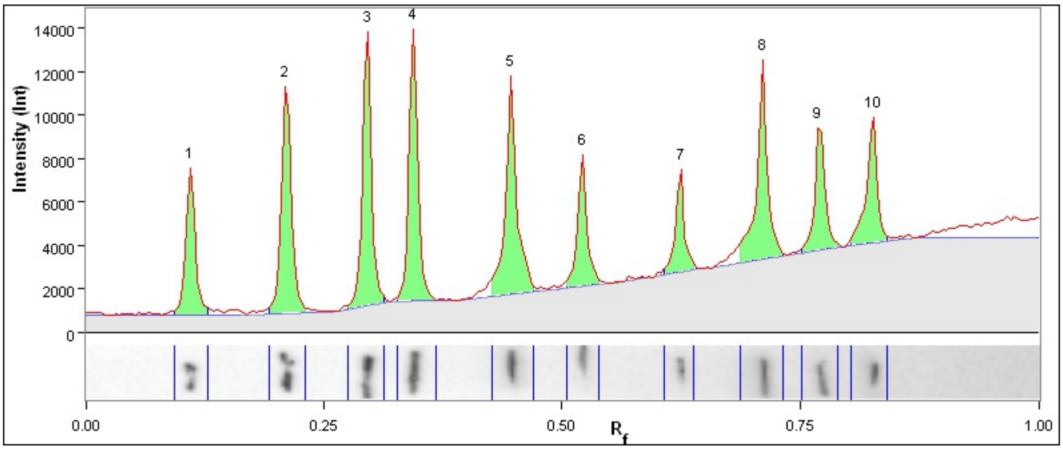

Band No.	Band Label	Mol. Wt. (KDa)	Relative Front	Volume (Int)	Abs. Quant.	Rel. Quant.	Band %	Lane %
1		250,0	0,113	620.712	N/A	N/A	7,8	7,0
2		150,0	0,212	1.018.200	N/A	N/A	12,8	11,4
3		100,0	0,298	1.114.824	N/A	N/A	14,0	12,5
4		75,0	0,346	1.074.936	N/A	N/A	13,5	12,0
5		50,0	0,449	1.073.976	N/A	N/A	13,5	12,0
6		37,0	0,524	541.080	N/A	N/A	6,8	6,1
7		25,0	0,627	400.416	N/A	N/A	5,0	4,5
8		20,0	0,712	927.264	N/A	N/A	11,7	10,4
9		15,0	0,771	575.616	N/A	N/A	7,2	6,5
10		10,0	0,829	592.728	N/A	N/A	7,5	6,6

Lane Background	Lane background subtracted with disk size: 10
Lane Width	4.91 mm
Regression Equation	A single equation is not available for this method

Lane 8

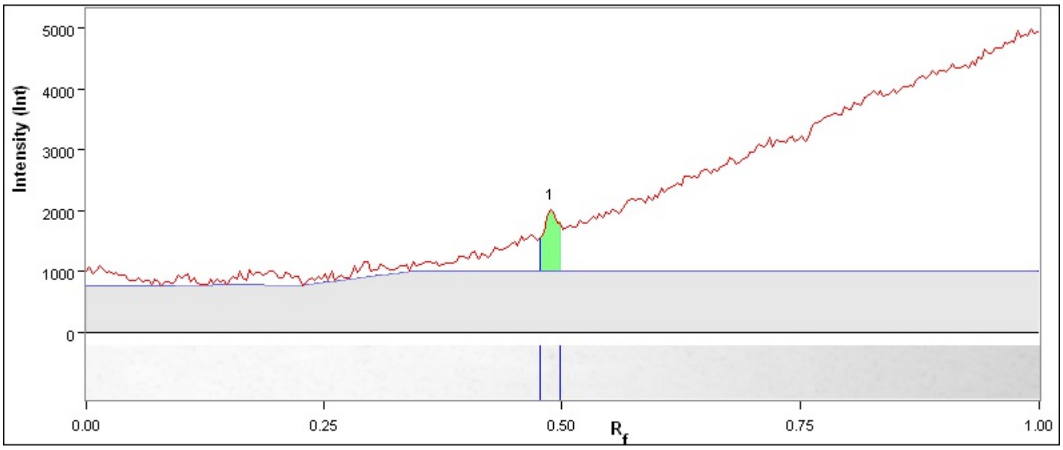

Band No.	Band Label	Mol. Wt. (KDa)	Relative Front	Volume (Int)	Abs. Quant.	Rel. Quant.	Band %	Lane %
1		42,4	0,490	168.960	N/A	N/A	100,0	1,8

Lane Background	Lane background subtracted with disk size: 10
Lane Width	4.50 mm
Regression Equation	A single equation is not available for this method
