# Supplementary material for: Short-chain fructo-oligosaccharides supplementation to suckling piglets: Assessment of pre- and post-weaning performance and gut health
Source: PLoS One. 2020 Jun 5;15(6):e0233910. doi: 10.1371/journal.pone.0233910 (PMC7274435; doi:10.1371/journal.pone.0233910)
Supplement: S6 Data — (PDF) [file pone.0233910.s008.pdf]

Image Report: PCNA-Casp3-LADDER_analyse

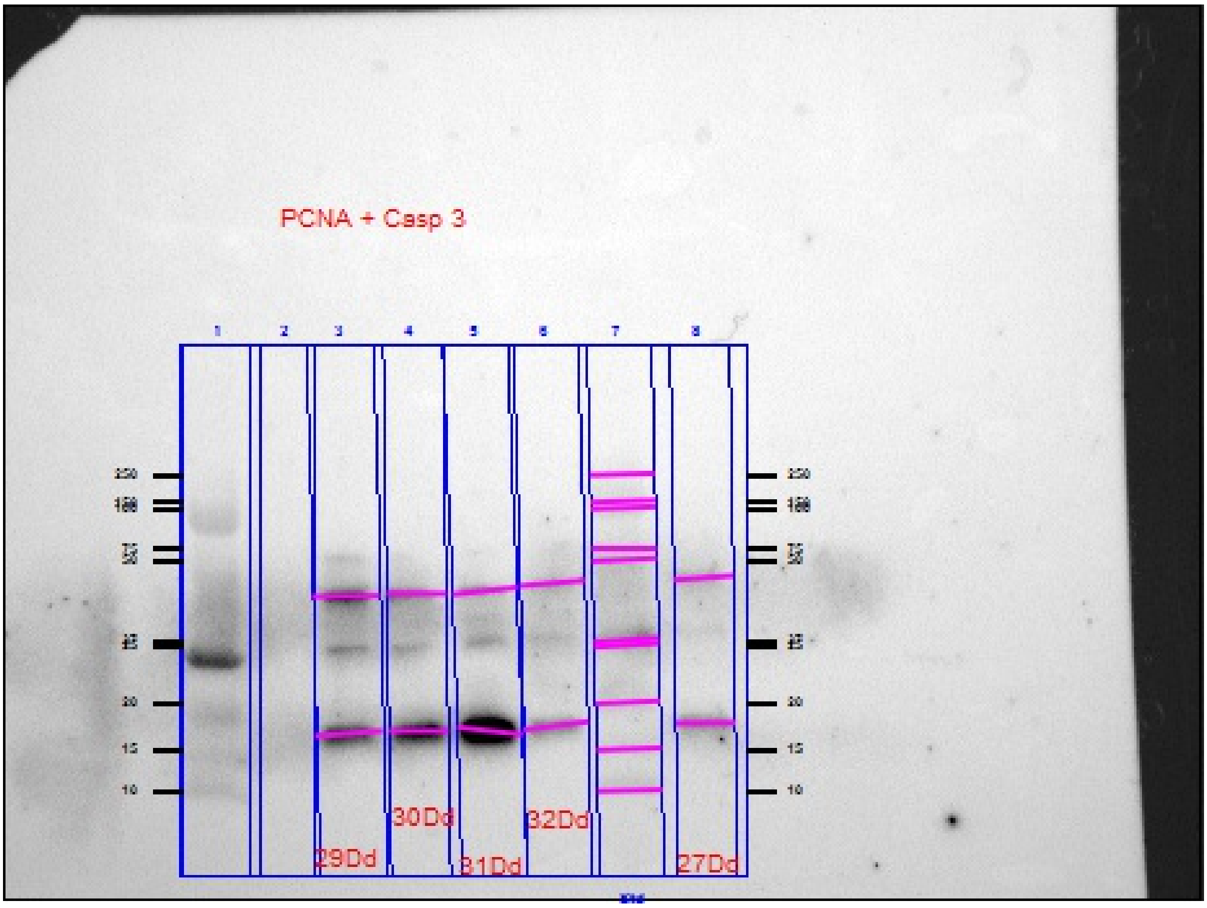

C:\Users\Bio-Rad\Desktop\Katty_Tereos\Dd28-32Dd\PCNA-Casp3-LADDER_analyse.scn

Acquisition Information

Imager	Merged Image

Image Information

Acquisition Date	16/05/2017 13:44:36
User Name	Bio-Rad
Image Area (mm)	X: 95.0 Y: 71.0
Pixel Size (um)	X: 204.7 Y: 205.1
Data Range (Int)	0 - 39035

Notes

Merged images:
Image 1: PCNA-3-LADDER
Image 2: PCNA-1

Analysis Settings

Detection	Lane detection:<br>Manually created lanes

	Band detection:<br>Automatically detected bands with sensitivity: Low<br>Manually adjusted bands<br><br>Lane Background Subtraction:<br>Lane background subtracted with disk size: 10<br><br>Lane width: Variable
Mol. Weight Analysis	Standard: Bio-Rad Precision Plus<br>Standard lanes: 7<br>Regression method: Point to Point (semi-log)

Lane And Band Analysis

Lane 1

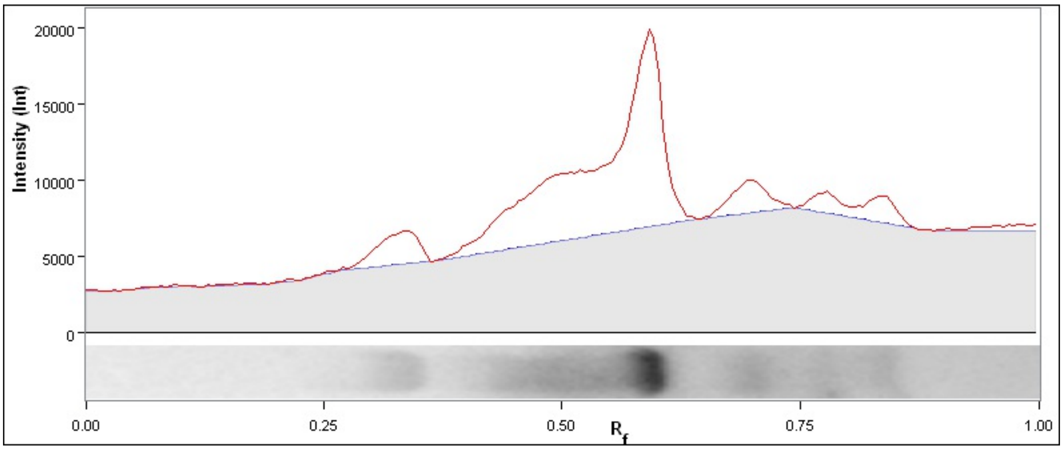

Band No.	Band Label	Mol. Wt. (KDa)	Relative Front	Volume (Int)	Abs. Quant.	Rel. Quant.	Band %	Lane %

Band Detection	Automatically detected bands with sensitivity: Low
Lane Background	Lane background subtracted with disk size: 10
Lane Width	5.32 mm
Regression Equation	A single equation is not available for this method

Lane 2

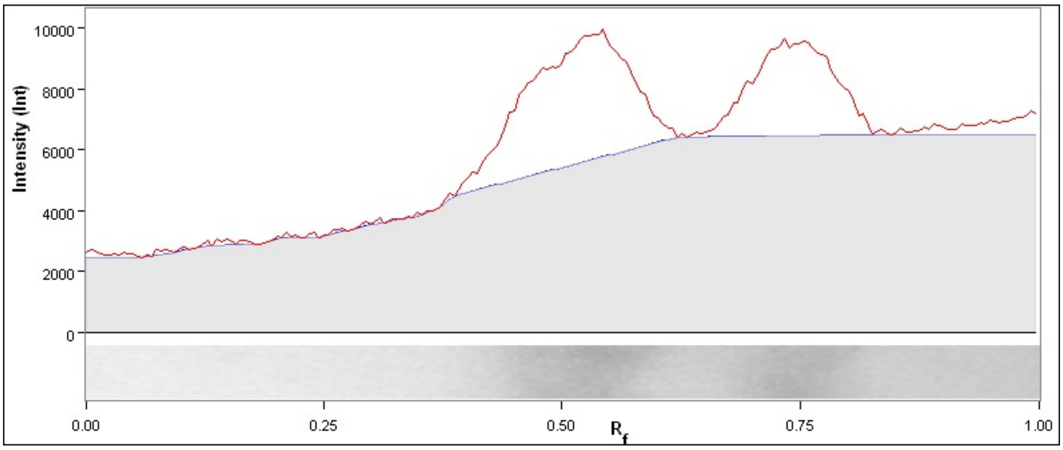

Band No.	Band Label	Mol. Wt. (KDa)	Relative Front	Volume (Int)	Abs. Quant.	Rel. Quant.	Band %	Lane %

Band Detection	Automatically detected bands with sensitivity: Low
Lane Background	Lane background subtracted with disk size: 10

Lane Width	4.30 mm
Regression Equation	A single equation is not available for this method

Lane 3

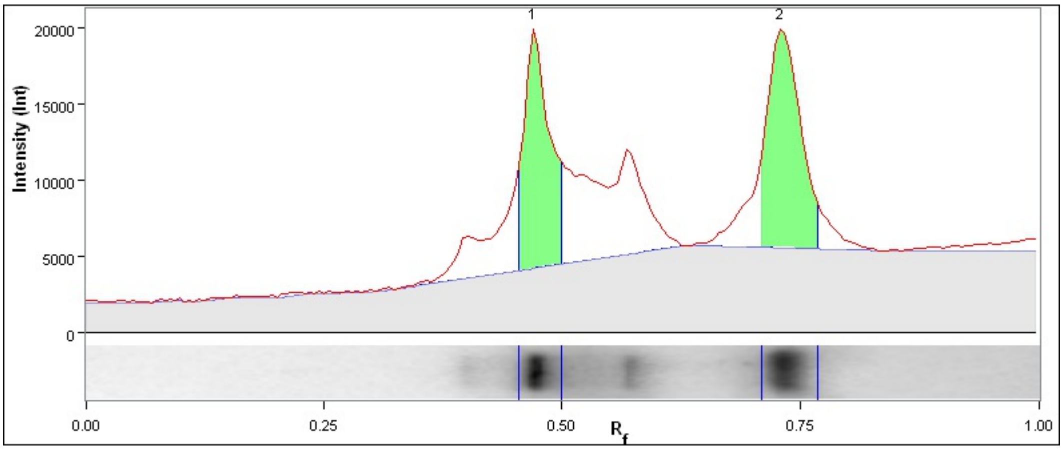

Band No.	Band Label	Mol. Wt. (KDa)	Relative Front	Volume (Int)	Abs. Quant.	Rel. Quant.	Band %	Lane %
1		43,6	0,473	3.315.936	N/A	N/A	46,7	24,3
2		16,5	0,732	3.778.814	N/A	N/A	53,3	27,6

Band Detection	Automatically detected bands with sensitivity: Low
Lane Background	Lane background subtracted with disk size: 10
Lane Width	5.32 mm
Regression Equation	A single equation is not available for this method

Lane 4

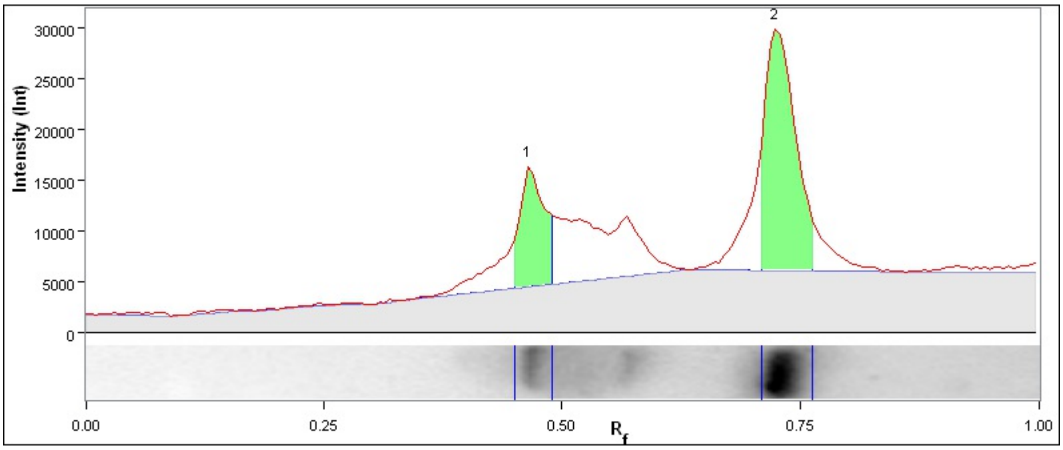

Band No.	Band Label	Mol. Wt. (KDa)	Relative Front	Volume (Int)	Abs. Quant.	Rel. Quant.	Band %	Lane %
1		44,1	0,468	1.899.846	N/A	N/A	30,2	16,0
2		16,8	0,727	4.392.540	N/A	N/A	69,8	36,9

Band Detection	Automatically detected bands with sensitivity: Low
Lane Background	Lane background subtracted with disk size: 10
Lane Width	4.71 mm
Regression Equation	A single equation is not available for this method

Lane 5

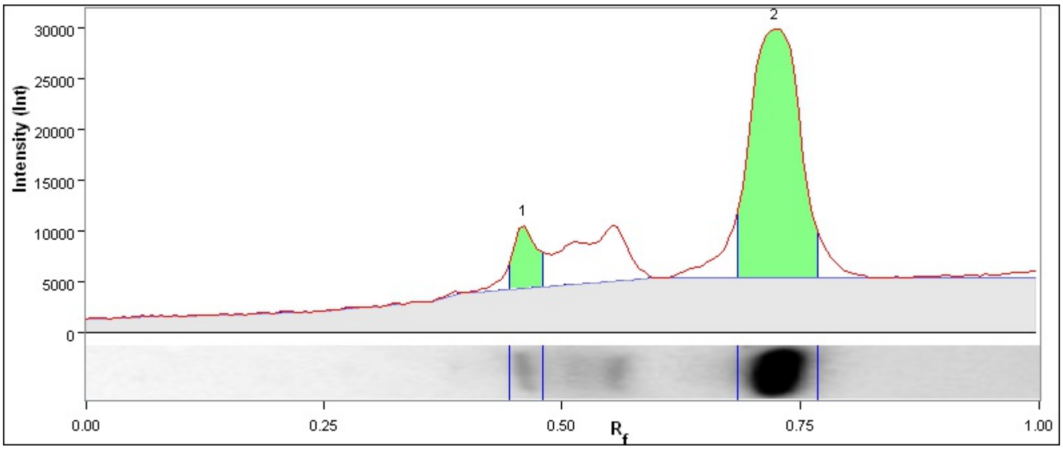

Band No.	Band Label	Mol. Wt. (KDa)	Relative Front	Volume (Int)	Abs. Quant.	Rel. Quant.	Band %	Lane %
1		44,5	0,463	967.525	N/A	N/A	10,1	7,1
2		16,8	0,727	8.573.450	N/A	N/A	89,9	63,0

Band Detection	Automatically detected bands with sensitivity: Low
Lane Background	Lane background subtracted with disk size: 10
Lane Width	5.12 mm
Regression Equation	A single equation is not available for this method

Lane 6

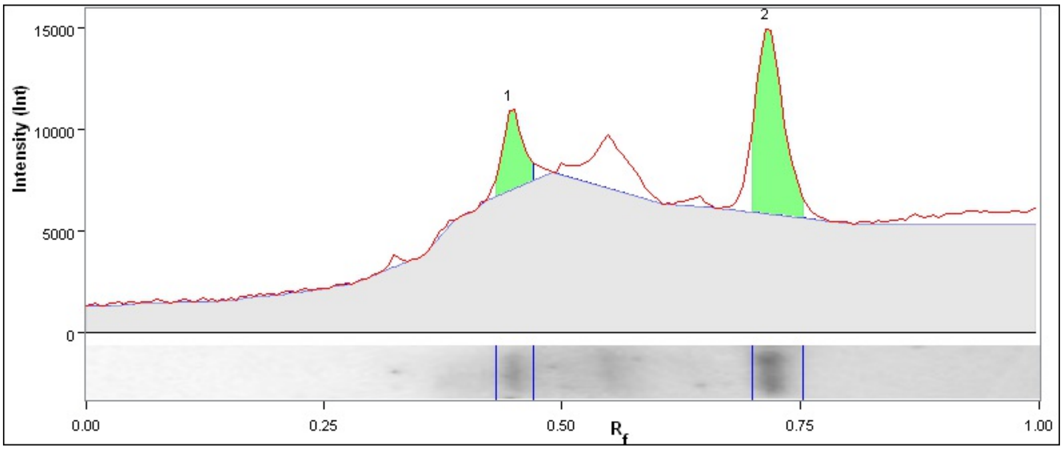

Band No.	Band Label	Mol. Wt. (KDa)	Relative Front	Volume (Int)	Abs. Quant.	Rel. Quant.	Band %	Lane %
1		45,8	0,449	515.200	N/A	N/A	24,3	13,4
2		17,3	0,717	1.601.125	N/A	N/A	75,7	41,7

Band Detection	Automatically detected bands with sensitivity: Low
Lane Background	Lane background subtracted with disk size: 10
Lane Width	5.12 mm
Regression Equation	A single equation is not available for this method

Lane 7 - Bio-Rad Precision Plus

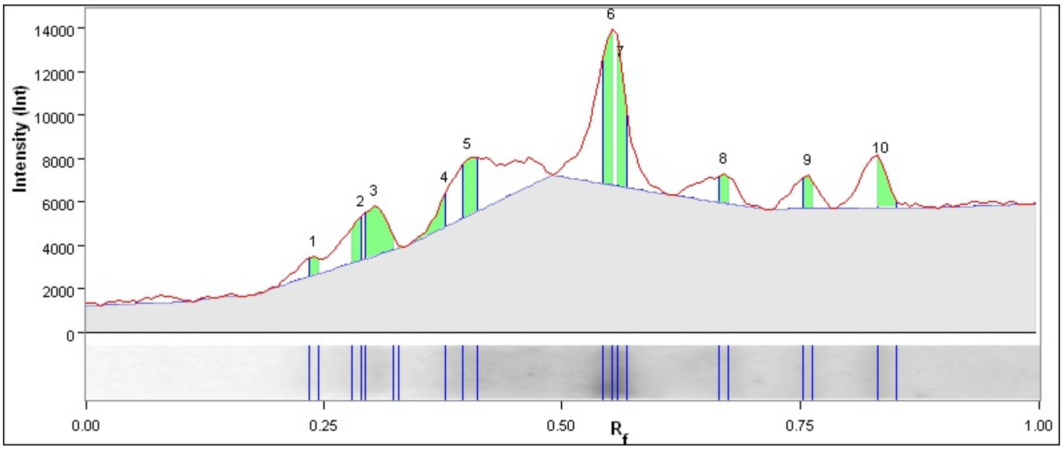

Band No.	Band Label	Mol. Wt. (KDa)	Relative Front	Volume (Int)	Abs. Quant.	Rel. Quant.	Band %	Lane %
1		250,0	0,244	59.300	N/A	N/A	2,7	1,2
2		150,0	0,298	167.600	N/A	N/A	7,6	3,4
3		100,0	0,307	281.875	N/A	N/A	12,8	5,7
4		75,0	0,385	183.450	N/A	N/A	8,3	3,7
5		50,0	0,405	279.075	N/A	N/A	12,6	5,7
6		37,0	0,556	587.375	N/A	N/A	26,6	12,0
7		25,0	0,566	316.075	N/A	N/A	14,3	6,4
8		20,0	0,673	101.950	N/A	N/A	4,6	2,1
9		15,0	0,761	101.950	N/A	N/A	4,6	2,1
10		10,0	0,839	129.000	N/A	N/A	5,8	2,6

Band Detection	Automatically detected bands with sensitivity: Low
Lane Background	Lane background subtracted with disk size: 10
Lane Width	5.12 mm
Regression Equation	A single equation is not available for this method

Lane 8

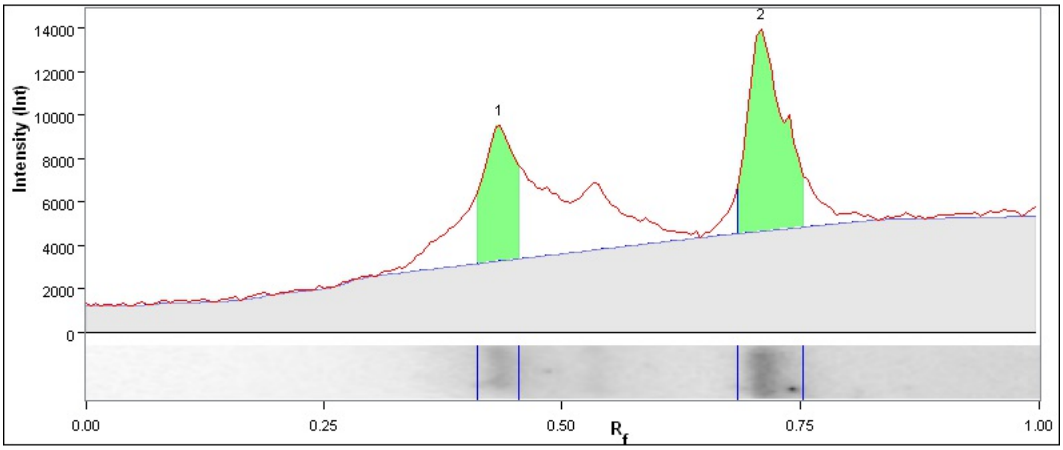

Band No.	Band Label	Mol. Wt. (KDa)	Relative Front	Volume (Int)	Abs. Quant.	Rel. Quant.	Band %	Lane %
1		46,7	0,439	1.324.478	N/A	N/A	37,5	19,7
2		17,6	0,712	2.207.172	N/A	N/A	62,5	32,9

Band Detection	Automatically detected bands with sensitivity: Low
Lane Background	Lane background subtracted with disk size: 10
Lane Width	4.71 mm
Regression Equation	A single equation is not available for this method
