# Supplementary material for: Short-chain fructo-oligosaccharides supplementation to suckling piglets: Assessment of pre- and post-weaning performance and gut health
Source: PLoS One. 2020 Jun 5;15(6):e0233910. doi: 10.1371/journal.pone.0233910 (PMC7274435; doi:10.1371/journal.pone.0233910)
Supplement: S7 Data — (PDF) [file pone.0233910.s009.pdf]

Image Report: PCNA-Casp3_LADDER_analyse

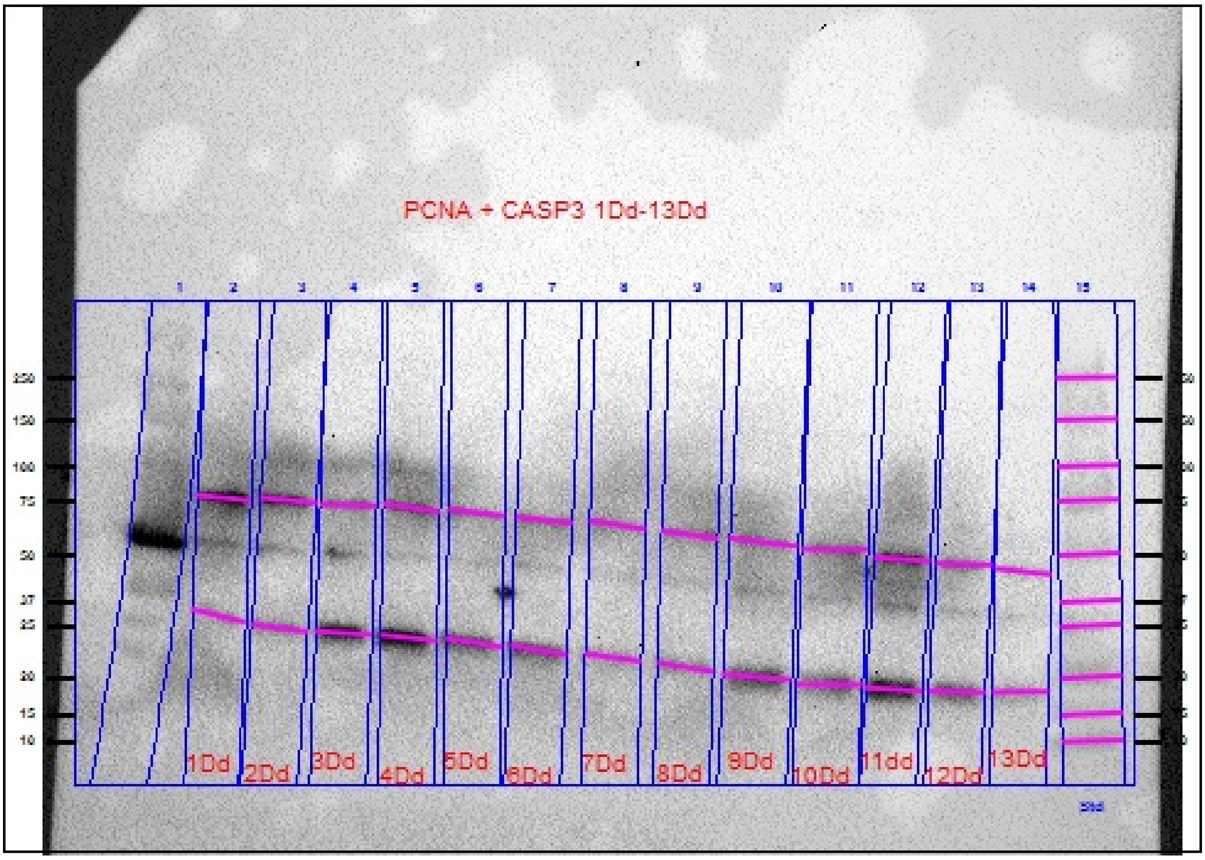

C:\Users\Bio-Rad\Desktop\Katty_Tereos\Dd1-13Dd\PCNA-Casp3_LADDER_analyse.scn

---

Acquisition Information

Imager	Merged Image

Image Information

Acquisition Date	16/05/2017 11:42:13
User Name	Bio-Rad
Image Area (mm)	X: 95.0 Y: 71.0
Pixel Size (um)	X: 204.7 Y: 205.1
Data Range (Int)	0 - 62719

Notes

Merged images:
Image 1: PCNA-17_LADDER
Image 2: PCNA-17

Analysis Settings

Detection	Lane detection:<br>Manually created lanes<br><br>Band detection:<br>Automatically detected bands with sensitivity: Low

	Manually adjusted bands<br><br>Lane Background Subtraction:<br>Lane background subtracted with disk size: 10<br><br>Lane width: Variable
Mol. Weight Analysis	Standard: Bio-Rad Precision Plus<br>Standard lanes: last<br>Regression method: Point to Point (semi-log)

Lane And Band Analysis

Lane 1

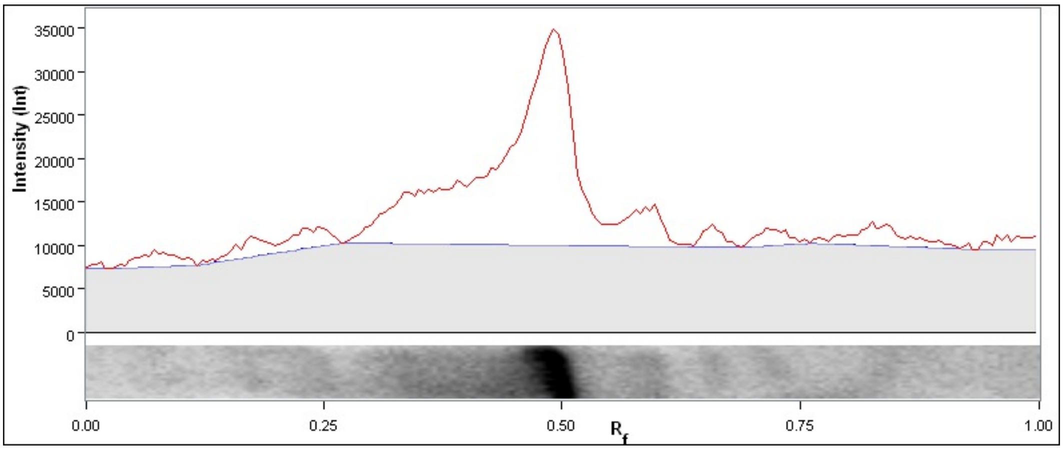

Band No.	Band Label	Mol. Wt. (KDa)	Relative Front	Volume (Int)	Abs. Quant.	Rel. Quant.	Band %	Lane %

Band Detection	Automatically detected bands with sensitivity: Low
Lane Background	Lane background subtracted with disk size: 10
Lane Width	4.71 mm
Regression Equation	A single equation is not available for this method

Lane 2

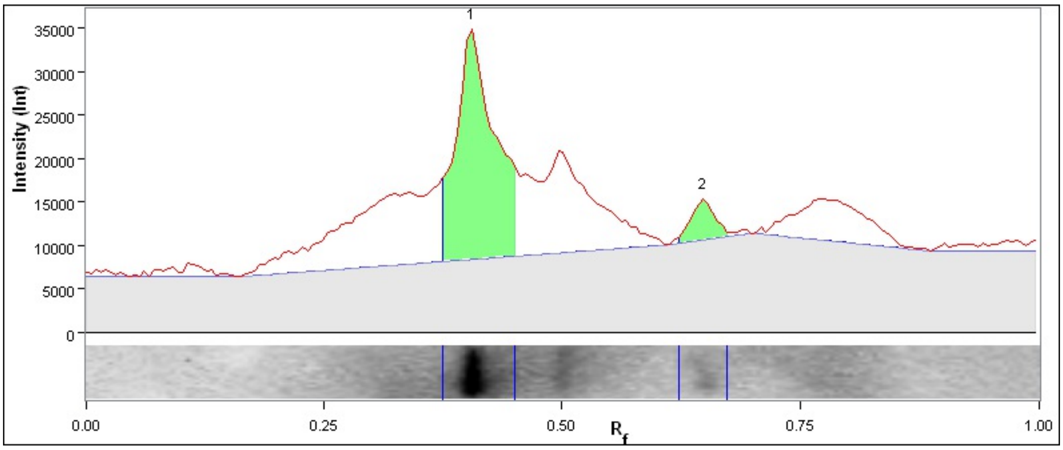

Band No.	Band Label	Mol. Wt. (KDa)	Relative Front	Volume (Int)	Abs. Quant.	Rel. Quant.	Band %	Lane %
1		76,6	0,409	5.778.498	N/A	N/A	90,4	32,1
2		29,2	0,652	610.434	N/A	N/A	9,6	3,4

Band Detection	Automatically detected bands with sensitivity: Low
Lane Background	Lane background subtracted with disk size: 10
Lane Width	4.50 mm

Regression Equation	A single equation is not available for this method

Lane 3

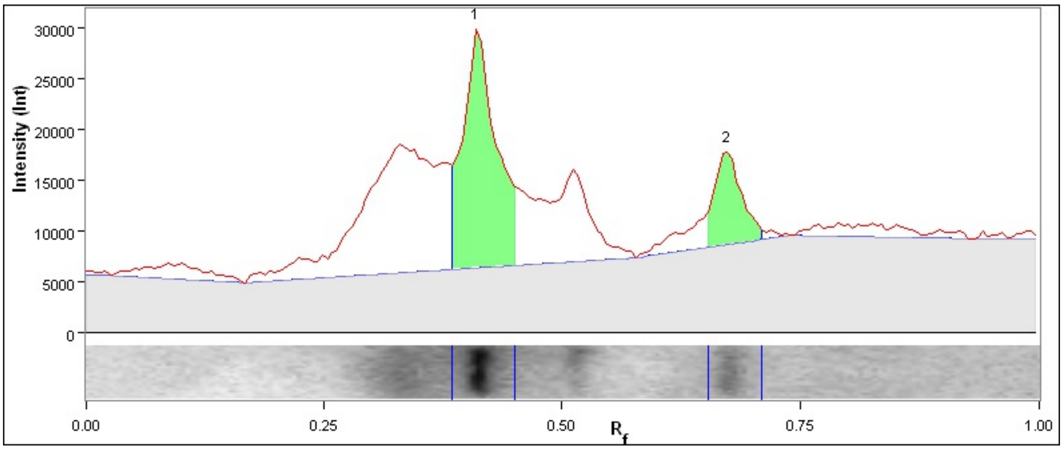

Band No.	Band Label	Mol. Wt. (KDa)	Relative Front	Volume (Int)	Abs. Quant.	Rel. Quant.	Band %	Lane %
1		75,0	0,414	4.672.519	N/A	N/A	77,0	27,3
2		24,7	0,677	1.399.435	N/A	N/A	23,0	8,2

Band Detection	Automatically detected bands with sensitivity: Low
Lane Background	Lane background subtracted with disk size: 10
Lane Width	4.71 mm
Regression Equation	A single equation is not available for this method

Lane 4

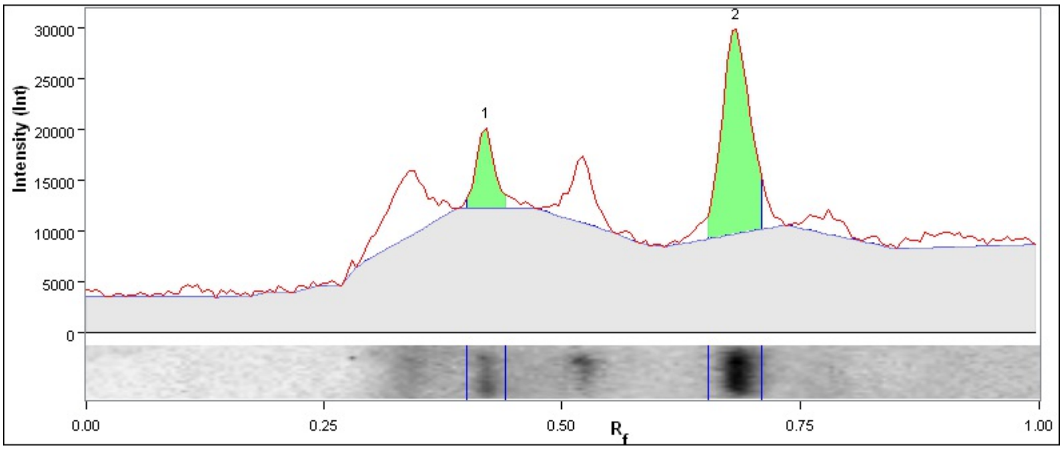

Band No.	Band Label	Mol. Wt. (KDa)	Relative Front	Volume (Int)	Abs. Quant.	Rel. Quant.	Band %	Lane %
1		72,3	0,424	857.026	N/A	N/A	20,2	9,8
2		24,2	0,687	3.392.270	N/A	N/A	79,8	39,0

Band Detection	Automatically detected bands with sensitivity: Low
Lane Background	Lane background subtracted with disk size: 10
Lane Width	4.71 mm
Regression Equation	A single equation is not available for this method

Lane 5

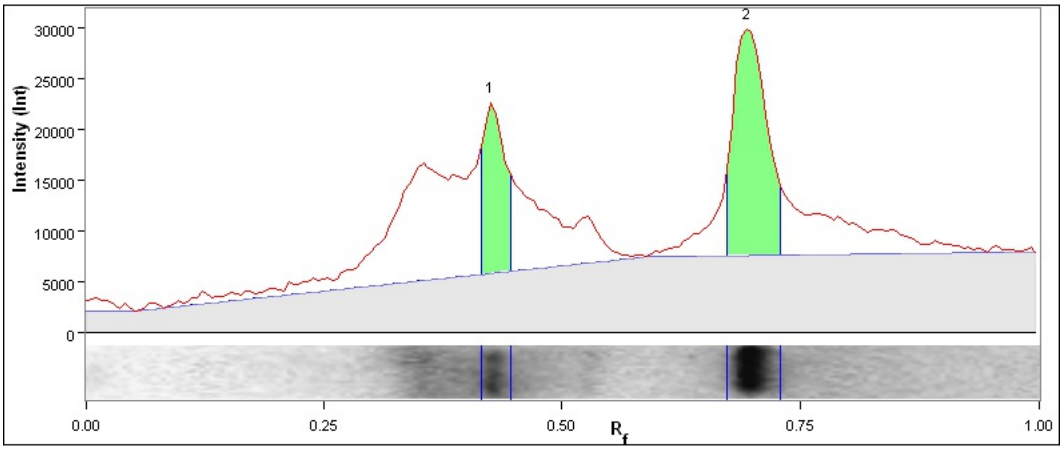

Band No.	Band Label	Mol. Wt. (KDa)	Relative Front	Volume (Int)	Abs. Quant.	Rel. Quant.	Band %	Lane %
1		71,0	0,429	2.313.823	N/A	N/A	33,0	11,7
2		23,7	0,697	4.699.590	N/A	N/A	67,0	23,7

Band Detection	Automatically detected bands with sensitivity: Low
Lane Background	Lane background subtracted with disk size: 10
Lane Width	4.71 mm
Regression Equation	A single equation is not available for this method

Lane 6

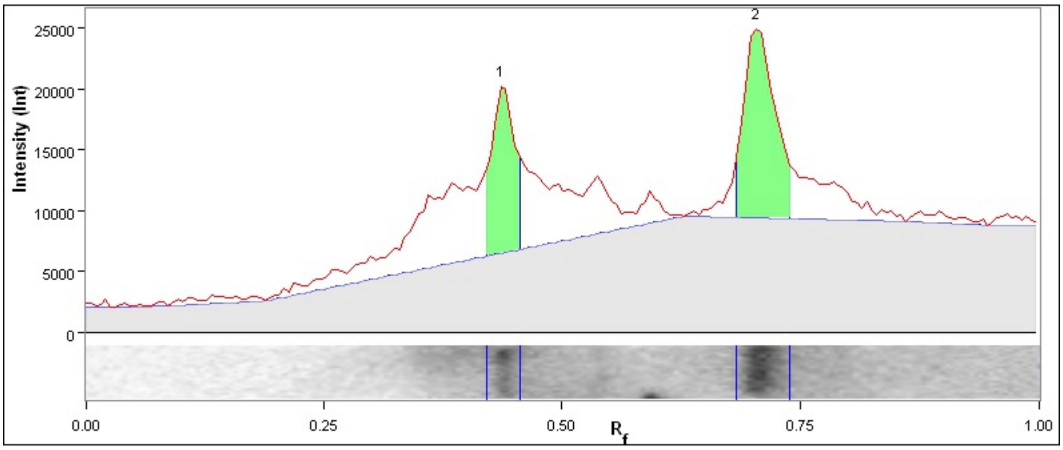

Band No.	Band Label	Mol. Wt. (KDa)	Relative Front	Volume (Int)	Abs. Quant.	Rel. Quant.	Band %	Lane %
1		68,4	0,439	1.854.582	N/A	N/A	40,3	15,8
2		23,2	0,707	2.746.499	N/A	N/A	59,7	23,3

Band Detection	Automatically detected bands with sensitivity: Low
Lane Background	Lane background subtracted with disk size: 10
Lane Width	4.71 mm
Regression Equation	A single equation is not available for this method

Lane 7

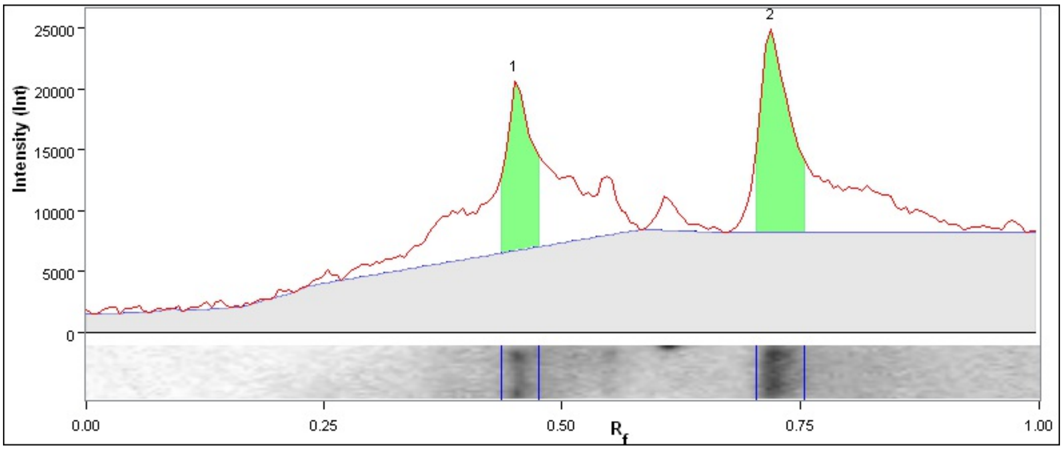

Band No.	Band Label	Mol. Wt. (KDa)	Relative Front	Volume (Int)	Abs. Quant.	Rel. Quant.	Band %	Lane %
1		64,7	0,455	2.103.465	N/A	N/A	42,5	17,7
2		22,5	0,722	2.841.190	N/A	N/A	57,5	23,9

Band Detection	Automatically detected bands with sensitivity: Low
Lane Background	Lane background subtracted with disk size: 10
Lane Width	4.71 mm
Regression Equation	A single equation is not available for this method

Lane 8

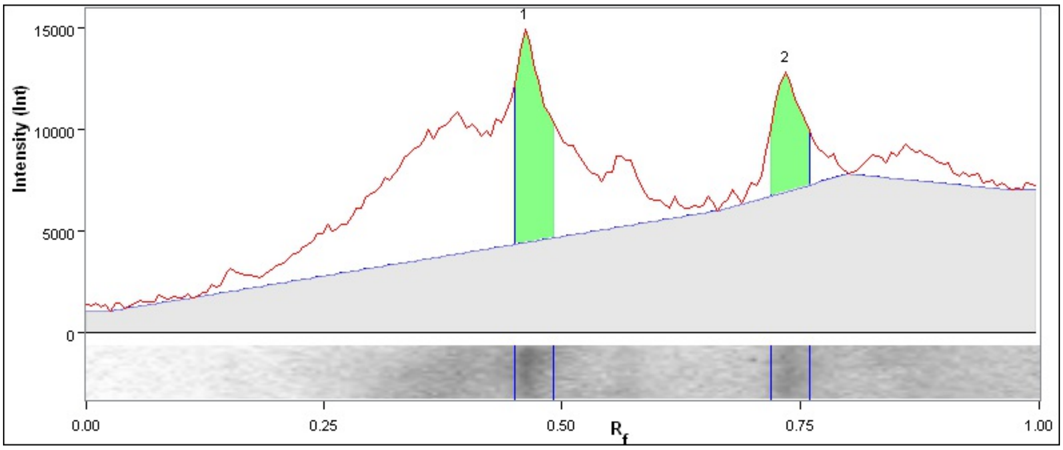

Band No.	Band Label	Mol. Wt. (KDa)	Relative Front	Volume (Int)	Abs. Quant.	Rel. Quant.	Band %	Lane %
1		62,4	0,465	2.008.567	N/A	N/A	65,0	15,5
2		21,8	0,737	1.080.678	N/A	N/A	35,0	8,3

Band Detection	Automatically detected bands with sensitivity: Low
Lane Background	Lane background subtracted with disk size: 10
Lane Width	4.71 mm
Regression Equation	A single equation is not available for this method

Lane 9

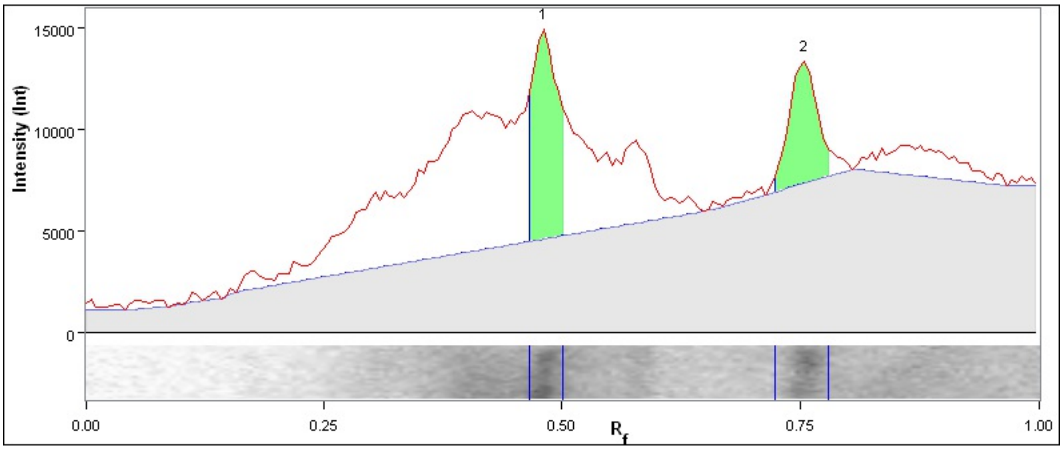

Band No.	Band Label	Mol. Wt. (KDa)	Relative Front	Volume (Int)	Abs. Quant.	Rel. Quant.	Band %	Lane %
1		57,9	0,485	1.963.142	N/A	N/A	60,8	14,9
2		20,9	0,758	1.265.759	N/A	N/A	39,2	9,6

Band Detection	Automatically detected bands with sensitivity: Low
Lane Background	Lane background subtracted with disk size: 10
Lane Width	4.71 mm
Regression Equation	A single equation is not available for this method

Lane 10

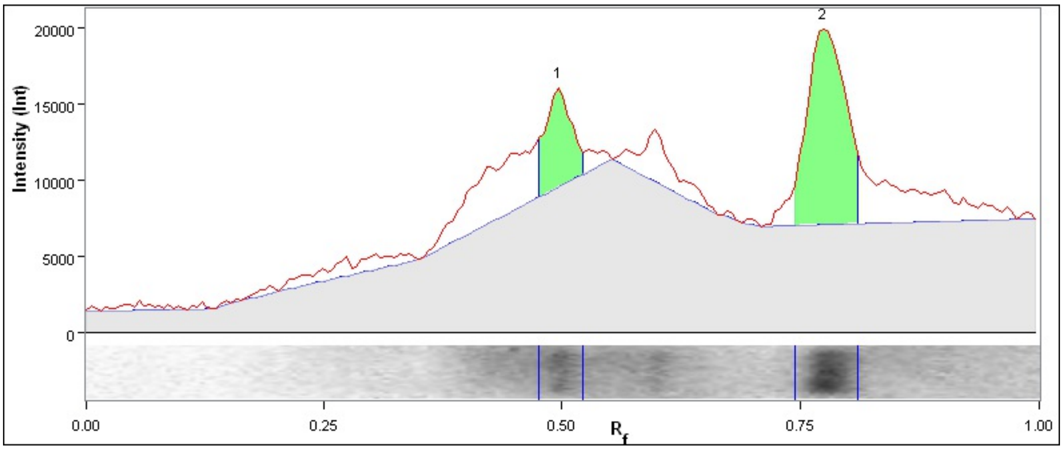

Band No.	Band Label	Mol. Wt. (KDa)	Relative Front	Volume (Int)	Abs. Quant.	Rel. Quant.	Band %	Lane %
1		54,8	0,500	1.300.796	N/A	N/A	24,2	11,1
2		20,0	0,778	4.079.600	N/A	N/A	75,8	34,9

Band Detection	Automatically detected bands with sensitivity: Low
Lane Background	Lane background subtracted with disk size: 10
Lane Width	5.73 mm
Regression Equation	A single equation is not available for this method

Lane 11

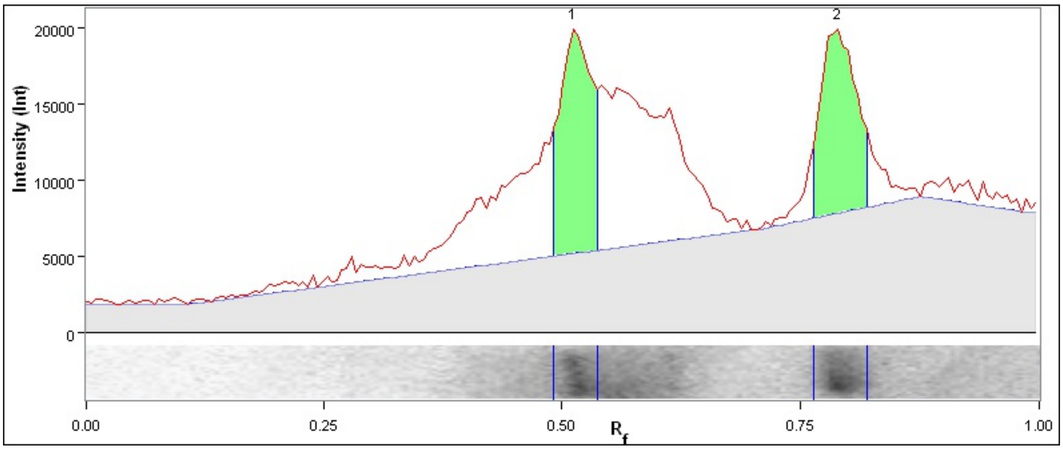

Band No.	Band Label	Mol. Wt. (KDa)	Relative Front	Volume (Int)	Abs. Quant.	Rel. Quant.	Band %	Lane %
1		51,9	0,515	3.087.612	N/A	N/A	51,4	18,4
2		18,9	0,793	2.918.592	N/A	N/A	48,6	17,4

Band Detection	Automatically detected bands with sensitivity: Low
Lane Background	Lane background subtracted with disk size: 10
Lane Width	5.53 mm
Regression Equation	A single equation is not available for this method

Lane 12

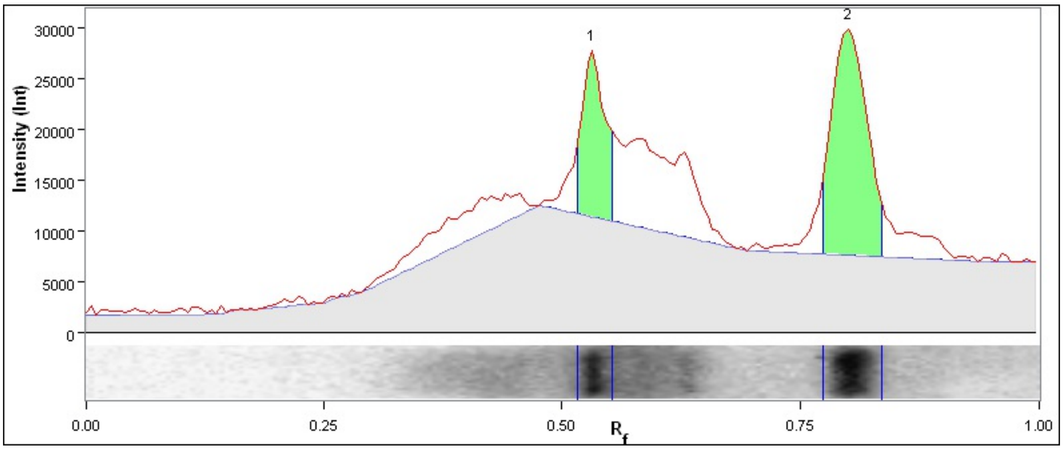

Band No.	Band Label	Mol. Wt. (KDa)	Relative Front	Volume (Int)	Abs. Quant.	Rel. Quant.	Band %	Lane %
1		48,4	0,535	2.318.676	N/A	N/A	33,3	15,9
2		18,2	0,803	4.654.349	N/A	N/A	66,7	31,9

Band Detection	Automatically detected bands with sensitivity: Low
Lane Background	Lane background subtracted with disk size: 10
Lane Width	4.71 mm
Regression Equation	A single equation is not available for this method

Lane 13

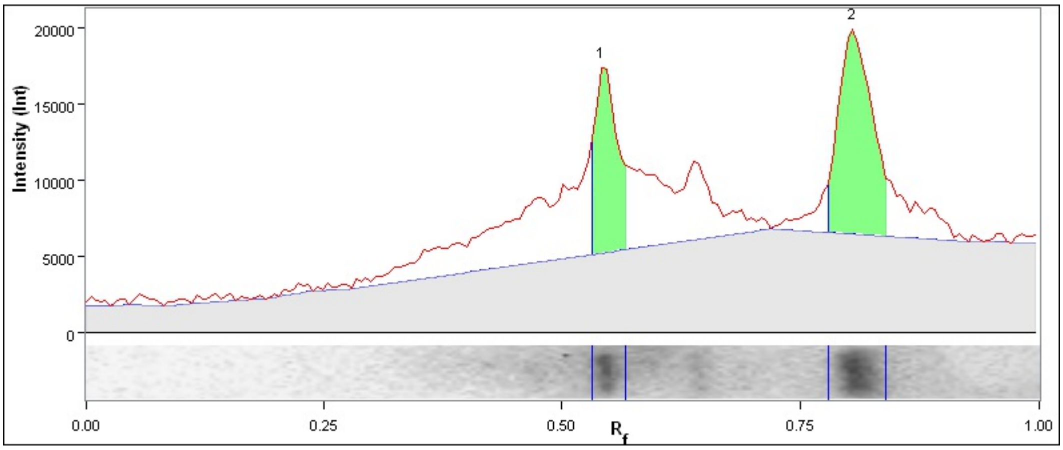

Band No.	Band Label	Mol. Wt. (KDa)	Relative Front	Volume (Int)	Abs. Quant.	Rel. Quant.	Band %	Lane %
1		46,9	0,545	1.761.409	N/A	N/A	37,8	15,2
2		17,8	0,808	2.894.044	N/A	N/A	62,2	24,9

Band Detection	Automatically detected bands with sensitivity: Low
Lane Background	Lane background subtracted with disk size: 10
Lane Width	4.71 mm
Regression Equation	A single equation is not available for this method

Lane 14

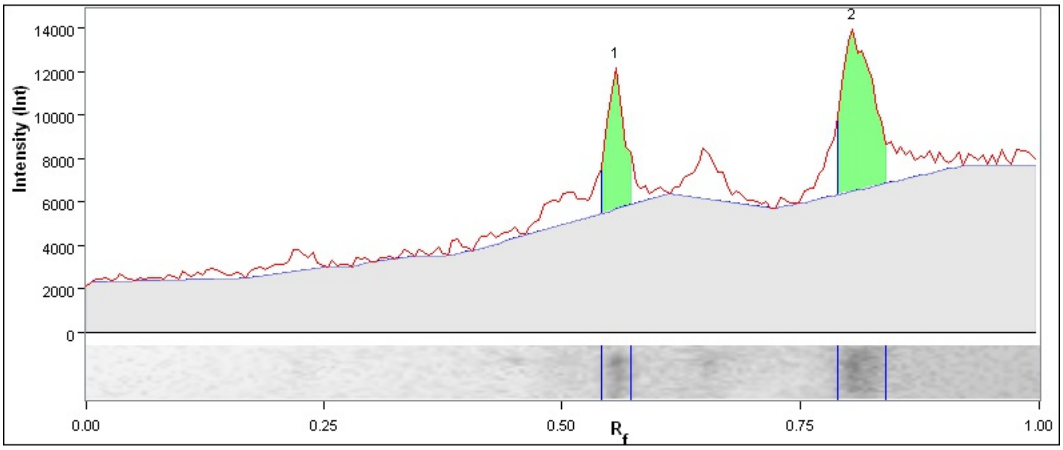

Band No.	Band Label	Mol. Wt. (KDa)	Relative Front	Volume (Int)	Abs. Quant.	Rel. Quant.	Band %	Lane %
1		44,7	0,561	665.413	N/A	N/A	33,4	15,5
2		17,8	0,808	1.328.549	N/A	N/A	66,6	30,9

Band Detection	Automatically detected bands with sensitivity: Low
Lane Background	Lane background subtracted with disk size: 10
Lane Width	4.71 mm
Regression Equation	A single equation is not available for this method

Lane 15 - Bio-Rad Precision Plus

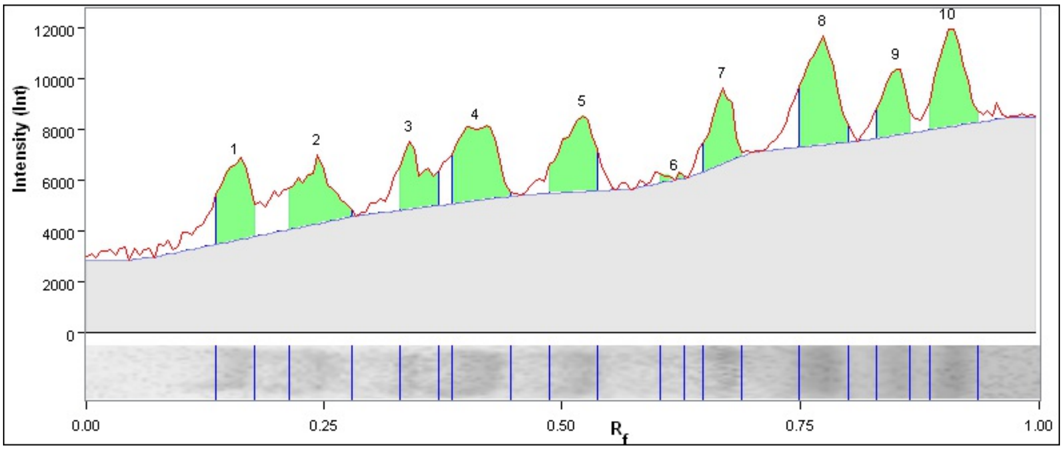

Band No.	Band Label	Mol. Wt. (KDa)	Relative Front	Volume (Int)	Abs. Quant.	Rel. Quant.	Band %	Lane %
1		250,0	0,162	593.600	N/A	N/A	10,9	8,3
2		150,0	0,247	572.550	N/A	N/A	10,5	8,0
3		100,0	0,343	402.925	N/A	N/A	7,4	5,6
4		75,0	0,414	767.650	N/A	N/A	14,1	10,8
5		50,0	0,525	707.550	N/A	N/A	13,0	9,9
6		37,0	0,621	23.050	N/A	N/A	0,4	0,3
7		25,0	0,672	423.975	N/A	N/A	7,8	5,9
8		20,0	0,778	834.150	N/A	N/A	15,4	11,7
9		15,0	0,854	399.800	N/A	N/A	7,4	5,6
10		10,0	0,909	707.300	N/A	N/A	13,0	9,9

Band Detection	Automatically detected bands with sensitivity: Low
Lane Background	Lane background subtracted with disk size: 10
Lane Width	5.12 mm
Regression Equation	A single equation is not available for this method
