# Supplementary material for: Short-chain fructo-oligosaccharides supplementation to suckling piglets: Assessment of pre- and post-weaning performance and gut health
Source: PLoS One. 2020 Jun 5;15(6):e0233910. doi: 10.1371/journal.pone.0233910 (PMC7274435; doi:10.1371/journal.pone.0233910)
Supplement: S8 Data — (PDF) [file pone.0233910.s010.pdf]

Image Report: PCNA-Casp3-LADDER_analyse

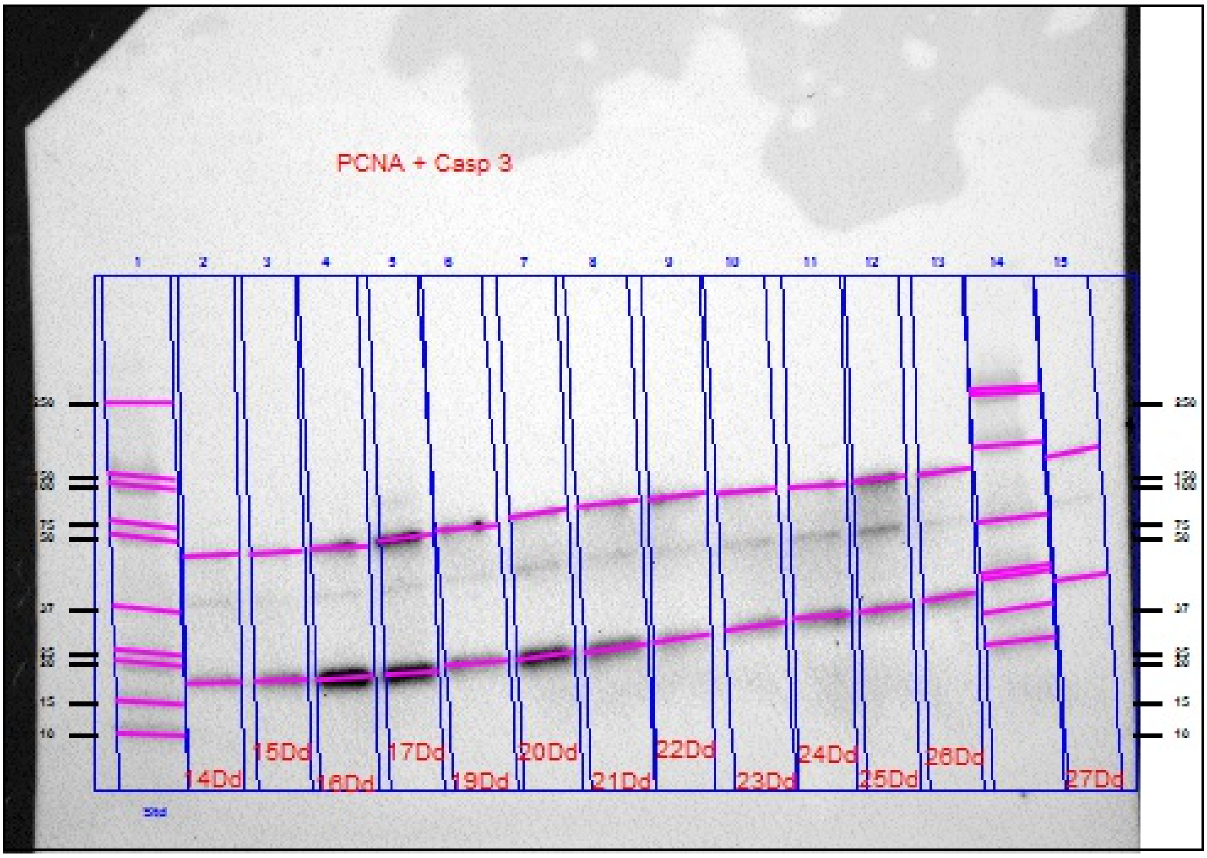

C:\Users\Bio-Rad\Desktop\Katty_Tereos\Dd14-27Dd\PCNA-Casp3-LADDER_analyse.scn

---

Acquisition Information

Imager	Merged Image

Image Information

Acquisition Date	16/05/2017 13:24:02
User Name	Bio-Rad
Image Area (mm)	X: 95.0 Y: 71.0
Pixel Size (um)	X: 204.7 Y: 205.1
Data Range (Int)	0 - 63696

Notes

Merged images:
Image 1: PCNA15-LADDER
Image 2: PCNA15

Analysis Settings

Detection	Lane detection:<br>Manually created lanes<br><br>Band detection:<br><br>Manually adjusted bands

	Lane Background Subtraction:<br>Lane background subtracted with disk size: 10<br><br>Lane width: Variable
Mol. Weight Analysis	Standard: Bio-Rad Precision Plus<br>Standard lanes: first<br>Regression method: Point to Point (semi-log)

Lane And Band Analysis

Lane 1 - Bio-Rad Precision Plus

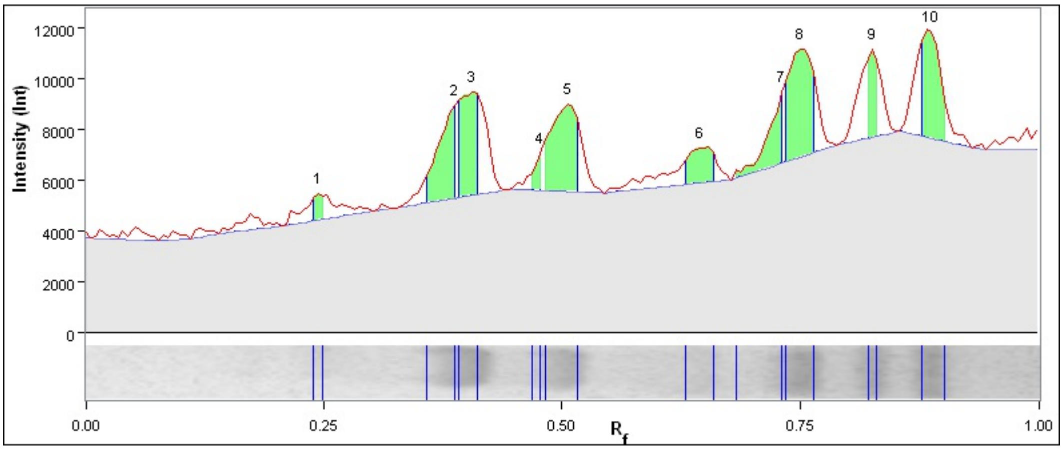

Band No.	Band Label	Mol. Wt. (KDa)	Relative Front	Volume (Int)	Abs. Quant.	Rel. Quant.	Band %	Lane %
1		250,0	0,248	86.632	N/A	N/A	2,0	1,4
2		150,0	0,390	586.236	N/A	N/A	13,6	9,4
3		100,0	0,410	584.164	N/A	N/A	13,5	9,3
4		75,0	0,486	155.652	N/A	N/A	3,6	2,5
5		50,0	0,510	691.348	N/A	N/A	16,0	11,0
6		37,0	0,648	252.672	N/A	N/A	5,9	4,0
7		25,0	0,738	423.584	N/A	N/A	9,8	6,8
8		20,0	0,752	761.152	N/A	N/A	17,6	12,1
9		15,0	0,829	254.576	N/A	N/A	5,9	4,1
10		10,0	0,890	518.280	N/A	N/A	12,0	8,3

Lane Background	Lane background subtracted with disk size: 10
Lane Width	5.73 mm
Regression Equation	A single equation is not available for this method

Lane 2

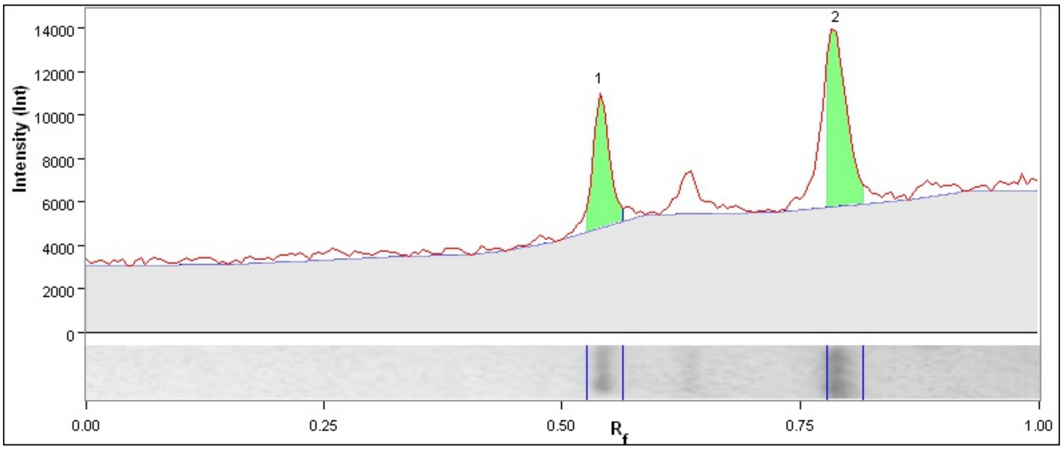

Band No.	Band Label	Mol. Wt. (KDa)	Relative Front	Volume (Int)	Abs. Quant.	Rel. Quant.	Band %	Lane %
1		46,5	0,543	608.419	N/A	N/A	41,0	19,0
2		17,3	0,790	875.679	N/A	N/A	59,0	27,3

Lane Background	Lane background subtracted with disk size: 10
Lane Width	4.71 mm
Regression Equation	A single equation is not available for this method

Lane 3

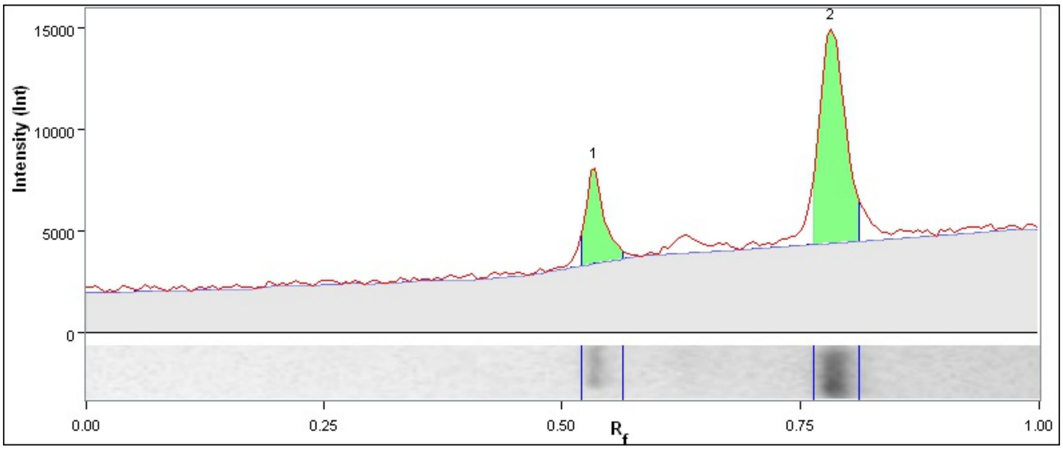

Band No.	Band Label	Mol. Wt. (KDa)	Relative Front	Volume (Int)	Abs. Quant.	Rel. Quant.	Band %	Lane %
1		47,0	0,538	583.308	N/A	N/A	23,6	15,7
2		17,6	0,786	1.883.530	N/A	N/A	76,4	50,6

Lane Background	Lane background subtracted with disk size: 10
Lane Width	4.50 mm
Regression Equation	A single equation is not available for this method

Lane 4

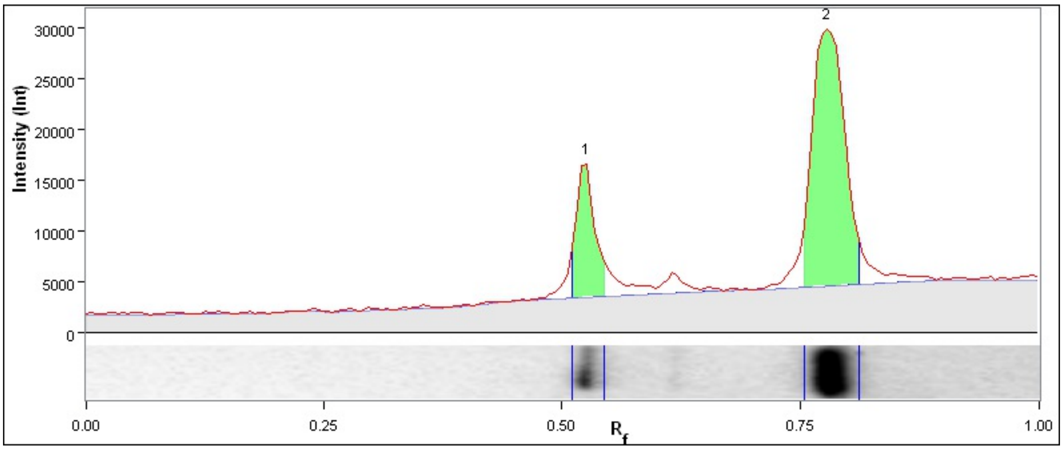

Band No.	Band Label	Mol. Wt. (KDa)	Relative Front	Volume (Int)	Abs. Quant.	Rel. Quant.	Band %	Lane %
1		48,0	0,529	1.689.288	N/A	N/A	22,7	17,5
2		18,0	0,781	5.742.360	N/A	N/A	77,3	59,5

Lane Background	Lane background subtracted with disk size: 10
Lane Width	4.91 mm
Regression Equation	A single equation is not available for this method

Lane 5

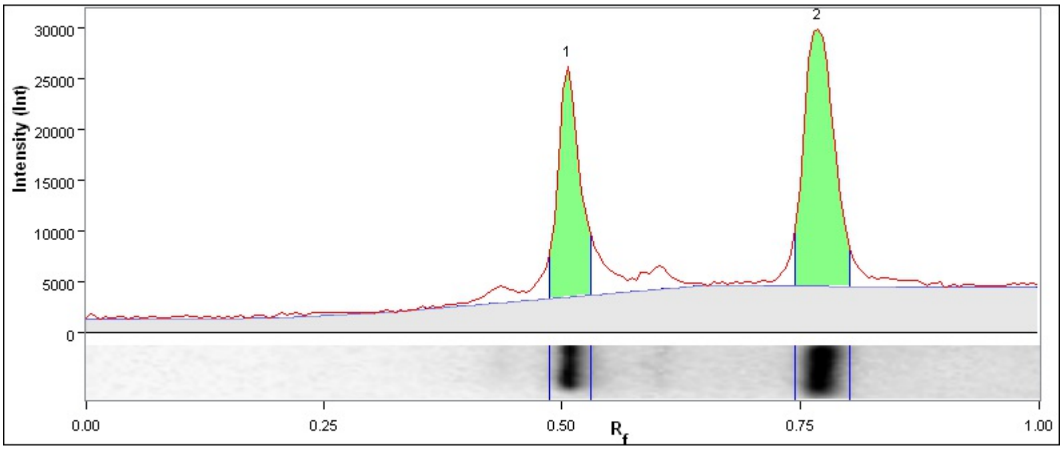

Band No.	Band Label	Mol. Wt. (KDa)	Relative Front	Volume (Int)	Abs. Quant.	Rel. Quant.	Band %	Lane %
1		50,0	0,510	3.123.010	N/A	N/A	39,4	29,0
2		18,6	0,771	4.813.226	N/A	N/A	60,6	44,7

Lane Background	Lane background subtracted with disk size: 10
Lane Width	4.50 mm
Regression Equation	A single equation is not available for this method

Lane 6

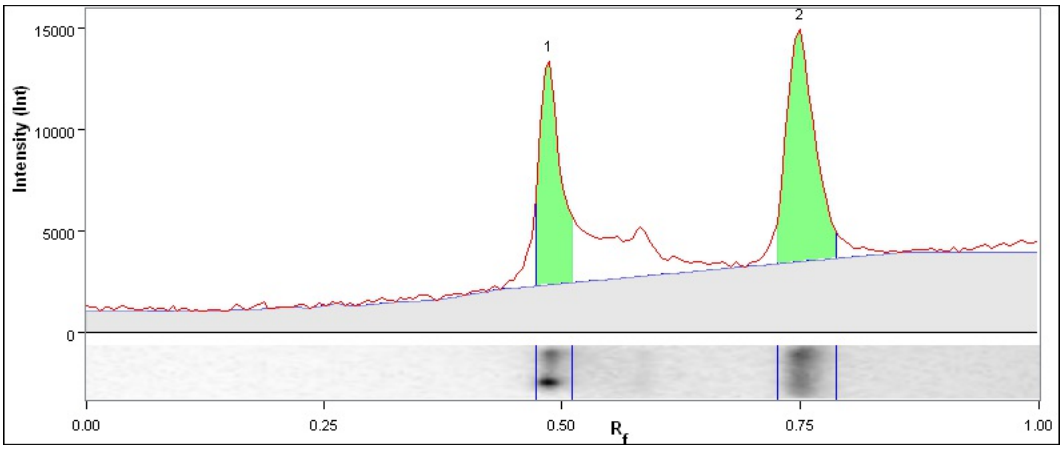

Band No.	Band Label	Mol. Wt. (KDa)	Relative Front	Volume (Int)	Abs. Quant.	Rel. Quant.	Band %	Lane %
1		67,0	0,490	1.803.958	N/A	N/A	41,3	26,2
2		20,0	0,752	2.567.552	N/A	N/A	58,7	37,3

Lane Background	Lane background subtracted with disk size: 10
Lane Width	5.32 mm
Regression Equation	A single equation is not available for this method

Lane 7

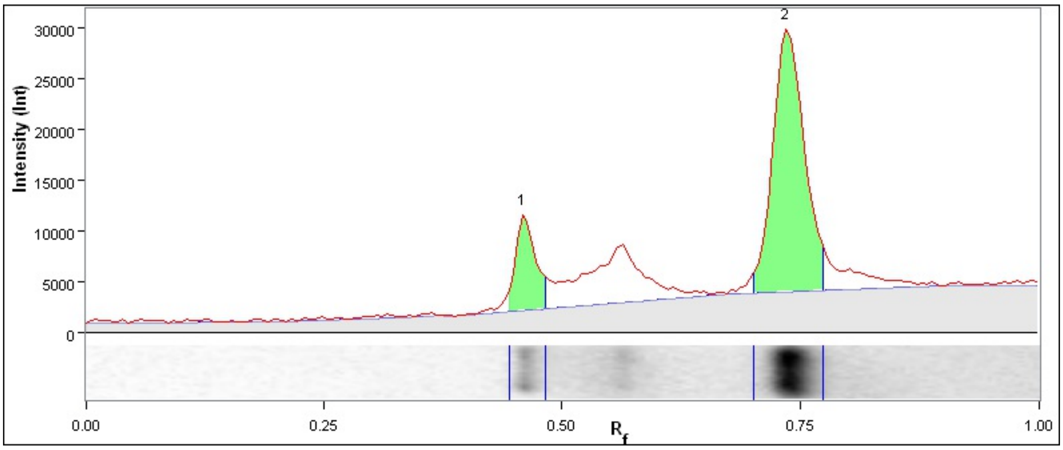

Band No.	Band Label	Mol. Wt. (KDa)	Relative Front	Volume (Int)	Abs. Quant.	Rel. Quant.	Band %	Lane %
1		81,5	0,462	1.214.328	N/A	N/A	19,0	12,3
2		23,3	0,738	5.167.848	N/A	N/A	81,0	52,4

Lane Background	Lane background subtracted with disk size: 10
Lane Width	4.91 mm
Regression Equation	A single equation is not available for this method

Lane 8

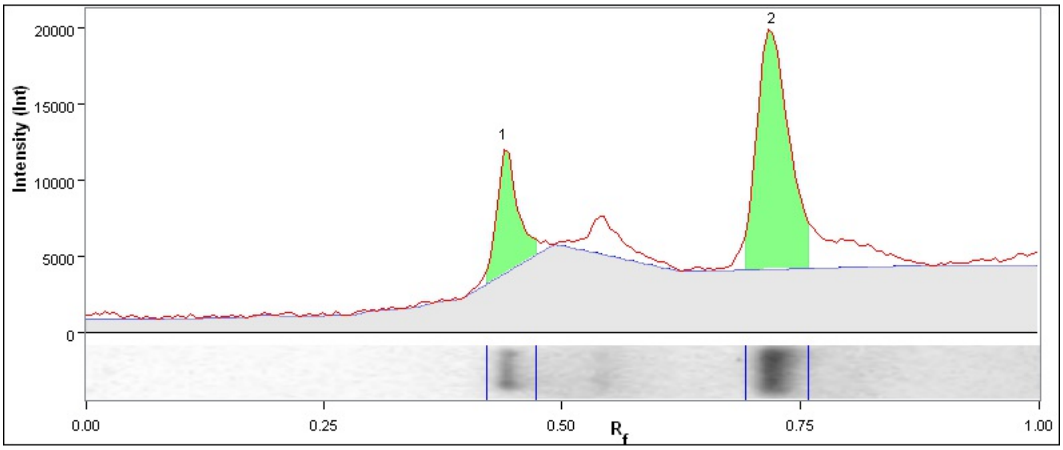

Band No.	Band Label	Mol. Wt. (KDa)	Relative Front	Volume (Int)	Abs. Quant.	Rel. Quant.	Band %	Lane %
1		87,8	0,443	1.153.334	N/A	N/A	24,7	16,9
2		25,9	0,724	3.516.032	N/A	N/A	75,3	51,6

Lane Background	Lane background subtracted with disk size: 10
Lane Width	5.32 mm
Regression Equation	A single equation is not available for this method

Lane 9

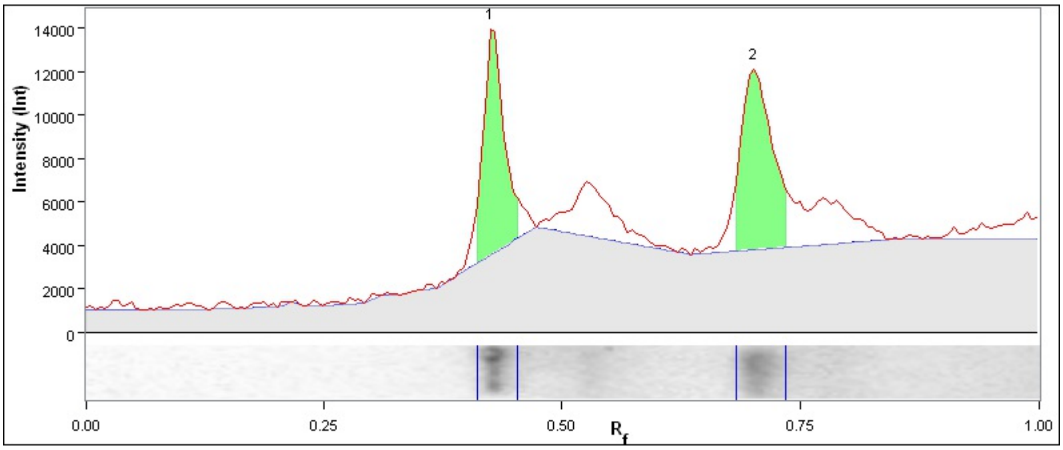

Band No.	Band Label	Mol. Wt. (KDa)	Relative Front	Volume (Int)	Abs. Quant.	Rel. Quant.	Band %	Lane %
1		92,8	0,429	1.327.176	N/A	N/A	45,2	25,1
2		28,3	0,705	1.609.944	N/A	N/A	54,8	30,4

Lane Background	Lane background subtracted with disk size: 10
Lane Width	4.91 mm
Regression Equation	A single equation is not available for this method

Lane 10

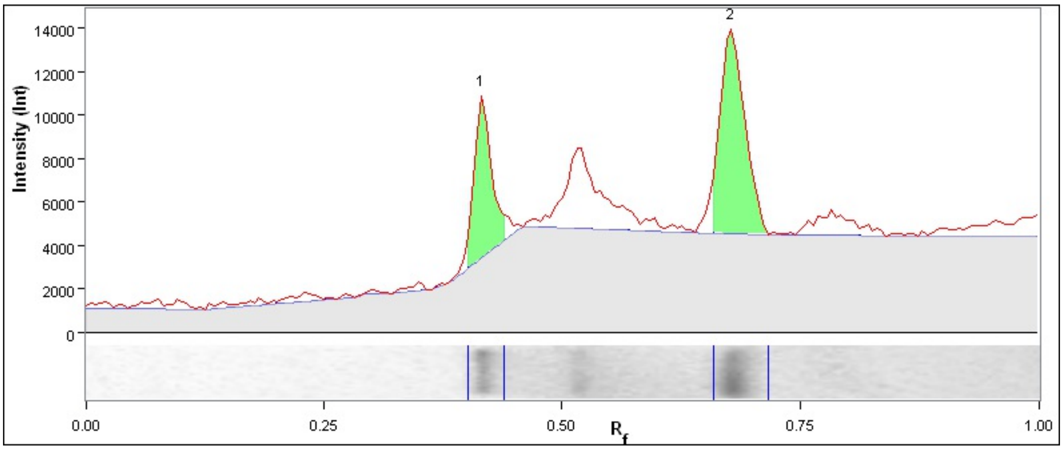

Band No.	Band Label	Mol. Wt. (KDa)	Relative Front	Volume (Int)	Abs. Quant.	Rel. Quant.	Band %	Lane %
1		96,3	0,419	833.150	N/A	N/A	36,1	18,4
2		31,7	0,681	1.473.725	N/A	N/A	63,9	32,6

Lane Background	Lane background subtracted with disk size: 10
Lane Width	5.12 mm
Regression Equation	A single equation is not available for this method

Lane 11

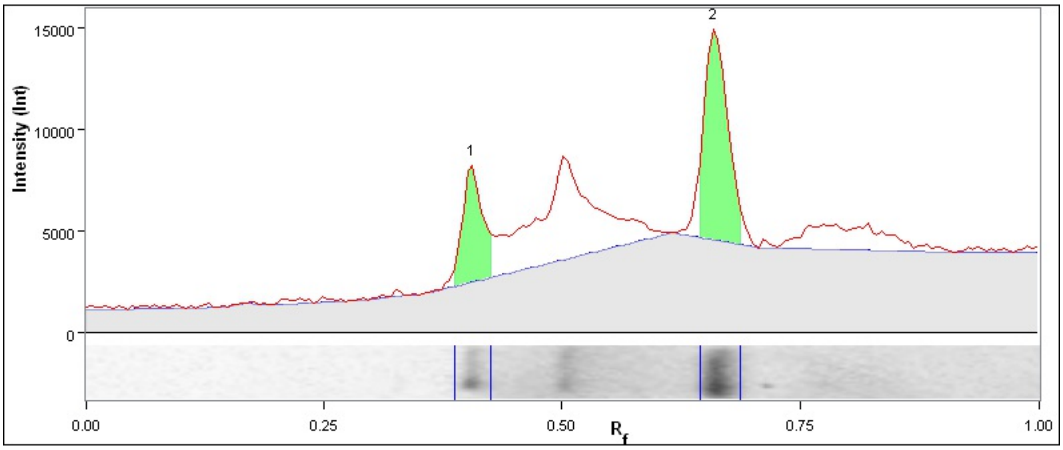

Band No.	Band Label	Mol. Wt. (KDa)	Relative Front	Volume (Int)	Abs. Quant.	Rel. Quant.	Band %	Lane %
1		100,0	0,410	888.216	N/A	N/A	32,8	13,7
2		34,6	0,662	1.820.088	N/A	N/A	67,2	28,1

Lane Background	Lane background subtracted with disk size: 10
Lane Width	4.91 mm
Regression Equation	A single equation is not available for this method

Lane 12

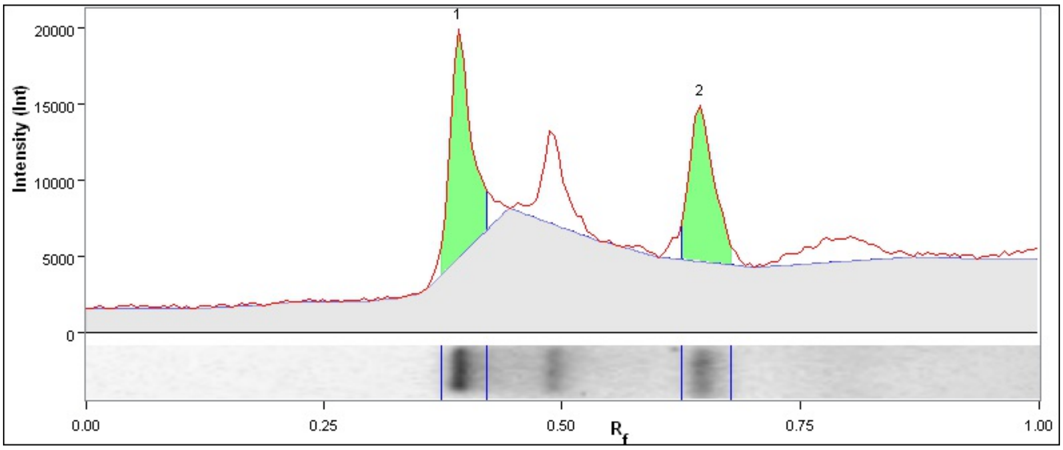

Band No.	Band Label	Mol. Wt. (KDa)	Relative Front	Volume (Int)	Abs. Quant.	Rel. Quant.	Band %	Lane %
1		135,5	0,395	1.922.624	N/A	N/A	55,3	33,3
2		37,0	0,648	1.553.552	N/A	N/A	44,7	26,9

Lane Background	Lane background subtracted with disk size: 10
Lane Width	4.50 mm
Regression Equation	A single equation is not available for this method

Lane 13

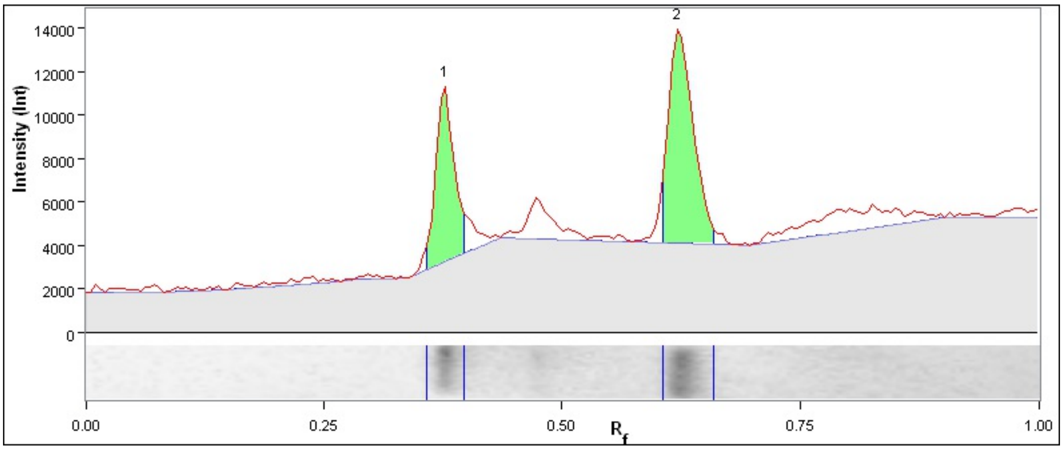

Band No.	Band Label	Mol. Wt. (KDa)	Relative Front	Volume (Int)	Abs. Quant.	Rel. Quant.	Band %	Lane %
1		155,2	0,381	934.450	N/A	N/A	39,2	23,6
2		39,0	0,624	1.450.548	N/A	N/A	60,8	36,7

Lane Background	Lane background subtracted with disk size: 10
Lane Width	4.50 mm
Regression Equation	A single equation is not available for this method

Lane 14

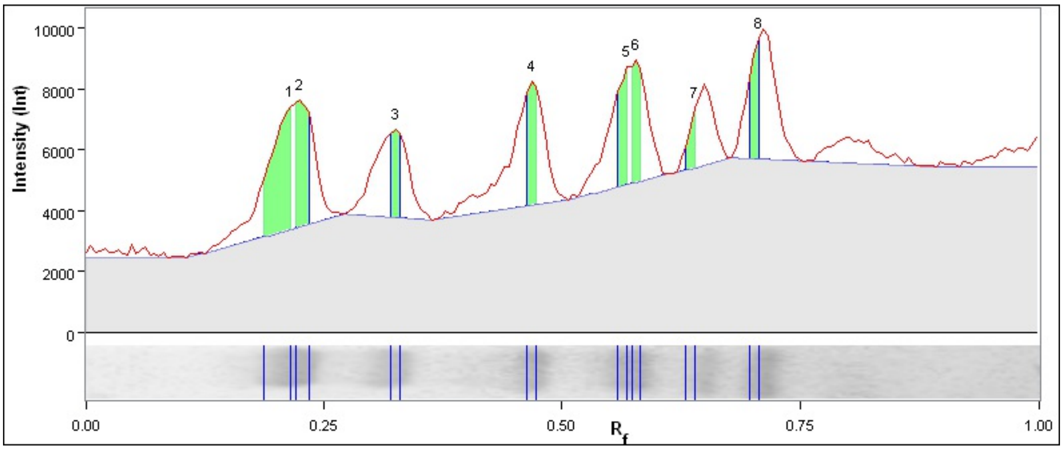

Band No.	Band Label	Mol. Wt. (KDa)	Relative Front	Volume (Int)	Abs. Quant.	Rel. Quant.	Band %	Lane %
1		250,0	0,219	763.918	N/A	N/A	24,6	11,0
2		250,0	0,229	469.075	N/A	N/A	15,1	6,7
3		187,2	0,329	258.564	N/A	N/A	8,3	3,7
4		78,5	0,471	361.688	N/A	N/A	11,7	5,2
5		43,9	0,571	356.120	N/A	N/A	11,5	5,1
6		42,8	0,581	335.646	N/A	N/A	10,8	4,8
7		37,2	0,648	184.411	N/A	N/A	5,9	2,6
8		27,8	0,714	372.737	N/A	N/A	12,0	5,3

Lane Background	Lane background subtracted with disk size: 10
Lane Width	5.94 mm
Regression Equation	A single equation is not available for this method

Lane 15

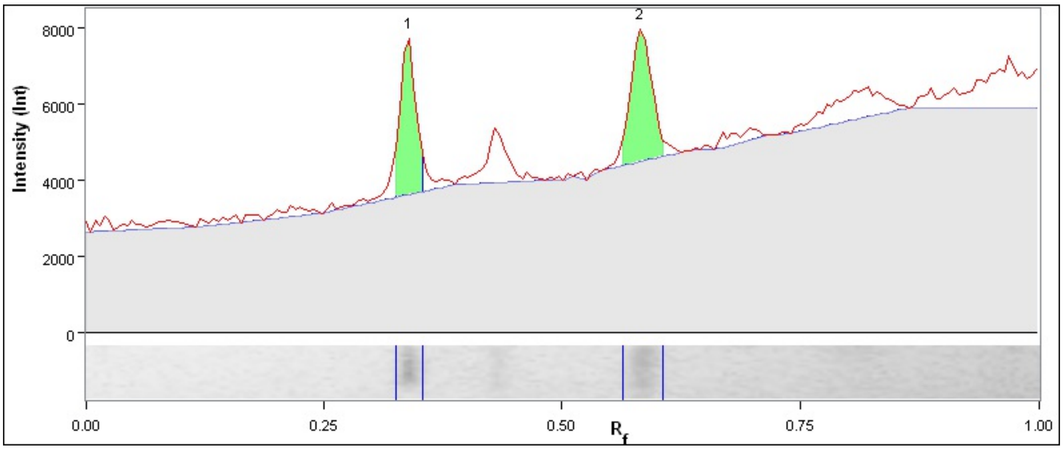

Band No.	Band Label	Mol. Wt. (KDa)	Relative Front	Volume (Int)	Abs. Quant.	Rel. Quant.	Band %	Lane %
1		177,8	0,343	400.972	N/A	N/A	46,4	18,3
2		42,3	0,586	462.836	N/A	N/A	53,6	21,1

Lane Background	Lane background subtracted with disk size: 10
Lane Width	4.50 mm
Regression Equation	A single equation is not available for this method
