# Supplementary material for: Short-chain fructo-oligosaccharides supplementation to suckling piglets: Assessment of pre- and post-weaning performance and gut health
Source: PLoS One. 2020 Jun 5;15(6):e0233910. doi: 10.1371/journal.pone.0233910 (PMC7274435; doi:10.1371/journal.pone.0233910)
Supplement: S9 Data — (PDF) [file pone.0233910.s011.pdf]

Image Report: PCNA_CASP3-12_LADDER+PCNA_CASP3-12

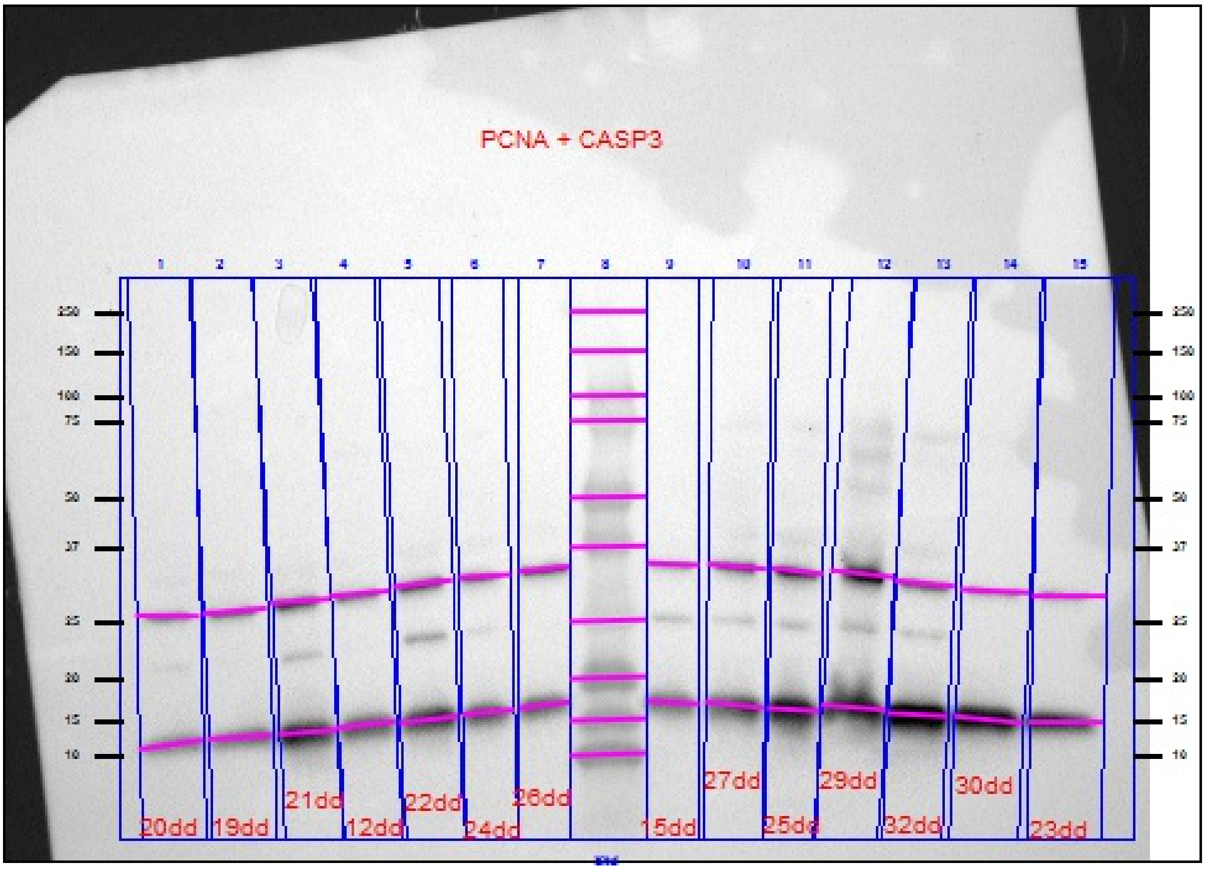

Acquisition Information

Imager	Merged Image

Image Information

Acquisition Date	27/04/2017 9:47:03
User Name	Bio-Rad
Image Area (mm)	X: 95.0 Y: 71.0
Pixel Size (um)	X: 204.7 Y: 205.1
Data Range (Int)	79 - 38575

Notes

Merged images:
Image 1: PCNA_CASP3-12_LADDER
Image 2: PCNA_CASP3-12

Analysis Settings

Detection	Lane detection:<br>Manually created lanes<br><br>Band detection:

	Manually adjusted bands<br><br>Lane Background Subtraction:<br>Lane background subtracted with disk size: 10<br><br>Lane width: Variable
Mol. Weight Analysis	Standard: Bio-Rad Precision Plus<br>Standard lanes: 8<br>Regression method: Point to Point (semi-log)

Lane And Band Analysis

Lane 1

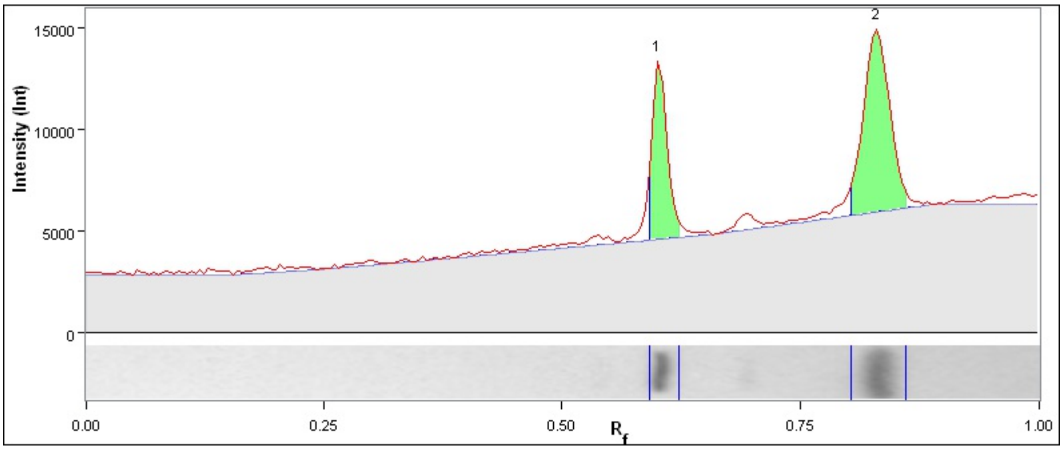

Band No.	Band Label	Mol. Wt. (KDa)	Relative Front	Volume (Int)	Abs. Quant.	Rel. Quant.	Band %	Lane %
1		25,7	0,604	997.490	N/A	N/A	33,6	23,1
2		11,2	0,833	1.972.620	N/A	N/A	66,4	45,6

Lane Background	Lane background subtracted with disk size: 10
Lane Width	5.32 mm
Regression Equation	A single equation is not available for this method

Lane 2

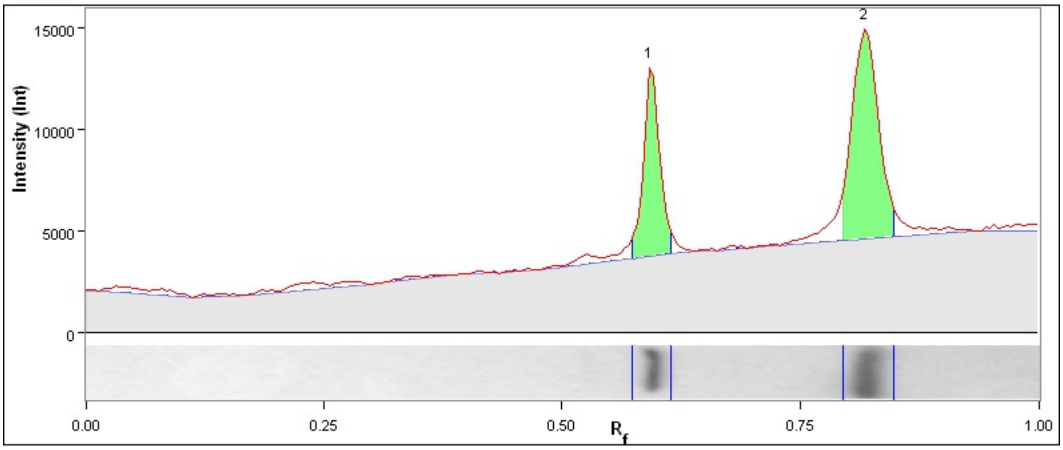

Band No.	Band Label	Mol. Wt. (KDa)	Relative Front	Volume (Int)	Abs. Quant.	Rel. Quant.	Band %	Lane %
1		26,3	0,595	1.450.410	N/A	N/A	35,9	26,6
2		12,2	0,819	2.587.806	N/A	N/A	64,1	47,5

Lane Background	Lane background subtracted with disk size: 10
Lane Width	5.32 mm
Regression Equation	A single equation is not available for this method

Lane 3

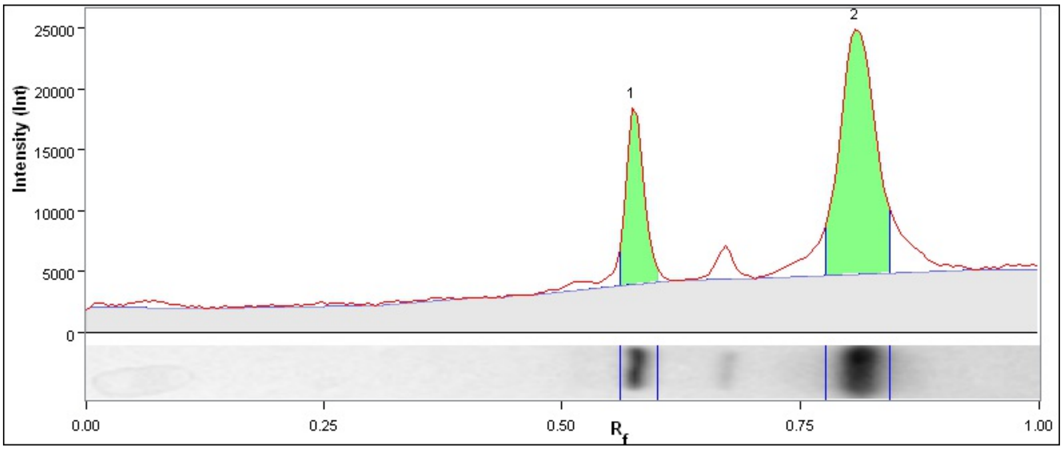

Band No.	Band Label	Mol. Wt. (KDa)	Relative Front	Volume (Int)	Abs. Quant.	Rel. Quant.	Band %	Lane %
1		27,8	0,577	1.941.936	N/A	N/A	25,8	19,0
2		13,0	0,811	5.571.720	N/A	N/A	74,2	54,6

Lane Background	Lane background subtracted with disk size: 10
Lane Width	4.91 mm
Regression Equation	A single equation is not available for this method

Lane 4

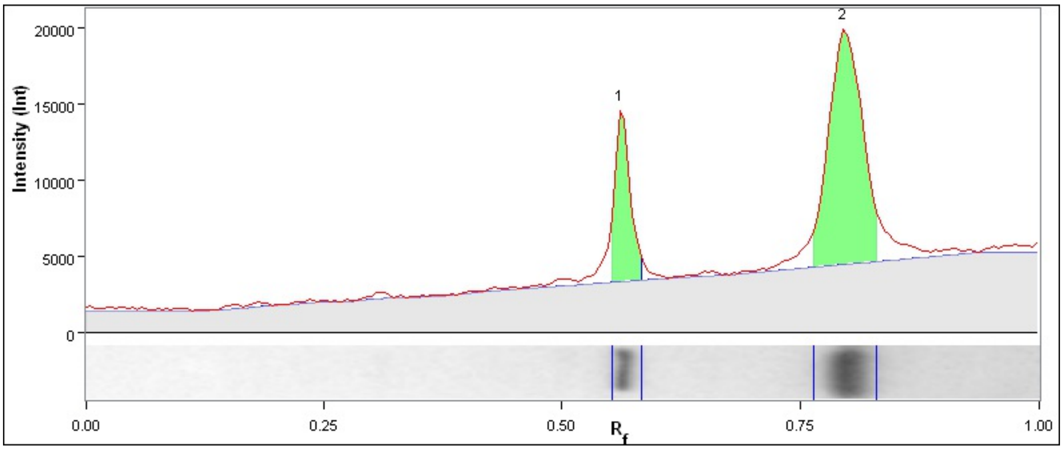

Band No.	Band Label	Mol. Wt. (KDa)	Relative Front	Volume (Int)	Abs. Quant.	Rel. Quant.	Band %	Lane %
1		28,9	0,564	1.240.128	N/A	N/A	24,3	18,4
2		14,2	0,797	3.857.904	N/A	N/A	75,7	57,1

Lane Background	Lane background subtracted with disk size: 10
Lane Width	4.91 mm
Regression Equation	A single equation is not available for this method

Lane 5

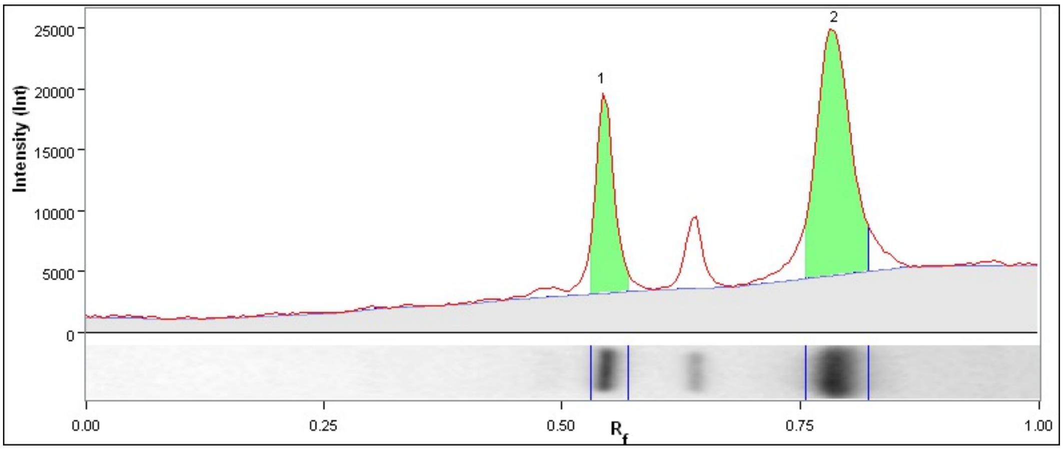

Band No.	Band Label	Mol. Wt. (KDa)	Relative Front	Volume (Int)	Abs. Quant.	Rel. Quant.	Band %	Lane %
1		30,4	0,546	2.015.448	N/A	N/A	29,4	21,8
2		15,0	0,789	4.841.184	N/A	N/A	70,6	52,4

Lane Background	Lane background subtracted with disk size: 10
Lane Width	4.91 mm
Regression Equation	A single equation is not available for this method

Lane 6

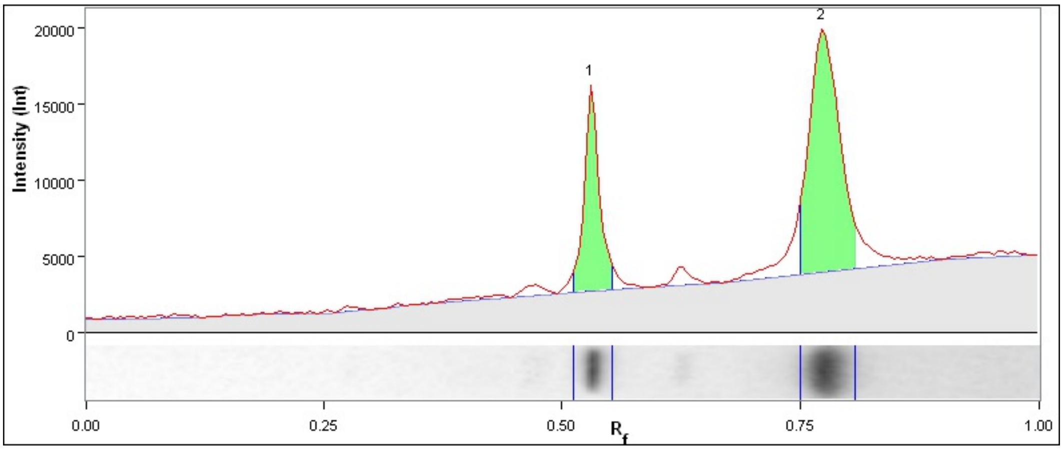

Band No.	Band Label	Mol. Wt. (KDa)	Relative Front	Volume (Int)	Abs. Quant.	Rel. Quant.	Band %	Lane %
1		31,6	0,533	1.354.584	N/A	N/A	30,4	23,1
2		15,8	0,775	3.095.442	N/A	N/A	69,6	52,7

Lane Background	Lane background subtracted with disk size: 10
Lane Width	4.30 mm
Regression Equation	A single equation is not available for this method

Lane 7

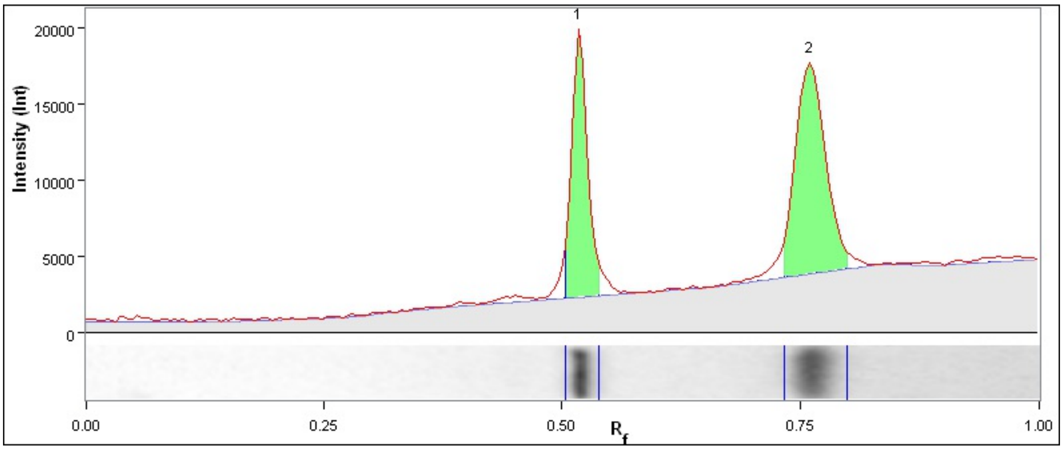

Band No.	Band Label	Mol. Wt. (KDa)	Relative Front	Volume (Int)	Abs. Quant.	Rel. Quant.	Band %	Lane %
1		32,9	0,520	1.867.677	N/A	N/A	39,4	32,3
2		16,6	0,762	2.875.047	N/A	N/A	60,6	49,7

Lane Background	Lane background subtracted with disk size: 10
Lane Width	4.30 mm
Regression Equation	A single equation is not available for this method

Lane 8 - Bio-Rad Precision Plus

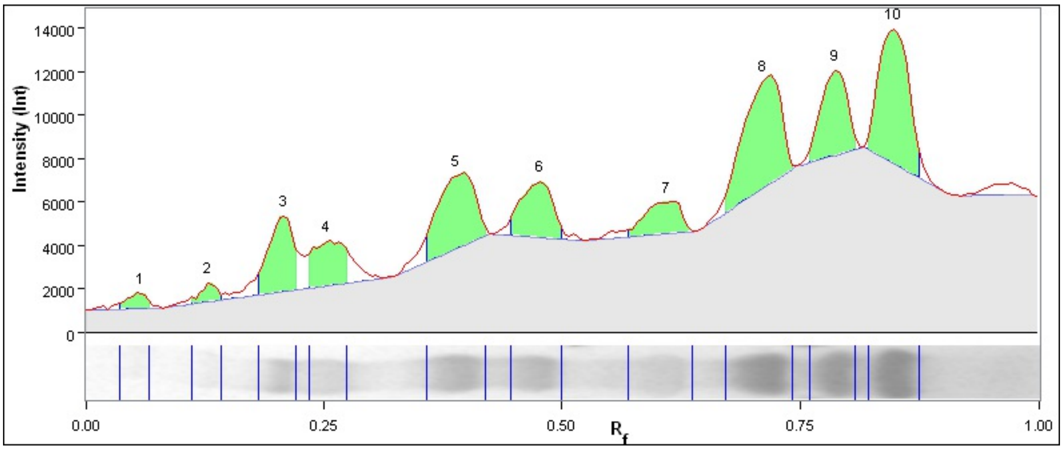

Band No.	Band Label	Mol. Wt. (KDa)	Relative Front	Volume (Int)	Abs. Quant.	Rel. Quant.	Band %	Lane %
1		250,0	0,062	138.074	N/A	N/A	1,6	1,5
2		150,0	0,132	122.264	N/A	N/A	1,4	1,3
3		100,0	0,211	777.139	N/A	N/A	9,1	8,2
4		75,0	0,256	653.046	N/A	N/A	7,7	6,9
5		50,0	0,392	1.143.032	N/A	N/A	13,4	12,1
6		37,0	0,480	763.189	N/A	N/A	9,0	8,1
7		25,0	0,612	495.380	N/A	N/A	5,8	5,2
8		20,0	0,714	1.721.368	N/A	N/A	20,2	18,2
9		15,0	0,789	937.471	N/A	N/A	11,0	9,9
10		10,0	0,850	1.756.925	N/A	N/A	20,7	18,6

Lane Background	Lane background subtracted with disk size: 10
Lane Width	6.35 mm
Regression Equation	A single equation is not available for this method

Lane 9

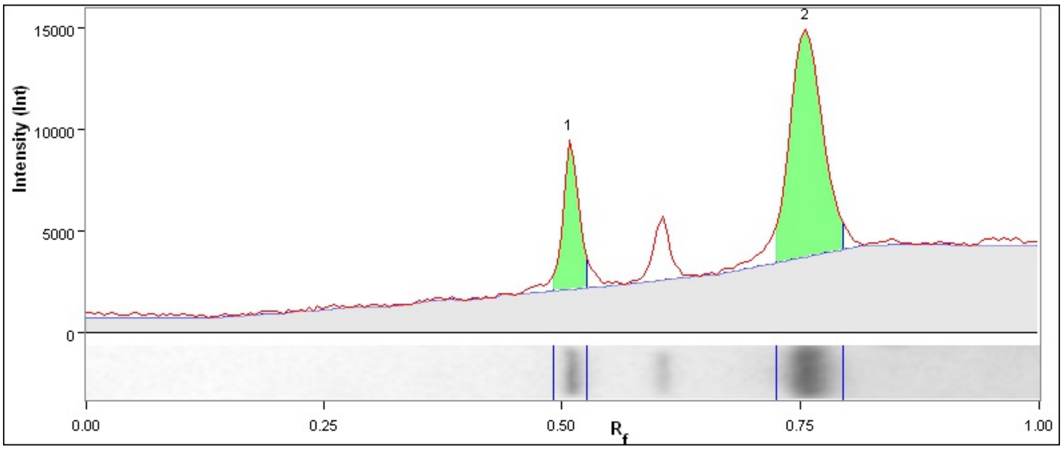

Band No.	Band Label	Mol. Wt. (KDa)	Relative Front	Volume (Int)	Abs. Quant.	Rel. Quant.	Band %	Lane %
1		33,8	0,511	887.649	N/A	N/A	24,0	17,9
2		16,9	0,758	2.807.028	N/A	N/A	76,0	56,6

Lane Background	Lane background subtracted with disk size: 10
Lane Width	4.30 mm
Regression Equation	A single equation is not available for this method

Lane 10

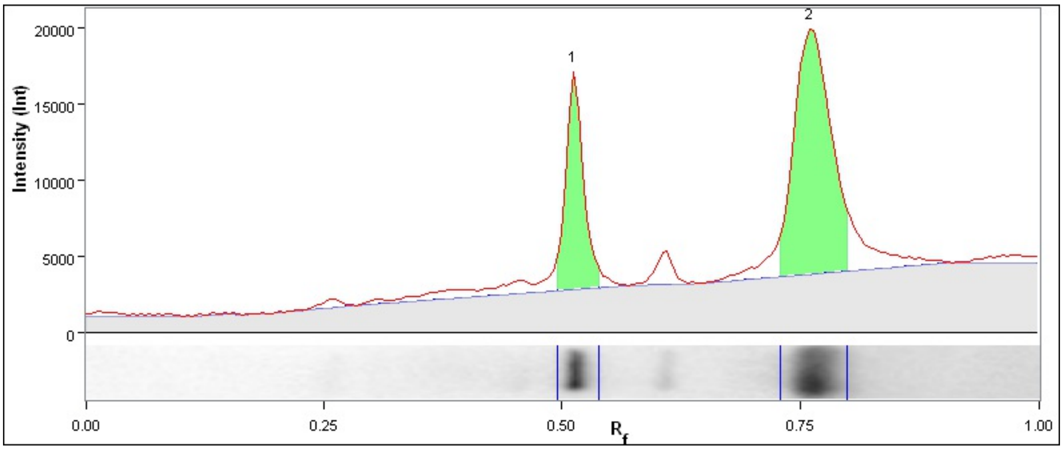

Band No.	Band Label	Mol. Wt. (KDa)	Relative Front	Volume (Int)	Abs. Quant.	Rel. Quant.	Band %	Lane %
1		33,3	0,515	1.957.992	N/A	N/A	29,1	21,3
2		16,6	0,762	4.775.616	N/A	N/A	70,9	52,0

Lane Background	Lane background subtracted with disk size: 10
Lane Width	4.91 mm
Regression Equation	A single equation is not available for this method

Lane 11

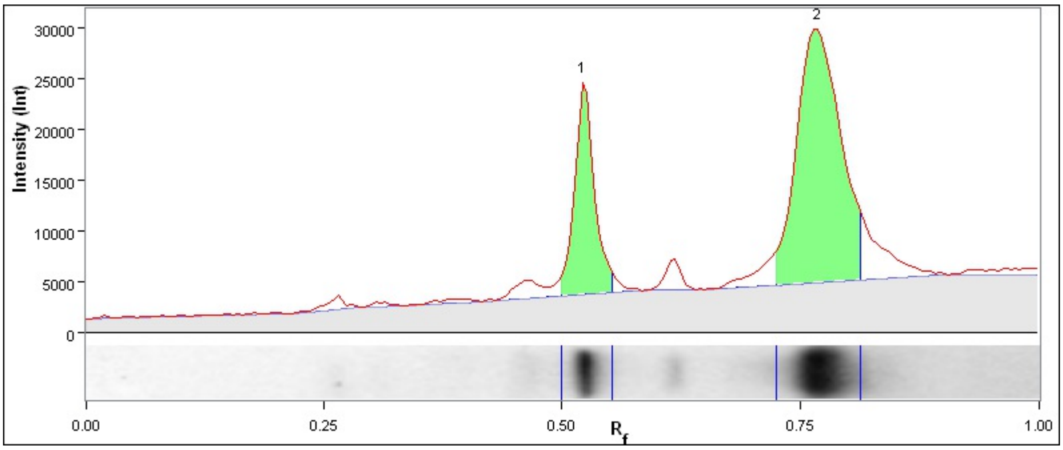

Band No.	Band Label	Mol. Wt. (KDa)	Relative Front	Volume (Int)	Abs. Quant.	Rel. Quant.	Band %	Lane %
1		32,5	0,524	2.512.083	N/A	N/A	27,0	21,3
2		16,1	0,771	6.789.867	N/A	N/A	73,0	57,7

Lane Background	Lane background subtracted with disk size: 10
Lane Width	4.30 mm
Regression Equation	A single equation is not available for this method

Lane 12

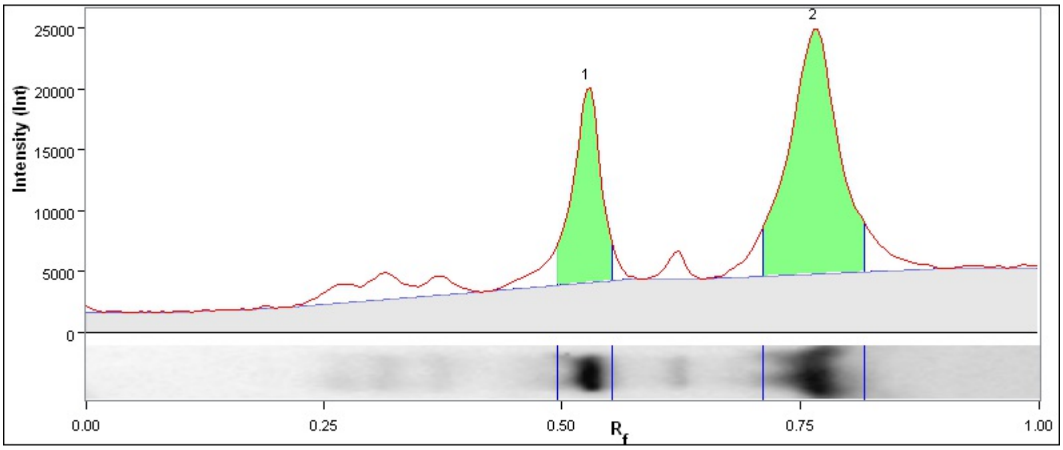

Band No.	Band Label	Mol. Wt. (KDa)	Relative Front	Volume (Int)	Abs. Quant.	Rel. Quant.	Band %	Lane %
1		32,0	0,529	3.859.778	N/A	N/A	30,9	23,5
2		16,3	0,767	8.639.774	N/A	N/A	69,1	52,6

Lane Background	Lane background subtracted with disk size: 10
Lane Width	5.32 mm
Regression Equation	A single equation is not available for this method

Lane 13

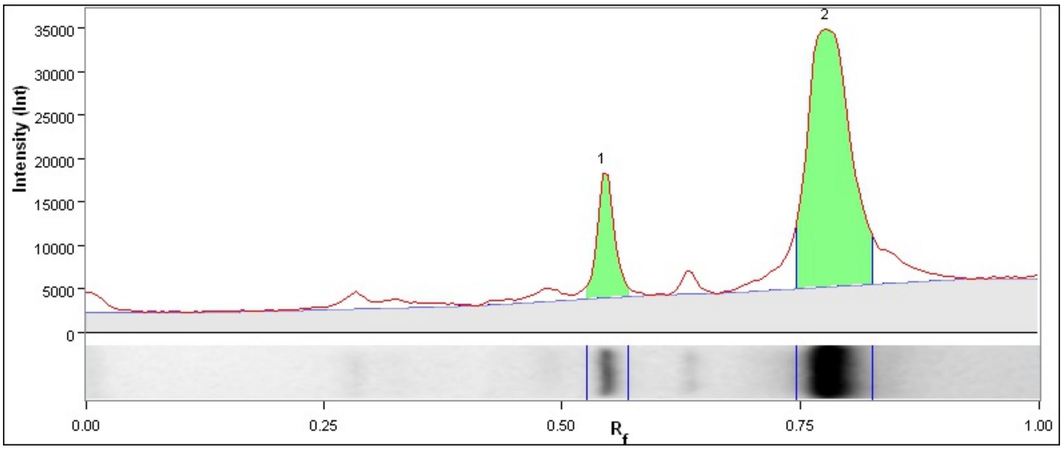

Band No.	Band Label	Mol. Wt. (KDa)	Relative Front	Volume (Int)	Abs. Quant.	Rel. Quant.	Band %	Lane %
1		30,4	0,546	1.746.576	N/A	N/A	16,1	11,9
2		15,5	0,780	9.107.616	N/A	N/A	83,9	61,8

Lane Background	Lane background subtracted with disk size: 10
Lane Width	4.91 mm
Regression Equation	A single equation is not available for this method

Lane 14

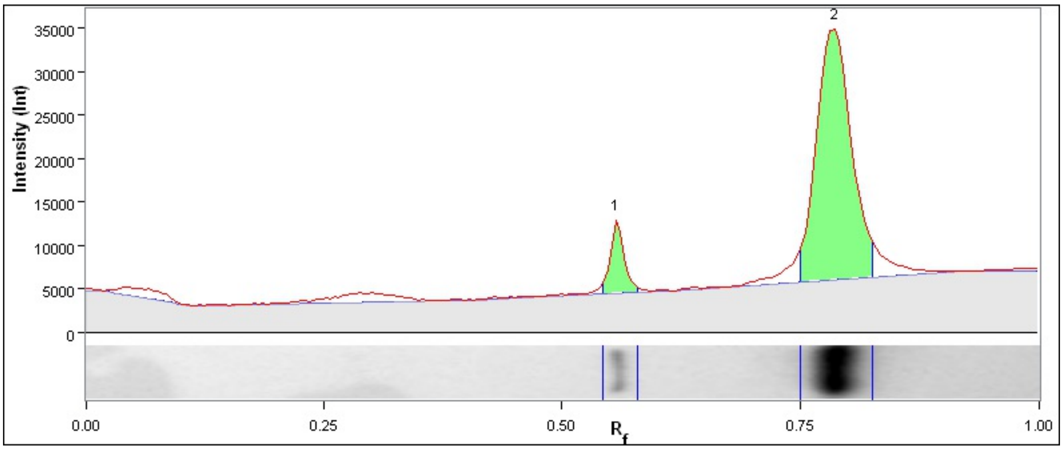

Band No.	Band Label	Mol. Wt. (KDa)	Relative Front	Volume (Int)	Abs. Quant.	Rel. Quant.	Band %	Lane %
1		29,2	0,559	914.312	N/A	N/A	10,3	8,1
2		15,0	0,789	7.979.356	N/A	N/A	89,7	70,8

Lane Background	Lane background subtracted with disk size: 10
Lane Width	5.73 mm
Regression Equation	A single equation is not available for this method

Lane 15

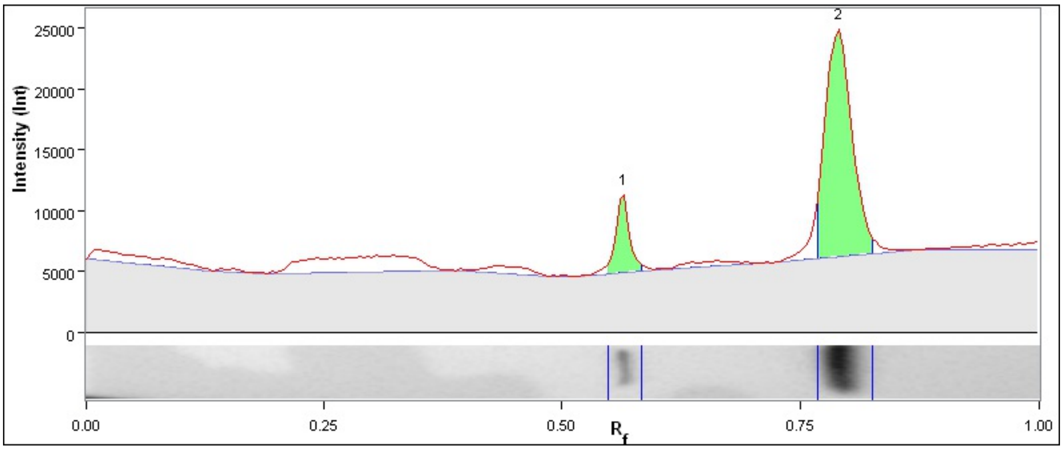

Band No.	Band Label	Mol. Wt. (KDa)	Relative Front	Volume (Int)	Abs. Quant.	Rel. Quant.	Band %	Lane %
1		28,5	0,568	815.850	N/A	N/A	15,5	9,6
2		14,6	0,793	4.454.820	N/A	N/A	84,5	52,2

Lane Background	Lane background subtracted with disk size: 10
Lane Width	6.14 mm
Regression Equation	A single equation is not available for this method
