# Supplementary material for: Short-chain fructo-oligosaccharides supplementation to suckling piglets: Assessment of pre- and post-weaning performance and gut health
Source: PLoS One. 2020 Jun 5;15(6):e0233910. doi: 10.1371/journal.pone.0233910 (PMC7274435; doi:10.1371/journal.pone.0233910)
Supplement: S10 Data — (PDF) [file pone.0233910.s012.pdf]

Image Report: PCNA_CASP3-13_LADDERanalyse9-10-1-14-4-5-11-13

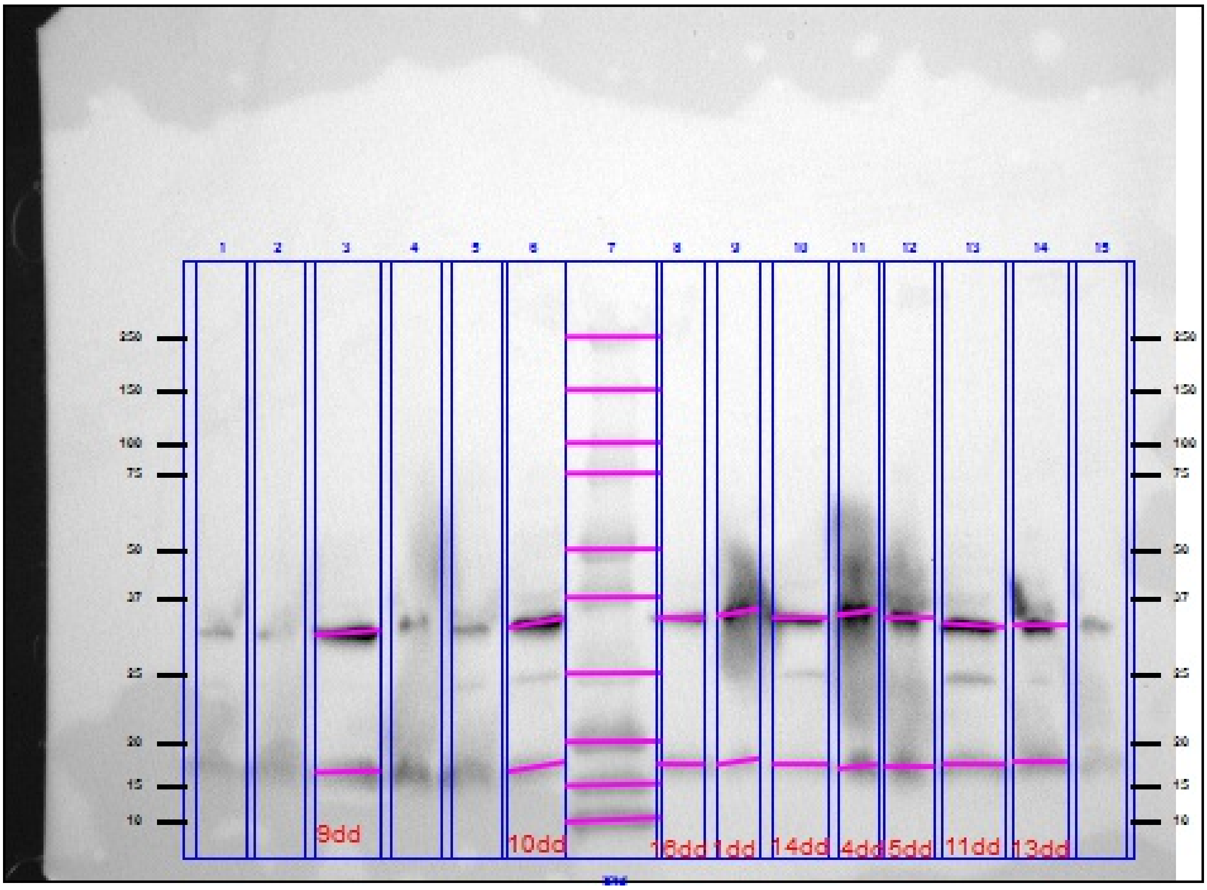

C:\Users\Bio-Rad\Desktop\Katty_Tereos\2N\PCNA_CASP3-13_LADDERanalyse9-10-1-14-4-5-11-13.scn

Acquisition Information

Imager	Merged Image

Image Information

Acquisition Date	26/04/2017 15:02:10
User Name	Bio-Rad
Image Area (mm)	X: 95.0 Y: 71.0
Pixel Size (um)	X: 204.7 Y: 205.1
Data Range (Int)	125 - 37254

Notes

Merged images:
Image 1: PCNA_CASP3-13_LADDER
Image 2: PCNA_CASP3-5_analyse9-10-1-14-4-5-11-13

Analysis Settings

Detection	Lane detection:<br>Manually created lanes<br><br>Band detection:

	Manually adjusted bands<br><br>Lane Background Subtraction:<br>Lane background subtracted with disk size: 10<br><br>Lane width: Variable
Mol. Weight Analysis	Standard: Bio-Rad Precision Plus<br>Standard lanes: 7<br>Regression method: Point to Point (semi-log)

Lane And Band Analysis

Lane 1

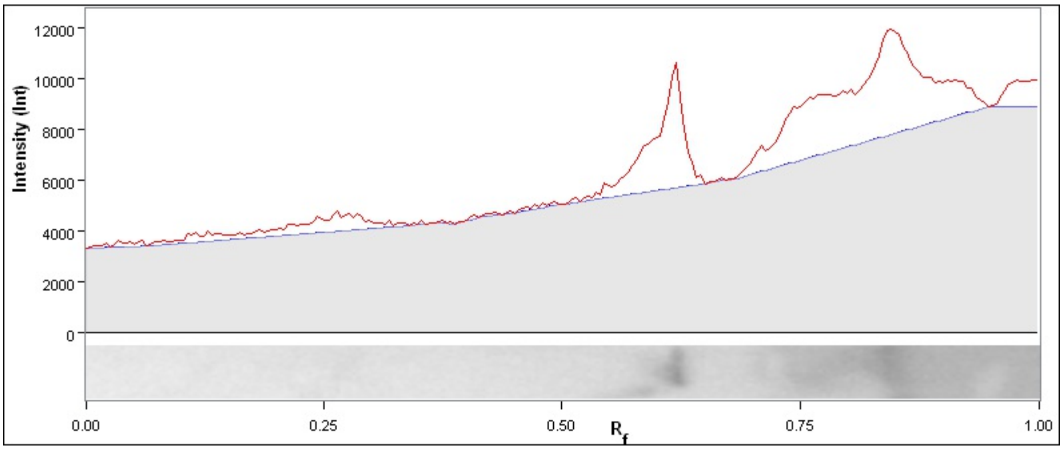

Band No.	Band Label	Mol. Wt. (KDa)	Relative Front	Volume (Int)	Abs. Quant.	Rel. Quant.	Band %	Lane %

Lane Background	Lane background subtracted with disk size: 10
Lane Width	4.09 mm
Regression Equation	A single equation is not available for this method

Lane 2

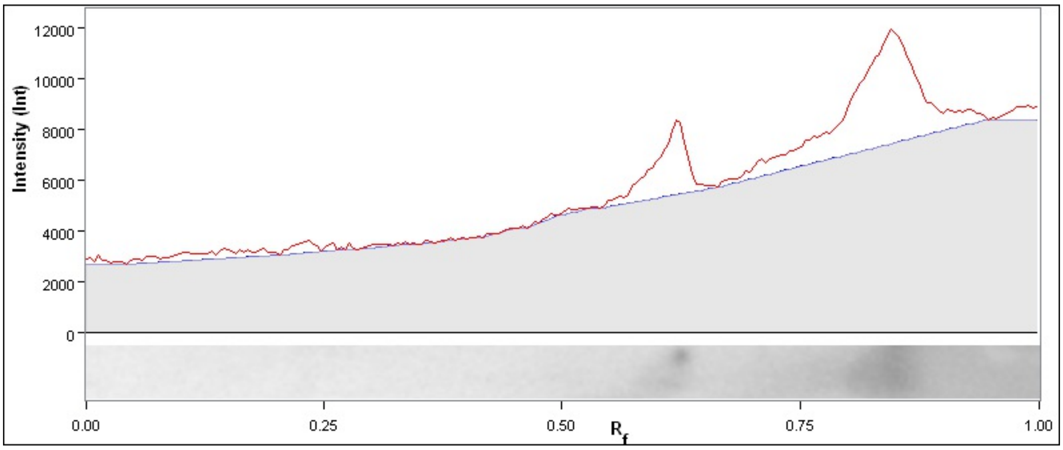

Band No.	Band Label	Mol. Wt. (KDa)	Relative Front	Volume (Int)	Abs. Quant.	Rel. Quant.	Band %	Lane %

Lane Background	Lane background subtracted with disk size: 10
Lane Width	4.09 mm
Regression Equation	A single equation is not available for this method

Lane 3

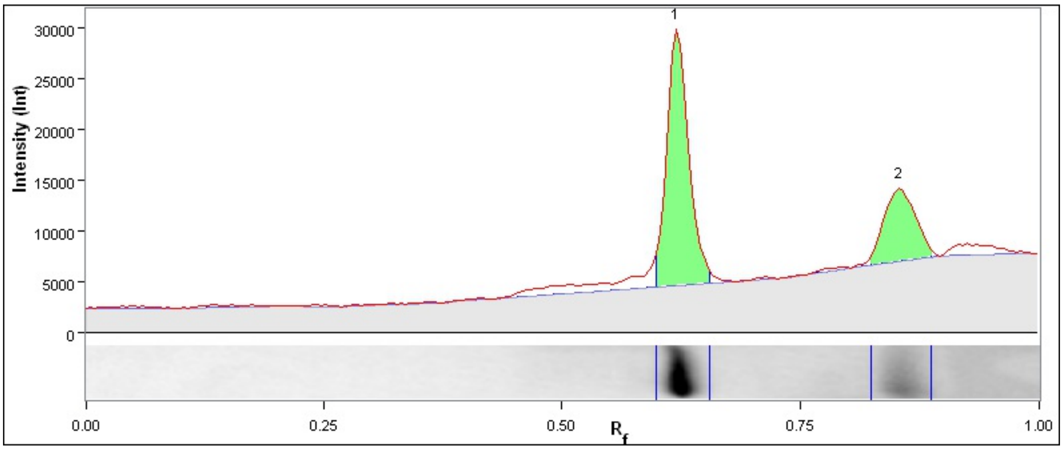

Band No.	Band Label	Mol. Wt. (KDa)	Relative Front	Volume (Int)	Abs. Quant.	Rel. Quant.	Band %	Lane %
1		30,8	0,623	4.354.766	N/A	N/A	70,7	55,1
2		16,3	0,856	1.800.786	N/A	N/A	29,3	22,8

Lane Background	Lane background subtracted with disk size: 10
Lane Width	5.32 mm
Regression Equation	A single equation is not available for this method

Lane 4

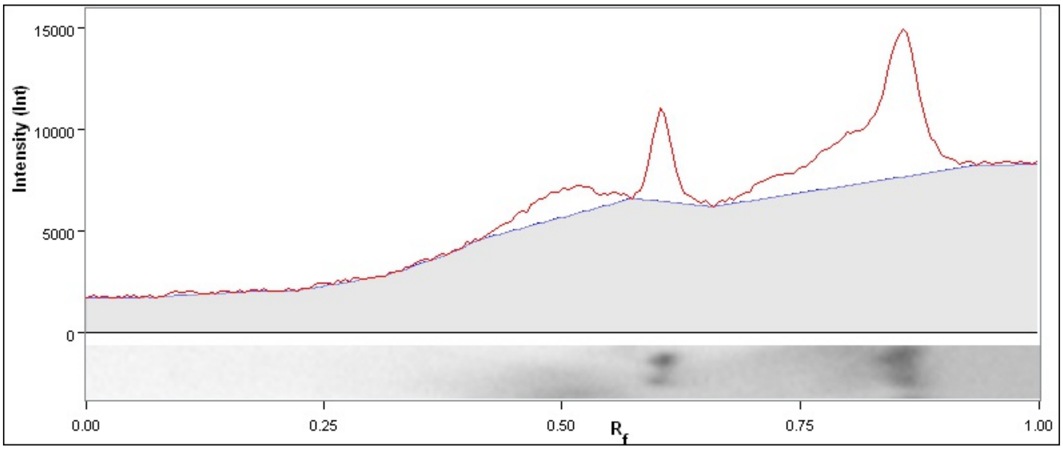

Band No.	Band Label	Mol. Wt. (KDa)	Relative Front	Volume (Int)	Abs. Quant.	Rel. Quant.	Band %	Lane %

Lane Background	Lane background subtracted with disk size: 10
Lane Width	4.09 mm
Regression Equation	A single equation is not available for this method

Lane 5

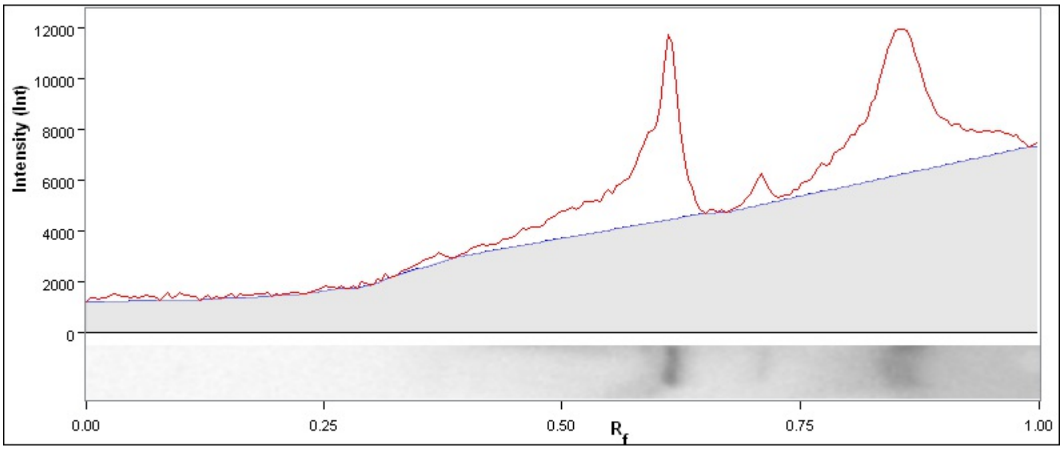

Band No.	Band Label	Mol. Wt. (KDa)	Relative Front	Volume (Int)	Abs. Quant.	Rel. Quant.	Band %	Lane %

Lane Background	Lane background subtracted with disk size: 10
Lane Width	4.09 mm
Regression Equation	A single equation is not available for this method

Lane 6

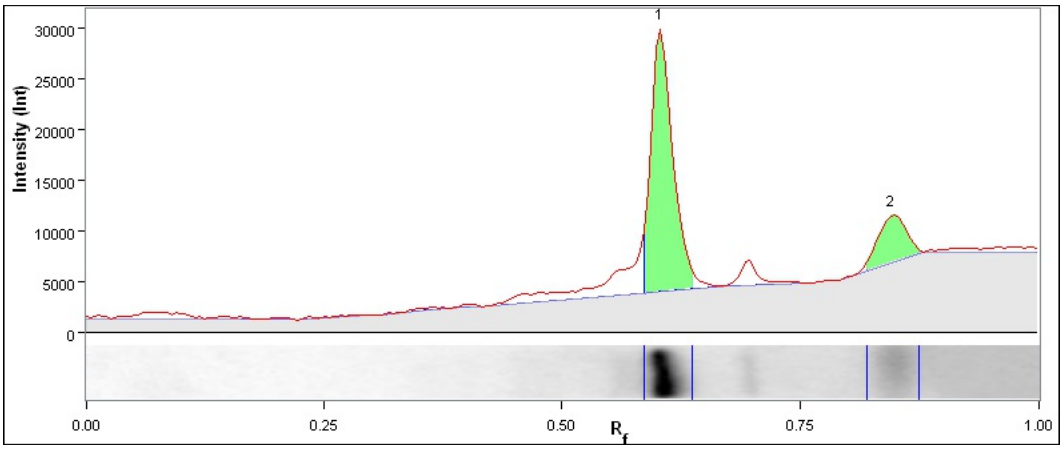

Band No.	Band Label	Mol. Wt. (KDa)	Relative Front	Volume (Int)	Abs. Quant.	Rel. Quant.	Band %	Lane %
1		32,5	0,606	3.824.118	N/A	N/A	81,2	55,0
2		16,9	0,847	888.007	N/A	N/A	18,8	12,8

Lane Background	Lane background subtracted with disk size: 10
Lane Width	4.71 mm
Regression Equation	A single equation is not available for this method

Lane 7 - Bio-Rad Precision Plus

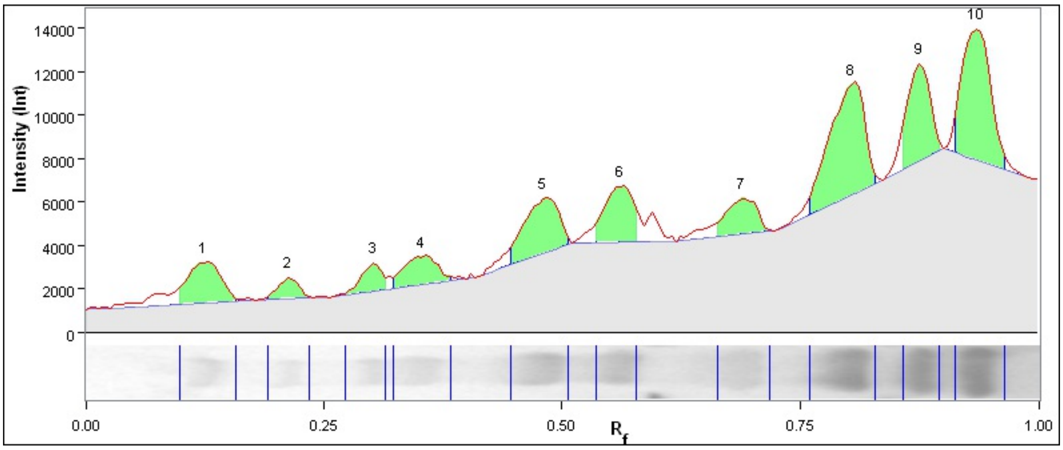

Band No.	Band Label	Mol. Wt. (KDa)	Relative Front	Volume (Int)	Abs. Quant.	Rel. Quant.	Band %	Lane %
1		250,0	0,127	696.274	N/A	N/A	7,7	6,7
2		150,0	0,216	215.878	N/A	N/A	2,4	2,1
3		100,0	0,305	307.040	N/A	N/A	3,4	3,0
4		75,0	0,356	507.300	N/A	N/A	5,6	4,9
5		50,0	0,483	1.019.958	N/A	N/A	11,2	9,8
6		37,0	0,564	852.568	N/A	N/A	9,4	8,2
7		25,0	0,691	550.772	N/A	N/A	6,1	5,3
8		20,0	0,805	2.011.150	N/A	N/A	22,2	19,4
9		15,0	0,877	1.064.304	N/A	N/A	11,7	10,2
10		10,0	0,936	1.846.496	N/A	N/A	20,4	17,8

Lane Background	Lane background subtracted with disk size: 10
Lane Width	7.78 mm
Regression Equation	A single equation is not available for this method

Lane 8

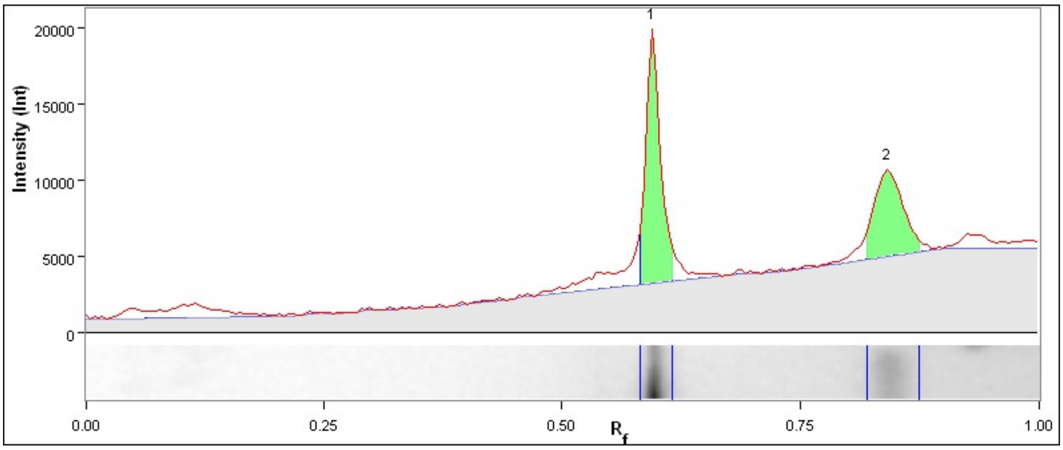

Band No.	Band Label	Mol. Wt. (KDa)	Relative Front	Volume (Int)	Abs. Quant.	Rel. Quant.	Band %	Lane %
1		33,3	0,597	1.370.755	N/A	N/A	60,4	37,2
2		17,2	0,843	900.505	N/A	N/A	39,6	24,5

Lane Background	Lane background subtracted with disk size: 10
Lane Width	3.89 mm
Regression Equation	A single equation is not available for this method

Lane 9

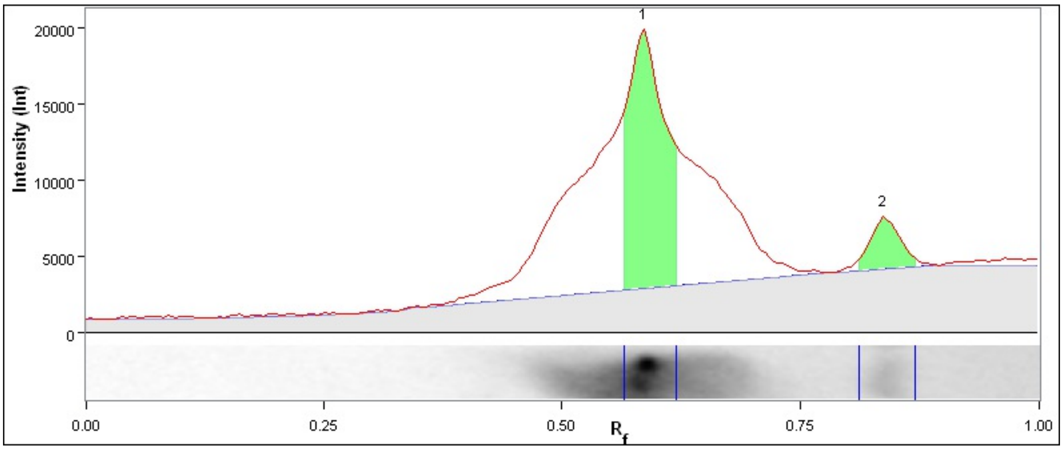

Band No.	Band Label	Mol. Wt. (KDa)	Relative Front	Volume (Int)	Abs. Quant.	Rel. Quant.	Band %	Lane %
1		34,2	0,589	3.793.057	N/A	N/A	86,1	33,0
2		17,5	0,839	611.439	N/A	N/A	13,9	5,3

Lane Background	Lane background subtracted with disk size: 10
Lane Width	3.48 mm
Regression Equation	A single equation is not available for this method

Lane 10

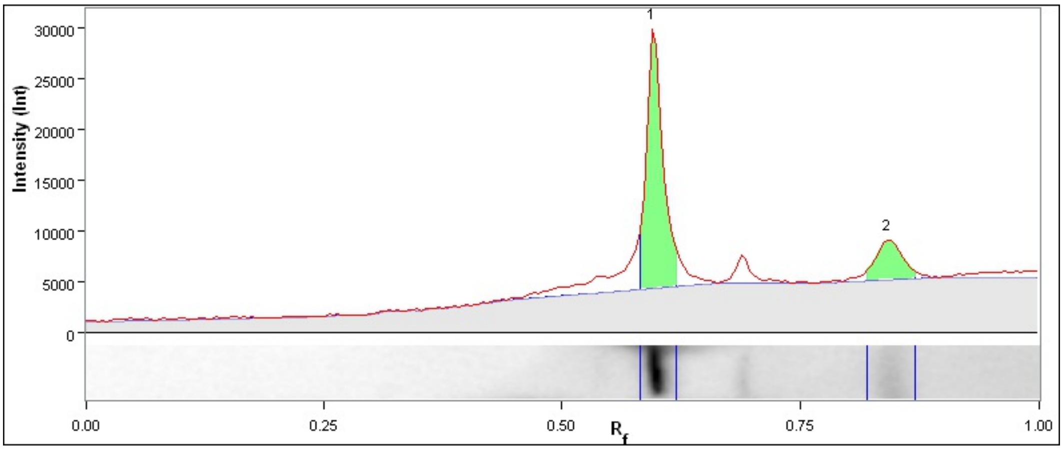

Band No.	Band Label	Mol. Wt. (KDa)	Relative Front	Volume (Int)	Abs. Quant.	Rel. Quant.	Band %	Lane %
1		33,3	0,597	2.752.112	N/A	N/A	80,4	50,9
2		17,2	0,843	672.474	N/A	N/A	19,6	12,4

Lane Background	Lane background subtracted with disk size: 10
Lane Width	4.50 mm
Regression Equation	A single equation is not available for this method

Lane 11

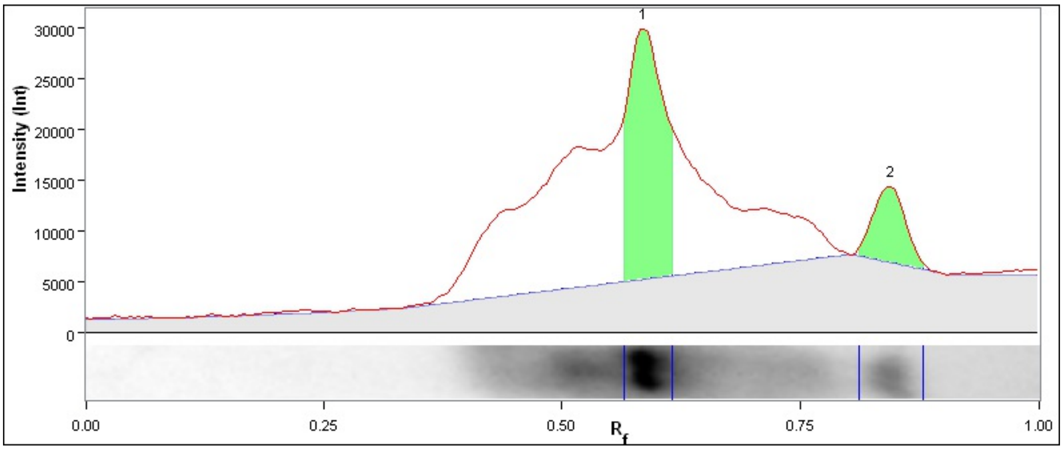

Band No.	Band Label	Mol. Wt. (KDa)	Relative Front	Volume (Int)	Abs. Quant.	Rel. Quant.	Band %	Lane %
1		34,2	0,589	4.268.640	N/A	N/A	78,7	25,6
2		16,9	0,847	1.158.496	N/A	N/A	21,3	6,9

Lane Background	Lane background subtracted with disk size: 10
Lane Width	3.28 mm
Regression Equation	A single equation is not available for this method

Lane 12

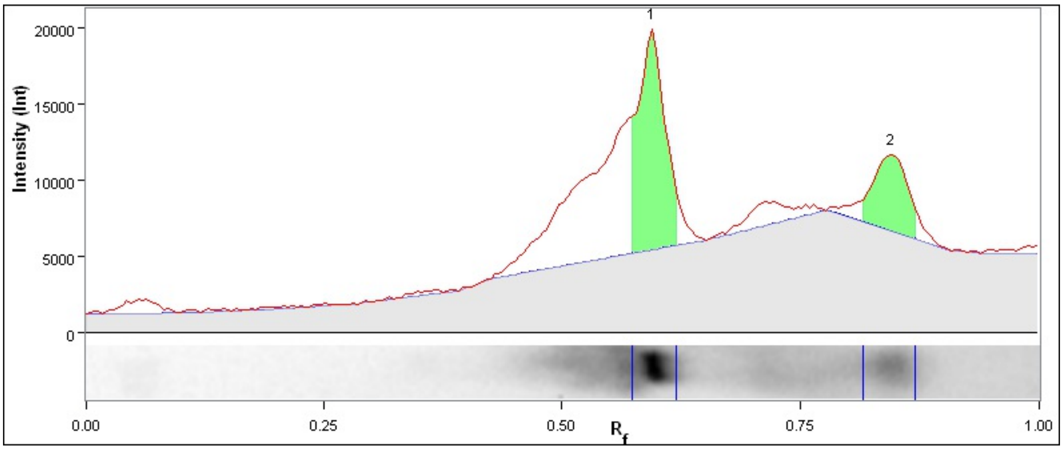

Band No.	Band Label	Mol. Wt. (KDa)	Relative Front	Volume (Int)	Abs. Quant.	Rel. Quant.	Band %	Lane %
1		33,3	0,597	2.584.980	N/A	N/A	68,7	30,5
2		16,9	0,847	1.180.000	N/A	N/A	31,3	13,9

Lane Background	Lane background subtracted with disk size: 10
Lane Width	4.09 mm
Regression Equation	A single equation is not available for this method

Lane 13

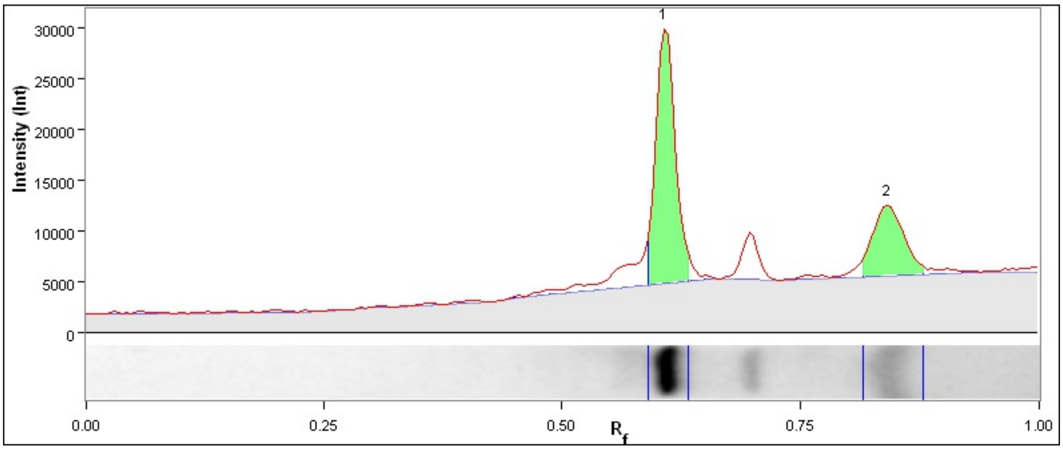

Band No.	Band Label	Mol. Wt. (KDa)	Relative Front	Volume (Int)	Abs. Quant.	Rel. Quant.	Band %	Lane %
1		32,0	0,610	3.804.875	N/A	N/A	69,5	48,7
2		17,2	0,843	1.669.275	N/A	N/A	30,5	21,4

Lane Background	Lane background subtracted with disk size: 10
Lane Width	5.12 mm
Regression Equation	A single equation is not available for this method

Lane 14

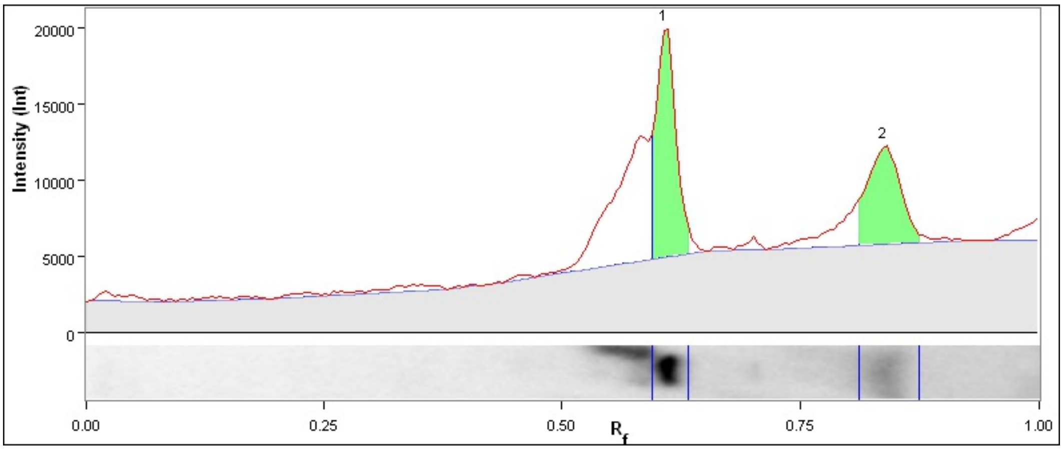

Band No.	Band Label	Mol. Wt. (KDa)	Relative Front	Volume (Int)	Abs. Quant.	Rel. Quant.	Band %	Lane %
1		32,0	0,610	2.049.366	N/A	N/A	57,9	27,8
2		17,5	0,839	1.492.854	N/A	N/A	42,1	20,2

Lane Background	Lane background subtracted with disk size: 10
Lane Width	4.50 mm
Regression Equation	A single equation is not available for this method

Lane 15

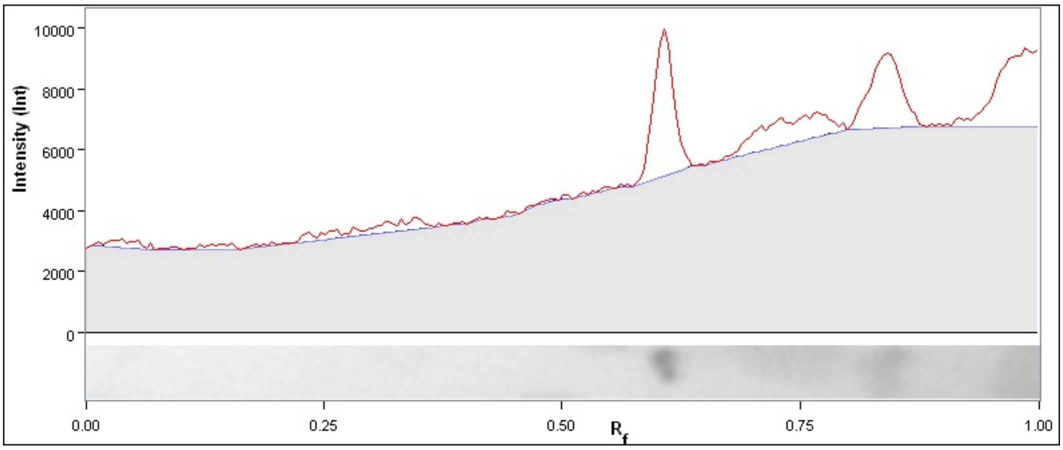

Band No.	Band Label	Mol. Wt. (KDa)	Relative Front	Volume (Int)	Abs. Quant.	Rel. Quant.	Band %	Lane %

Lane Background	Lane background subtracted with disk size: 10
Lane Width	4.09 mm
Regression Equation	A single equation is not available for this method
