# Supplementary material for: Short-chain fructo-oligosaccharides supplementation to suckling piglets: Assessment of pre- and post-weaning performance and gut health
Source: PLoS One. 2020 Jun 5;15(6):e0233910. doi: 10.1371/journal.pone.0233910 (PMC7274435; doi:10.1371/journal.pone.0233910)
Supplement: S11 Data — (PDF) [file pone.0233910.s013.pdf]

Image Report:
PCNA_CASP3-13_LADDER+PCNA_CASP3-12_analyse3-6-16-2dd

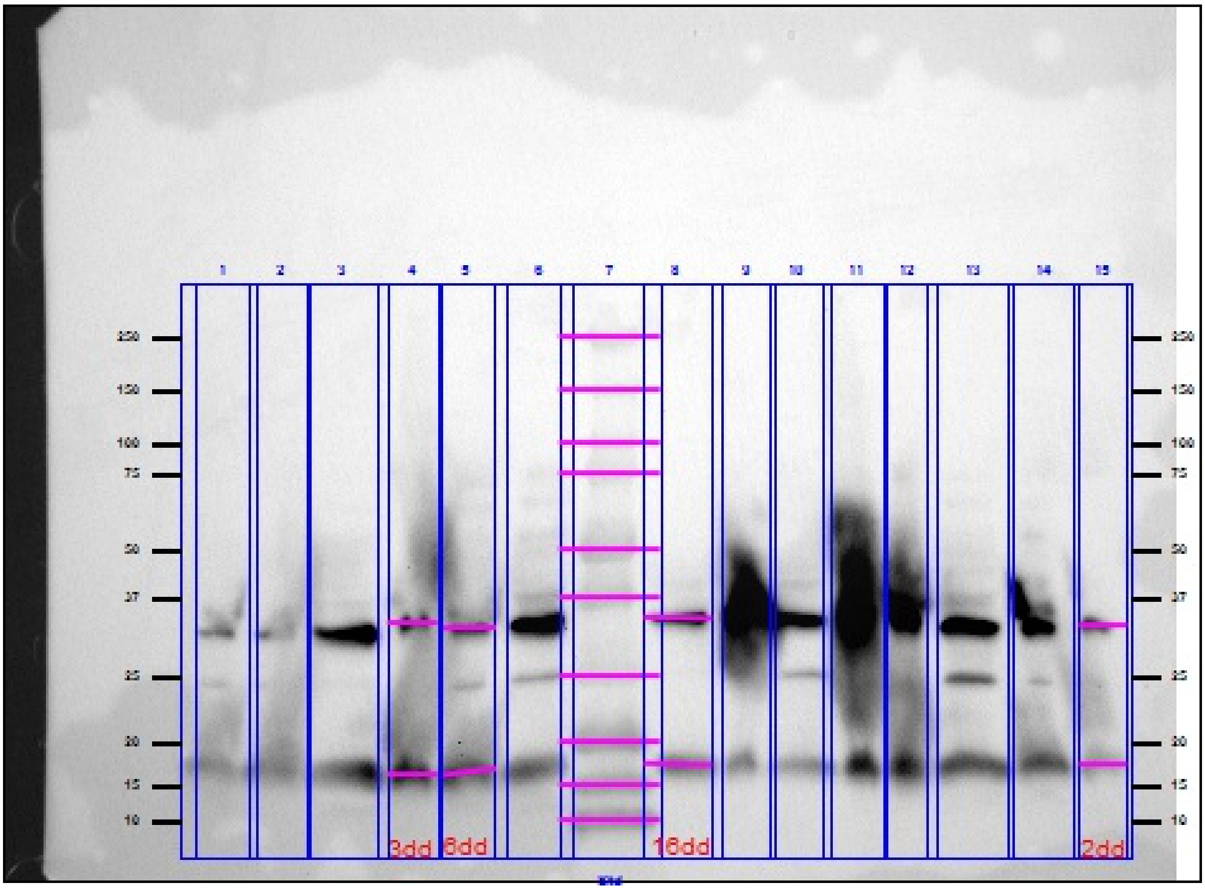

Acquisition Information

Imager	Merged Image

Image Information

Acquisition Date	26/04/2017 15:13:41
User Name	Bio-Rad
Image Area (mm)	X: 95.0 Y: 71.0
Pixel Size (um)	X: 204.7 Y: 205.1
Data Range (Int)	128 - 37275

Notes

Merged images:
Image 1: PCNA_CASP3-13_LADDER
Image 2: PCNA_CASP3-12_analyse3-6-16-2dd

Analysis Settings

Detection	Lane detection:<br>Manually created lanes

	Band detection:<br><br>Manually adjusted bands<br><br>Lane Background Subtraction:<br>Lane background subtracted with disk size: 10<br><br>Lane width: Variable
Mol. Weight Analysis	Standard: Bio-Rad Precision Plus<br>Standard lanes: 7<br>Regression method: Point to Point (semi-log)

Lane And Band Analysis

Lane 1

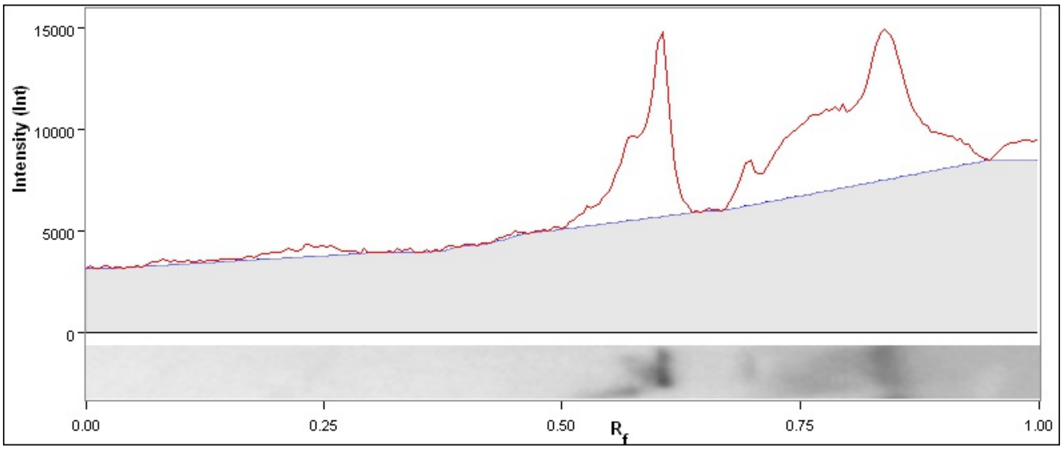

Band No.	Band Label	Mol. Wt. (KDa)	Relative Front	Volume (Int)	Abs. Quant.	Rel. Quant.	Band %	Lane %

Lane Background	Lane background subtracted with disk size: 10
Lane Width	4.30 mm
Regression Equation	A single equation is not available for this method

Lane 2

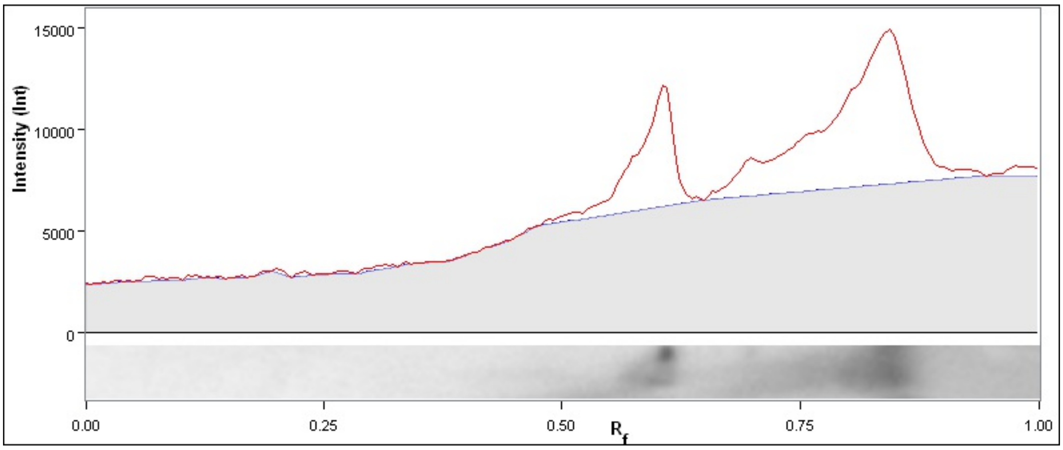

Band No.	Band Label	Mol. Wt. (KDa)	Relative Front	Volume (Int)	Abs. Quant.	Rel. Quant.	Band %	Lane %

Lane Background	Lane background subtracted with disk size: 10
Lane Width	4.30 mm
Regression Equation	A single equation is not available for this method

Lane 3

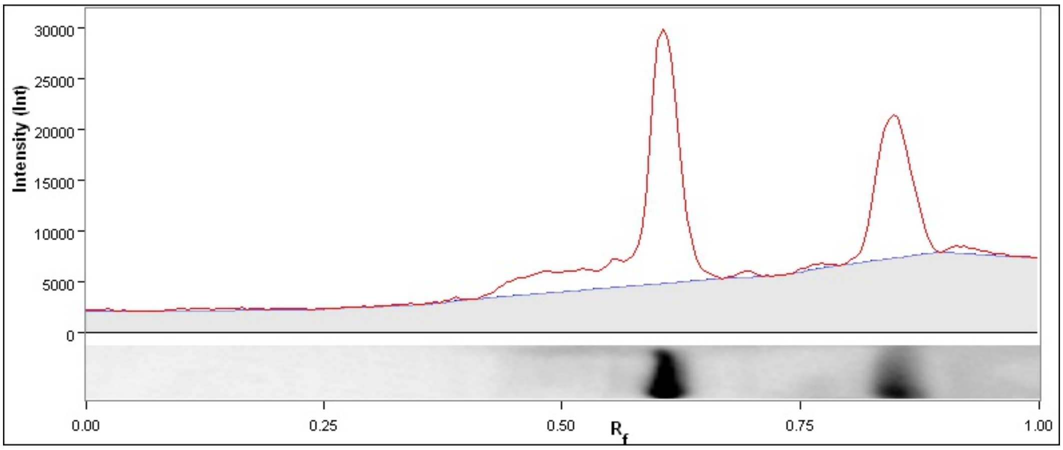

Band No.	Band Label	Mol. Wt. (KDa)	Relative Front	Volume (Int)	Abs. Quant.	Rel. Quant.	Band %	Lane %

Lane Background	Lane background subtracted with disk size: 10
Lane Width	5.73 mm
Regression Equation	A single equation is not available for this method

Lane 4

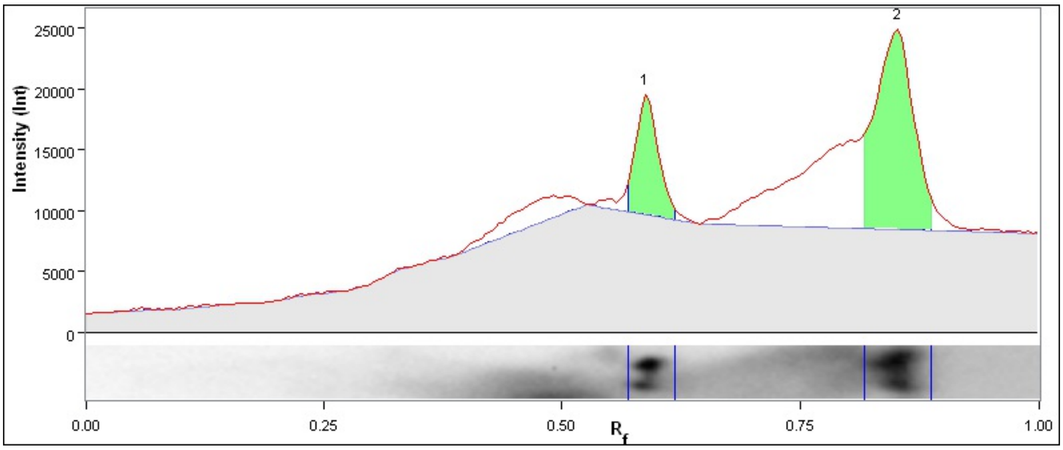

Band No.	Band Label	Mol. Wt. (KDa)	Relative Front	Volume (Int)	Abs. Quant.	Rel. Quant.	Band %	Lane %
1		32,6	0,590	1.361.800	N/A	N/A	26,2	13,9
2		16,1	0,855	3.836.800	N/A	N/A	73,8	39,2

Lane Background	Lane background subtracted with disk size: 10
Lane Width	4.09 mm
Regression Equation	A single equation is not available for this method

Lane 5

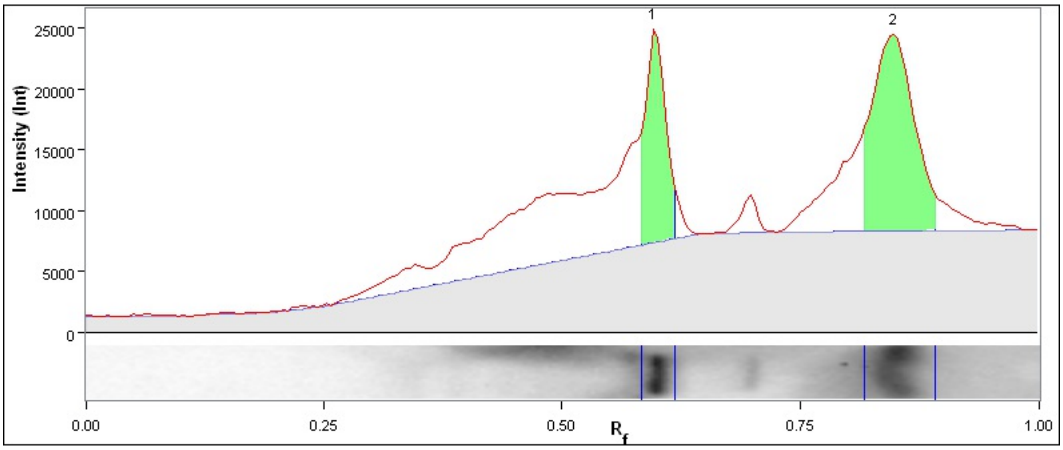

Band No.	Band Label	Mol. Wt. (KDa)	Relative Front	Volume (Int)	Abs. Quant.	Rel. Quant.	Band %	Lane %
1		31,8	0,599	2.007.852	N/A	N/A	34,0	14,4
2		16,3	0,850	3.896.676	N/A	N/A	66,0	28,0

Lane Background	Lane background subtracted with disk size: 10
Lane Width	4.30 mm
Regression Equation	A single equation is not available for this method

Lane 6

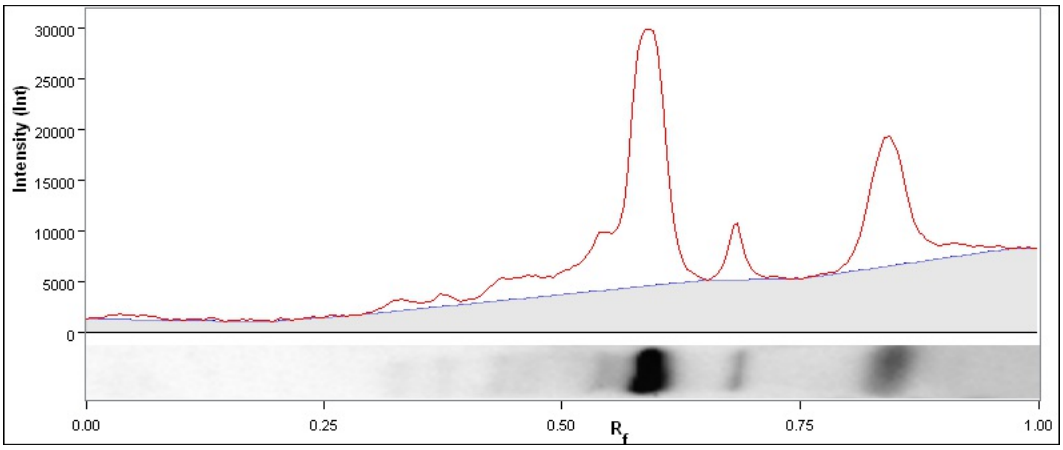

Band No.	Band Label	Mol. Wt. (KDa)	Relative Front	Volume (Int)	Abs. Quant.	Rel. Quant.	Band %	Lane %

Lane Background	Lane background subtracted with disk size: 10
Lane Width	5.32 mm
Regression Equation	A single equation is not available for this method

Lane 7 - Bio-Rad Precision Plus

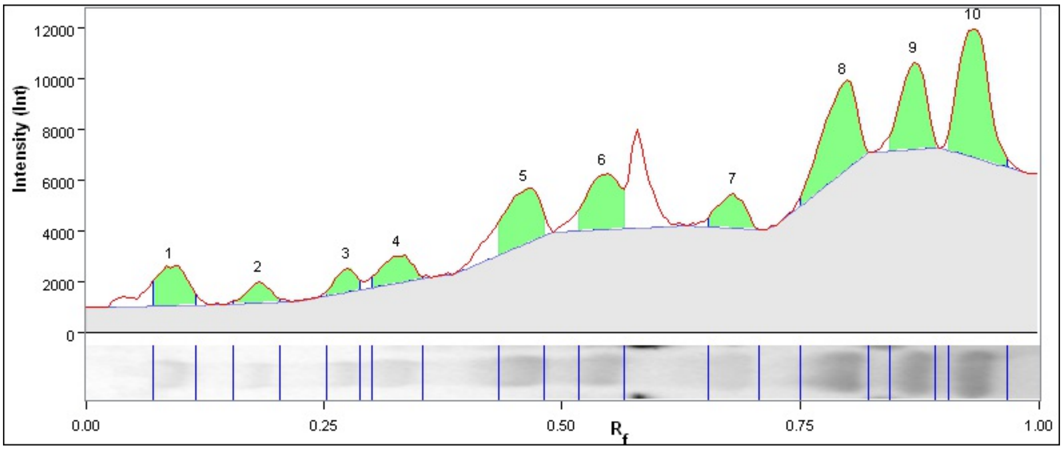

Band No.	Band Label	Mol. Wt. (KDa)	Relative Front	Volume (Int)	Abs. Quant.	Rel. Quant.	Band %	Lane %
1		250,0	0,093	589.640	N/A	N/A	6,8	5,7
2		150,0	0,185	253.680	N/A	N/A	2,9	2,4
3		100,0	0,278	253.960	N/A	N/A	2,9	2,4
4		75,0	0,330	434.360	N/A	N/A	5,0	4,2
5		50,0	0,463	985.720	N/A	N/A	11,4	9,5
6		37,0	0,546	971.160	N/A	N/A	11,3	9,3
7		25,0	0,683	463.840	N/A	N/A	5,4	4,5
8		20,0	0,797	1.569.760	N/A	N/A	18,2	15,1
9		15,0	0,872	1.115.400	N/A	N/A	13,0	10,7
10		10,0	0,934	1.973.040	N/A	N/A	22,9	18,9

Lane Background	Lane background subtracted with disk size: 10
Lane Width	8.19 mm
Regression Equation	A single equation is not available for this method

Lane 8

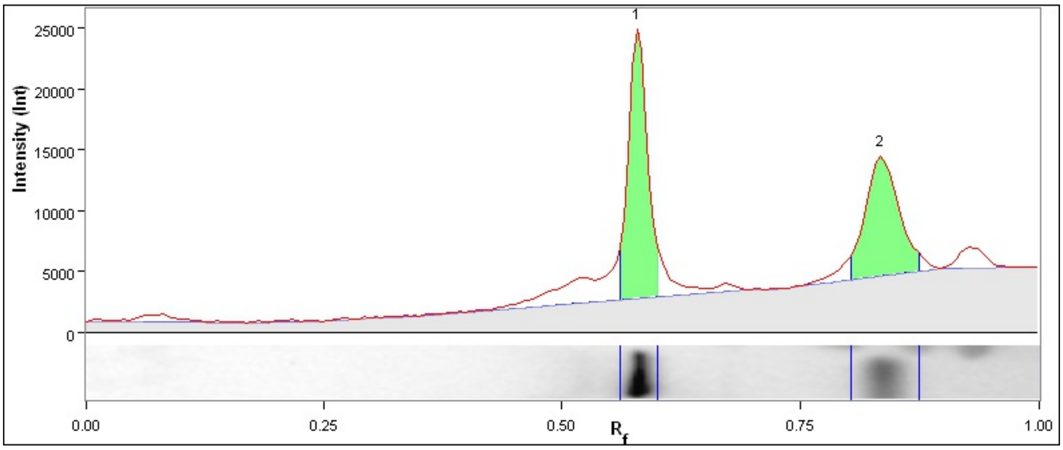

Band No.	Band Label	Mol. Wt. (KDa)	Relative Front	Volume (Int)	Abs. Quant.	Rel. Quant.	Band %	Lane %
1		33,4	0,581	3.643.245	N/A	N/A	55,9	38,7
2		17,2	0,837	2.869.830	N/A	N/A	44,1	30,5

Lane Background	Lane background subtracted with disk size: 10
Lane Width	5.53 mm
Regression Equation	A single equation is not available for this method

Lane 9

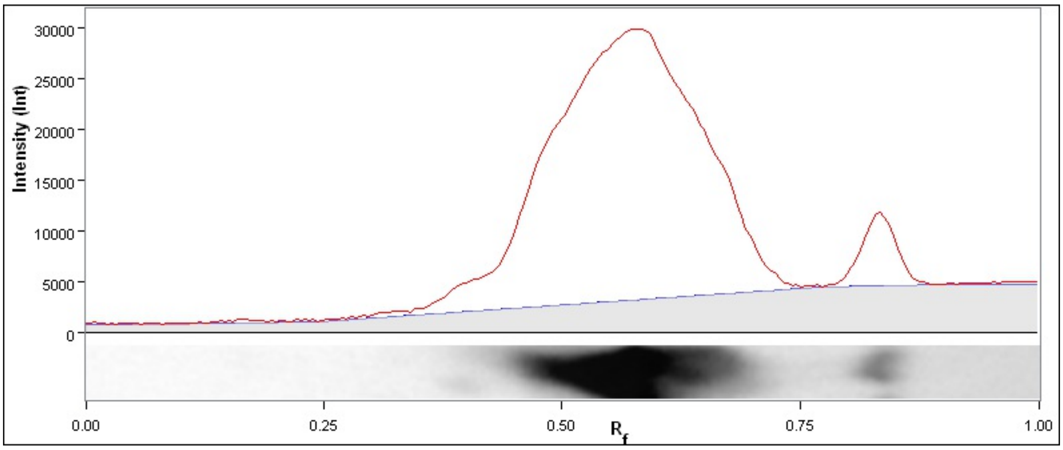

Band No.	Band Label	Mol. Wt. (KDa)	Relative Front	Volume (Int)	Abs. Quant.	Rel. Quant.	Band %	Lane %

Lane Background	Lane background subtracted with disk size: 10
Lane Width	4.30 mm
Regression Equation	A single equation is not available for this method

Lane 10

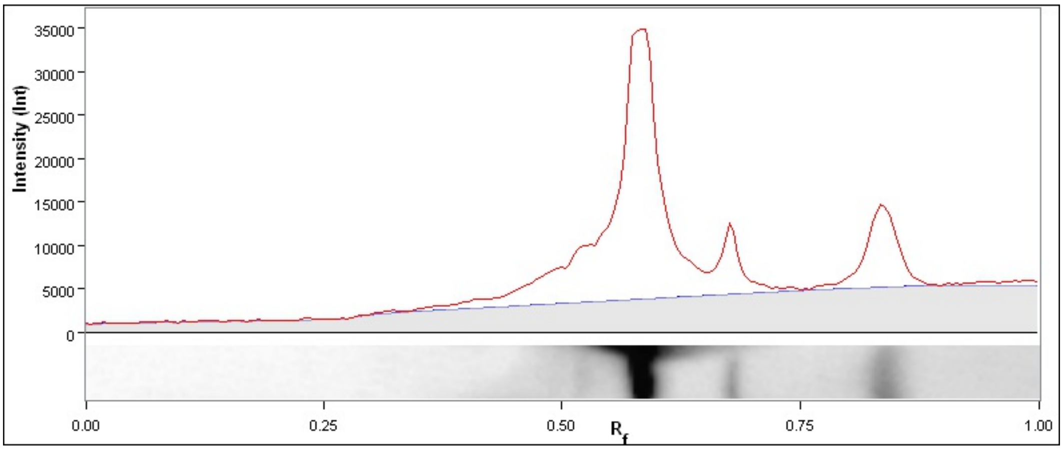

Band No.	Band Label	Mol. Wt. (KDa)	Relative Front	Volume (Int)	Abs. Quant.	Rel. Quant.	Band %	Lane %

Lane Background	Lane background subtracted with disk size: 10
Lane Width	4.30 mm
Regression Equation	A single equation is not available for this method

Lane 11

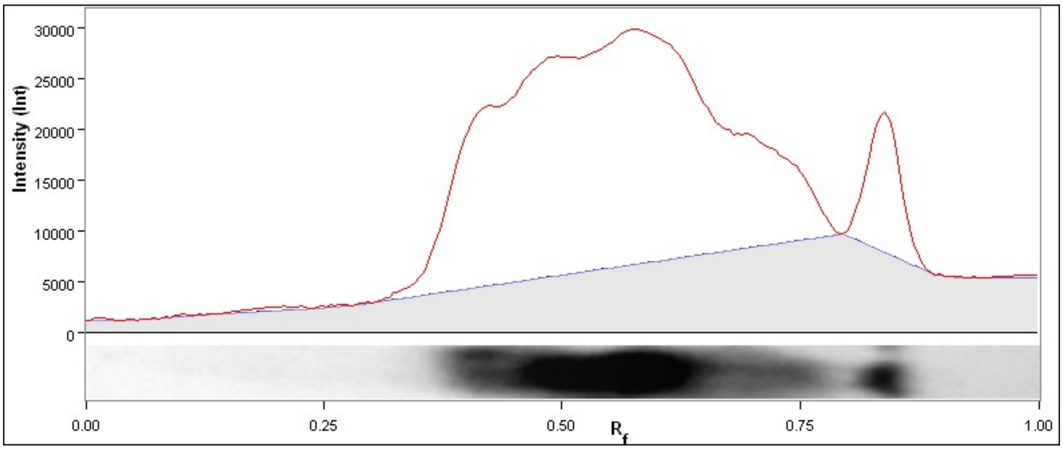

Band No.	Band Label	Mol. Wt. (KDa)	Relative Front	Volume (Int)	Abs. Quant.	Rel. Quant.	Band %	Lane %

Lane Background	Lane background subtracted with disk size: 10
Lane Width	4.30 mm
Regression Equation	A single equation is not available for this method

Lane 12

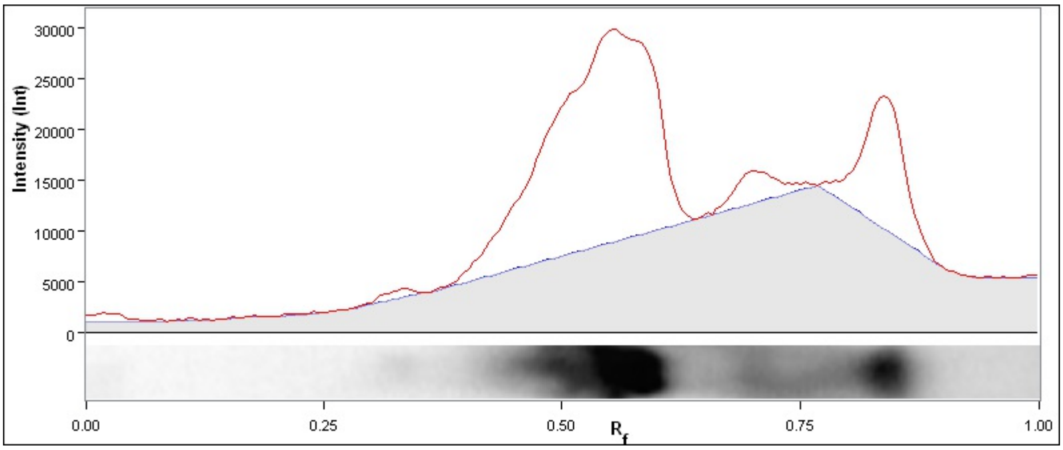

Band No.	Band Label	Mol. Wt. (KDa)	Relative Front	Volume (Int)	Abs. Quant.	Rel. Quant.	Band %	Lane %

Lane Background	Lane background subtracted with disk size: 10
Lane Width	3.28 mm
Regression Equation	A single equation is not available for this method

Lane 13

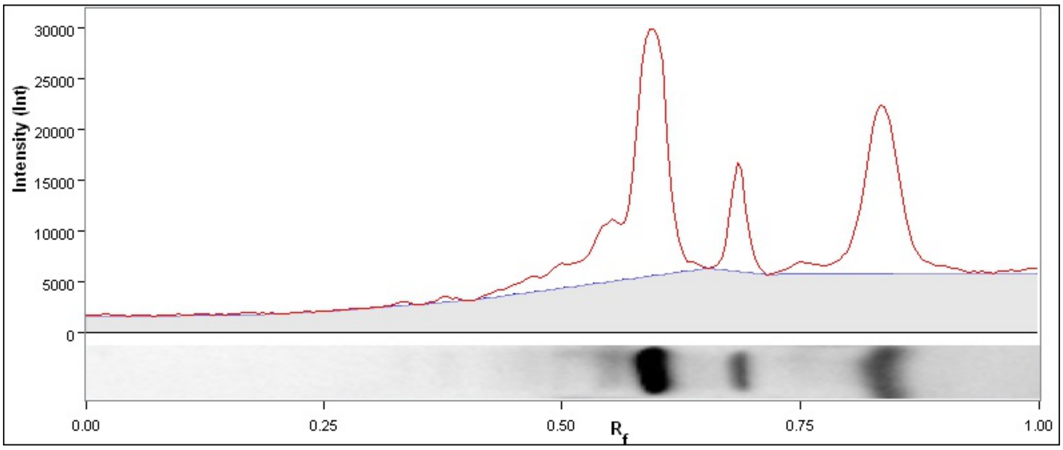

Band No.	Band Label	Mol. Wt. (KDa)	Relative Front	Volume (Int)	Abs. Quant.	Rel. Quant.	Band %	Lane %

Lane Background	Lane background subtracted with disk size: 10
Lane Width	5.73 mm
Regression Equation	A single equation is not available for this method

Lane 14

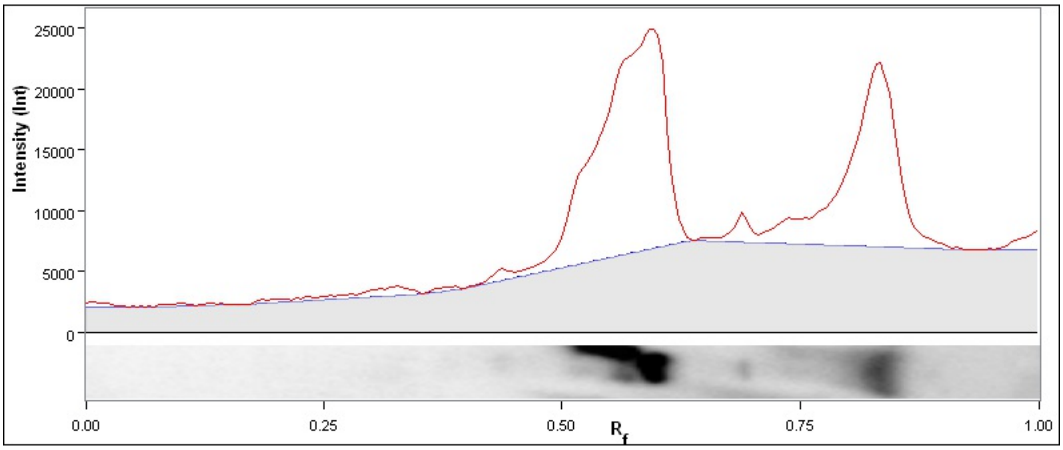

Band No.	Band Label	Mol. Wt. (KDa)	Relative Front	Volume (Int)	Abs. Quant.	Rel. Quant.	Band %	Lane %

Lane Background	Lane background subtracted with disk size: 10
Lane Width	4.91 mm
Regression Equation	A single equation is not available for this method

Lane 15

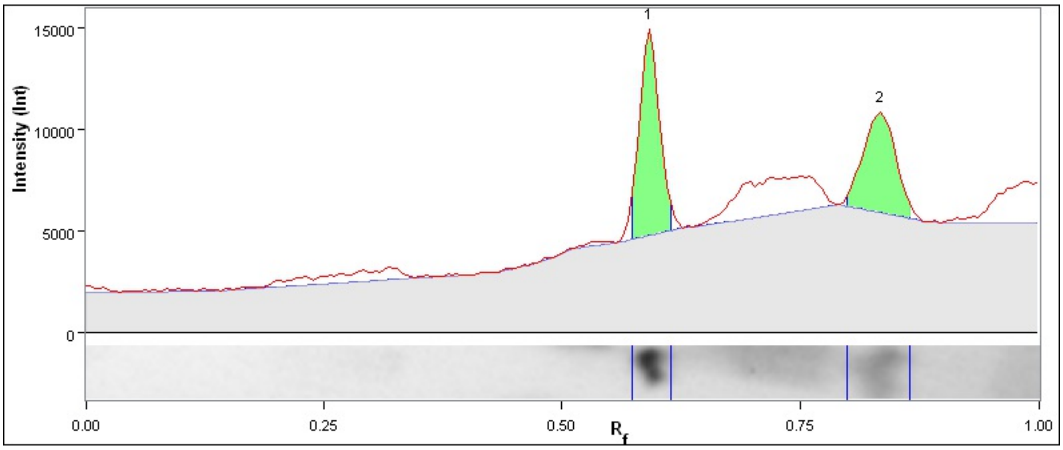

Band No.	Band Label	Mol. Wt. (KDa)	Relative Front	Volume (Int)	Abs. Quant.	Rel. Quant.	Band %	Lane %
1		32,2	0,595	1.346.758	N/A	N/A	55,1	29,5
2		17,2	0,837	1.098.447	N/A	N/A	44,9	24,1

Lane Background	Lane background subtracted with disk size: 10
Lane Width	3.89 mm
Regression Equation	A single equation is not available for this method
