# Supplementary material for: Short-chain fructo-oligosaccharides supplementation to suckling piglets: Assessment of pre- and post-weaning performance and gut health
Source: PLoS One. 2020 Jun 5;15(6):e0233910. doi: 10.1371/journal.pone.0233910 (PMC7274435; doi:10.1371/journal.pone.0233910)
Supplement: S14 Data — (PDF) [file pone.0233910.s016.pdf]

Image Report: PCNA_CASP3-BActin15_LADDER+PCNA_CASP3-BActin14_analyse7-8-9-3-6-10-1-14-11-13-2dd

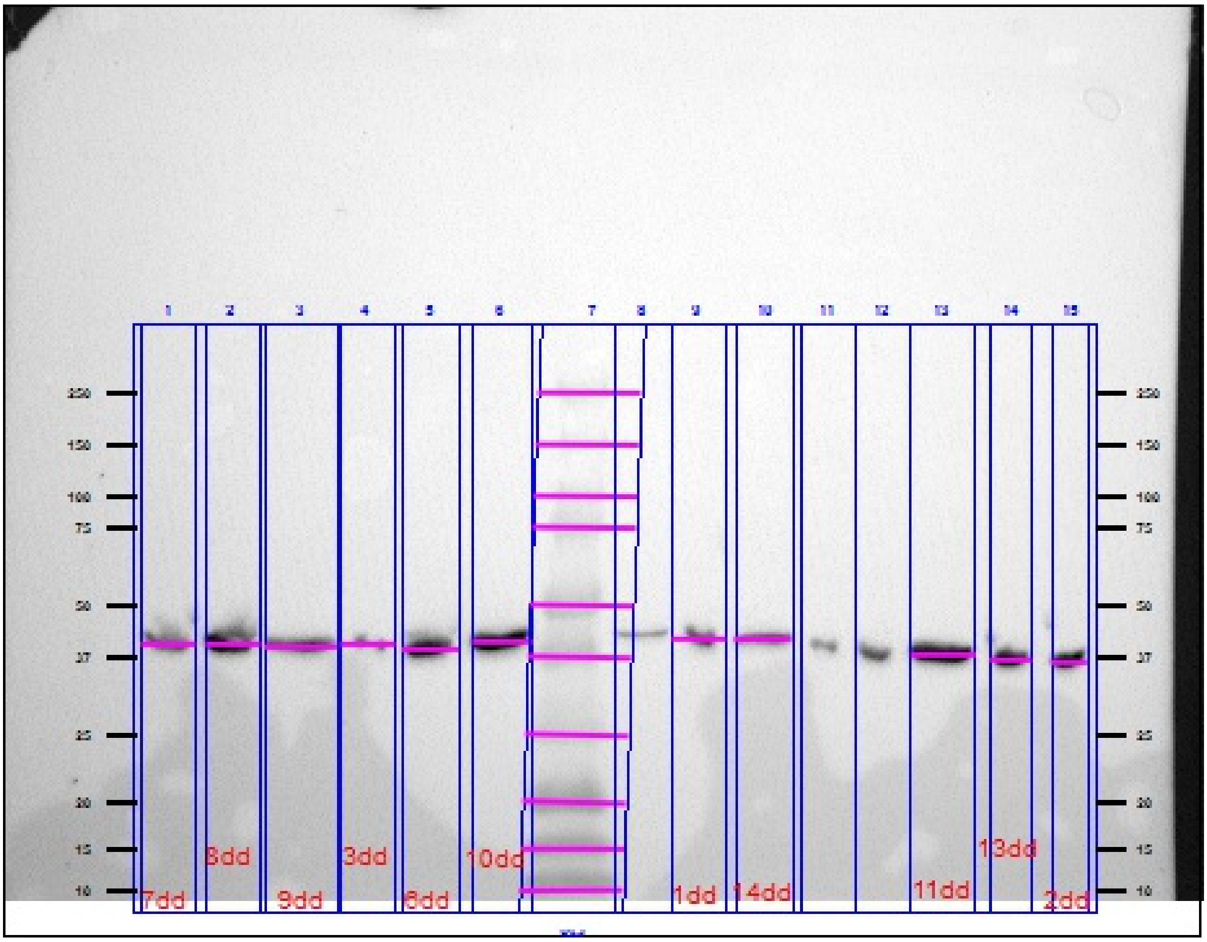

Acquisition Information

Imager	Merged Image

Image Information

Acquisition Date	27/04/2017 10:12:38
User Name	Bio-Rad
Image Area (mm)	X: 95.0 Y: 71.0
Pixel Size (um)	X: 204.7 Y: 205.1
Data Range (Int)	0 - 38456

Notes

Merged images:
Image 1: PCNA_CASP3-BActin15_LADDER
Image 2: PCNA_CASP3-BActin14_analyse7-8-9-3-6-10-1-14-11-13-2dd

Analysis Settings

Detection	Lane detection:

	Manually created lanes<br><br>Band detection:<br><br>Manually adjusted bands<br><br>Lane Background Subtraction:<br>Lane background subtracted with disk size: 10<br><br>Lane width: Variable
Mol. Weight Analysis	Standard: Bio-Rad Precision Plus<br>Standard lanes: 7<br>Regression method: Point to Point (semi-log)

Lane And Band Analysis

Lane 1

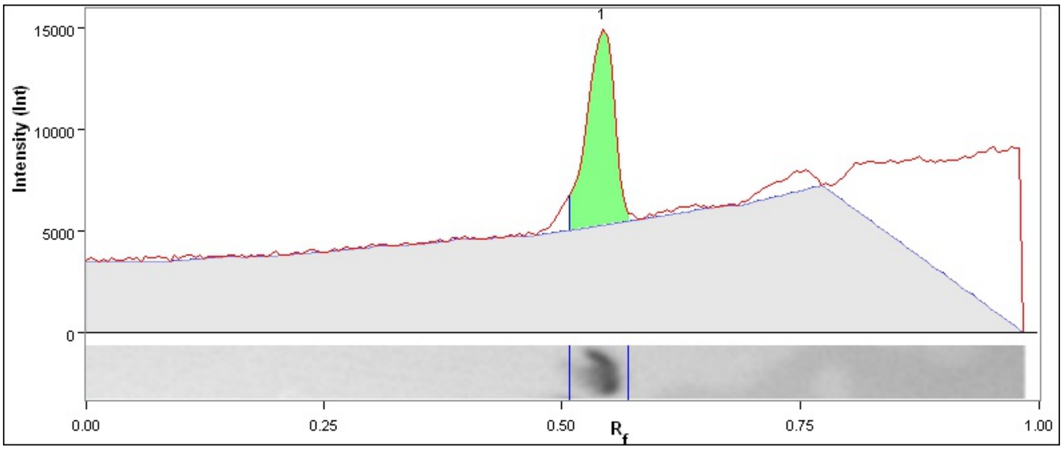

Band No.	Band Label	Mol. Wt. (KDa)	Relative Front	Volume (Int)	Abs. Quant.	Rel. Quant.	Band %	Lane %
1		39,9	0,546	1.913.982	N/A	N/A	100,0	22,3

Lane Background	Lane background subtracted with disk size: 10
Lane Width	4.30 mm
Regression Equation	A single equation is not available for this method

Lane 2

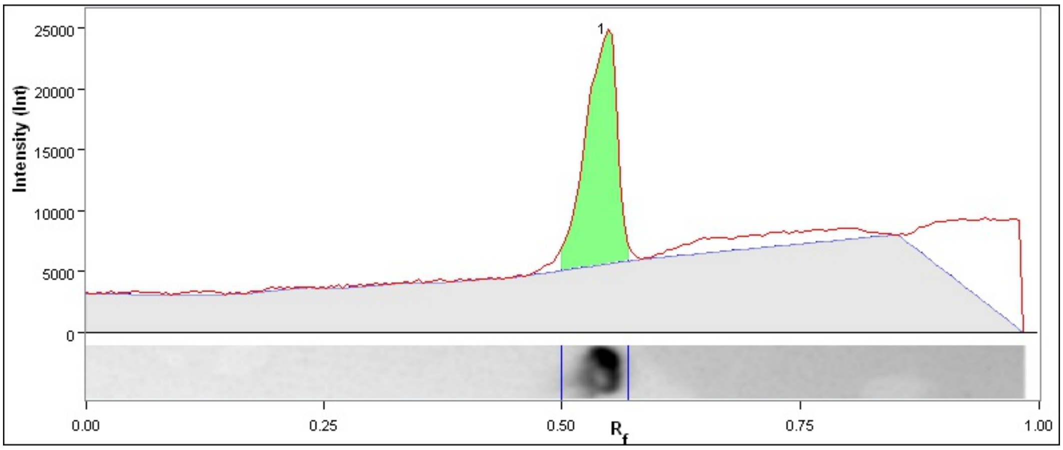

Band No.	Band Label	Mol. Wt. (KDa)	Relative Front	Volume (Int)	Abs. Quant.	Rel. Quant.	Band %	Lane %
1		39,9	0,546	3.945.669	N/A	N/A	100,0	44,0

Lane Background	Lane background subtracted with disk size: 10
Lane Width	4.30 mm

Regression Equation	A single equation is not available for this method

Lane 3

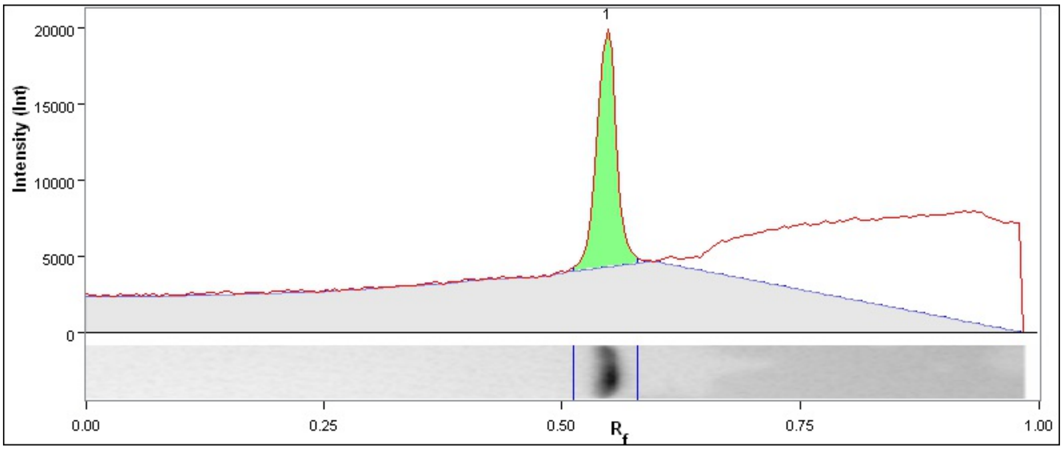

Band No.	Band Label	Mol. Wt. (KDa)	Relative Front	Volume (Int)	Abs. Quant.	Rel. Quant.	Band %	Lane %
1		39,3	0,551	2.895.648	N/A	N/A	100,0	17,3

Lane Background	Lane background subtracted with disk size: 10
Lane Width	5.73 mm
Regression Equation	A single equation is not available for this method

Lane 4

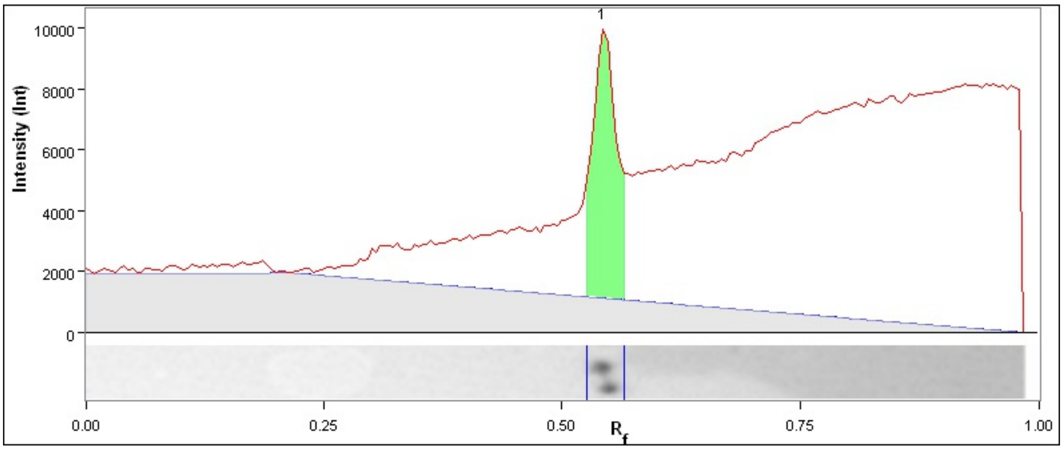

Band No.	Band Label	Mol. Wt. (KDa)	Relative Front	Volume (Int)	Abs. Quant.	Rel. Quant.	Band %	Lane %
1		39,9	0,546	1.376.214	N/A	N/A	100,0	7,4

Lane Background	Lane background subtracted with disk size: 10
Lane Width	4.30 mm
Regression Equation	A single equation is not available for this method

Lane 5

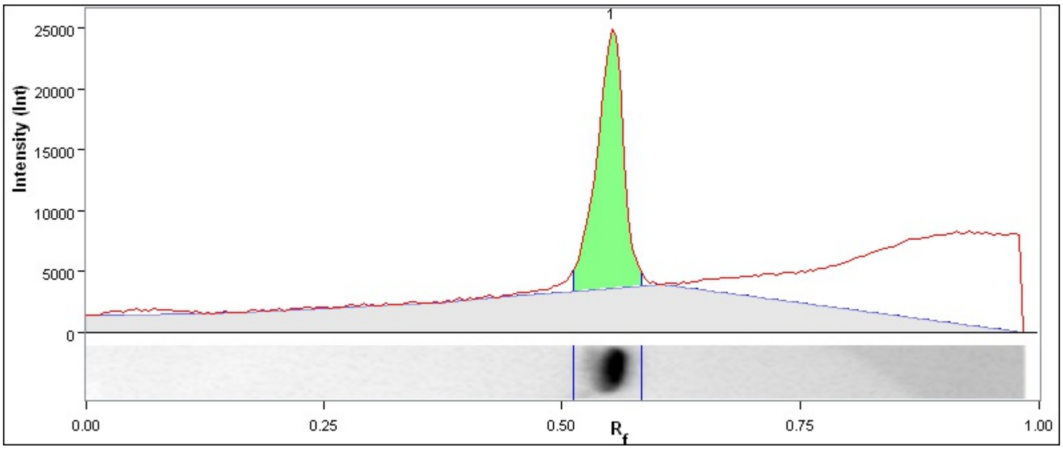

Band No.	Band Label	Mol. Wt. (KDa)	Relative Front	Volume (Int)	Abs. Quant.	Rel. Quant.	Band %	Lane %
1		38,7	0,555	3.896.442	N/A	N/A	100,0	29,9

Lane Background	Lane background subtracted with disk size: 10
Lane Width	4.50 mm
Regression Equation	A single equation is not available for this method

Lane 6

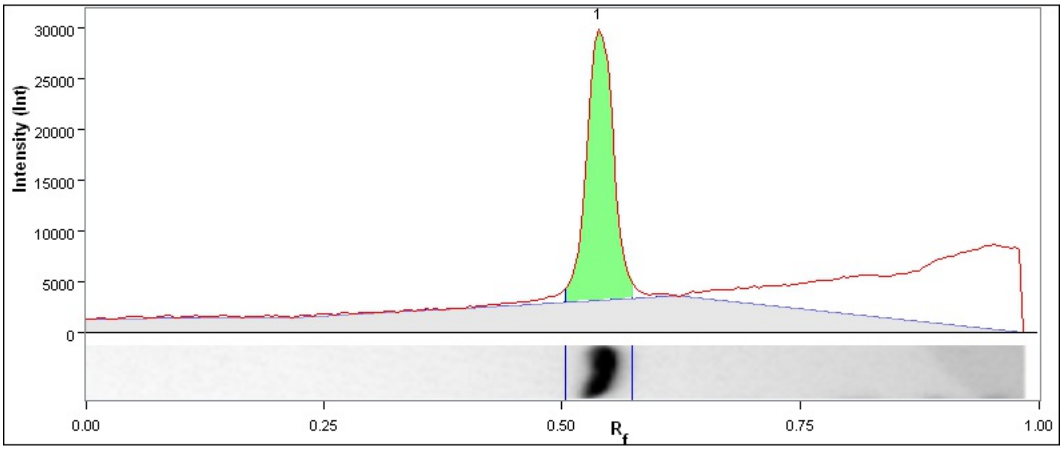

Band No.	Band Label	Mol. Wt. (KDa)	Relative Front	Volume (Int)	Abs. Quant.	Rel. Quant.	Band %	Lane %
1		40,5	0,542	4.921.977	N/A	N/A	100,0	36,7

Lane Background	Lane background subtracted with disk size: 10
Lane Width	4.71 mm
Regression Equation	A single equation is not available for this method

Lane 7 - Bio-Rad Precision Plus

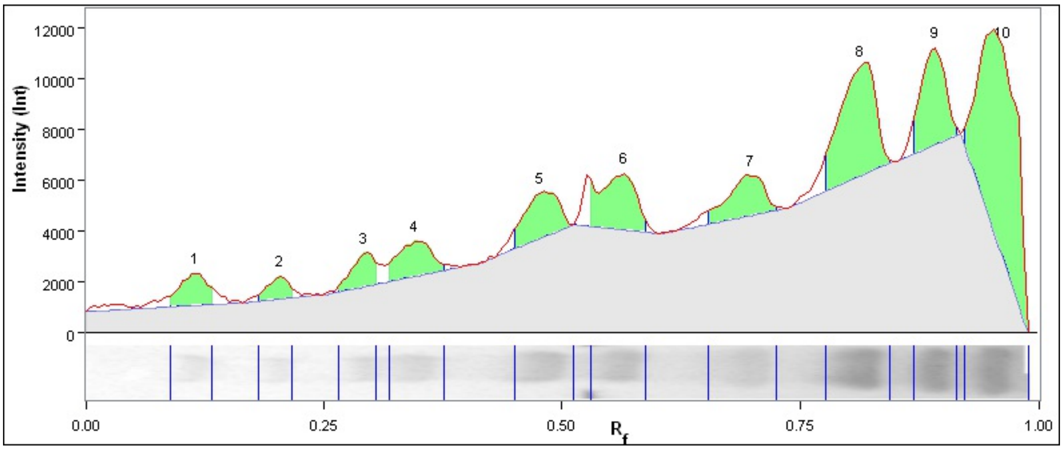

Band No.	Band Label	Mol. Wt. (KDa)	Relative Front	Volume (Int)	Abs. Quant.	Rel. Quant.	Band %	Lane %
1		250,0	0,119	424.880	N/A	N/A	4,1	3,7
2		150,0	0,207	229.760	N/A	N/A	2,2	2,0
3		100,0	0,295	364.280	N/A	N/A	3,5	3,2
4		75,0	0,348	575.200	N/A	N/A	5,5	5,0
5		50,0	0,480	732.080	N/A	N/A	7,0	6,3
6		37,0	0,568	888.680	N/A	N/A	8,5	7,7
7		25,0	0,700	679.840	N/A	N/A	6,5	5,9
8		20,0	0,815	1.818.800	N/A	N/A	17,5	15,8
9		15,0	0,894	1.048.840	N/A	N/A	10,1	9,1
10		10,0	0,965	3.657.840	N/A	N/A	35,1	31,7

Lane Background	Lane background subtracted with disk size: 10
Lane Width	8.19 mm
Regression Equation	A single equation is not available for this method

Lane 8

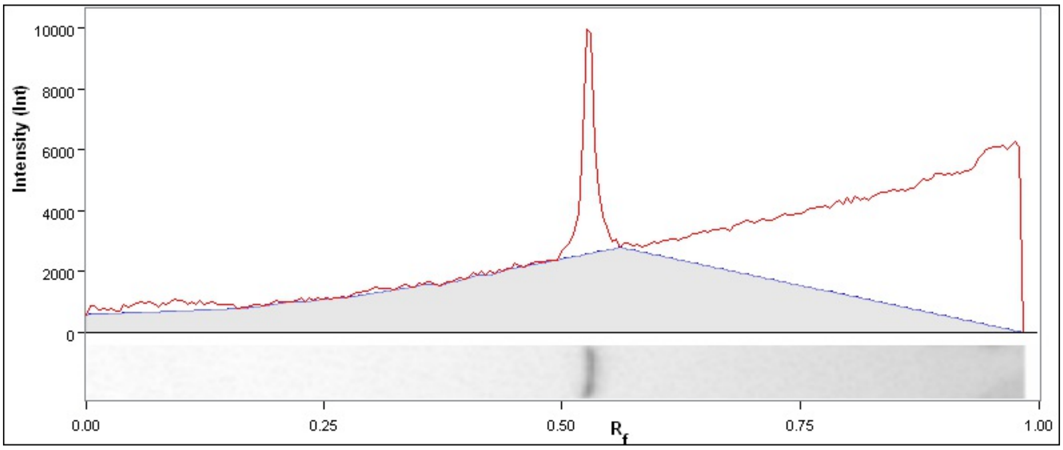

Band No.	Band Label	Mol. Wt. (KDa)	Relative Front	Volume (Int)	Abs. Quant.	Rel. Quant.	Band %	Lane %

Lane Background	Lane background subtracted with disk size: 10
Lane Width	4.50 mm
Regression Equation	A single equation is not available for this method

Lane 9

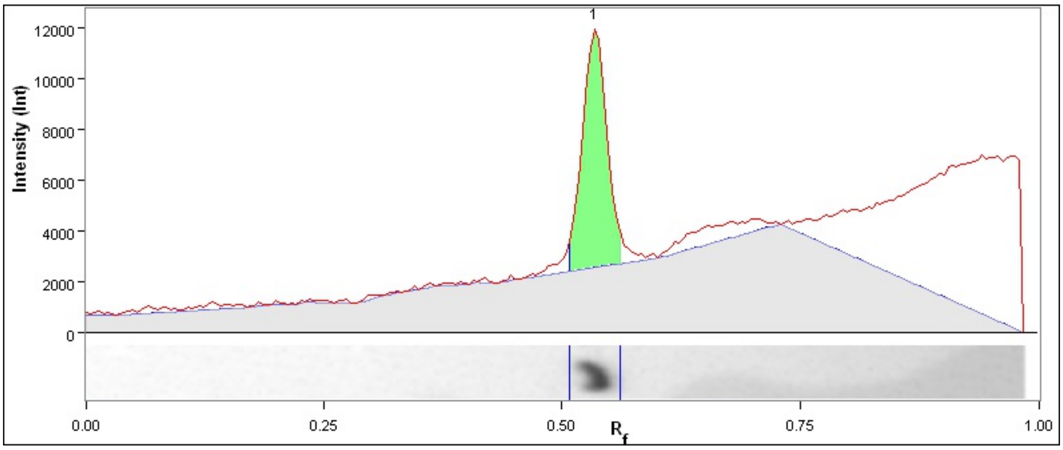

Band No.	Band Label	Mol. Wt. (KDa)	Relative Front	Volume (Int)	Abs. Quant.	Rel. Quant.	Band %	Lane %
1		41,1	0,537	1.577.520	N/A	N/A	100,0	22,1

Lane Background	Lane background subtracted with disk size: 10
Lane Width	4.30 mm
Regression Equation	A single equation is not available for this method

Lane 10

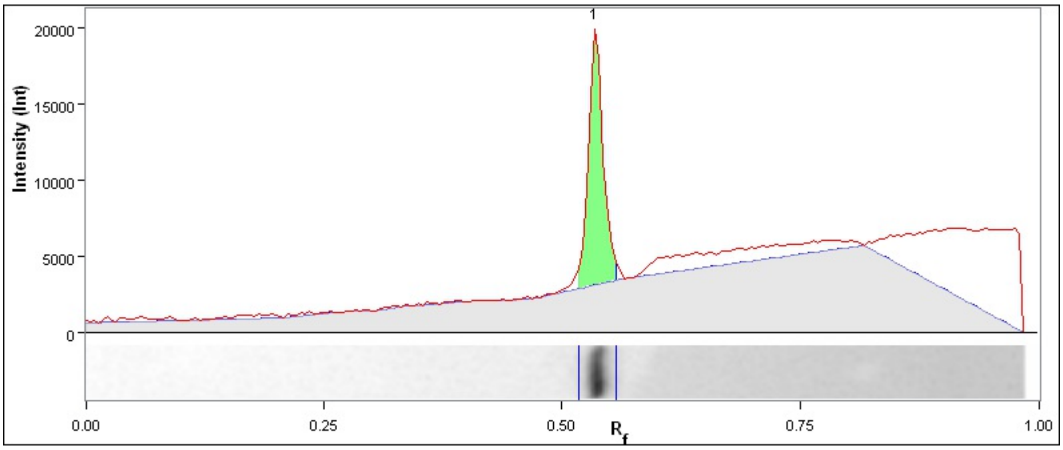

Band No.	Band Label	Mol. Wt. (KDa)	Relative Front	Volume (Int)	Abs. Quant.	Rel. Quant.	Band %	Lane %
1		41,1	0,537	1.805.210	N/A	N/A	100,0	26,6

Lane Background	Lane background subtracted with disk size: 10
Lane Width	4.50 mm
Regression Equation	A single equation is not available for this method

Lane 11

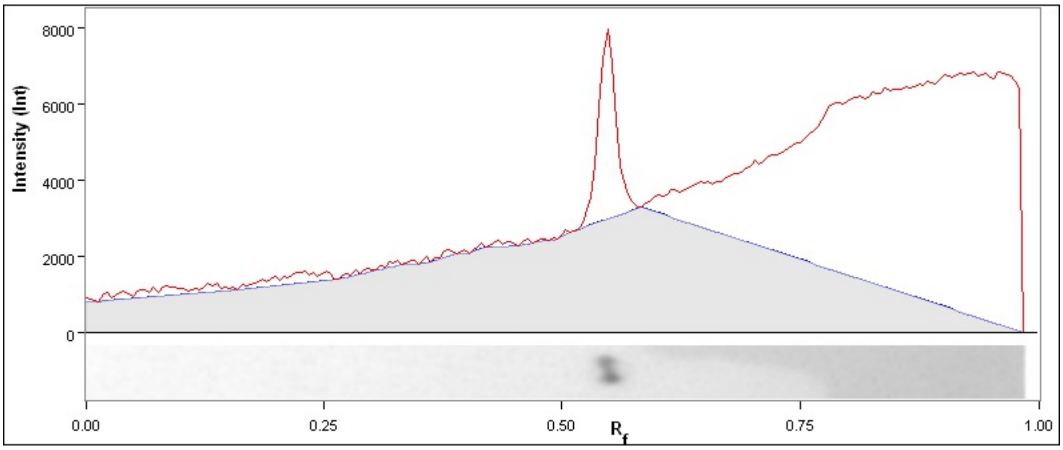

Band No.	Band Label	Mol. Wt. (KDa)	Relative Front	Volume (Int)	Abs. Quant.	Rel. Quant.	Band %	Lane %

Lane Background	Lane background subtracted with disk size: 10
Lane Width	4.30 mm
Regression Equation	A single equation is not available for this method

Lane 12

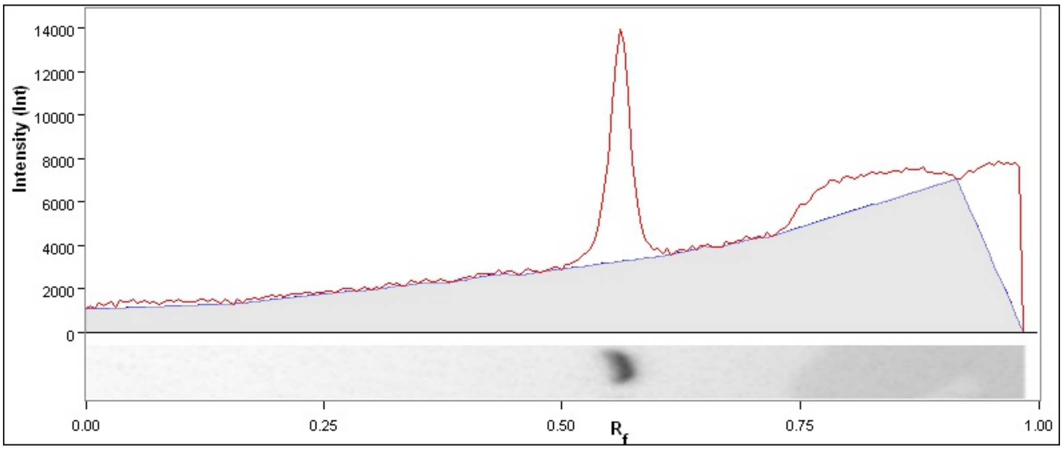

Band No.	Band Label	Mol. Wt. (KDa)	Relative Front	Volume (Int)	Abs. Quant.	Rel. Quant.	Band %	Lane %

Lane Background	Lane background subtracted with disk size: 10
Lane Width	4.30 mm
Regression Equation	A single equation is not available for this method

Lane 13

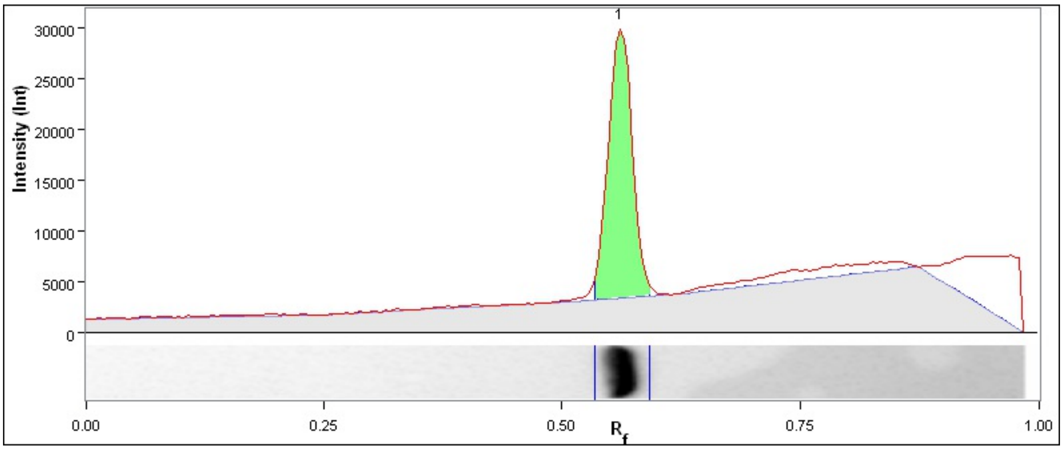

Band No.	Band Label	Mol. Wt. (KDa)	Relative Front	Volume (Int)	Abs. Quant.	Rel. Quant.	Band %	Lane %
1		37,6	0,564	5.008.625	N/A	N/A	100,0	54,0

Lane Background	Lane background subtracted with disk size: 10
Lane Width	5.12 mm
Regression Equation	A single equation is not available for this method

Lane 14

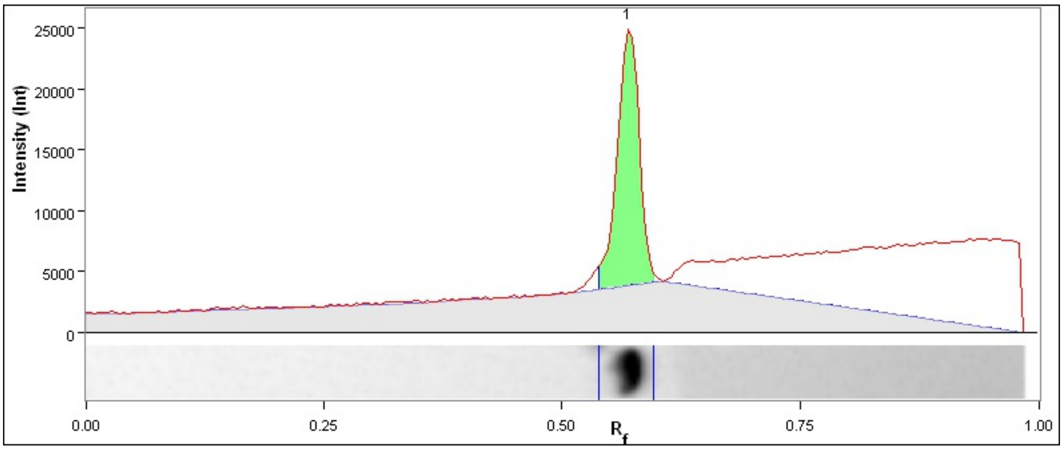

Band No.	Band Label	Mol. Wt. (KDa)	Relative Front	Volume (Int)	Abs. Quant.	Rel. Quant.	Band %	Lane %
1		36,5	0,573	2.367.776	N/A	N/A	100,0	24,1

Lane Background	Lane background subtracted with disk size: 10
Lane Width	3.28 mm
Regression Equation	A single equation is not available for this method

Lane 15

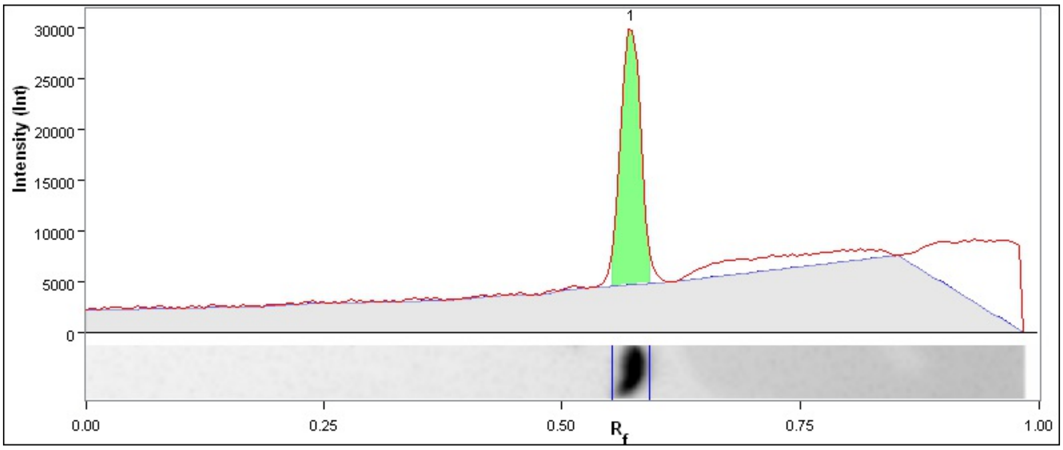

Band No.	Band Label	Mol. Wt. (KDa)	Relative Front	Volume (Int)	Abs. Quant.	Rel. Quant.	Band %	Lane %
1		36,0	0,577	1.975.456	N/A	N/A	100,0	38,9

Lane Background	Lane background subtracted with disk size: 10
Lane Width	2.87 mm
Regression Equation	A single equation is not available for this method
