# Supplementary material for: Short-chain fructo-oligosaccharides supplementation to suckling piglets: Assessment of pre- and post-weaning performance and gut health
Source: PLoS One. 2020 Jun 5;15(6):e0233910. doi: 10.1371/journal.pone.0233910 (PMC7274435; doi:10.1371/journal.pone.0233910)
Supplement: S15 Data — (PDF) [file pone.0233910.s017.pdf]

Image Report: PCNA_CASP3-BActin13_LADDER+PCNA_CASP3-BActin13_analyse16-4-5dd

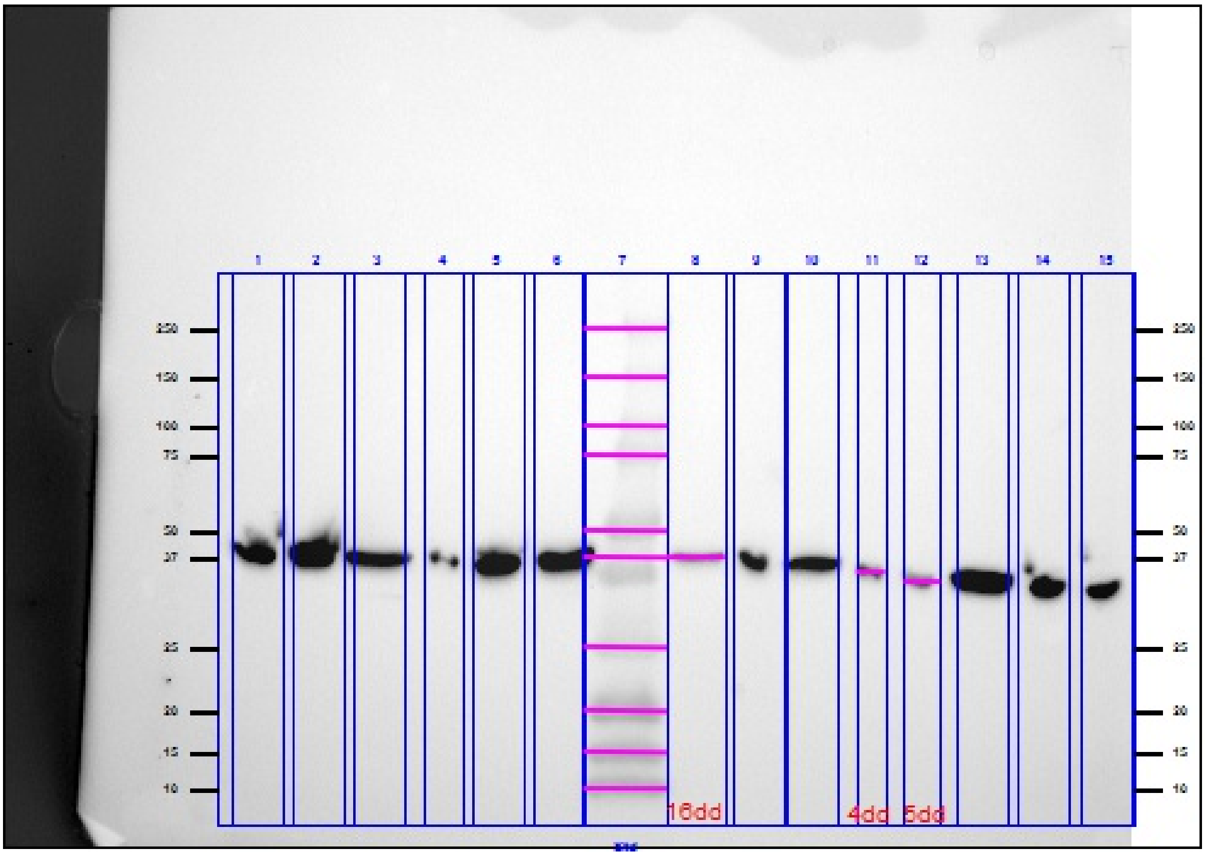

Acquisition Information

Imager	Merged Image

Image Information

Acquisition Date	26/04/2017 15:54:20
User Name	Bio-Rad
Image Area (mm)	X: 95.0 Y: 71.0
Pixel Size (um)	X: 204.7 Y: 205.1
Data Range (Int)	4 - 49170

Notes

Merged images:
Image 1: PCNA_CASP3-BActin13_LADDER
Image 2: PCNA_CASP3-BActin13_analyse16-4-5dd

Analysis Settings

Detection	Lane detection:<br>Manually created lanes<br><br>Band detection:

	Manually adjusted bands<br><br>Lane Background Subtraction:<br>Lane background subtracted with disk size: 10<br><br>Lane width: Variable
Mol. Weight Analysis	Standard: Bio-Rad Precision Plus<br>Standard lanes: 7<br>Regression method: Point to Point (semi-log)

Lane And Band Analysis

Lane 1

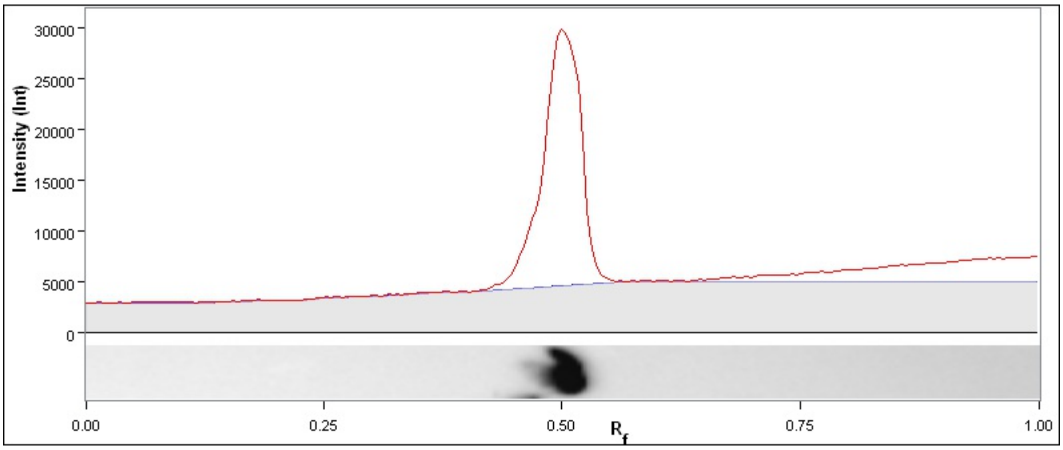

Band No.	Band Label	Mol. Wt. (KDa)	Relative Front	Volume (Int)	Abs. Quant.	Rel. Quant.	Band %	Lane %

Lane Background	Lane background subtracted with disk size: 10
Lane Width	4.30 mm
Regression Equation	A single equation is not available for this method

Lane 2

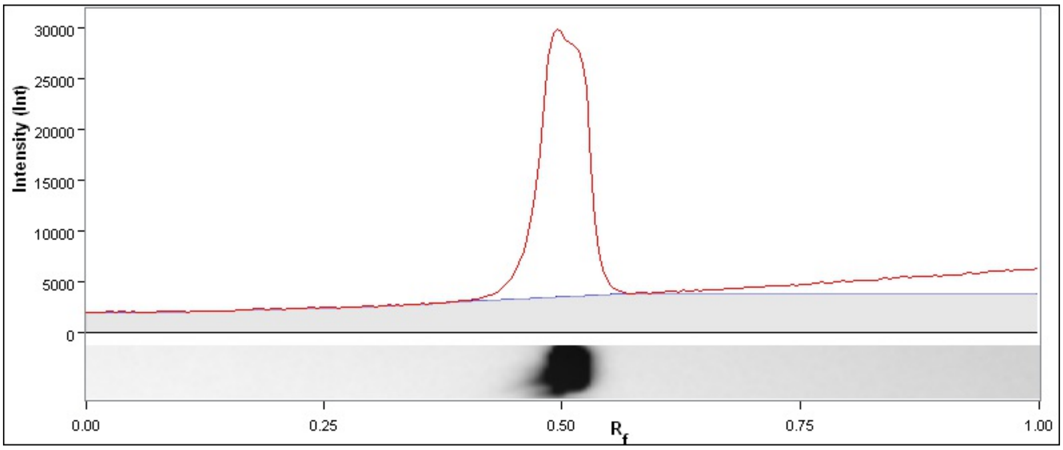

Band No.	Band Label	Mol. Wt. (KDa)	Relative Front	Volume (Int)	Abs. Quant.	Rel. Quant.	Band %	Lane %

Lane Background	Lane background subtracted with disk size: 10
Lane Width	4.30 mm
Regression Equation	A single equation is not available for this method

Lane 3

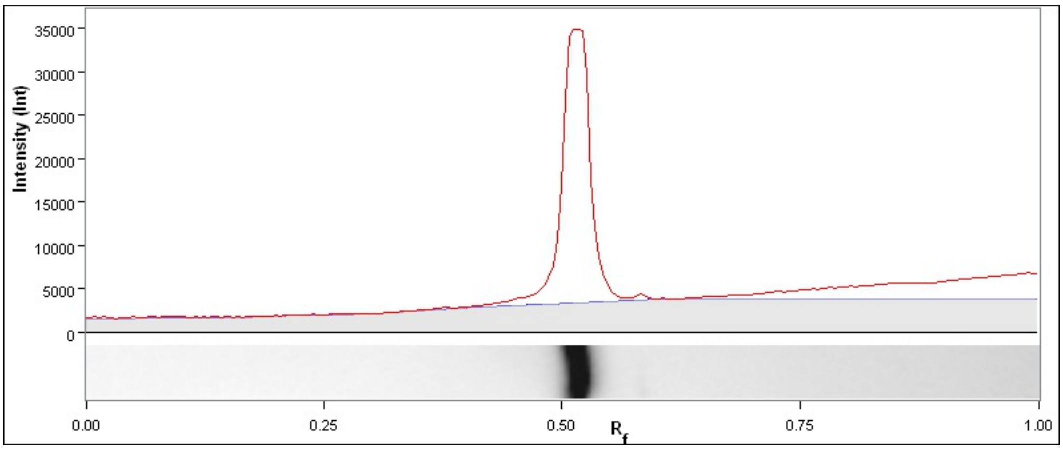

Band No.	Band Label	Mol. Wt. (KDa)	Relative Front	Volume (Int)	Abs. Quant.	Rel. Quant.	Band %	Lane %

Lane Background	Lane background subtracted with disk size: 10
Lane Width	4.30 mm
Regression Equation	A single equation is not available for this method

Lane 4

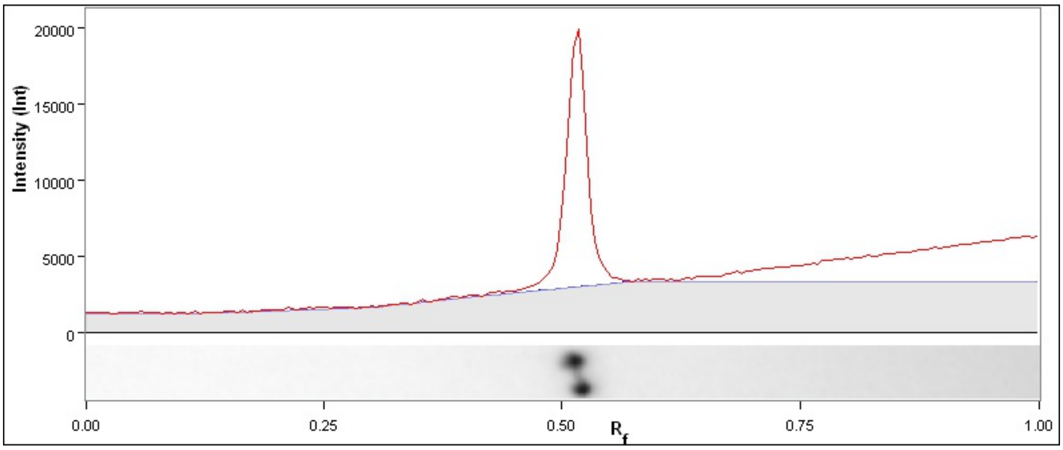

Band No.	Band Label	Mol. Wt. (KDa)	Relative Front	Volume (Int)	Abs. Quant.	Rel. Quant.	Band %	Lane %

Lane Background	Lane background subtracted with disk size: 10
Lane Width	3.28 mm
Regression Equation	A single equation is not available for this method

Lane 5

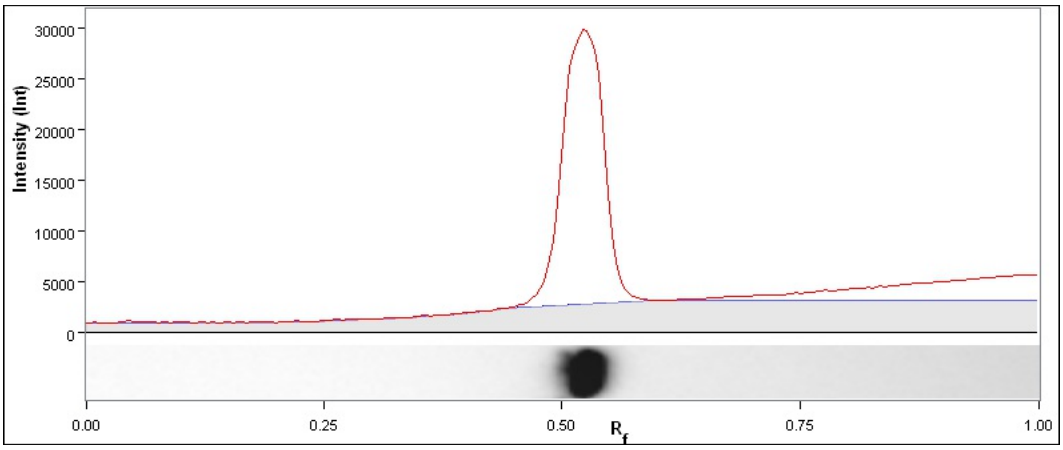

Band No.	Band Label	Mol. Wt. (KDa)	Relative Front	Volume (Int)	Abs. Quant.	Rel. Quant.	Band %	Lane %

Lane Background	Lane background subtracted with disk size: 10
Lane Width	4.30 mm
Regression Equation	A single equation is not available for this method

Lane 6

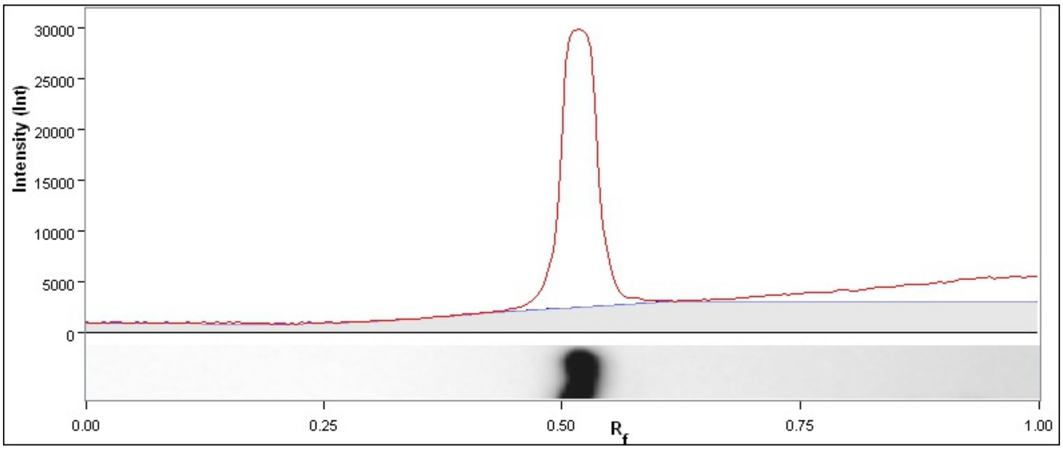

Band No.	Band Label	Mol. Wt. (KDa)	Relative Front	Volume (Int)	Abs. Quant.	Rel. Quant.	Band %	Lane %

Lane Background	Lane background subtracted with disk size: 10
Lane Width	4.30 mm
Regression Equation	A single equation is not available for this method

Lane 7 - Bio-Rad Precision Plus

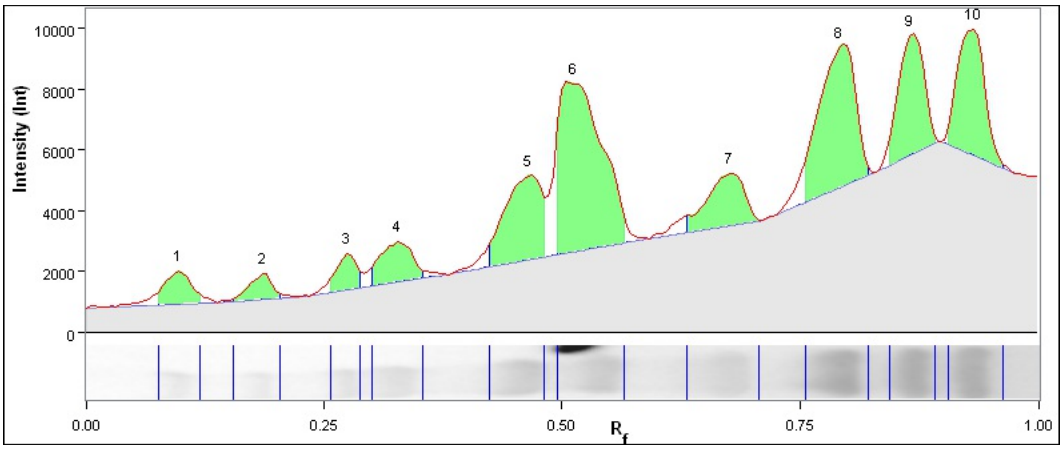

Band No.	Band Label	Mol. Wt. (KDa)	Relative Front	Volume (Int)	Abs. Quant.	Rel. Quant.	Band %	Lane %
1		250,0	0,101	328.335	N/A	N/A	3,2	2,9
2		150,0	0,189	230.755	N/A	N/A	2,3	2,0
3		100,0	0,278	260.225	N/A	N/A	2,5	2,3
4		75,0	0,330	471.555	N/A	N/A	4,6	4,2
5		50,0	0,467	1.214.290	N/A	N/A	11,9	10,8
6		37,0	0,515	2.639.245	N/A	N/A	25,8	23,4
7		25,0	0,678	731.290	N/A	N/A	7,1	6,5
8		20,0	0,793	1.911.315	N/A	N/A	18,7	16,9
9		15,0	0,868	1.141.350	N/A	N/A	11,1	10,1
10		10,0	0,934	1.310.435	N/A	N/A	12,8	11,6

Lane Background	Lane background subtracted with disk size: 10
Lane Width	7.17 mm
Regression Equation	A single equation is not available for this method

Lane 8

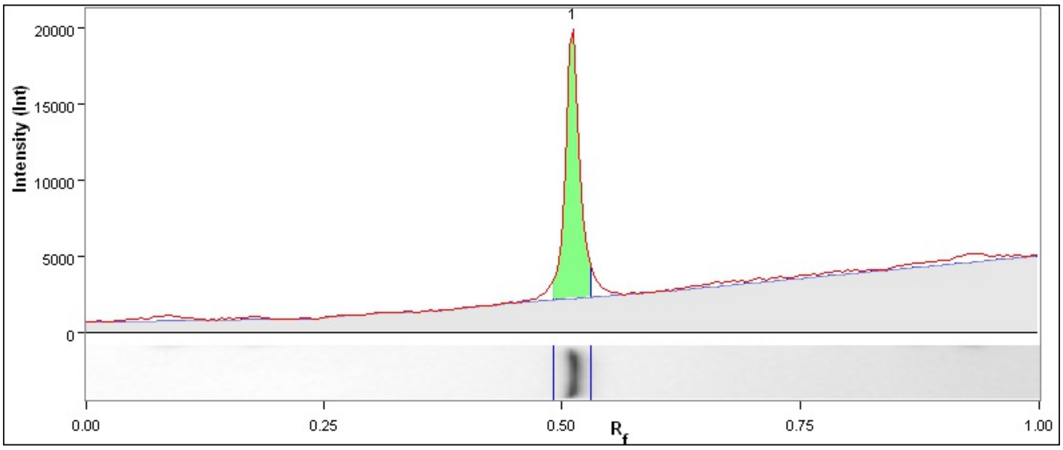

Band No.	Band Label	Mol. Wt. (KDa)	Relative Front	Volume (Int)	Abs. Quant.	Rel. Quant.	Band %	Lane %
1		37,0	0,515	1.899.240	N/A	N/A	100,0	68,4

Lane Background	Lane background subtracted with disk size: 10
Lane Width	4.91 mm
Regression Equation	A single equation is not available for this method

Lane 9

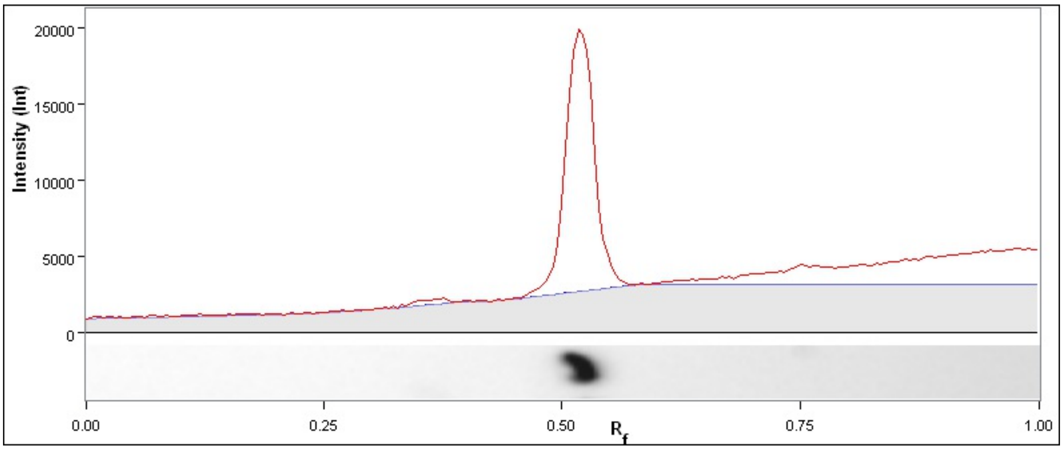

Band No.	Band Label	Mol. Wt. (KDa)	Relative Front	Volume (Int)	Abs. Quant.	Rel. Quant.	Band %	Lane %

Lane Background	Lane background subtracted with disk size: 10
Lane Width	4.30 mm
Regression Equation	A single equation is not available for this method

Lane 10

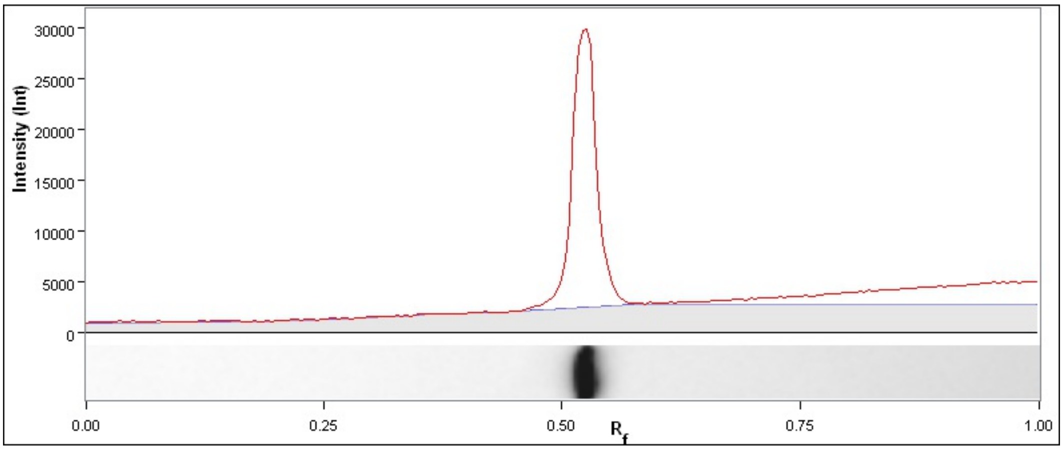

Band No.	Band Label	Mol. Wt. (KDa)	Relative Front	Volume (Int)	Abs. Quant.	Rel. Quant.	Band %	Lane %

Lane Background	Lane background subtracted with disk size: 10
Lane Width	4.30 mm
Regression Equation	A single equation is not available for this method

Lane 11

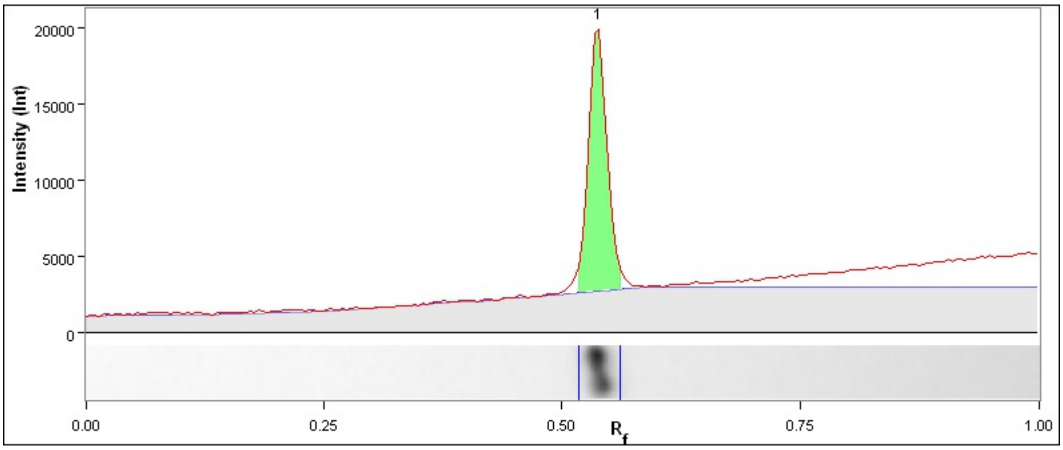

Band No.	Band Label	Mol. Wt. (KDa)	Relative Front	Volume (Int)	Abs. Quant.	Rel. Quant.	Band %	Lane %
1		34,7	0,542	1.259.424	N/A	N/A	100,0	43,9

Lane Background	Lane background subtracted with disk size: 10
Lane Width	2.46 mm
Regression Equation	A single equation is not available for this method

Lane 12

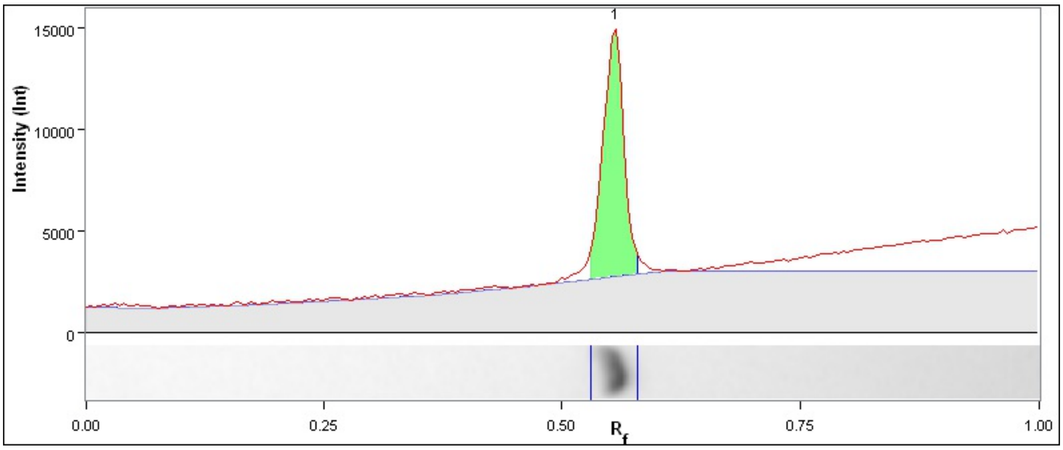

Band No.	Band Label	Mol. Wt. (KDa)	Relative Front	Volume (Int)	Abs. Quant.	Rel. Quant.	Band %	Lane %
1		33,3	0,559	1.304.775	N/A	N/A	100,0	40,0

Lane Background	Lane background subtracted with disk size: 10
Lane Width	3.07 mm
Regression Equation	A single equation is not available for this method

Lane 13

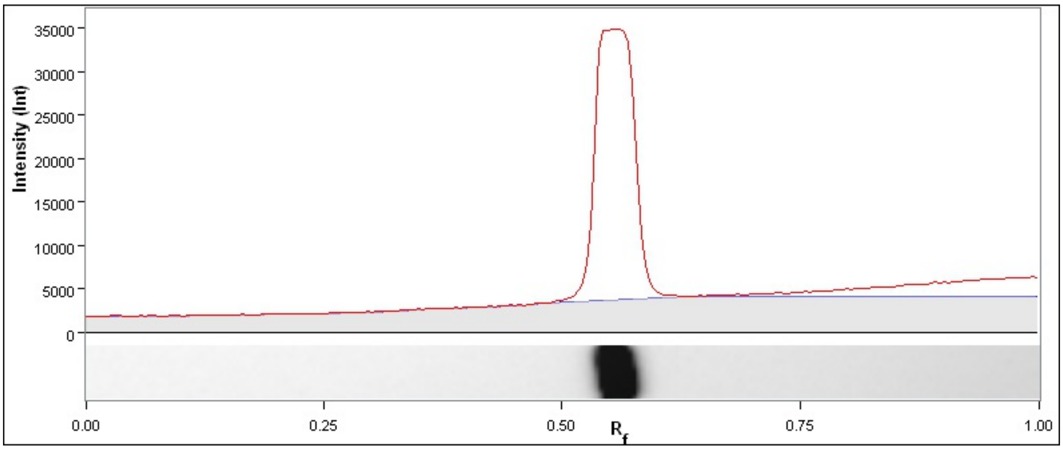

Band No.	Band Label	Mol. Wt. (KDa)	Relative Front	Volume (Int)	Abs. Quant.	Rel. Quant.	Band %	Lane %

Lane Background	Lane background subtracted with disk size: 10
Lane Width	4.30 mm
Regression Equation	A single equation is not available for this method

Lane 14

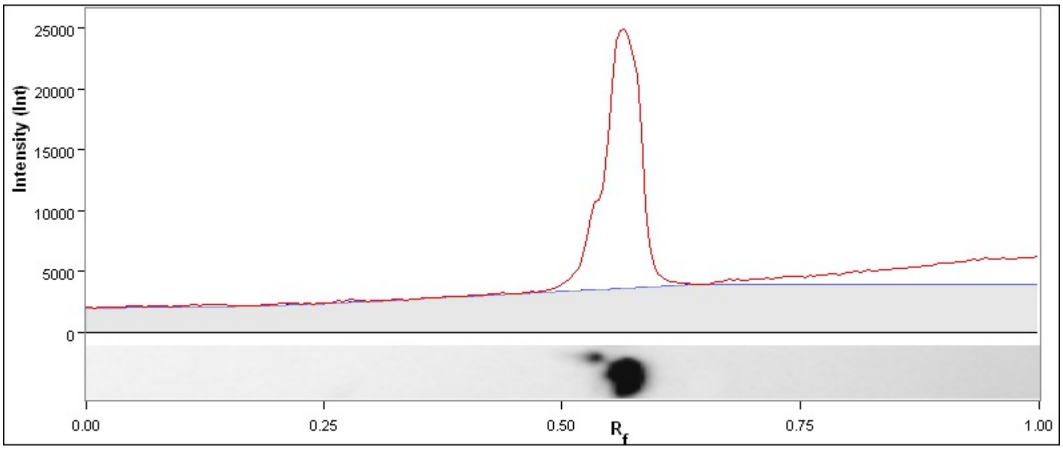

Band No.	Band Label	Mol. Wt. (KDa)	Relative Front	Volume (Int)	Abs. Quant.	Rel. Quant.	Band %	Lane %

Lane Background	Lane background subtracted with disk size: 10
Lane Width	4.30 mm
Regression Equation	A single equation is not available for this method

Lane 15

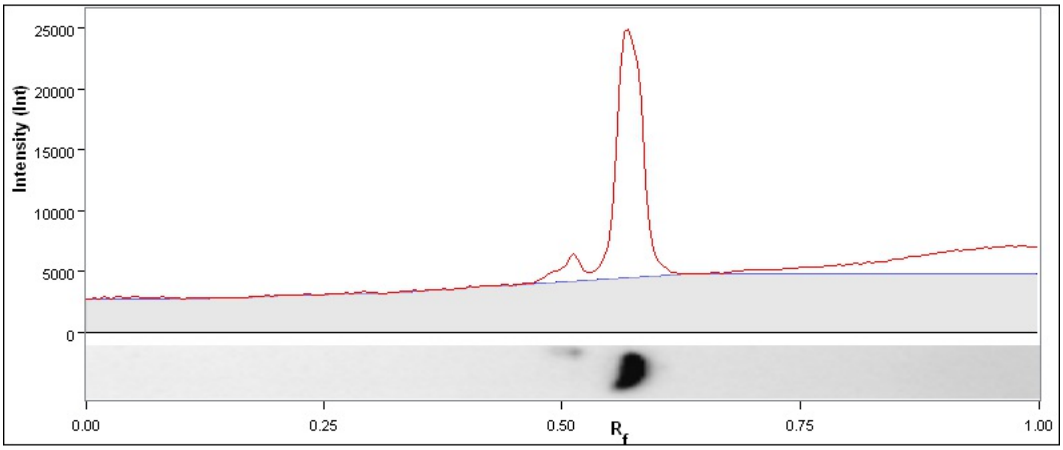

Band No.	Band Label	Mol. Wt. (KDa)	Relative Front	Volume (Int)	Abs. Quant.	Rel. Quant.	Band %	Lane %

Lane Background	Lane background subtracted with disk size: 10
Lane Width	4.30 mm
Regression Equation	A single equation is not available for this method
